# Supplementary material for: Changing Agendas on Sleep, Treatment and Learning in Epilepsy (CASTLE) Sleep-E: a protocol for a randomised controlled trial comparing an online behavioural sleep intervention with standard care in children with Rolandic epilepsy
Source: BMJ Open. 2023 Mar 10;13(3):e065769. doi: 10.1136/bmjopen-2022-065769 (PMC10008377; doi:10.1136/bmjopen-2022-065769)
Supplement: Supplementary data [file bmjopen-2022-065769supp001.pdf]

Supplemental Table 1. World Health Organization Trial Registration Data Set (Version 1.3.1) for CASTLE Sleep-E

**Data category**	**Information**
1. Primary registry and trial identifying number	ISRCTN: ISRCTN13202325
2. Date of registration in primary registry	09/September/2021
3. Secondary identifying numbers	CPMS 50413<br>RP-PG-0615-20007<br>IRAS 289580<br>21/EM/0205
4. Source(s) of monetary or material support	National Institute for Health and Care Research (NIHR)
5. Primary sponsor	Ms Jasmine Palmer<br>Research & Innovation Operational Manager<br>King's College Hospital NHS Foundation Trust<br>The Research & Innovation Office<br>First Floor, Coldharbour Works<br>245a Coldharbour Lane, Brixton<br>London SW9 8RR<br>jasmine.palmer1@nhs.net<br>+44 (0) 7790 950 219
6. Secondary sponsor(s)	Professor Reza Razavi<br>Director of Research Management &<br>Director of Administration (Health Schools)<br>Room 5.31<br>James Clerk Maxwell Building<br>57 Waterloo Road<br>London<br>SE1 8WA<br>reza.razavi@kcl.ac.uk<br>+44 (0)20 7848 3224
7. Contact for public queries	Trial Manager: Lucy Stibbs-Eaton<br>Liverpool Clinical Trials Centre<br>University of Liverpool<br>Liverpool<br>L69 3BX<br>LCTC@liverpool.ac.uk<br>+44 (0)151 795 8751
8. Contact for scientific queries	Professor Deb Pal<br>Professor of Paediatric Epilepsy<br>Maurice Wohl Clinical Neuroscience Institute<br>King's College London<br>5 Cutcombe Road<br>London<br>SE5 9RX<br>deb.pal@kcl.ac.uk<br>+44 (0) 207 848 5762
9. Public title	A trial comparing the effectiveness of an online sleep behavioural intervention versus standard care in children with rolandic epilepsy
10. Scientific title	Changing Agendas on Sleep, Treatment and Learning in Epilepsy (CASTLE) Sleep-E: A randomised controlled trial comparing an online behavioural sleep intervention with standard care in children with Rolandic epilepsy

Al-Najjar N, *et al. BMJ Open* 2023; 13:e065769. doi: 10.1136/bmjopen-2022-065769

Data category	Information
11. Countries of recruitment	England<br>Scotland<br>Wales<br>Northern Ireland
12. Health condition(s) or problem(s) studied	Sleep problems in Rolandic epilepsy also known as childhood epilepsy with centro-temporal spikes
13. Intervention(s)	**Intervention arm (SC + COSI)**: Novel, tailored, parent-led CASTLE Online Sleep Intervention (COSI) that incorporates evidence-based behavioural components. Delivered by parents to enrolled children with Rolandic epilepsy in their own homes after completion of self-paced online training. Standard care (SC) is augmented with the CASTLE Online Sleep Intervention (COSI).<br><br>**Active control arm (SC)**: UK National Health Service standard care (SC) for children with Rolandic epilepsy, which consists of a comprehensive care plan with the option of pharmacological treatment with anti-epileptic drugs (first-line mono-therapy with lamotrigine, levetiracetam, oxcarbazepine [girls and boys], carbamazepine or sodium valproate [both boys only]).
14. Key inclusion and exclusion criteria	Inclusion criteria<br>Main CASTLE Sleep-E study<br>1. Children diagnosed with RE/CECTS (see International League Against Epilepsy Diagnostic Manual at https://www.epilepsydiagnosis.org/syndrome/ects-overview.html)<br>2. EEG showing focal sharp waves with normal background (see International League Against Epilepsy Diagnostic Manual at https://www.epilepsydiagnosis.org/syndrome/ects-eeg.html)<br>3. Aged 5 to <13 years at the time of randomisation<br>4. Parent/Carer reported child sleep problem as defined by mild, moderate or severe score on Hiscock Australian global sleep question (Poor sleeper defined by caregiver responding 'Mild', 'Moderate' or 'Severe' to "Over the last 2 weeks, how much of a problem has your child's sleep been?")<br>5. Documented informed consent received from a person with parental responsibility<br>6. Family have an email address and mobile phone<br>7. Parent and child are to have a good enough understanding of the English language to read and answer study questionnaires<br>Qualitative component<br>1. Consent of care giver to participate and for their child to participate (optional item on main trial consent form)<br>2. Children need to be >=7 years of age<br><br>Exclusion criterion<br>1. Children with moderate/severe learning disability
15. Study type	Interventional<br>• Allocation: Minimisation using a bespoke LCTC system<br>Allocation concealment: Central web-interface<br>Sequence generation: Randomised, 1:1 ratio<br>Intervention model: Parallel assignment<br>• Blinding<br>Child, parent, healthcare providers, data collectors, qualitative researchers: None (open label)<br>Quantitative data analysts: Blinded<br>• Primary purpose: Clinical- and cost-effectiveness, process evaluation (qualitative trial component, COSI e-analytics and evaluation module)<br>• Phase: III (behavioural intervention)

Al-Najjar N, *et al. BMJ Open* 2023; 13:e065769. doi: 10.1136/bmjopen-2022-065769

Data category	Information
16. Date of first enrolment	24/June/2022
17. Target sample size	110 (55 children per arm)<br>Calculation based on:<br>• Achieving 90 % statistical power to detect Minimal Clinically Meaningful Difference in primary outcome<br>• 10 % expected attrition
18. Recruitment status	Recruiting<br>• First trial site opened: 12/May/2022<br>• First recruitment: 30/August/2022
19. Primary outcome(s)	• Clinical: Children's Sleep Habits Questionnaire at 3 months<br>• Health economic: Cost-effectiveness of the intervention over 6 months after randomisation, measured in terms of incremental cost per quality-adjusted life year gained (Child Health Utility instrument or EQ-5D-Y) from the perspective of the National Health Services and Personal Social Services in the UK.
20. Key secondary outcome(s)	• Clinical Outcome: Sleep problem reduction<br>Metric/method: Children's Sleep Habits Questionnaire<br>Timepoint: 6 months<br>• Clinical Outcome: Seizure frequency reduction<br>Metric/method: Time to first seizure (days)<br>Timepoint: 3 months, 6 months
21. Ethics Review	• Status: Approved<br>• Approval reference: 21/EM/0205<br>• Health Research Authority<br>East Midlands – Nottingham 1 Research Ethics Committee<br>Chair: Mr Paul Hamilton<br>+44 (0) 207 104 8115 or +44 (0) 207 104 8283<br>nottingham1.rec@hra.nhs.uk
22. Completion date	31/July/2023
23. Summary results	TBC
24. Individual patient data (IPD) sharing statement	• Plan to share IPD: Yes<br>• Plan description: At the end of the trial, after the primary results have been published, the pseudo-anonymised Individual Patient Data and associated documentation (e.g. protocol, statistical analysis plan, annotated blank case report form) will be prepared to be shared with external researchers on reasonable request.
25. Protocol version and date	• Internal protocol: V4.0, 08/December/2021<br>• Manuscript for protocol publication: V3.2, 20/December/2022

Al-Najjar N, *et al. BMJ Open* 2023; 13:e065769. doi: 10.1136/bmjopen-2022-065769

Supplemental Table 2. Composition, roles and responsibilities of the Trial Management Group, Programme Steering Committee, and Independent Data and Safety Monitoring Committee for CASTLE Sleep-E.

Role	Name (Initials)	Affiliation
**Trial management Group (TMG)**<br>**Responsibilities**: Day-to-day running and management of the trial.<br>**Meeting frequency**: Bi-weekly to three-monthly, depending on trial stage.		
1. King's College Hospital Sponsor Representative	Jasmine Palmer	King's College Hospital NHS Foundation Trust, UK
2. Chief Investigator	Deb K. Pal	King's College London, UK
3. Co-Chief Investigator	Paul Gringras	Evelina London Children's Hospital, UK
4. Co-Investigator<br>Public and Patient Involvement Lead	Lucy Bray	Edge Hill University, UK
5. Co-Investigator<br>Qualitative Research Lead<br>Public and Patient Involvement Co-Lead	Bernie Carter	Edge Hill University, UK
6. Co-Investigator<br>Health Economics Lead	Dyfrig Hughes	Bangor University, UK
7. Co-Investigator<br>Patient Reported Outcome Lead<br>Public and Patient Involvement Co-Lead	Christopher Morris	University of Exeter, UK
8. Co-Investigator<br>Lead Statistician	Catrin Tudur Smith	University of Liverpool, UK
9. Co-Investigator<br>Intervention Development Lead	Luci Wiggs	Oxford Brookes University, UK
10. Supervising Trials Manager	Catherine Spowart	University of Liverpool, UK
11. Trial Manager	Lucy Stibbs-Eaton	University of Liverpool, UK
12. Trial Statistician	Liam Whittle	University of Liverpool, UK
13. CASTLE Programme Manager	Amber Collingwood	King's College London, UK
14. Researcher	Georgia Cook	Oxford Brookes University, UK
15. Researcher	Kristina C. Dietz	King's College London, UK
16. Health economist	Will A. S. Hardy	Bangor University, UK
17. Researcher	Holly Saron	Edge Hill University, UK
**Trial Steering Committee (TSC)**<br>**Responsibilities**: Overall trial supervision and advice, ultimate decision for the continuation of the trial.<br>**Meeting frequency**: At least annually.		
1. Chair	Jeremy Parr	Newcastle University, UK
2. Medical statistician	Martyn Lewis	Keele University, UK
3. Paediatrician	Desaline Joseph	Evelina London Children's Hospital, UK
4. Public and Patient Involvement Representative	Jo Conduit-Smith	CASTLE Advisory Panel
5. Chief Investigator	Deb K. Pal	King's College London, UK
6. Co-Chief Investigator	Paul Gringras	Evelina London Children's Hospital, UK

Al-Najjar N, *et al. BMJ Open* 2023; 13:e065769. doi: 10.1136/bmjopen-2022-065769

**Independent Data and Safety Monitoring Committee (IDSMC)**<br>**Responsibilities:** Interim monitoring of safety and effectiveness, trial conduct and external data. Recommendation to TSC about trial continuation.<br>**Meeting frequency:** At least annually		
1. Chair	Helen Cross	University College London, UK
2. Paediatrician	Alberto Verroti	University of L'aquila, Italy
3. Medical statistician	• Anthony Johnson (to 31/August/2022)<br>• Appointment pending (20/December/2022)	University College London, UK

Al-Najjar N, *et al. BMJ Open* 2023; 13:e065769. doi: 10.1136/bmjopen-2022-065769

Supplemental Table 3. Psychometrics and clinical relevance/minimal clinically important difference (CR/MCID) for CASTLE Sleep-E outcomes (Table 1). Metrics refer to the single referenced publication. Further validation studies exist, but, due to differences in population, setting, and/or methods, results cannot be merged.

Outcome	Description	Validity	Reliability	CR/MCID
Children's Sleep Habits Questionnaire (CSHQ)[1]	Parent-reported, one-week retrospective sleep screening tool for children (4–10 years)<br><br>35 items (2 duplicated across subscales)<br>3-point Likert scales (rarely, sometimes, usually)<br>Total score (33 items): 33–99, lower is better<br>8 subscales:<br>• Bedtime Resistance (6 items)<br>• Sleep Onset Delay (1 item)<br>• Sleep Duration (3 items)<br>• Sleep Anxiety (4 items)<br>• Night Wakings (3 items)<br>• Parasomnias (7 items)<br>• Sleep-Disordered Breathing (3 items)<br>• Daytime Sleepiness (8 items)<br><br><u>Validation samples</u><br>Parents of 469 school children (community setting) and 154 children diagnosed with sleep disorder (hospital setting); English language; England, UK.<br>Test-retest: 60 parents from control sample	<u>Classification accuracy</u><br>Sleep disorder (yes/no)<br>Receiver Operating Characteristic (ROC) analyses: See MCID<br><br><u>Construct validity</u><br>See MCID<br><br><u>Criterion validity</u><br>Not assessed	<u>Test-retest</u><br>2-week delay<br>Pearson's *r:* 0.62–0.79<br><br><u>Internal consistency</u><br>Cronbach's α<br>Control sample: 0.68<br>Clinical sample: 0.78<br><br><u>Inter-rater reliability</u><br>Not assessed	Cut-off (total score): 41<br>• Sensitivity: 80 %<br>• Specificity: 72 %<br>• Accuracy: 80 %<br><br><u>MCID</u><br>Not assessed
EQ-5D-Y[2 3]	Child- or adolescent reported (4–7 years: EQ-5D-Y proxy; 8–16 years: EQ-5D-Y, ≥16 years: EQ-5D-5L), standardised measure of current ('today')<br>• *health profile* across 5 dimensions,<br>• self-rated *health status*, and<br>• *EQ-5D-Y index value,* using a country-specific weighting	Not yet validated in UK (last updated 07/March/2022)	Not yet validated in UK (last updated 07/March/2022)	<u>CR/MCID</u><br>Applicability to utility scores debated, suggested MCID: difference in index score between baseline health profile and single-level transitions in single domain (e.g. 33333 to 33332).

Al-Najjar N, *et al. BMJ Open* 2023; 13:e065769. doi: 10.1136/bmjopen-2022-065769

Outcome	Description	Validity	Reliability	CR/MCID
	(value set) of a given health profile.<br><br>Two components:<br>1. Descriptive system<br>5 dimensions with 3 response severity options each (tick-box):<br>• Mobility<br>• Self-care<br>• Usual activities<br>• Pain/discomfort<br>• Anxiety/depression<br>2. Visual Analogue Scale<br>Self-rated health on a vertical Visual Analogue Scale (VAS) that ranges from 'The best health you can imagine' (100) to 'The worst health you can imagine' (0).<br><br>Scoring:<br>• Descriptive system: 5-digit *health profile* (best health state: 11111, indicating no problem in each of the 5 dimensions; worst health state: 33333 indicating many problems in each of the 5 dimensions; 243 possible health states are coded)<br>• VAS: 0–100 subjective *health state* (worst to best)<br>• *EQ-5D-5L index value* Single summary number, calculated by subtracting country-specific weighing (value set) of an obtained health profile from 1, where 1 represents the best possible health profile of 11111.<br><br>Value set validation sample (UK)<br>Not yet validated in UK (last updated 07/March/2022)			

Al-Najjar N, *et al. BMJ Open* 2023; 13:e065769. doi: 10.1136/bmjopen-2022-065769

Outcome	Description	Validity	Reliability	CR/MCID
Child Health Utility instrument (CHU-9D)[4]	Child-reported (7–11 years) descriptive system for current ('today') generic health-related quality-of-life<br><br>9 dimensions with 5 response severity options each (circle):<br>• Worried<br>• Sad<br>• Pain<br>• Tired<br>• Annoyed<br>• School-/homework<br>• Sleep<br>• Daily routine<br>• Activities<br><br>Scoring:<br>• Descriptive system: 9-digit *health profile* (best health state: 111111111, indicating no problem in each of the 9 dimensions; worst health state: 555555555 indicating many problems in each of the 5 dimensions; 1953125 possible health states are coded)<br>• *CHU-9D index value* Single summary number indicating the utility value of a given health state, established using Standard Gamble (SG) tasks.<br><br><u>Value set validation sample (England)</u><br>1245 households were randomly sampled from a database of UK names and addresses in Sheffield and Huddersfield (England) were contacted by a research team of the Centre for Research and Evaluation (CRE) at Sheffield Hallam	<u>Predictive accuracy</u><br>Standard ordinary least squares (OLS) regression: 98.41 %<br>No systematic bias, no auto-correlated errors.<br><br><u>Construct validity</u><br>Not assessed<br><br><u>Criterion validity</u><br>Not assessed<br><br><u>Face-validity</u><br>Preference elicitation using Standard Gamble (SG) task, which give the choice of living in a specific health-state until death with certainty (Choice A), or taking a gamble (Choice B) that could result in living in perfect health for the rest of life with a probability *p*, or dying with a probability 1-*p*. The utility value of a given health-state is the point of indifference between options A and B.<br>Utility values are consistent with health profiles but required merging of response options.	<u>Test-retest</u><br>Not assessed<br><br><u>Internal consistency</u><br>Utility values are consistent with health profiles, but required merging of the initial 5 response-levels for all but one of the 9 dimensions as follows:<br>• Worried: 2<br>• Sad: 4<br>• Pain: 4<br>• Tired: 2<br>• Annoyed: 2<br>• School-/homework: 2<br>• Sleep: 4<br>• Daily routine: 5<br>• Activities: 3<br><br><u>Inter-rater reliability</u><br>Not assessed	<u>CR/MCID</u><br>Applicability to utility scores debated, suggested MCID: difference in index score between baseline health profile and single-level transitions in single domain (e.g. 555555555 to 555555554).

Outcome	Description	Validity	Reliability	CR/MCID
	University. 1195 households were approached at the door, of which 661 (55 %) were in, and 300 (25 %) agreed to take part. 282 respondents (all adults) were analysed (94 %). Compared to the general UK population, this adult sample was broadly representative, but more affluent and highly restricted geographically. Modelling did not include key demographic characteristics (e.g. age, gender, education, employment, religion and ethnicity). The sample consisted exclusively of adults but was used to derive a paediatric value set.			
EQ-5D-5L[5]	Adolescent or adult-reported (≥16 years), standardised measure of current ('today'):<br>• *health profile* across 5 dimensions,<br>• subjective *health status*, and<br>• *EQ-5D-5L index value,* using a country-specific weighting (value set) of an obtained health profile.<br><br>Two components:<br>1. <u>Descriptive system</u><br>5 dimensions with 5 response severity options each (tick-box):<br>• Mobility<br>• Self-care<br>• Usual activities<br>• Pain/discomfort<br>• Anxiety/depression<br>2. <u>Visual Analogue Scale</u><br>Self-rated health on a vertical Visual Analogue Scale (VAS) that ranges from 'The best health	<u>Classification accuracy</u><br>Not assessed<br><br><u>Construct validity</u><br>Not assessed<br><br><u>Criterion validity</u><br>Not assessed<br><br><u>Face-validity</u><br>Preference elicitation using time trade-off (TTO) and discrete choice experiments (DCEs).<br>• TTOs: Confirmation of negative relationship between level sum score and average observed value.<br>• DCEs: Confirmation of assumption that health states with lower-level sum	<u>Test-retest</u><br>Not assessed<br><br><u>Internal consistency</u><br>Not assessed<br><br><u>Inter-rater reliability</u><br>Not assessed	<u>CR/MCID</u><br>Applicability to utility scores debated, suggested MCID: difference in index score between baseline health profile and single-level transitions in single domain (e.g. 55555 to 55554).

Al-Najjar N, *et al. BMJ Open* 2023; 13:e065769. doi: 10.1136/bmjopen-2022-065769

Outcome	Description	Validity	Reliability	CR/MCID
	you can imagine' (100) to 'The worst health you can imagine' (0).<br><br>Scoring:<br>• Descriptive system: 5-digit *health profile* (best health state: 11111, indicating no problem in each of the 5 dimensions; worst health state: 55555 indicating many problems in each of the 5 dimensions; 3125 possible health states are coded)<br>• VAS: 0–100 subjective *health state* (worst to best)<br>• *EQ-5D-5L index value* Single summary number, calculated by subtracting country-specific weighing (value set) of an obtained health profile from 1, where 1 represents the best possible health profile of 11111.<br><br><u>Value set validation sample (England)</u><br>2220 households from 66 post-code based primary sampling units in England were contacted by the market research company Ipsos MORI. 2088 participants were invited, of which 996 (47.7 %) completed the valuation questionnaire. Only complete responses were analysed (985 participants, 98.9 %). Compared to the general population of England, the sample included more people aged over 75 years, retired, and with health problems, but fewer younger	scores are more likely to be chosen.		

Outcome	Description	Validity	Reliability	CR/MCID
	participants, and fewer males.			
Knowledge About Sleep in Childhood (KASC, custom-scale devised for CASTLE Sleep-E)	13 items<br>Self-reported Likert-scales assessing parental efficacy in managing child sleep and knowledge about child sleep	Not evaluated	Not evaluated	Not evaluated
Hospital Anxiety and Depression Scale (HADS)[6]	Self-reported, one-week retrospective screening tool for anxiety and depression in people aged 16–65.<br><br>14 items<br>5-point Likert scales (0–3)<br>No total score<br>Subscale score: 0–21, lower is better<br>2 subscales (7 items each):<br>• Depression<br>• Anxiety<br><br>Validation samples<br>2 x 50 patients (16–65 years) with and without psychiatric disorders (hospital setting); English language; England, UK.	Classification accuracy<br>Psychiatric interview,<br>see CR/MCID<br><br>Construct validity<br>See CR/MCID<br><br>Convergent validity<br>Spearman's ρ<br>Interview/self-rating<br>Depression/Depression: 0.79<br>Anxiety/Anxiety: 0.54<br><br>Discriminant validity<br>Spearman's ρ<br>Interview/self-rating<br>Depression/Anxiety ns<br>Anxiety/Depression ns<br><br>Criterion validity<br>See CR/MCID	Test-retest<br>Not assessed<br><br>Internal consistency<br>Spearman's ρ<br>Anxiety: 0.41–0.76<br>Depression: 0.30–0.60<br><br>Inter-rater reliability<br>Not assessed	Cut-offs (subscales)<br>Depression<br>Absent:≤ 7<br>Borderline: 8–10<br>Definite: ≥ 11<br>• False positives: 1 %<br>• False negatives: 1 %<br>Borderline not counted as error<br><br>Anxiety<br>Absent:≤ 7<br>Doubtful: 8–10<br>Definite: ≥ 11<br>• False positives: 5 %<br>• False negatives: 1 %<br>Borderline not counted as error<br><br>MCID<br>Not assessed

Al-Najjar N, *et al. BMJ Open* 2023; 13:e065769. doi: 10.1136/bmjopen-2022-065769

**Outcome**	**Description**	**Validity**	**Reliability**	**CR/MCID**
Insomnia Severity Index (ISI)[7], patient version	Self-reported, one-month retrospective screening tool for insomnia in adults (≥18 years)<br>7 items<br>5-point Likert scales (0–4, no problem to severe problem)<br>Total score: 0–28, lower is better<br>• 0–7: Absence of insomnia<br>• 8–14: Subthreshold insomnia<br>• 15–21: Moderate insomnia<br>• 22–28: Severe insomnia<br>Dimensions:<br>• Severity of sleep onset Sleep maintenance<br>• Early morning awakening problems<br>• Sleep dissatisfaction<br>• Interference of sleep difficulties with daytime functioning<br>• Noticeability of sleep problems by others<br>• Distress caused by the sleep difficulties<br><br>Validation samples<br>959 adults with and without insomnia (community setting), 183 adults with insomnia and 62 controls (clinical setting); English language; Québec, Canada.	Classification accuracy<br>Insomnia (yes/no)<br>ROC analyses, see MCID<br><br>Construct validity<br>See CR/MCID<br>Pearson's *r*<br>• Daily sleep diary: 0.54–0.59<br>• Activity level, Anxiety (state, trait), Depression, Fatigue (general, physical, mental), Motivation: 0.20–0.48<br><br>Criterion validity<br>Pearson's *r*<br>Polysomnography<br>• Sleep onset latency: ns<br>• Wake after sleep onset: ns<br>• Number of awakenings: ns<br>• Early morning awakening: ns<br>• Total wake time: ns<br>• Sleep efficiency: -0.16	Test-retest<br>Not assessed<br><br>Internal consistency<br>Cronbach's α, Control sample: 0.71<br>Clinical sample: 0.73<br><br>Inter-rater reliability<br>Not assessed	Control sample (self-diagnosis)<br>Cut-off (total score): 10<br>• Sensitivity: 86 %<br>• Specificity: 88 %<br>• Accuracy: 87 %<br><br>Clinical sample<br>Cut-off (total score): 11<br>• Sensitivity: 97 %<br>• Specificity: 100%<br>Accuracy: 98 %<br><br>MCID<br>Change required for improvement<br>Blinded assessor, *M*, [CI_{95}]:<br>• Slight: 4.65 [2.61–6.69]<br>• Moderate: 8.36 [7.20–9.53]<br>• Marked: 9.89 [8.74–11.04]<br>ROC analyses:<br>• Slight: not reported<br>• Moderate: ≥7<br>○ Sensitivity: 60 %<br>○ Specificity: 70 %<br>○ Accuracy: not reported<br>• Marked: ≥8<br>○ Sensitivity: 64 %<br>○ Specificity: 80 %<br>○ Accuracy: not reported

Al-Najjar N, *et al. BMJ Open* 2023; 13:e065769. doi: 10.1136/bmjopen-2022-065769

Outcome	Description	Validity	Reliability	CR/MCID
SleepSuite[8] (iPad App): Bubble task<br><br>• Executive function (accuracy and response times [RT])	SleepSuite bubble tasks (iPad games) are adapted from a validated Balloon Task[9]: The goal is to burst upward drifting balloons with children's faces under multiple target conditions (e.g. happy faces only) and at increasing presentation conditions (speed, load: number of faces shown simultaneously).<br><br><u>Validation sample[9]</u><br>134 healthy children (7–12 years, 58 boys, 23 with clinical behavioural problems, 40% first-born) from middle- and upper-class families of which 25% included at least one parent who immigrated more than 10 years ago. Children lived with their parents in small households (on average 4.53 members). Parents were largely employed full-time (fathers: 90.71%, mothers: 49.31%) and well educated (on average for 16 years). Community setting (school, number unspecified); paid participation ($15 school supply voucher); language: Hebrew, Israel.	<u>Classification accuracy</u><br>Not assessed<br><br><u>Construct validity</u><br>Not assessed<br><br><u>Criterion validity</u><br>Child Behavior Checklist (CBCL): total score, sub-scales (8), recode to externalising and internalising behaviours.<br><br>Pearson's *r* (age and sex partialled out), across conditions<br><br>**Completed levels/RT**<br>• Total score: -0.24/ns<br>• Delinquency: ns/0.18<br>• Aggression: -0.20/0.23<br>• Attention problems: -0.18/ns<br>• Social withdrawal: -0.24/ns<br>• Somatic complaints: ns/0.18<br>• Thought disorders: ns/ns<br>• Anxiety-Depression: -.28/ns<br>• Social problems: -0.20/ns<br>• Externalising behaviours: -0.18/0.23<br>• Internalising behaviours: -0.25/ns	<u>Test-retest</u><br>Delay unspecified (likely none [immediate retest])<br><br>Pearson's *r*<br>• Hits: 0.60<br>• Misses: 0.37<br>• Completed levels: 0.39<br>• RT: 0.78<br><br><u>Internal consistency</u><br>Not assessed<br><br><u>Inter-rater reliability</u><br>Not assessed	Not assessed

Al-Najjar N, *et al. BMJ Open* 2023; 13:e065769. doi: 10.1136/bmjopen-2022-065769

Outcome	Description	Validity	Reliability	CR/MCID
Health-Related **Q**uality **O**f **L**ife Measure for **Ch**ildren with **E**pilepsy (CHEQOL)[10]	Quality of life assessment tool for children or parents with epilepsy (no specified time-period); child reported if ≥8 years, parent proxy-report if child 5 to <8 years<br>25 items<br>4-point Likert scales (0–4, opposites: true/sort of true)<br>Total score: 25–100, higher is better<br>5 subscales (5 items each):<br>• Interpersonal/social consequences<br>• Future worries<br>• Present worries<br>• Intrapersonal/emotional<br>• Epilepsy secrecy<br><br>Validation samples<br>381 children (6–15 years) with epilepsy and their parents (clinical setting); English language; Ontario, Canada. Test-retest: Additional 89, then 31 children; additional 48 parents.<br>Metrics refer to self-report for children 8–15 years and parent proxy report for children 5 to <8 years and were assessed for sub-scales, not total score.	Classification accuracy<br>Not assessed<br><br>Construct validity (child)<br>Pearson's *r*<br>• Health care utilisation: 0.13–0.31<br>• Drug Adverse Events: 0.18–0.25<br>• Number of friends: 0.18<br>• Nº of extracurricular activities: 0.13<br>One-way ANOVA ($p \leq .05$)<br>• Seizure severity: All 5 subscales<br>• Anti-epileptic drug use: 4 subscales<br>*t–tests* ($p \leq .05$)<br>• Help at school: All 5 subscales<br>Results for parent-proxy similar<br><br>Criterion validity<br>Not assessed	Test-retest<br>10– 14 days delay<br>Intraclass correlation coefficient<br>Child: 0.59–0.69<br>Parent: 0.60–0.81<br><br>*Internal consistency*<br>Cronbach's *α,* subscales<br>*Child: 0.63*–0.84<br>*Parent: 0.64*–0.86<br><br>Inter-rater reliability<br>Pearson's *r*<br>• Child/mother: 0.24–0.56<br>• Child/father: 0.18–0.54<br>• Mother/father: 0.40–0.71	Not assessed

Al-Najjar N, *et al. BMJ Open* 2023; 13:e065769. doi: 10.1136/bmjopen-2022-065769

Outcome	Description	Validity	Reliability	CR/MCID
World Health Organisation – Five Well-Being Index (WHO-5)[11]	Self-reported, two-week retrospective tool to assess subjective psychological well-being in people aged 9 years and older.<br><br>5 items<br>6-point Likert scales (0–5, 'at no time' to 'all the time')<br>Raw score: 0–25<br>Total score multiplied by 4 to give final score: 0–100, higher is better<br><br>Validation samples<br>446 children analysed (9–12 years, 16 [3.6 %] with depressive disorder), 6 additional participants dropped due to incomplete data.<br>Hospital setting: 3 paediatric hospitals and 3 paediatric surgery hospitals (in- and out-patients for non-psychiatric reasons), Munich, Germany.<br>German language.	Classification accuracy<br>Depressive disorder (yes/no)<br>Receiver Operating Characteristic (ROC) analyses: See CR/MCID<br><br>Construct validity<br>See CR/MCID<br><br>Criterion validity<br>Diagnostic and Statistical Manual of Mental Disorders (DSM-IV) criteria for depressive disorder (major or minor depression only, dysthymia dropped due to mismatch in time-period of concept definitions), see CR/MCID.	Test-retest<br>Not assessed<br><br>Internal consistency<br>Not assessed<br><br>Inter-rater reliability<br>Cohen's k = .90	Cut-off (total score): 10<br>• Sensitivity: 75 %<br>• Specificity: 92 %<br>• Accuracy: 88 %<br><br>MCID<br>Not assessed

Outcome	Description	Validity	Reliability	CR/MCID
Strengths and Difficulties Questionnaire (SDQ)[12]	Parent-, teacher-, or child-reported, retrospective screening tool of child psychopathology (2–18 years). Retrospective period: 6 months or current school year<br><br>25 items<br>3-point Likert scales (0–2, not/somewhat/certainly true)<br>Total score: 0–40, lower is better<br>5 subscales (5 items each):<br>• hyperactivity/inattention,<br>• emotional problems<br>• conduct problems<br>• peer problems<br>• prosocial behaviours (omitted from total score)<br><br>Validation samples<br>541 children (5–12 years) with and without psychiatric disorders (school setting); multiple languages; Italy, Germany, the Netherlands, Lithuania, Bulgaria, Romania, and Turkey. Metrics refer to parent-report, total score, and data aggregated across countries and psychiatric disorders.	Classification accuracy<br>Psychiatric disorder (yes/no)<br>Receiver Operating Characteristic (ROC) analyses: See CR/MCID<br>Original total score cut-offs:<br>• Normal: 0–13<br>• Borderline: 14–16<br>• Abnormal: 17–40<br>transformed to binary:<br>• No: 0–16<br>• Yes: 17–40<br><br>Construct validity<br>See CR/MCID<br><br>Criterion validity<br>Diagnostic and Statistical Manual of Mental Disorders (DSM-IV), see CR/MCID.	Test-retest<br>Not assessed<br><br>Internal consistency<br>Cronbach's α: 0.84<br><br>Inter-rater reliability<br>Not assessed.	Cut-off (total score): 17<br>• Sensitivity: 88 %<br>• Specificity: 59 %<br>• Accuracy: 74 %<br><br>MCID<br>Not assessed

Al-Najjar N, *et al. BMJ Open* 2023; 13:e065769. doi: 10.1136/bmjopen-2022-065769

Outcome	Description	Validity	Reliability	CR/MCID
Parenting Self Agency Measure (PSAM)[13]	Self-reported tool assessing overall confidence to successfully parent (including managing the child's behaviour and resolving problems with the child). The time-period for parental self-assessment is unspecified.<br><br>5 items<br>7-point Likert scales (1–7, rarely to always)<br>Total score: 5–35, higher is better<br><br>Validation sample<br>90 English-speaking mothers (all European-American, median age 36–40 years, median annual income >$40,000, median education bachelor's degree, 82% married or co-habiting) of 3–12-year-olds (community setting); 2 day-care centres and classes at a large university, 2 churches. English language, southwestern USA.	Classification accuracy<br>Not assessed<br><br>Construct validity<br>Convergent validity<br>Pearson's *r*<br>Active coping: 0.31<br>Parenting acceptance: 0.55<br>Positive re-interpretation: ns<br><br>Discriminant validity<br>Pearson's *r*<br>Inconsistent parental disciplining: -0.34<br>Acceptance coping: ns<br><br>Criterion validity<br>Not assessed	Test-retest<br>Not assessed<br><br>Internal consistency<br>Cronbach's α: 0.70<br>Comparative Fit Index: 0.94<br><br>Inter-rater reliability<br>Not assessed	Not assessed

Al-Najjar N, *et al. BMJ Open* 2023; 13:e065769. doi: 10.1136/bmjopen-2022-065769

Outcome	Description	Validity	Reliability	CR/MCID
Actigraphy: Micro Motionlogger® Watch, Watchware Software V 1.99.17.4, Action-W software, V 2.7.3285 (Ambulatory Monitoring, Inc., NY: USA) combined with sleep diaries (Child and Parent)<br><br>• Total sleep time (minutes)<br>• Sleep latency (minutes)<br>• Sleep efficiency (% asleep of sleep period)<br><br>All 2-week averages	The Micro-Motionlogger® Watch directly measures 3-D acceleration (in CASTLE Sleep-E and the referenced validation study of the non-dominant wrist). Raw data (zero-crossing mode) is initially recorded as periods of activity and inactivity (1 min epochs), and then recoded into periods of wakefulness and sleep using a combination of proprietary algorithms and manual processing (e.g. sleep periods are visually inspected and manually corrected with the aid of participant sleep diaries). Sleep- and wake parameters are then calculated automatically using validated public algorithms.<br><br><u>Validation sample[9]</u><br>27 children (3–17 years) with medically refractory epilepsy, of which 12 had parent-indicated sleep problems (44%). Hospital setting (in-patient epilepsy monitoring unit in tertiary paediatric hospital), English language, Toronto, Canada.	<u>Classification accuracy</u><br>Not assessed<br><br><u>Construct validity</u><br>Not assessed<br><br><u>Criterion validity</u><br>Agreement of actigraphy with continuous video-electroencephalography (24 hours), scored by neurologist and neurophysiologist.<br><br>Bland-Altman plots in combination with *t*-tests for significant bias:<br>• Total sleep time (minutes): Bias = 8.3 (SD = 31), n.s.<br>• Wake duration: Bias = -4.8 (SD = 31.1), n.s.<br><br>Pearson's *r:*<br>• Total sleep time (minutes): 0.96<br>• Wake duration: 0.93	<u>Test-retest</u><br>Not assessed<br><br><u>Internal consistency</u><br>Not assessed<br><br><u>Inter-rater reliability</u><br>Not assessed	Not assessed

Al-Najjar N, *et al. BMJ Open* 2023; 13:e065769. doi: 10.1136/bmjopen-2022-065769

Table 4. Estimated overall time requirement for CASTLE Sleep-E (participant perspective). Time estimates for questionnaires/instruments are based on published estimates where available, and otherwise on an estimate (indicated by *) of 30 seconds per item derived from the Children's Sleep Habits Questionnaire (35 items, 10 minutes published completion time), plus an arbitrary estimate of 2 minutes to read instructions and consider responses. The total time requirement for participation in CASTLE Sleep-E varies from minimally 2 hours per month over a 6-month period in the Standard Care arm omitting optional qualitative interviews to maximally 3 hours per month over a 6-month period in the intervention arm including optional qualitative interviews.

Trial component	Time (mins)	Frequency	Overall time (mins)
Study visits (4)<br>Remote or in-person, combinable with standard care visits<br>• Consent and baseline data<br>• Randomisation<br>• Follow-up at 3 months<br>• Follow-up at 6 months	 <br>• 60 minutes<br>• 30 minutes<br>• 30 minutes<br>• 30 minutes	 <br>• 1<br>• 1<br>• 1<br>• 1	150 minutes
Questionnaires/instruments in order of the participant timeline shown in Table 4<br>• Children's Sleep Habits Questionnaire[1], 35 items<br>• World Health Organisation – Five Well-Being Index[11], 5 items<br>• Health-Related Quality Of Life Measure for Children with Epilepsy[10], 25 items<br>• Strengths and Difficulties Questionnaire[12], 25 items<br>• Child Health Utility Index 9D (CHU-9D)/CHU-9D proxy[4], 9 items<br>• EQ-5D-Y/EQ-5D-Y proxy[2], 15 items<br>• EQ-5D-5L[5], 25 items (note: Published time estimate same as for EQ-5D-Y [15 items])<br>• Parenting Self Agency Measure[13], 5 items<br>• Insomnia Severity Index[7], patient version, 7 items<br>• Hospital Anxiety and Depression Scale[6], 14 items<br>• Resource Use questionnaire (custom instrument), 11 items<br>• Knowledge About Sleep in Childhood (custom scale), 13 items	 • 10 minutes<br>• 5 minutes<br>• 12.5 + 2 minutes*<br>• 12.5 + 2 minutes*<br>• 4.5 + 2 minutes*<br>• 5 minutes<br>• 5 minutes<br>• 2.5 + 2 minutes*<br>• 3.5 + 2 minutes*<br>• 5 minutes<br>• 5.5 + 2 minutes*<br>• 6.5 + 2 minutes*	 • 3<br>• 2<br>• 2<br>• 3<br>• 3<br>• 3<br>• 3<br>• 3<br>• 3<br>• 3<br>• 3<br>• 2	246.5 minutes<br>• 30 minutes<br>• 10 minutes<br>• 29 minutes<br>• 43.5 minutes<br>• 19.5 minutes<br>• 15 minutes<br>• 15 minutes<br>• 13.5 minutes<br>• 16.5 minutes<br>• 15 minutes<br>• 22.5 minutes<br>• 21 minutes
SleepSuite[8] (iPad App)<br>• Morning of single day<br>• Evening of single day	40 minutes<br>• 20 minutes<br>• 20 minutes	2	80 minutes

Al-Najjar N, *et al. BMJ Open* 2023; 13:e065769. doi: 10.1136/bmjopen-2022-065769

Trial component	Time (mins)	Frequency	Overall time (mins)
Actigraphy<br>• Delivery arrangements to participants' home or collection point (incl. SleepSuite iPad)<br>○ Baseline<br>○ Follow-up at 3 months<br>• Return arrangements to participants' home or collection point (incl. SleepSuite iPad)<br>○ Baseline<br>○ Follow-up at 3 months<br>• Use: Removal and re-fitting of device once daily (2 x 0.25 minute) when showering, bathing, or swimming; otherwise, the device is worn like a wristwatch without requiring participant interventions.<br>○ Baseline: 14 days<br>○ Follow-up at 3 months: 14 days	• 15 minutes<br>• 15 minutes<br>• 15 minutes<br>• 15 minutes<br>• 7 minutes<br>• 7 minutes	• 1<br>• 1<br>• 1<br>• 1<br>• 1<br>• 1	74 minutes
Sleep diary<br>Once daily completion of parent- and child diary (2 x 2.5 minutes)<br>• Baseline: 14 days<br>• Follow-up at 3 months: 14 days	• 70 minutes<br>• 70 minutes	• 1<br>• 1	140 minutes
COSI (*intervention arm only*)<br>• 3 mandatory modules (core information about sleep relevant to all families)<br>• 3 recommended modules (e.g. sleep hygiene)<br>• 5 tailored modules (addressing specific sleep issues indicated by a given parent)<br>• List of additional resources, *optional*, 10 webpages, not included in time estimate<br>• Evaluation questionnaire, 3 sections, 47 items overall<br>A parent assigned to COSI (i.e. the intervention arm) would be expected to look at minimally 7 and maximally 11 modules. All modules are self-paced (i.e. do not have a fixed duration). To read and engage with a single module could take anywhere between 5–20 minutes depending on how quickly one reads, whether one watches the videos, does the quizzes, etc. Consequently, the estimated time requirement for initial material completion not including breaks or re-visits is 35–220 minutes for modules alone.<br>To be conservative, maximal estimates are used in calculations.	• 60 minutes<br>• 60 minutes<br>• 100 minutes<br>• 0 minutes<br>• 23.5 + 2 minutes*	• 1<br>• 1<br>• 1<br>• 1<br>• 1	245.5 minutes

**Trial component**	**Time (mins)**	**Frequency**	**Overall time (mins)**
Qualitative interviews (*optional*)<br>Two time-points (Follow-up at 3 months + 3 weeks, at 6 months + 3 weeks)<br>• Interview date and time arrangement<br>• Interview preparation using supplied interview guide<br>• Actual interview<br>• De-brief<br>For the qualitative interviews with parents, we typically expect that the total time burden for each of the two interviews would range from 30–70 minutes. However, we will tailor the core interview to fit with the time the parent has available, so some interviews may be a little longer or shorter.<br>To be conservative, maximal estimates are used in calculations.	 <br>• 10 minutes<br>• 10 minutes<br>• 40 minutes<br>• 10 minutes	 <br>• 2<br>• 2<br>• 2<br>• 2	140 minutes<br><br>• 20 minutes<br>• 20 minutes<br>• 80 minutes<br>• 20 minutes
**Total time for participation over a 6-months period**<br>• **Standard Care arm (SC), not participating in optional qualitative interviews**<br>• **Standard Care arm (SC), participating in optional qualitative interviews**<br>• **Intervention arm (SC + COSI), not participating in optional qualitative interviews**<br>• **Intervention arm (SC + COSI), participating in optional qualitative interviews**			 • **690.5 minutes**<br>• **830.50 minutes**<br>• **936 minutes**<br>• **1076 minutes**

Supplemental Table 5. Categories used to define the causality and severity of Adverse Events in CASTLE Sleep-E

**Category**	**Definition**
Causality	
Almost Certainly	There is clear evidence to suggest a causal relationship, and other possible contributing factors can be ruled out.
Probably	There is evidence to suggest a causal relationship, and the influence of other factors is unlikely.
Possibly	There is some evidence to suggest a causal relationship (e.g. the event occurred within a reasonable time after administration of the study procedure). However, the influence of other factors may have contributed to the event (e.g. the participant's clinical condition, other concomitant events).
Unlikely	There is little evidence to suggest there is a causal relationship (e.g. the event did not occur within a reasonable time after administration of the study procedure). There is another reasonable explanation for the event (e.g. the participant's clinical condition).
Not related	There is no evidence of any causal relationship.
Severity	
Mild	The Adverse Event does not interfere with the participant's daily routine and does not require further procedure; it causes slight discomfort.
Moderate	The Adverse Event interferes with some aspects of the participant's routine, or requires further procedure, but is not damaging to health; it causes moderate discomfort.
Severe	The Adverse Event results in alteration, discomfort or disability which is clearly damaging to health.

Al-Najjar N, *et al. BMJ Open* 2023; 13:e065769. doi: 10.1136/bmjopen-2022-065769

References

1. Owens JA, Spirito A, McGuinn M. The Children's Sleep Habits Questionnaire (CSHQ): psychometric properties of a survey instrument for school-aged children. Sleep 2000;**23**(8):1043-51.
2. Wille N, Badia X, Bonsel G, et al. Development of the EQ-5D-Y: a child-friendly version of the EQ-5D. Qual Life Res 2010;**19**(6):875-86 doi: 10.1007/s11136-010-9648-y [published Online First: 20100420].
3. EuroQol Research Foundation. EQ-5D-Y User Guide, 2020.
4. Stevens K. Valuation of the Child Health Utility 9D Index. Pharmacoeconomics 2012;**30**(8):729-47 doi: 10.2165/11599120-000000000-00000.
5. Devlin NJ, Shah KK, Feng Y, et al. Valuing health-related quality of life: An EQ-5D-5L value set for England. Health Econ 2018;**27**(1):7-22 doi: 10.1002/hec.3564 [published Online First: 20170822].
6. Zigmond AS, Snaith RP. The hospital anxiety and depression scale. Acta Psychiatr Scand 1983;**67**(6):361-70 doi: 10.1111/j.1600-0447.1983.tb09716.x.
7. Morin CM, Belleville G, Bélanger L, et al. The Insomnia Severity Index: psychometric indicators to detect insomnia cases and evaluate treatment response. Sleep 2011;**34**(5):601-08 doi: 10.1093/sleep/34.5.601.
8. Colonna A, Smith AB, Smith S, et al. The Effects of Sleep on Emotional Target Detection Performance: A Novel iPad-Based Pediatric Game. Frontiers in psychology 2018;**9**:241-41 doi: 10.3389/fpsyg.2018.00241.
9. Sadaka Y, Sadeh A, Bradbury L, et al. Validation of actigraphy with continuous video-electroencephalography in children with epilepsy. Sleep Med 2014;**15**(9):1075-81 doi: 10.1016/j.sleep.2014.04.021 [published Online First: 20140602].
10. Ronen GM, Streiner DL, Rosenbaum P, et al. Health-related quality of life in children with epilepsy: development and validation of self-report and parent proxy measures. Epilepsia 2003;**44**(4):598-612 doi: 10.1046/j.1528-1157.2003.46302.x.
11. Allgaier A-K, Pietsch K, Frühe B, et al. Depression in pediatric care: is the WHO-Five Well-Being Index a valid screening instrument for children and adolescents? General Hospital Psychiatry 2012;**34**(3):234-41 doi: https://doi.org/10.1016/j.genhosppsych.2012.01.007.
12. Goodman R. Psychometric Properties of the Strengths and Difficulties Questionnaire. Journal of the American Academy of Child & Adolescent Psychiatry 2001;**40**(11):1337-45 doi: 10.1097/00004583-200111000-00015.
13. Dumka LE, Stoerzinger HD, Jackson KM, et al. Examination of the Cross-Cultural and Cross-Language Equivalence of the Parenting Self-Agency Measure. Family Relations 1996;**45**(2):216-22 doi: 10.2307/585293.
