# Supplementary material for: Changing Agendas on Sleep, Treatment and Learning in Epilepsy (CASTLE) Sleep-E: a protocol for a randomised controlled trial comparing an online behavioural sleep intervention with standard care in children with Rolandic epilepsy
Source: BMJ Open. 2023 Mar 10;13(3):e065769. doi: 10.1136/bmjopen-2022-065769 (PMC10008377; doi:10.1136/bmjopen-2022-065769)
Supplement: Author's manuscript [file bmjopen-2022-065769.draft_revisions.pdf]

BMJ Open is committed to open peer review. As part of this commitment we make the peer review history of every article we publish publicly available.

When an article is published we post the peer reviewers' comments and the authors' responses online. We also post the versions of the paper that were used during peer review. These are the versions that the peer review comments apply to.

The versions of the paper that follow are the versions that were submitted during the peer review process. They are not the versions of record or the final published versions. They should not be cited or distributed as the published version of this manuscript.

BMJ Open is an open access journal and the full, final, typeset and author-corrected version of record of the manuscript is available on our site with no access controls, subscription charges or pay-per-view fees (http://bmjopen.bmj.com).

If you have any questions on BMJ Open's open peer review process please email info.bmjopen@bmj.com

BMJ Open

Changing Agendas on Sleep, Treatment and Learning in Epilepsy (CASTLE) Sleep-E: A protocol for a randomised controlled trial comparing an online behavioural sleep intervention with standard care in children with Rolandic epilepsy

Journal:	*BMJ Open*
Manuscript ID	bmjopen-2022-065769
Article Type:	Protocol
Date Submitted by the Author:	10-Jul-2022
Complete List of Authors:	Group, CASTLE; King's College London<br>Advisory Panel, CASTLE; Edge Hill University<br>Al-Najjar, Nadia; University of Liverpool<br>Bray, Lucy; Edge Hill University<br>Carter, Bernie; Edge Hill University<br>Collingwood, Amber; King's College London<br>Cook, Georgia; Oxford Brookes University<br>Crudgington, Holly; King's College London<br>Dietz, Kristina; King's College London<br>Gringras, Paul; Evelina London Children's Healthcare<br>Hardy, Will A. S.; Bangor University<br>Hiscock, Harriet; Murdoch Children's Research Institute<br>Hughes, Dyfrig; Bangor University<br>Morris, Christopher; University of Exeter<br>Morris, Hannah; University of Exeter<br>Pal, Deb; King's College London<br>Rogers, Morwenna; University of Exeter<br>Rouncefield-Swales, Alison; Edge Hill University<br>Saron, Holly; Edge Hill University<br>Spowart, Catherine; University of Liverpool<br>Stibbs-Eaton, Lucy; University of Liverpool<br>Tudur-Smith, Catrin; University of Liverpool<br>Watson, Victoria; University of Liverpool<br>Wiggs, Luci; Oxford Brookes University<br>Williamson, Paula; University of Liverpool<br>Wood, Elfiona; Bangor University
Keywords:	Epilepsy < NEUROLOGY, Paediatric neurology < NEUROLOGY, SLEEP MEDICINE, Clinical trials < THERAPEUTICS

SCHOLARONE™
Manuscripts

TITLE

Changing Agendas on Sleep, Treatment and Learning in Epilepsy (CASTLE) Sleep-E: A protocol for a randomised controlled trial comparing an online behavioural sleep intervention with standard care in children with Rolandic epilepsy

Authorship including affiliations and ORCID IDs (alphabetic surname order)

1. CASTLE Group, King's College London, UK CASTLE_group@twistedsquare.com
2. CASTLE Advisory Panel (CAP), CASTLE Research Programme, UK, martinra@edgehill.ac.uk
3. Nadia Al-Najjar (NA-N), University of Liverpool, UK, Nadia.Al-Najjar@liverpool.ac.uk
4. Lucy Bray (LB), Edge Hill University, UK, brayl@edgehill.ac.uk http://orcid.org/0000-0001-8414-3233
5. Bernie Carter (BC), Edge Hill University, UK, bernie.carter@edgehill.ac.uk http://orcid.org/0000-0001-5226-9878
6. Amber Collingwood (AC), King's College London, UK, amber.collingwood@kcl.ac.uk
7. Georgia Cook (GC), Oxford Brookes University, UK, gcook@brookes.ac.uk http://orcid.org/0000-0002-1651-866X
8. Holly Crudgington (HC), King's College London, UK, holly.1.crudgington@kcl.ac.uk http://orcid.org/0000-0003-1048-4953
9. Kristina C. Dietz (KCD), King's College London, UK, kristina.dietz@kcl.ac.uk http://orcid.org/0000-0002-3074-6319
10. Paul Gringras (PG), Evelina London Children's Hospital, UK, paul.gringras@gstt.nhs.uk http://orcid.org/0000-0002-0495-3517
11. Will A. S. Hardy (WASH), Bangor University, UK, w.hardy@bangor.ac.uk http://orcid.org/0000-0001-5227-567X
12. Harriet Hiscock (HH), Murdoch Children's Research Institute, Australia, harriet.hiscock@mcri.edu.au http://orcid.org/0000-0003-3017-2770
13. Dyfrig Hughes (DH) Bangor University, UK, d.a.hughes@bangor.ac.uk http://orcid.org/0000-0001-8247-7459
14. Christopher Morris (CM), University of Exeter, UK, Christopher.Morris@exeter.ac.uk http://orcid.org/0000-0002-9916-507X
15. Hannah Morris (HM), University of Exeter, UK, Hannah.Morris@exeter.ac.uk
16. Deb K. Pal (DKP), King's College London, UK, deb.pal@kcl.ac.uk http://orcid.org/0000-0003-2655-0564
17. Morwenna Rogers (MR), University of Exeter, UK, Morwenna.Rogers@exeter.ac.uk http://orcid.org/0000-0002-6039-238X
18. Alison Rouncefield-Swales (AR-S), University of Central Lancashire, UK, rouncefia@edgehill.ac.uk https://orcid.org/0000-0001-9947-7375
19. Holly Saron (HS) Edge Hill University, UK, saronh@edgehill.ac.uk http://orcid.org/0000-0001-7563-3409
20. Catherine Spowart (CS), University of Liverpool, UK Catherine.Spowart@liverpool.ac.uk http://orcid.org/0000-0001-8641-2871
21. Lucy Stibbs-Eaton, University of Liverpool, UK, l.stibbs-eaton@liverpool.ac.uk http://orcid.org/0000-0002-3672-4006
22. Catrin Tudor Smith (CT-S), University of Liverpool, UK, Cat1@liverpool.ac.uk http://orcid.org/0000-0003-3051-1445

For peer review only

23. Victoria Watson (VW), University of Liverpool, UK, victoria.watson@liverpool.ac.uk
24. Luci Wiggs (LW), Oxford Brookes University, UK, lwiggs@brookes.ac.uk
http://orcid.org/0000-0002-5697-6550
25. Paula R Williamson (PRW), University of Liverpool, UK, P.R.Williamson@liverpool.ac.uk
26. Eifiona Wood (EW), Bangor University, UK, e.wood@bangor.ac.uk
https://orcid.org/0000-0002-2785-7325

Corresponding author: Dr Kristina C Dietz, Maurice Wohl Clinical Neuroscience Institute, King's College London, 5 Cutcombe Road, London, SE5 9RX, kristina.dietz@kcl.ac.uk, +44 (0) 207 848 025 9

Keywords (Medical Subject Headings 2022): Epilepsy, Rolandic; Sleep; Child; Randomized Controlled Trials as Topic; Evidence-Based Medicine

Word count: 3 964

For peer review only

ABSTRACT

Introduction Sleep and epilepsy have an established bi-directional relationship yet only one randomised controlled clinical trial has assessed the effectiveness of behavioural sleep interventions for children with epilepsy. The intervention was successful, but delivered via face-to-face educational sessions with parents, which are costly and non-scalable to population level. The Changing Agendas on Sleep, Treatment and Learning in Epilepsy (CASTLE) Sleep-E trial addresses this problem by comparing clinical- and cost-effectiveness in children with Rolandic epilepsy between standard care and standard care augmented with a novel, tailored parent-led CASTLE Online Sleep Intervention (COSI) that incorporates evidence-based behavioural components.

Methods and analyses CASTLE Sleep-E is a UK-based, multi-centre, open label, randomised, parallel-group, pragmatic superiority trial. A total of 110 children with Rolandic epilepsy will be recruited in out-patient clinics and allocated 1:1 to standard care (SC) or standard care augmented with COSI (SC + COSI). Primary clinical outcome is parent-reported sleep problem score (Children's Sleep Habits Questionnaire). Primary health economic outcome is the Incremental Cost Effectiveness Ratio (National Health Service and Personal Social Services perspective, Child Health Utility 9D instrument). Parents and children (≥ 7 years) can opt into qualitative interviews and activities to share their experiences and perceptions. Jointly, the qualitative trial component and the COSI system (e-analytics and evaluation module) will provide information for a process evaluation (context, implementation, and mechanisms of impact).

Ethics and dissemination The CASTLE Sleep-E protocol was approved by the Health Research Authority East Midlands – Nottingham 1 Research Ethics Committee, reference: 21/EM/0205. Trial results will be disseminated to scientific audiences, families, professional groups, managers, commissioners, and policy makers. Pseudo-anonymised Individual Patient Data will be made available after dissemination on reasonable request.

Registration details ISRCTN registry (Trial ID: ISRCTN13202325, prospective registration 09/Sep/2021). See Supplemental Table 1 for the World Health Organisation Trial Registration Data Set (Version 1.3.1).

For peer review only

Strengths and limitations of this study

- First randomised controlled trial to evaluate the clinical- and cost-effectiveness of a novel, tailored, parent-led CASTLE Online Sleep Intervention (COSI) that incorporates evidence-based behavioural components for children with Rolandic epilepsy
- Extensive Patient and Public Involvement via dedicated CASTLE Advisory Panel
- Embedded health economic evaluation
- Embedded process evaluation
- **Limitation**: Heavily reliant on participant self-report to assess intervention implementation, ameliorated by COSI e-analytics and actigraphy data

For peer review only

INTRODUCTION

Epilepsy is one of the most common long-term neurological conditions worldwide whose prevalence peaks during childhood (5–9 years) and again late in life (over 80 years).[1] Epilepsy in children (5 to <13 years) accounts for the annual loss of over 2.6 million disability-adjusted life years, equivalent to 1.8 % of the global burden of disease from all causes among children and adolescents.[2] Rolandic Epilepsy (RE) is the most common childhood epilepsy.[3]

In the UK, RE has a stable crude incidence rate of 5 in 100 000 children (<16 years) or 542 new cases annually.[4] Concurrent neuro-developmental disorders are very common (35 %).[5] Seizures are often triggered by sleep fragmentation.[6] Many parents co-sleep or monitor children with nocturnal seizures, and children themselves experience a fear of death during and after a seizure.[7] Problems related to sleep emerge as one of the top concerns for both children and parents,[8] but are often unaddressed.[9 10]

A recent systematic review and meta-analysis of clinical trials shows that parent-based behavioural sleep interventions are effective for typically-developing children and those affected by neurological and neuro-developmental disorders.[10] The review concluded that randomised controlled clinical trials assessing functional outcomes (e.g. cognition, emotion, behaviour) and targeting specific populations (e.g. epilepsy) are missing (but see two recent trials).[11 12] Harms capture for cognitive-behavioural and behavioural sleep interventions has been sparse (only 32.3 % of trials address Adverse Events) and predominantly inadequate (92.9 % of trials do not meet adequate reporting criteria).[13] Observed harms of behavioural sleep interventions in adults have been mild (e.g. transient fatigue/exhaustion from sleep restriction in insomnia in 25–33 % of participants).[14] The only published paediatric and adult epilepsy trials did not address harms.[11 12] Based on the existing evidence, the benefits of behavioural sleep interventions in children with epilepsy outweigh potential harms, especially because sleep problems not only affect seizure control, but overall child well-being, learning and memory, and parental quality of life.[9 10] There remains, however, uncertainty whether sleep interventions, which can be resource intensive, are cost-effective in public health systems.

This protocol describes the design for the Changing Agendas on Sleep, Treatment and Learning in Epilepsy (CASTLE) Sleep-E trial, which evaluates the clinical- and cost-effectiveness of a novel, tailored, parent-led CASTLE Online Sleep Intervention (COSI) that incorporates evidence-based behavioural components for children with epilepsy. COSI and CASTLE Sleep-E outcome-selection were co-produced by affected children, young people, and their parents, sleep- and epilepsy experts.[8 15-17] The CASTLE Sleep-E protocol follows Standard Protocol Items: Recommendations for Interventional Trials (SPIRIT),[18 19] its extension for Patient Reported Outcomes (SPIRIT-PRO),[20] and the Guidance for Reporting Involvement of Patients and the Public (GRIPP2).[21]

As CASTLE Sleep-E is a pragmatic superiority trial assessing whether UK standard care for children with RE should be augmented with an online behavioural sleep intervention, standard care is the appropriate comparator.[22-24] Current UK clinical guidelines[25-27] recommend that standard care for children with RE consist of a comprehensive care plan with the option of pharmacological treatment with anti-epileptic drugs.

The primary objective of CASTLE Sleep-E is to determine if standard care augmented with COSI is superior to standard care alone in reducing sleep problems in children with RE and cost-effective. Implementation details and secondary objectives are reported in Table 1.

For peer review only

Table 1. Outcomes for CASTLE Sleep-E (incl. participant level metrics, time-points, aggregation method). Child measures may be collected by parent proxy.

Outcome type	Specific measurement variable	Collected for	Participant-level analysis metric	Measurement time-point(s)
Primary				
Clinical	Children's Sleep Habits Questionnaire[28]	Child	Total score	Baseline<br>3 months
Health economic	• Health utility[a]<br>• Cost[bc]	Child and Parent	• Time integral of utility<br>• Total costs	Baseline<br>3 months<br>6 months
Secondary				
Clinical	• Children's Sleep Habits Questionnaire[28]<br>• Health-Related Quality Of Life Measure for Children with Epilepsy[29]	Child	Total score	Baseline<br>6 months
Clinical	Seizure-free period	Child	Time to first seizure from randomisation (days)	Randomisation<br>3 months<br>6 months
Clinical	Seizure remission	Child	Time to 6-months seizure remission from randomisation (days)	Randomisation<br>3 months<br>6 months
Clinical	Knowledge about Sleep in Childhood (unpublished custom-scale)	Parent	Total score	Baseline<br>3 months
Clinical	• Hospital Anxiety and Depression Scale[30]<br>• Insomnia Severity Index[31]	Parent	Total score	Baseline<br>3 months<br>6 months
Clinical	• Strengths and Difficulties Questionnaire[32]<br>• Sickness-related school absences	Child	• Total score<br>• Total number of days	Baseline<br>3 months<br>6 months

[a] Children: CHU-9D, EQ-5D-Y; parents: EQ-5D, Insomnia Severity Index

[b] **Sources for cost calculation (NHS and Personal Social Services):** At randomisation, 3- and 6-months: Resource use questionnaire, Case Report Form data (concomitant medication, Serious Adverse Events); At end of trial: Patient Level Information and Costing System data, Hospital Episode Statistics

[c] **Sources for cost calculation (Indirect and direct non-medical):** At randomisation, 3- and 6-months: Case Report Form (school absences). At baseline, 3- and 6-months: School absences, parental lost productivity [time off work], out of pocket expenses.

For peer review only

Outcome type	Specific measurement variable	Collected for	Participant-level analysis metric	Measurement time-point(s)
Clinical	World Health Organisation – Five Well-Being Index[33]	Parent	Total score	Baseline<br>6 months
Clinical	• Parenting Self Agency Measure[34]	Parent	Total score	Baseline<br>3 months<br>6 months
Clinical	• SleepSuite[35]: Animal task<br>• SleepSuite: Bubble task<br>○ Shape detection<br>○ Emotion detection<br>○ Gender detection<br>• SleepSuite: Maze task	Child	Executive function (accuracy)<br>Reaction time (ms)<br>Variability (Co-efficient of variation [CV])<br><br>Specifically:<br>• Number of correctly named animals (count)<br>• Number of correctly solved mazes (count)<br>• Incidence rate ratio<br>• Mean reaction time (ms)<br>• Accuracy (Misses, False Alarms)<br>• Intra-individual CV	Baseline<br>3 months
Clinical	Actigraphy[36]	Child and Parent	• Total sleep time (minutes)<br>• Sleep latency (minutes)<br>• Sleep efficiency (% asleep of sleep period)<br>All 2-week averages	Baseline<br>3 months
Qualitative	Trial experience[d]	Child and Parent	Interview transcript<br>Activity booklet transcript/photos	3 months + 3 weeks<br>6 months + 3 weeks

[d] **Source data for trial experience**: Qualitative interviews (parents and children), activity booklets (children only)

For peer review only

Outcome type	Specific measurement variable	Collected for	Participant-level analysis metric	Measurement time-point(s)
Process evaluation (context, implementation, and mechanisms of impact)	• COSI[15] system:<br>○ e-analytics<br>○ evaluation module<br><br>• Qualitative trial component	Parent<br><br>Child and Parent	• Module completion (%)<br>• Time spent (minutes)<br>• Intervention experience<br>○ Ease of use (Likert scale)<br>○ Techniques used (binary)<br>○ Technique helpfulness (Likert scale)<br>○ Thoughts and feelings (Likert scale)<br><br>• Interview transcript<br>• Activity booklet transcript/photos	Randomisation to 3 months<br><br>3 months + 3 weeks<br>6 months + 3 weeks

For peer review only

METHODS AND ANALYSES

Trial design

CASTLE Sleep-E is a UK-based, multi-centre, open-label, active concurrent control, randomised (1:1), parallel-group, pragmatic superiority trial. Compared are clinical- and cost-effectiveness of standard care (SC) alone and SC augmented with a novel, tailored, parent-led CASTLE Online Sleep Intervention (SC + COSI) in reducing sleep problems in children (5 to <13 years) with RE at 3- and 6 months after randomisation. Parents and children (≥ 7years) can opt into qualitative interviews and activities to share their experiences and perceptions within 3 weeks of completion of other data collection at 3- and 6 months after randomisation. Jointly, the qualitative trial component and the COSI system (e-analytics and evaluation module) will provide information for a process evaluation (context, implementation, and mechanisms of impact).[37]

Patient and Public Involvement

The CASTLE programme (which subsumes CASTLE Sleep-E) recruited a dedicated Patient and Public Involvement (PPI) Advisory Panel (AP) through social media and epilepsy charities in 2017. The CASTLE Advisory Panel (CAP) consists of 17 adults with experience of childhood epilepsy and five children with epilepsy (aged 6–15 years). CAP has been involved in the research programme from the funding application onward (2 CAP members are co-applicants). Full PPI details are provided in GRIPP2 Short Form in Table 2.

Trial setting and eligibility criteria

Participants will be identified by staff in NHS out-patient general paediatric and paediatric epilepsy clinics in the UK (pre-dominantly urban setting). Eligibility criteria for trial sites include a Capacity and Capability assessment as advised for NHS site set-up by the UK Health Research Authority (HRA). The expected number of trial sites is 40 (England: 34, Scotland: 4, Wales: 1, Northern Ireland: 1). A list of trial sites can be obtained from the Trial Manager (see Supplemental Table 1).

Intervention

Participants will be allocated to trial arms (SC or SC + COSI) using minimisation (1:1 ratio). On allocation to SC + COSI, participants will receive an email with access details to COSI. COSI consists of a self-paced, novel, tailored, e-learning package for parents of children with epilepsy that incorporates evidence-based behavioural components

Supplemental Table 3). CAP feedback and opinion is formally communicated to the CASTLE Sleep-E Trial Management Group (TMG, see Supplemental Table 3) via the CASTLE PPI lead (LB).	
**3: Study results**<br>Outcomes—Report the results of PPI in the study, including both positive and negative outcomes	To date (at the recruitment stage of CASTLE Sleep-E), CAP has contributed to the following trial aspects:<br>**Initial funding application**<br>Two adults with experience of childhood epilepsy are co-applicants on the CASTLE Research Programme NIHR Award (https://tinyurl.com/ycyfkc63)<br>**Trial design**<br>• CAP strongly endorsed the investigation focus (sleep problems) and the focus on non-seizure related issues linked to epilepsy<br>• CAP tested and consulted on the trial intervention (CASTLE Online Sleep Intervention [COSI]) in respect to content, format, and acceptability (e.g. knowledge evaluation quiz was changed from compulsory to optional)<br>• CAP informed the selection of study questionnaires to ensure relevance to parents and children with epilepsy<br>• CAP guided trial design to ensure acceptability of processes (e.g. time, effort, schedule from a family perspective)<br>**Trial procedure**<br>• CAP led the development of a trial flowchart and clinician's guide (top tips for explaining the research to families to aid recruitment)<br>• CAP guided data collection processes (assent/consent procedure, delivery, instructions, and packaging of Actigraphs and iPads)<br>• CAP guided the qualitative interview content and format (e.g. topics, question wording, length, delivery method and format)<br>**Trial materials**<br>• CAP informed the logo design (e.g. CASTLE website https://castlestudy.org.uk/) and name of the CASTLE Sleep-E trial<br>• CAP guided the development of all participant-facing trial materials including:<br>○ Information Sheets and Consent Forms<br>○ Child-friendly postcards to update and maintain interest in the trial<br>○ Wording of trial emails sent to participating families, Strap lines for promotional materials (e.g. mugs and pens for trial sites)<br>**Dissemination**<br>• CAP informed liaison with stakeholders via social media and direct contact (charities, patient groups)<br>• CAP developed lay summaries for completed work as part of the CASTLE programme and helped ensure the CASTLE Sleep-E trial website (https://castlesleepetrial.org.uk/) is accessible to families<br>• CAP informed ongoing work to attract new CAP members
**4: Discussion and conclusions**<br>Outcomes—Comment on the extent to which PPI influenced the study overall. Describe positive and negative outcomes	• To date (recruitment stage of CASTLE Sleep-E), overall **positive outcomes** of CAP contributions to CASTLE Sleep-E have resulted in a trial design, procedure, materials, and dissemination that is likely to have greater appeal and relevance to parents of children affected by Rolandic epilepsy and to the children themselves. CAP has made the trial more family-focused, and enabled more direct public involvement (e.g. contact details of the Family Engagement Officer on the CASTLE Sleep-E webpage). This should increase the proportion of eligible patients to assent/consent to trial participation. Materials (including the trial intervention itself) and procedures should be more accessible and more feasible to complete for participants, which should positively affect adherence, compliance, and retention. Throughout their involvement, CAP contributions to the CASTLE programme have exceeded expectations, and taken on a greater,

For peer review only

	independent purpose (e.g. forming a support group via social media). The Coronavirus (COVID-19) pandemic meant that CAP's work had to move online, and whilst this has facilitated engagement between CAP members across the country, it made it more difficult for the children to join in some of the consultation exercises.
**5: Reflections/critical perspective** Comment critically on the study, reflecting on the things that went well and those that did not, so others can learn from this experience	TBC (currently at recruitment stage of CASTLE Sleep-E)

For peer review only

Table 3. Table 3 provides a brief overview; detailed reports on the development, content, and evaluation of COSI have been published.[15 16] COSI is divided into 13 modules (1 screening for child-specific sleep problems to allow tailoring, 10 content, 1 additional resources, 1 initially hidden evaluation), of which three are compulsory (1 screening, 2 content). The non-compulsory modules are recommended based on screening outcome, but all modules are accessible, repeatable, and printable. The advice in COSI supports parents to implement general prevention techniques (e.g. good sleep hygiene) and specific behavioural change techniques (e.g. bedtime fading) relevant to their child's sleep problems. Three months after first being given access to COSI, parents will be asked by email to complete a COSI evaluation module. At the end of a participant's trial timeline (6 months), access to COSI will be revoked. After the trial, all families (irrespective of trial allocation) have the option to receive the COSI content in electronic format via email.

Fidelity, adherence, retention and acceptability

Fidelity (intervention delivery) will be monitored through e-analytics embedded in the COSI system (modules accessed, and time spent per module). Strategies to improve completion of COSI training in case of non-access include: (1) an automated text-reminder after two days; (2) an email reminder after four days; (3) a phone call from researchers who developed COSI (the Sleep Team) after six days. To improve adherence to the intervention, (1) all participants will receive a phone call from the Sleep Team six weeks after account creation; and (2) children will receive postcards with child-oriented activities (e.g. maze) at three time-points to welcome them to the trial (weeks 1–2), to stay in touch (weeks: 4–5), and to thank them for participating (weeks 4–8 post-trial). To encourage completion of the intervention evaluation, participants will receive: (1) an automated text-reminder after three days of non-completion, (2) and a phone call from the Sleep Team after eight days of non-completion. Fidelity (intervention implementation, acceptability, perceived helpfulness) will be captured jointly by the COSI evaluation module and the qualitative trial component.

Discontinuation, withdrawal, concomitant care or interventions

Participants may discontinue the trial intervention or withdraw from the trial if (1) the parent/child withdraws consent/assent respectively; or (2) a change in the child's condition justifies discontinuation of treatment in their clinician's opinion. Trial site staff will record withdrawal with reason where provided in electronic Case Report Forms (eCRFs). Pseudo-anonymised data up to the time of consent withdrawal will be included in analyses in accordance with General Data Protection Regulation (GDPR)[38] under the UK Data Protection Act 2018[39] — the trial Data Controller relies on the legal bases of 'public interest' and 'research purposes'.

To avoid confounding and to minimise participant burden, co-enrolment into other clinical trials is discouraged. Where recruitment into another trial is considered appropriate, the trial coordinating centre will discuss enrolment with the Chief Investigator (CI). Participation in the Rolandic Epilepsy Genomewide Association International Study (REGAIN: https://childhoodepilepsy.org/research-studies/regain/) is complementary (same CI).

For peer review only

Table 2. GRIPP2- Short Form (SF)21: Guidance for Reporting Involvement of Patients and the Public in research

Section and topic	Item
**1: Aim**<br>Report the aim of PPI in the study	To contribute to and guide the CASTLE Sleep-E study:<br>• To ensure greater relevance and acceptability of the study and study procedures to children with epilepsy and their parents.<br>• To ensure the study is communicated to families and the public in an accessible way (e.g. recruitment, dissemination).
**2: Methods**<br>Provide a clear description of the methods used for PPI in the study	Two adults with experience of childhood epilepsy are co-applicants on the Changing Agendas on Sleep, Treatment and Learning in Childhood Epilepsy (CASTLE) Research Programme National Institute for Health and Care Research (NIHR) Award (https://tinyurl.com/ycyfkc63) and are an integral part of the CASTLE Advisory Panel (CAP). CAP is a dedicated Patient and Public Involvement (PPI) Advisory Panel that was recruited in 2017 through social media and epilepsy charities. CAP consists of 17 adults with experience of childhood epilepsy and five children with epilepsy (aged 6–15 years). CAP members are reimbursed for expenses and offered honorarium payments in acknowledgement of their contributions. Facilitated by a salaried Family Engagement Officer and the PPI lead (LB), CAP members have co-developed working practices (CAP Handbook: Adult version https://tinyurl.com/28u8jex4, child version: https://tinyurl.com/2p8d6bnx) and undertaken research training. CAP members communicate by video conference, telephone, email, social media, and face-to-face. CAP is represented in the Trial Steering Group (TSC, see Supplemental Table 3). CAP feedback and opinion is formally communicated to the CASTLE Sleep-E Trial Management Group (TMG, see Supplemental Table 3) via the CASTLE PPI lead (LB).
**3: Study results**<br>Outcomes—Report the results of PPI in the study, including both positive and negative outcomes	To date (at the recruitment stage of CASTLE Sleep-E), CAP has contributed to the following trial aspects:<br>**Initial funding application**<br>Two adults with experience of childhood epilepsy are co-applicants on the CASTLE Research Programme NIHR Award (https://tinyurl.com/ycyfkc63)<br>**Trial design**<br>• CAP strongly endorsed the investigation focus (sleep problems) and the focus on non-seizure related issues linked to epilepsy<br>• CAP tested and consulted on the trial intervention (CASTLE Online Sleep Intervention [COSI]) in respect to content, format, and acceptability (e.g. knowledge evaluation quiz was changed from compulsory to optional)<br>• CAP informed the selection of study questionnaires to ensure relevance to parents and children with epilepsy<br>• CAP guided trial design to ensure acceptability of processes (e.g. time, effort, schedule from a family perspective)<br>**Trial procedure**<br>• CAP led the development of a trial flowchart and clinician's guide (top tips for explaining the research to families to aid recruitment)<br>• CAP guided data collection processes (assent/consent procedure, delivery, instructions, and packaging of Actigraphs and iPads)<br>• CAP guided the qualitative interview content and format (e.g. topics, question wording, length, delivery method and format)<br>**Trial materials**<br>• CAP informed the logo design (e.g. CASTLE website https://castlestudy.org.uk/) and name of the CASTLE Sleep-E trial

For peer review only

Section and topic	Item
	• CAP guided the development of all participant-facing trial materials including:<br>○ Information Sheets and Consent Forms<br>○ Child-friendly postcards to update and maintain interest in the trial<br>○ Wording of trial emails sent to participating families, Strap lines for promotional materials (e.g. mugs and pens for trial sites)<br>**Dissemination**<br>• CAP informed liaison with stakeholders via social media and direct contact (charities, patient groups)<br>• CAP developed lay summaries for completed work as part of the CASTLE programme and helped ensure the CASTLE Sleep-E trial website (https://castlesleepetrial.org.uk/) is accessible to families<br>• CAP informed ongoing work to attract new CAP members
**4: Discussion and conclusions**<br>Outcomes—Comment on the extent to which PPI influenced the study overall. Describe positive and negative outcomes	• To date (recruitment stage of CASTLE Sleep-E), overall **positive outcomes** of CAP contributions to CASTLE Sleep-E have resulted in a trial design, procedure, materials, and dissemination that is likely to have greater appeal and relevance to parents of children affected by Rolandic epilepsy and to the children themselves. CAP has made the trial more family-focused, and enabled more direct public involvement (e.g. contact details of the Family Engagement Officer on the CASTLE Sleep-E webpage). This should increase the proportion of eligible patients to assent/consent to trial participation. Materials (including the trial intervention itself) and procedures should be more accessible and more feasible to complete for participants, which should positively affect adherence, compliance, and retention. Throughout their involvement, CAP contributions to the CASTLE programme have exceeded expectations, and taken on a greater, independent purpose (e.g. forming a support group via social media). The Coronavirus (COVID-19) pandemic meant that CAP's work had to move online, and whilst this has facilitated engagement between CAP members across the country, it made it more difficult for the children to join in some of the consultation exercises.
**5: Reflections/critical perspective**<br>Comment critically on the study, reflecting on the things that went well and those that did not, so others can learn from this experience	TBC (currently at recruitment stage of CASTLE Sleep-E)

For peer review only

Table 3. Content of the CASTLE Online Sleep Intervention (COSI)

Module	Module Name	Outline content	Compulsory or recommended
A	What is sleep and why is it important	Education about normal sleep physiology and processes	Compulsory
B	Sleep and seizures: a vicious cycle	Information about the relationship between sleep and seizures	Compulsory
C	Personalising this advice for your child	A sleep screening questionnaire to identify key areas of concern or problems around individual child sleep	Compulsory
D	Tips on sleep hygiene for everyone	General advice about key aspects of sleep hygiene	Recommended for all
E	Advanced sleep behaviour training	Introduction to principles of behavioural sleep interventions	Recommended for all
F	Learning difficulties, ADHD, and Autism Spectrum Disorders	Advice for parents of children with other comorbid conditions	Recommended to parents who highlighted (in module C) their child may have comorbid conditions
G	Solving falling asleep problems	Sleep intervention options for typical falling asleep problems	Recommended to parents who highlighted (in module C) their child may have problems falling asleep
H	Solving difficult night wakings and early morning waking	Behavioural techniques to address typical night or early waking problems	Recommended to parents who highlight (in module C) their child may have problems with their sleep during night or early morning wakings
I	Solving night-time fears	Behavioural techniques to address typical night-time fears	Recommended to parents who highlight (in module C) their child may have problems with night-time fears
J	Sleep walking, sleep terrors, and nightmares	Information about different sleep behaviours, what causes them and how to identify and manage different conditions	Recommended to parents who highlight (in module C) their child may have problems with sleep walking, sleep terrors, and/or nightmares
K	Troubleshooting and maintaining good sleep	How to deal with common issues, such as the child being ill or parents' disagreeing about how to manage sleep and advice about how to maintain any benefits	Recommended to all
L	Resources	Links to additional resources of support, information and advice relating to sleep	Recommended to all
M	Evaluation	Questionnaire in which parents are asked to report on their experiences of using COSI	Recommended to all

For peer review only

Outcomes and participant timeline

Outcomes are reported in Table 1 and were chosen collaboratively by children and young people with epilepsy and their parents, sleep- and epilepsy experts[8 17] in accordance with Core Outcome Measures in Effectiveness Trials (COMET) guidelines.[40] Psychometric properties and clinical relevance of outcomes are reported in Supplemental Table 2. Each participant will be followed up for 6 months. The participant timeline is shown in Table 4.

Sample size

The target sample size (110 children with RE, 55 per trial arm) was calculated based on achieving 90 % power to detect the minimal clinically important difference (MCID) in the primary clinical outcome (CSHQ) at 3 months after randomisation, accounting for 10 % expected attrition (non-parametric test with two-sided 5% significance level). MCID was defined based on an individual-focused anchor-based method,[41] that is, 'the smallest difference in outcome that patients perceive as beneficial and which mandates a change in patient management'.[42] The MCID value was based on the estimated reduction in total CSHQ score required for children with epilepsy (M = 48.25, SD = 8.91)[7] to fall at or below the diagnostic cut-off score of 41 for sleep disorders in paediatric populations.[28]

Recruitment, stopping guidelines, interim analyses

An Independent Data and Safety Monitoring Committee (IDSMC) will monitor recruitment and make recommendations to the Trial Steering Committee (TSC) concerning trial continuation, adjustments of recruitment methods, and follow-up optimisation (see Supplemental Table 3). A traffic light approach will determine trial continuation: (1) Green: Continue if at least 30 trial sites have opened and 22 participants have been randomised by end of month 6; (2) Amber: Implement additional recruitment strategies if 15–21 participants have been randomised by end of month 6; (3) Red: If recruitment is <15 participants by end of month 6, then stopping the trial early will be discussed with the TSC. Formal interim analyses of the accumulating data will not be performed.

Treatment allocation

Participants will be allocated with a 1:1 ratio to either SC or SC + COSI based on a computer generated adaptive restricted randomisation procedure that minimises differences between trial arms in variables likely to affect outcomes. The allocation concealment mechanism is an online, central randomisation service implemented and maintained by the Liverpool Clinical Trial Centre (LCTC). The service will be accessed within four weeks of participant enrolment (once consent and eligibility confirmed, Participant ID issued, baseline dataset completed) by trained, authorised staff at trial sites. Randomisation will trigger allocation emails to the Trial Manager at LCTC and to the relevant trial site as well as enable COSI access for participants allocated to the intervention arm. Trial sites will notify the participant's General Practitioner (GP) of the treatment allocation by letter (electronic or hard copy, depending on preference).

Blinding

Only quantitative data analysts will be blinded (Participant IDs do not reveal treatment allocation). All other stakeholders (participants, parents, healthcare providers, data

For peer review only

collectors, qualitative researchers) will be aware of the allocated intervention. Emergency unblinding procedures are therefore unnecessary.

For peer review only

Table 4. CASTLE SLEEP-E participant timeline and order of outcome completion.

	**T-4 weeks**[a] Consent and Baseline	**T0**[b] Randomisation	**T+3 months** Follow up visit	**T+6 months** Follow up visit
**Visit No**	**1**	**2**	**3**	**4**
Informed consent/assent	X			
Review of medical history and EEG[c] results	X			
Eligibility confirmation	X	X		
COVID-19 Screener	X		X	
Review of seizure occurrence		X	X	X
Hospital admissions		X	X	X
Demographics	X			
School absences		X	X	X
Check contact details for accuracy		X	X	X
Child Sleep Habit Questionnaire[28]	X		X	X
SleepSuite[35] (iPad)	X		X	
WHO–Five Well-Being Index[33]	X			X
Health-Related Quality Of Life Measure for Children with Epilepsy[29]	X			X
Strengths and Difficulties Questionnaire[32]	X		X	X
CHU-9D[d]/CHU-9D proxy[43]	X		X	X
EQ-5D-Y/EQ-5D-Y proxy[44]	X		X	X
EQ-5D-5L[45]	X		X	X
Parenting Self Agency Measure[34]	X		X	X
Insomnia Severity Index[31]	X		X	X
Hospital Anxiety and Depression Scale[30]	X		X	X
Resource Use Questionnaire	X		X	X
Knowledge about Sleep in Childhood	X		X	
Randomisation Standard Care (SC) or (SC + COSI[e])[16]		X		
Intervention arm only: COSI[16]		←	—	→
Actigraphy and sleep diary[36] (14 days)	X		X	
Confirm continuing trial participation			X	X
Assessment of Serious Adverse Events			X	X
Completion of Follow-up Case Report Form			X	X
Review of concomitant medications		X	X	X

For peer review only

[a] Up to four weeks flexibility between consent and randomisation to allow delivery of actigraph and iPad.
[b] Randomisation may be performed once two weeks of actigraphy and the minimum dataset are complete.
[c] Electro-encephalogram (EEG)
[d] Child Health Utility Index 9D (CHU-9D)
[e] CASTLE Online Sleep Intervention (COSI)

	T-4 weeks[a] Consent and Baseline	T0[b] Randomisation	T+3 months Follow up visit	T+6 months Follow up visit
**Visit No**	**1**	**2**	**3**	**4**
Qualitative Interview[f]			X	X

For peer review only

[f] Optional trial component: Consenting participants are interviewed within 3 weeks of follow up visits 3 and 4

Assent and consent

Potentially eligible children will be screened at trial centres by trained site staff. Screening outcome will be documented. Eligible children with interested parents will be invited to participate and provided with a Patient Information Sheet and Consent Form electronically and/or hard-copy (PISC, three versions: Parent, child [5–6 and 7–12 years]). Sufficient time will be allowed for discussion of the trial and the decision to assent/consent to trial entry and the optional qualitative component. Assent (children aged 7-12 years) and Consent (parents) may be given face-to-face or remotely and will be electronically captured in a secure Consent Database managed by LCTC. Reasons for declining participation will be asked, but it will be made clear that children and parents do not have to provide a reason.

Data collection and management

Data collection will be carried out electronically except for Serious Adverse Events and Participant Transfer Forms (hard copy). At consent/assent, site staff will enter patient medical history (including electro-encephalogram), eligibility confirmation, COVID-19 screening, and demographics (see Table 4) into eCRFs stored in a secure Data Management System managed by LCTC. Trial participation will be added to the patient's medical records alongside their unique Participant ID.

Consent- and Contacts Databases are securely linked. The addition of a new participant will trigger email notifications to the Parents containing access links to baseline assessments (see Table 4) and the Sleep Team who will access the Contacts Database to arrange the delivery of an iPad pre-configured by LCTC (optionally fitted with pre-paid SIMs), and two actigraphs with supporting documents. iPads (Generations 7–8, iOS 15.2 or 15.3) will be used to access the SleepSuite App, (V 1.4)[35], which assesses executive functions in child-friendly, interactive games (e.g. popping virtual bubbles with smiling children's faces). Access requires the Participant ID and is only possible at pre-specified trial time-points (see Table 4). Data is only stored on the iPad until the test-session completion, then automatically uploaded to a cloud-based server, and then securely downloaded for analyses by authorised LCTC staff. Families lacking other means of internet access can use iPads fitted with pre-paid SIMS to access other online trial materials (including email).

Actigraphs (Micro Motionlogger® Watch and Watchware Software V 1.99.17.4, Ambulatory Monitoring, Inc., NY: USA) will be used to collect 14 days of objective sleep data from child and parent. Concurrent sleep diaries (hard copies) will be completed by the parent with or without child input. At the end of the baseline period, actigraphs will be returned to the Sleep Team via pre-paid courier, and iPads to LCTC via trial sites. The Sleep Team will download and securely store pseudo-anonymised (using Participant IDs) actigraphy data for pre-processing (manual selection of sleep periods cross-checked against sleep diaries) per night at participant-level. Summary variables (sleep latency, total sleep time and sleep efficiency) are then automatically calculated by actigraph software, manually collated and securely transferred electronically to LCTC for trial-level analyses by the Trial Statistician.

Participants will be randomised to trial arms during a telephone/video call or clinic visit only *after* site staff have confirmed that baseline data (see Table 4) is complete, and eligibility, consent/assent, and contact details are still valid. Data collection will be repeated 3- and 6 months after randomisation (see Table 4).

For peer review only - http://bmjopen.bmj.com/site/about/guidelines.xhtml

The Qualitative Research Team will access the Contacts Database to schedule audio-recorded interviews with children and parents who consented/assented to this optional trial component. Interviews (audio- or audio-video) will take place remotely within 3 weeks of completion of other data collection at 3- and 6 months after randomisation. Parents and children will be interviewed together or separately as preferred. Parents and children will have the opportunity to think through their ideas prior to the interview (as proposed by parents and children from the CASTLE Advisory Panel). Children will be invited to complete activity booklets in advance of their interviews (the booklets will be mailed or e-mailed one week prior to their interview); the content they complete will support the interview. Parents will receive a list of proposed questions/topics. Children will be able to share the booklet with the Qualitative Team (e.g. screen or photograph sharing, verbal description).

The direct costs of health and personal social services, and indirect costs of productivity losses and school absenteeism will be collected using a Resource Use Questionnaire administered at baseline and during follow-up visits. Other data such as concomitant medications, study visits and Adverse Events will be collected using eCRFs. Trial participants' use of secondary care services will be collected from Patient-Level Information and Costing Systems (PLICS) data obtained from the finance departments of each recruiting hospital; and based on Hospital Episode Statistics (HES) obtained from NHS Digital at the end of the trial. PLICS and HES data will be pseudo-anonymised and transferred securely to the trial health economists at Bangor University.

Data quality, security, and trial oversight

Reliability, validity, and clinical relevance of outcomes are reported in Supplemental Table 2. Processes to promote quality and security of collected data include general local training of site staff and research teams (Good Clinical Practice); and trial-specific training in the use of electronic forms and databases by LCTC. LCTC will request to see evidence of appropriate training and experience of all trial staff. Staff will be signed off as appropriately qualified by the CI. Electronic data capture provides several in-built validity and security checks (e.g. data type, range, and missingness checks in eCRFs, SleepSuite use/access restrictions). Some electronic and all hard-copy data will be repeat checked (e.g. eligibility, contact details). Data processing requiring more subjective judgement will be performed by minimum of two trained researchers on at least a subset of data (i.e. manually-assisted selection of actigraphy sleep period; thematic and content analysis of qualitative data).

Data will be processed and stored in accordance with GDPR under the UK Data Protection Act 2018. Central data monitoring will be performed by LCTC who will raise and resolve queries with site and research teams within the online system. The University of Liverpool is registered with the Information Commissioners Office. LCTC will receive trial participants' HES identifiers for secure transfer to the Health Economic team, who will access, securely store, and dispose of HES data in accordance with the Bangor University and NHS Digital Data Sharing Framework Contract.

Statistical methods

Statistical analyses of all but health economic and qualitative data will be performed by the Trial Statistician (LCTC) using SAS software, Version 9.4 or later. Intention-To-Treat (ITT) will be the main analysis strategy for primary and secondary outcomes (see Table 1 and Table 5). Statistical significance will be set at the conventional two-sided 5 % level; clinical relevance will be based on previous research (see Supplemental Table 2). Point estimates

with 95 % two-sided confidence intervals will be reported adjusted and unadjusted for covariates. No multiplicity adjustments will be made (only one primary clinical outcome, uncorrected secondary outcome analyses).

Sensitivity analyses will be carried out if the amount of missing data is greater than 10 %. Multiple imputation will be used to assess the robustness of the analysis to missing primary outcome data. The multiple imputation method will follow published guidelines.[46] PROC MI in SAS will be used to generate 50 complete data sets. The imputation model will include all variables included in the primary outcome analysis model. The overall summary adjusted mean difference will be presented with 95 % confidence intervals, to assess the sensitivity of the primary analysis to missing data. All analyses will be reported in accordance with the Consolidated Standards of Reporting Trials Checklist (CONSORT)[47] and regardless of statistical significance.

Health economic evaluation

The economic analysis will be performed by the trial health economists at Bangor University. The primary analysis will adopt an NHS and Personal Social Services perspective and, based on Quality-Adjusted Life Years (QALYs) as a measure of health outcome, estimate the incremental cost-effectiveness ratio from an incremental analysis of the mean costs and QALYs for the intervention and control trial arms.[48] Data assumed to be missing at random will be imputed using multiple imputation by chained equations.[49]

Sensitivity analyses will be conducted to test whether, and to what extent, the incremental cost effectiveness ratio is sensitive to key assumptions in the analysis (e.g. unit prices, different utility estimates CHU-9D[43] vs. EQ-5D-Y[44]). The joint uncertainty in costs and QALYs will be addressed through application of bootstrapping and estimation of cost-effectiveness acceptability curves.[50] Alternative scenarios considering a broader cost perspective (including indirect costs, such as school absences and loss of productivity, valued by reference to published sources), and a range of outcomes (including parental QALYs, measured using the EQ-5D-5L[51] and ISI[31 52]) will be conducted. Inclusion of spillover disutility[53] (impact on parents' utility) will be based on the NICE reference case specification[54] that all QALYs are of equal weight and calculated assuming additive effects. Health-economic findings will be reported according to the Consolidated Health Economic Evaluation Reporting Standards (CHEERS).[55]

Qualitative component

Child and parent interviews will be analysed by the Qualitative Research Team using an interpretive, reflexive, and conceptual analytical approach. Audio-recordings of interviews will be transcribed and thematically analysed in discrete sets (e.g. intervention/control, child/parent, engagement/lack of engagement with intervention, types of decision-making, different responses/experiences). Parent and child transcripts will first be analysed separately, and then as dyads. All data will be used for synthesis. Thematic and content will be used for child activity booklets (text and images). Qualitative- and selected quantitative data (e.g. anxiety measures, actigraphy data) will be compared, as appropriate.

For peer review only

Table 5. Analysis plan for outcome variables in CASTLE Sleep-E. Further analyses details are reported in-text.

**Outcome type**	**Specific measurement variable**	**Hypothesis**	**Method of analysis**
Primary			
Clinical	Children's Sleep Habits Questionnaire[28]	Total score lower in intervention arm at 3 months	Linear mixed effect regression:<br>• Fixed effects: Intervention (binary)<br>• Random effects: Trial site (categorical)<br>• Co-variates:<br>○ Baseline score<br>○ Use of sleep medication (binary)
Health economic	Cost[a] per quality-adjusted life year gained	Incremental Cost Effectiveness Ratio for intervention below NHS threshold	Cost-effectiveness analysis
Secondary			
Clinical	Children's Sleep Habits Questionnaire[28]	Total score lower in intervention arm at 6 months	Linear mixed effect regression (as before)
Clinical	Seizure-free period	Time to first seizure (days) differs between trial arms at 3 and 6 months	Survival analyses<br>• Kaplan-Meier curves by trial arm<br>• Cox proportional hazards regression (if applicable)<br>○ Co-variates:<br>▪ Use of sleep medication (binary)<br>▪ Trial site (categorical)
Clinical	Time to 6-months seizure remission from randomisation (days)	Time to 6-months seizure remission (days) differs between trial arms at 6 months	Survival analyses (as before)
Clinical	• Knowledge about Sleep in Childhood<br>• Actigraphy[36] (2-week average):<br>○ Total sleep time<br>○ Sleep latency<br>○ Sleep efficiency	Total score differs between trial arms at 3 months	Linear mixed effect regression (as before)
Clinical	• Hospital Anxiety and Depression Scale[30]<br>• Insomnia severity index[31]	Total score lower in intervention arm at 3 and 6 months	Linear mixed effect regression (as before)
Clinical	• Sickness-related school absences	Total days differs between trial arms at 3 and 6 months	Poisson mixed-effects regression

[a] **Perspective**: NHS and PSS perspective; **Alternative perspective**: Societal (Indirect and direct non-medical costs)

For peer review only - http://bmjopen.bmj.com/site/about/guidelines.xhtml

Outcome type	Specific measurement variable	Hypothesis	Method of analysis
Clinical	• Health-Related Quality Of Life Measure for Children with Epilepsy[29]<br>• World Health Organisation – Five Well-Being Index[33]	Total score differs between trial arms at 6 months	Linear mixed effect regression (as before)
Clinical	• SleepSuite[35]: Animal task<br>• SleepSuite: Bubble task<br>○ Shape detection<br>○ Emotion detection<br>○ Gender detection<br>• SleepSuite: Maze task	Executive function, reaction time, and variability differ between trials arm at 3 months	• Poisson/zero-inflated negative binomial regression (depending on presence of overdispersion)<br>• 2 x 2 multi-variate repeated-measures Analysis of Variance (ANOVA)<br>Factors: Time (PM/AM) x Intervention (Pre/Post)<br>Fitted per detection task (Shape, Emotion, Gender)<br>• Linear mixed effect regression (as before)
Clinical	• Strengths and Difficulties[32] Questionnaire<br>• Parenting Self Agency Measure[34]	Total score differs between trial arms at 3 and 6 months	Linear mixed effect regression (as before)
Qualitative	Trial experience[b]	Not applicable (inductive)	Thematic analysis (interpretive, reflexive, and conceptual analytical approach)<br>• Discrete sets: Intervention/Control, Child/parent, Engagement with intervention/lack thereof, Decision making types, Responses/experiences<br>• Separately for child and parent, then jointly (dyad)<br>• Comparisons to selective objective data as emerging from analysis (e.g. Anxiety measures, Actigraphy)

[b] **Source data for trial experience**: Qualitative interviews (parents and children individually and as dyad), activity booklets (children only)

For peer review only

Outcome type	Specific measurement variable	Hypothesis	Method of analysis
Process evaluation (context, implementation, and mechanisms of impact).	• COSI[15] system:<br>○ e-analytics<br>○ evaluation module<br><br>• Qualitative trial component: See previous entry	Not applicable (descriptive or inductive)	Descriptive only (no formal statistical testing).<br>• Participant number and percentage who accessed/attempted/completed modules<br>• COSI completion rate (number of quiz questions attempted out of total per module and overall).<br>• Mean and standard deviation of completion percentages will be presented as well as the median and range (minimum and maximum).

For peer review only

Harms

A flowchart of Adverse Event (AE) reporting requirements is shown in Supplemental Figure 1. Harms severity and causality will be graded by the investigator responsible for the care of the participant based on categories shown in Supplemental Table 4. If any doubt about causality exists, the local investigator should inform LCTC who will notify the CI. In case of discrepant views, the Research Ethics Committee (REC) will be informed of both views. Seriousness and expectedness of AEs will be defined based on International Council for Harmonisation of Technical Requirements for Pharmaceuticals for Human Use Definitions and Standards for Expedited Reporting (ICH E2A, ref: CPMP/ICH/377/95). Expectedness will be assessed by the CI. The only expected AEs in CASTLE Sleep-E are mild and transient worsening of sleep behaviours targeted by the trial intervention. Safety data will be quality-checked by a statistician not otherwise involved in the trial. Safety analysis will include all patients randomised and starting treatment and be presented descriptively split by treatment arm.

Auditing

The CI will ensure that the trial team conducts monitoring activities of sufficient quality and quantity (e.g. protocol adherence, consent/assent, data quality). The Sponsor will delegate monitoring duties and activities to LCTC. The CI and LCTC will inform the Sponsor of any concerns. Auditing does not meet the National Institute for Health and Care Research (NIHR) or SPIRIT Statement definitions of independence[19 56] as auditors (LCTC and CI) are part of the trial team.

Protocol amendments

Substantive protocol assentents will be notified to HRA via the UK's Integrated Research Application System (IRAS). Trial sites will receive an amendment pack of HRA- and REC-approved changes and unless an objection is received within 35 days, the trial will continue at site with a GO LIVE email.

Ancillary and post-trial care

King's College London (KCL) holds insurance against claims from participants for harm caused by their participation in this clinical study; compensation can be claimed in case of KCL negligence.

Ethics and dissemination

The CASTLE Sleep-E protocol was approved by the HRA East Midlands – Nottingham 1 REC, reference: 21/EM/0205. Trial results will be disseminated to scientific audiences in peer-reviewed publications and conferences, and — with the help of the CASTLE Advisory Panel (parent and child experts-by-experience), relevant charities (e.g. Epilepsy Action, Epilepsy Society and Cerebra) and professional groups (e.g. Royal College of Paediatrics and Child Health, Epilepsy Specialist Nurses Association) — as plain language summaries to families, other professional groups, managers, commissioners, and policy makers. Pseudo-anonymised Individual Patient Data and associated documentation (e.g. protocol, statistical analysis plan, annotated blank Case Report Form) will be made available after dissemination on reasonable request.

For peer review only

Registration details

ISRCTN registry (Trial ID: ISRCTN13202325, prospective registration 09/Sep/2021). The World Health Organisation Trial Registration Data Set (Version 1.3.1) for CASTLE Sleep-E is shown in Supplemental Table 1.

For peer review only

Author Statement

Contributorship (alphabetic surname order)
Conception of study/award holders: PG, DKP (Chief Investigators); CAP, CT-S, HH, LW, BC, CM, DH, LB (Co-Investigators)
Topic expertise (alphabetic topic order):

- Core outcome set development: CAP, LB, BC, AC, HC, PG, DH, CM, HM, DKP, MR, CT-S, PRW
- Epilepsy expert-by-experience: CAP
- Epilepsy: DKP
- Health economic evaluation: WASH, DH, EW
- Intervention development: GC, PG, HH, DKP, LW
- Patient and Public Involvement (Advisory Panel and Family Engagement): CAP, LB, BC, CM
- Responsibility for Patient-Reported Outcomes: CM
- Programme management: AC
- Qualitative research: CAP, LB, BC, HS
- Sleep: GC, PG, HH, LW
- Statistical analyses: CT-S, VW
- Trial management: NA-N, CS, LS-E

All authors contributed to the design and refinement of the study protocol. The final protocol manuscript was written by KCD, incorporated feedback from DKP, PG, CT-S, LW, BC, CM, DH, LB, GC, LS-E, CS, VW, and was approved for submission by the Chief Investigator (DKP). GRIPP2 content was checked for accuracy by LB. Sponsor name and contact information are provided in Supplemental Table 1. Details of trial committees and other groups and individuals overseeing the trial are listed in Supplemental Table 3. Trial site Principal Investigators will be listed alphabetically in resulting publications as members of the CASTLE Sleep-E Consortium in the Acknowledgements section. There has not been and will not be any use of hired writers.

Funding. This work is supported by the National Institute for Health and Care Research (NIHR), award number RP-PG-0615-20007.

Disclaimer. To avoid potential bias, neither the funder nor the sponsor of this trial has any role in or authority over the design, execution, analyses, interpretation of data, or result dissemination.

Competing interests. None declared.

Patient and Public Involvement. The CASTLE Research Programme has benefitted from extensive Patient and Public Involvement (PPI) via a dedicated CASTLE Advisory Panel from its funding application onward. PPI details are reported in the Methods and Analyses section, subsection Patient and Public Involvement and in Guidance for Reporting Involvement of Patients and the Public (GRIPP2) Short Form in Table 2.

Patient consent for publication. Not applicable.

Provenance and peer review. Not commissioned, externally peer reviewed.

Supplemental material. This content has been supplied by the author(s). It has not been vetted by BMJ Publishing Group Limited (BMJ) and may not have been

For peer review only

peer-reviewed. Any opinions or recommendations discussed are solely those of the author(s) and are not endorsed by BMJ. BMJ disclaims all liability and responsibility arising from any reliance placed on the content. Where the content includes any translated material, BMJ does not warrant the accuracy and reliability of the translations (including but not limited to local regulations, clinical guidelines, terminology, drug names and drug dosages), and is not responsible for any error and/or omissions arising from translation and adaptation or otherwise.

Licence statement. I, the Submitting Author has the right to grant and does grant on behalf of all authors of the Work (as defined in the below author licence), an exclusive licence and/or a non-exclusive licence for contributions from authors who are: i) UK Crown employees; ii) where BMJ has agreed a CC-BY licence shall apply, and/or iii) in accordance with the terms applicable for US Federal Government officers or employees acting as part of their official duties; on a worldwide, perpetual, irrevocable, royalty-free basis to BMJ Publishing Group Ltd ("BMJ") its licensees and where the relevant Journal is co-owned by BMJ to the co-owners of the Journal, to publish the Work in BMJ Open and any other BMJ products and to exploit all rights, as set out in our licence.

The Submitting Author accepts and understands that any supply made under these terms is made by BMJ to the Submitting Author unless you are acting as an employee on behalf of your employer or a postgraduate student of an affiliated institution which is paying any applicable article publishing charge ("APC") for Open Access articles. Where the Submitting Author wishes to make the Work available on an Open Access basis (and intends to pay the relevant APC), the terms of reuse of such Open Access shall be governed by a Creative Commons licence – details of these licences and which Creative Commons licence will apply to this Work are set out in our licence referred to above.

For peer review only

References

1. Collaborators GBDE. Global, regional, and national burden of epilepsy, 1990-2016: a systematic analysis for the Global Burden of Disease Study 2016. *Lancet Neurol* 2019;18(4):357-75. doi: 10.1016/S1474-4422(18)30454-X [published Online First: 20190214]
2. Olusanya BO, Wright SM, Nair MKC, et al. Global Burden of Childhood Epilepsy, Intellectual Disability, and Sensory Impairments. *Pediatrics* 2020;146(1) doi: 10.1542/peds.2019-2623
3. Li Q, Westover MB, Zhang R, et al. Computational Evidence for a Competitive Thalamocortical Model of Spikes and Spindle Activity in Rolandic Epilepsy. *Front Comput Neurosci* 2021;15:680549. doi: 10.3389/fncom.2021.680549 [published Online First: 20210618]
4. Stephen J, Weir CJ, Chin RF. Temporal trends in incidence of Rolandic epilepsy, prevalence of comorbidities and prescribing trends: birth cohort study. *Arch Dis Child* 2020;105(6):569-74. doi: 10.1136/archdischild-2019-318212 [published Online First: 20200114]
5. Ross EE, Stoyell SM, Kramer MA, et al. The natural history of seizures and neuropsychiatric symptoms in childhood epilepsy with centrotemporal spikes (CECTS). *Epilepsy Behav* 2020;103(Pt A):106437. doi: 10.1016/j.yebeh.2019.07.038 [published Online First: 20191020]
6. Petropoulos M-C, Bonaiuto K, Currier J, et al. Practical aspects of childhood epilepsy. *BMJ* 2019;367:l6096. doi: 10.1136/bmj.l6096
7. Larson AM, Ryther RC, Jennesson M, et al. Impact of pediatric epilepsy on sleep patterns and behaviors in children and parents. *Epilepsia* 2012;53(7):1162-9. doi: 10.1111/j.1528-1167.2012.03515.x [published Online First: 20120517]
8. Crudgington H, Rogers M, Bray L, et al. Core Health Outcomes in Childhood Epilepsy (CHOICE): Development of a core outcome set using systematic review methods and a Delphi survey consensus. *Epilepsia* 2019;60(5):857-71. doi: 10.1111/epi.14735 [published Online First: 20190425]
9. Gibbon FM, Maccormac E, Gringras P. Sleep and epilepsy: unfortunate bedfellows. *Archives of Disease in Childhood* 2019;104(2):189-92. doi: 10.1136/archdischild-2017-313421
10. Phillips NL, Moore T, Teng A, et al. Behavioral interventions for sleep disturbances in children with neurological and neurodevelopmental disorders: a systematic review and meta-analysis of randomized controlled trials. *Sleep* 2020;43(9) doi: 10.1093/sleep/zsaa040
11. Tsai S-Y, Lee W-T, Lee C-C, et al. Behavioral-educational sleep interventions for pediatric epilepsy: a randomized controlled trial. *Sleep* 2020;43(1):zsz211. doi: 10.1093/sleep/zsz211
12. Ahorsu DK, Lin CY, Imani V, et al. Testing an app-based intervention to improve insomnia in patients with epilepsy: A randomized controlled trial. *Epilepsy Behav* 2020;112:107371. doi: 10.1016/j.yebeh.2020.107371 [published Online First: 20200827]
13. Condon HE, Maurer LF, Kyle SD. Reporting of adverse events in cognitive behavioural therapy for insomnia: A systematic examination of randomised controlled trials. *Sleep Medicine Reviews* 2021;56:101412. doi: https://doi.org/10.1016/j.smrv.2020.101412
14. Sunnhed R, Hesser H, Andersson G, et al. Comparing internet-delivered cognitive therapy and behavior therapy with telephone support for insomnia disorder: a randomized controlled trial. *Sleep* 2019;43(2) doi: 10.1093/sleep/zsz245
15. Wiggs L, Cook G, Hiscock H, et al. Development and Evaluation of the CASTLE Trial Online Sleep Intervention for Parents of Children with Epilepsy. *Frontiers in Psychology* 2021;12 doi: 10.3389/fpsyg.2021.679804
16. Cook G, Gringras P, Hiscock H, et al. A Qualitative Investigation Into What Parents Want From an Online Behavioural Sleep Intervention for Children With Epilepsy. *Frontiers in Psychology* 2021;12 doi: 10.3389/fpsyg.2021.628605
17. Morris C, Dunkley C, Gibbon FM, et al. Core Health Outcomes In Childhood Epilepsy (CHOICE): protocol for the selection of a core outcome set. *Trials* 2017;18(1):572. doi: 10.1186/s13063-017-2323-7
18. Chan A-W, Tetzlaff JM, Altman DG, et al. SPIRIT 2013 Statement: Defining Standard Protocol Items for Clinical Trials. *Annals of Internal Medicine* 2013;158(3):200-07. doi: 10.7326/0003-4819-158-3-201302050-00583
19. Chan A-W, Tetzlaff JM, Gøtzsche PC, et al. SPIRIT 2013 explanation and elaboration: guidance for protocols of clinical trials. *BMJ : British Medical Journal* 2013;346:e7586. doi: 10.1136/bmj.e7586
20. Calvert M, King M, Mercieca-Bebber R, et al. SPIRIT-PRO Extension explanation and elaboration: guidelines for inclusion of patient-reported outcomes in protocols of clinical trials. *BMJ Open* 2021;11(6):e045105. doi: 10.1136/bmjopen-2020-045105

For peer review only - http://bmjopen.bmj.com/site/about/guidelines.xhtml

21. Staniszewska S, Brett J, Simera I, et al. GRIPP2 reporting checklists: tools to improve reporting of patient and public involvement in research. *BMJ* 2017;358:j3453. doi: 10.1136/bmj.j3453
22. Freedland KE, Mohr DC, Davidson KW, et al. Usual and unusual care: existing practice control groups in randomized controlled trials of behavioral interventions. *Psychosom Med* 2011;73(4):323-35. doi: 10.1097/PSY.0b013e318218e1fb [published Online First: 2011/05/02]
23. Thompson BT, Schoenfeld D. Usual care as the control group in clinical trials of nonpharmacologic interventions. *Proc Am Thorac Soc* 2007;4(7):577-82. doi: 10.1513/pats.200706-072JK
24. Zuidgeest MGP, Welsing PMJ, van Thiel GJMW, et al. Series: Pragmatic trials and real world evidence: Paper 5. Usual care and real life comparators. *Journal of Clinical Epidemiology* 2017;90:92-98. doi: https://doi.org/10.1016/j.jclinepi.2017.07.001
25. National Institute for Health and Care Excellence (NICE). Epilepsies: diagnosis and management (CG137). London: NICE, 2012.
26. Scottish Intercollegiate Guidelines Network (SIGN). Epilepsies in children and young people: investigative procedures and management (SIGN 159). Edinburgh: SIGN, 2020.
27. Health and Social Care Board (HSCB) Northern Ireland. Recommended Clinical Guidelines (CGs) 2022 [updated 19/Jan/2022. Available from: http://www.hscboard.hscni.net/nice/recommended-clinical-guidelines-cgs/ accessed 08/Feb/2022.
28. Owens JA, Spirito A, McGuinn M. The Children's Sleep Habits Questionnaire (CSHQ): psychometric properties of a survey instrument for school-aged children. *Sleep* 2000;23(8):1043-51.
29. Ronen GM, Streiner DL, Rosenbaum P, et al. Health-related quality of life in children with epilepsy: development and validation of self-report and parent proxy measures. *Epilepsia* 2003;44(4):598-612. doi: 10.1046/j.1528-1157.2003.46302.x
30. Zigmond AS, Snaith RP. The hospital anxiety and depression scale. *Acta Psychiatr Scand* 1983;67(6):361-70. doi: 10.1111/j.1600-0447.1983.tb09716.x
31. Morin CM, Belleville G, Bélanger L, et al. The Insomnia Severity Index: psychometric indicators to detect insomnia cases and evaluate treatment response. *Sleep* 2011;34(5):601-08. doi: 10.1093/sleep/34.5.601
32. Goodman R. Psychometric Properties of the Strengths and Difficulties Questionnaire. *Journal of the American Academy of Child & Adolescent Psychiatry* 2001;40(11):1337-45. doi: 10.1097/00004583-200111000-00015
33. Allgaier A-K, Pietsch K, Frühe B, et al. Depression in pediatric care: is the WHO-Five Well-Being Index a valid screening instrument for children and adolescents? *General Hospital Psychiatry* 2012;34(3):234-41. doi: https://doi.org/10.1016/j.genhosppsych.2012.01.007
34. Dumka LE, Stoerzinger HD, Jackson KM, et al. Examination of the Cross-Cultural and Cross-Language Equivalence of the Parenting Self-Agency Measure. *Family Relations* 1996;45(2):216-22. doi: 10.2307/585293
35. Colonna A, Smith AB, Smith S, et al. The Effects of Sleep on Emotional Target Detection Performance: A Novel iPad-Based Pediatric Game. *Frontiers in psychology* 2018;9:241-41. doi: 10.3389/fpsyg.2018.00241
36. Sadaka Y, Sadeh A, Bradbury L, et al. Validation of actigraphy with continuous video-electroencephalography in children with epilepsy. *Sleep Med* 2014;15(9):1075-81. doi: 10.1016/j.sleep.2014.04.021 [published Online First: 20140602]
37. Moore GF, Audrey S, Barker M, et al. Process evaluation of complex interventions: Medical Research Council guidance. *BMJ : British Medical Journal* 2015;350:h1258. doi: 10.1136/bmj.h1258
38. The European Parlament and the Council of the European Union. EU General Data Protection Regulation (GDPR): The European Parlament and the Council of the European Union; 2018 [Available from: https://eur-lex.europa.eu/legal-content/EN/TXT/?uri=CELEX%3A02016R0679-20160504&qid=1532348683434.
39. The Government of the United Kingdom. UK Data Protection Act 2018: The Government of the United Kingdom; 2018 [Available from: https://www.legislation.gov.uk/ukpga/2018/12/contents/enacted.
40. Williamson PR, Altman DG, Bagley H, et al. The COMET Handbook: version 1.0. *Trials* 2017;18(3):280. doi: 10.1186/s13063-017-1978-4
41. Kallogjeri D, Spitznagel EL, Jr, Piccirillo JF. Importance of Defining and Interpreting a Clinically Meaningful Difference in Clinical Research. *JAMA Otolaryngology–Head & Neck Surgery* 2020;146(2):101-02. doi: 10.1001/jamaoto.2019.3744

For peer review only - http://bmjopen.bmj.com/site/about/guidelines.xhtml

42. Jaeschke R, Singer J, Guyatt GH. Measurement of health status. Ascertaining the minimal clinically important difference. *Control Clin Trials* 1989;10(4):407-15. doi: 10.1016/0197-2456(89)90005-6
43. Stevens K. Valuation of the Child Health Utility 9D Index. *Pharmacoeconomics* 2012;30(8):729-47. doi: 10.2165/11599120-000000000-00000
44. Wille N, Badia X, Bonsel G, et al. Development of the EQ-5D-Y: a child-friendly version of the EQ-5D. *Qual Life Res* 2010;19(6):875-86. doi: 10.1007/s11136-010-9648-y [published Online First: 20100420]
45. Devlin NJ, Shah KK, Feng Y, et al. Valuing health-related quality of life: An EQ-5D-5L value set for England. *Health Econ* 2018;27(1):7-22. doi: 10.1002/hec.3564 [published Online First: 20170822]
46. Jakobsen JC, Gluud C, Wetterslev J, et al. When and how should multiple imputation be used for handling missing data in randomised clinical trials – a practical guide with flowcharts. *BMC Medical Research Methodology* 2017;17(1):162. doi: 10.1186/s12874-017-0442-1
47. Schulz KF, Altman DG, Moher D. CONSORT 2010 statement: Updated guidelines for reporting parallel group randomised trials. *J Pharmacol Pharmacother* 2010;1(2):100-07. doi: 10.4103/0976-500X.72352
48. Petrou S, Gray A. Economic evaluation alongside randomised controlled trials: design, conduct, analysis, and reporting. *BMJ* 2011;342:d1548. doi: 10.1136/bmj.d1548 [published Online First: 20110407]
49. Manca A, Palmer S. Handling missing data in patient-level cost-effectiveness analysis alongside randomised clinical trials. *Applied Health Economics and Health Policy* 2005;4(2):65-75. doi: 10.2165/00148365-200504020-00001
50. Fenwick E, Claxton K, Sculpher M. Representing uncertainty: the role of cost-effectiveness acceptability curves. *Health Econ* 2001;10(8):779-87. doi: 10.1002/hec.635
51. Bergmann M, Tschiderer L, Stefani A, et al. Sleep quality and daytime sleepiness in epilepsy: Systematic review and meta-analysis of 25 studies including 8,196 individuals. *Sleep Medicine Reviews* 2021;57:101466. doi: https://doi.org/10.1016/j.smrv.2021.101466
52. Gu NY, Botteman MF, Ji X, et al. Mapping of the Insomnia Severity Index and other sleep measures to EuroQol EQ-5D health state utilities. *Health Qual Life Outcomes* 2011;9:119. doi: 10.1186/1477-7525-9-119 [published Online First: 20111230]
53. Wittenberg E, James LP, Prosser LA. Spillover Effects on Caregivers' and Family Members' Utility: A Systematic Review of the Literature. *PharmacoEconomics* 2019;37(4):475-99. doi: 10.1007/s40273-019-00768-7
54. National Institute for Health and Care Excellence (NICE). NICE health technology evaluations: the manual. Process and methods [PMG36]. London: NICE, 2022.
55. Husereau D, Drummond M, Petrou S, et al. Consolidated Health Economic Evaluation Reporting Standards (CHEERS) statement. *BMJ : British Medical Journal* 2013;346:f1049. doi: 10.1136/bmj.f1049
56. National Institute for Health and Care Excellence (NICE). Clinical Trials Toolkit: Audit. Audit. London: NICE, 2022.

For peer review only

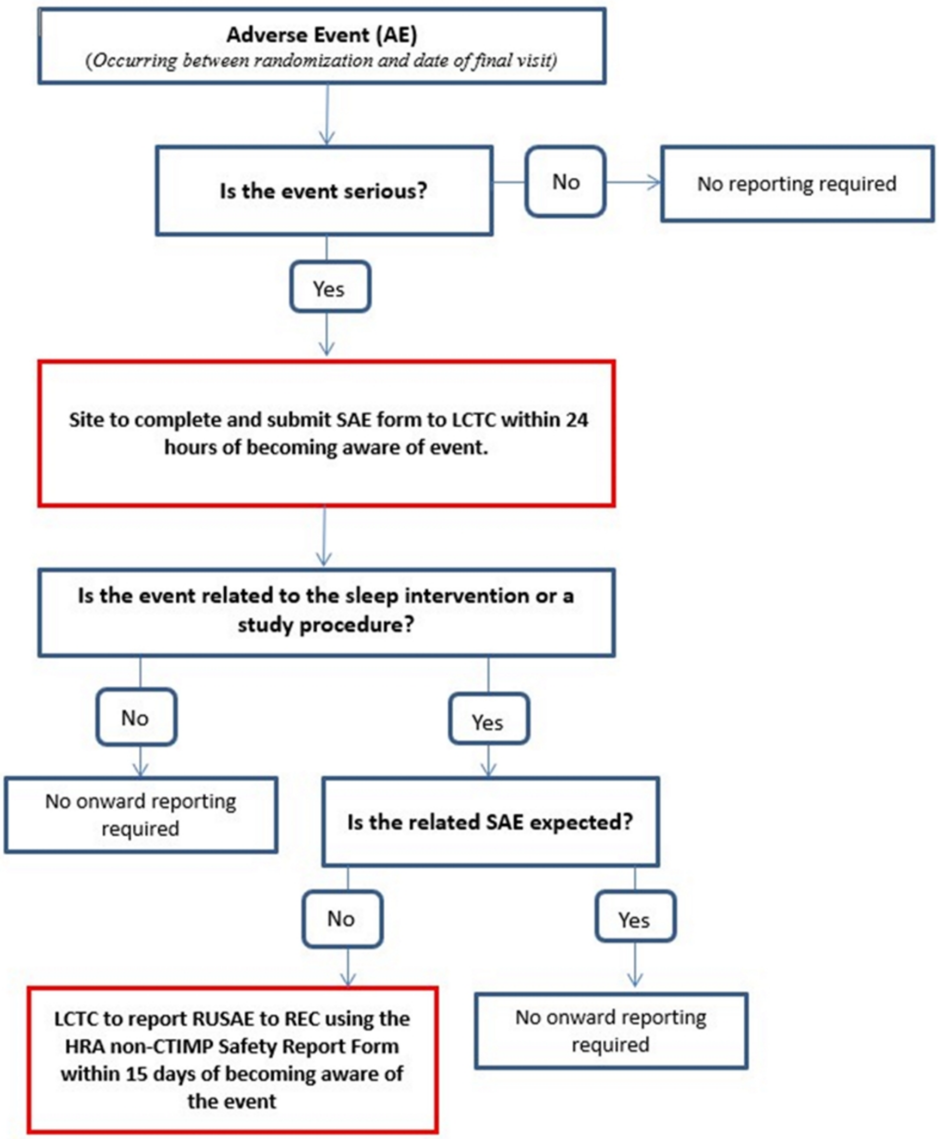

Supplemental Figure 1. Flowchart showing reporting requirements of Adverse Events for the CASTLE Sleep-E trial. Acronyms: Serious Adverse Event (SAE), Liverpool Clinical Trial Centre (LCTC), Related Unexpected Serious Adverse Event (RUSAE), Health Research Authority (HRA), non- Clinical Trial of Investigational Medicinal Products (non-CTIMP).

2184x2612mm (38 x 38 DPI)

Supplemental Table 1. World Health Organization Trial Registration Data Set (Version 1.3.1) for CASTLE Sleep-E

Data category	Information
1. Primary registry and trial identifying number	ISRCTN: ISRCTN13202325
2. Date of registration in primary registry	09 September 2021
3. Secondary identifying numbers	CPMS 50413<br>RP-PG-0615-20007<br>IRAS 289580<br>21/EM/0205
4. Source(s) of monetary or material support	National Institute for Health and Care Research (NIHR)
5. Primary sponsor	Mr Rahman Ahmed<br>Research & Innovation Governance Manager<br>King's College Hospital NHS Foundation Trust<br>161 Denmark Hill<br>London<br>SE5 8EF<br>rahman.ahmed1@nhs.net<br>+44 (0)20 3299 6625
6. Secondary sponsor(s)	Professor Reza Razavi<br>Director of Research Management &<br>Director of Administration (Health Schools)<br>Room 5.31<br>James Clerk Maxwell Building<br>57 Waterloo Road<br>London<br>SE1 8WA<br>reza.razavi@kcl.ac.uk<br>+44 (0)20 7848 3224
7. Contact for public queries	Trial Manager: Lucy Stibbs-Eaton<br>Liverpool Clinical Trials Centre<br>University of Liverpool<br>Liverpool<br>L69 3BX<br>LCTC@liverpool.ac.uk<br>+44 (0)151 795 8751
8. Contact for scientific queries	Professor Deb Pal<br>Professor of Paediatric Epilepsy<br>Maurice Wohl Clinical Neuroscience Institute<br>King's College London<br>5 Cutcombe Road<br>London<br>SE5 9RX<br>deb.pal@kcl.ac.uk<br>+44 (0) 207 848 5762
9. Public title	Changing Agendas on Sleep, Treatment and Learning in Epilepsy (CASTLE) Sleep-E: A randomised controlled trial comparing an online behavioural sleep intervention with standard care in children with Rolandic epilepsy
10. Scientific title	Changing Agendas on Sleep, Treatment and Learning in Epilepsy (CASTLE) Sleep-E: A randomised controlled trial comparing an online behavioural sleep intervention with standard care in children with Rolandic epilepsy

For peer review only - http://bmjopen.bmj.com/site/about/guidelines.xhtml

Data category	Information
11. Countries of recruitment	England<br>Scotland<br>Wales<br>Northern Ireland
12. Health condition(s) or problem(s) studied	Sleep problems in Rolandic epilepsy also known as childhood epilepsy with centro-temporal spikes
13. Intervention(s)	**Intervention arm (SC + COSI)**: Novel, tailored, parent-led CASTLE Online Sleep Intervention (COSI) that incorporates evidence-based behavioural components. Delivered by parents to enrolled children with Rolandic epilepsy in their own homes after completion of self-paced online training. Standard care (SC) is augmented with the CASTLE Online Sleep Intervention (COSI).<br><br>**Active control arm (SC)**: UK National Health Service standard care (SC) for children with Rolandic epilepsy, which consists of a comprehensive care plan with the option of pharmacological treatment with anti-epileptic drugs (first-line mono-therapy with lamotrigine, levetiracetam, oxcarbazepine [girls and boys], carbamazepine or sodium valproate [both boys only]).
14. Key inclusion and exclusion criteria	Inclusion criteria<br>• Children aged 5 to <13 years at the time of randomisation<br>• Formal diagnosis with Rolandic epilepsy based on centro-temporal spikes in electro-encephalogram<br>• Parent-reported child sleep problems over last 2 weeks ranked as mild, moderate, or severe on the Hiscock Australian Global Sleep question<br>• Family has email address and mobile phone<br>• Parent and child have a good enough understanding of the English language to read and answer study questionnaires<br>• Documented parental informed consent<br><br>Exclusion criterion<br>• Formal diagnosis with moderate to severe learning disability
15. Study type	Interventional<br>• Allocation: Minimisation using a bespoke LCTC system<br>Allocation concealment: Central web-interface<br>Sequence generation: Randomised, 1:1 ratio<br>Intervention model: Parallel assignment<br>• Blinding<br>Child, parent, healthcare providers, data collectors, qualitative researchers: None (open label)<br>Quantitative data analysts: Blinded<br>• Primary purpose: Clinical- and cost-effectiveness, process evaluation (qualitative trial component, COSI e-analytics and evaluation module)<br>• Phase: III (behavioural intervention)
16. Date of first enrolment	24/June/2022

For peer review only - http://bmjopen.bmj.com/site/about/guidelines.xhtml

Data category	Information
17. Target sample size	110 (55 children per arm)<br>Calculation based on:<br>• Achieving 90 % statistical power to detect Minimal Clinically Meaningful Difference in primary outcome<br>• 10 % expected attrition
18. Recruitment status	Recruiting (first trial site opened 12/05/2022)
19. Primary outcome(s)	• Clinical: Children's Sleep Habits Questionnaire at 3 months<br>• Health economic: Cost-effectiveness of the intervention over 6 months after randomisation, measured in terms of incremental cost per quality-adjusted life year gained (Child Health Utility instrument or EQ-5D-Y) from the perspective of the National Health Services and Personal Social Services in the UK.<br>• Process evaluation (qualitative trial component, COSI e-analytics and evaluation module)
20. Key secondary outcome(s)	• Clinical Outcome: Sleep problem reduction<br>Metric/method: Children's Sleep Habits Questionnaire<br>Timepoint: 6 months<br>• Clinical Outcome: Seizure frequency reduction<br>Metric/method: Time to first seizure (days)<br>Timepoint: 3 months, 6 months
21. Ethics Review	• Status: Approved<br>• Approval reference: 21/EM/0205<br>• Health Research Authority<br>East Midlands – Nottingham 1 Research Ethics Committee<br>Chair: Mr Paul Hamilton<br>+44 (0) 207 104 8115 or +44 (0) 207 104 8283<br>nottingham1.rec@hra.nhs.uk
22. Completion date	August 2023
23. Summary results	TBC
24. Individual patient data (IPD) sharing statement	• Plan to share IPD: Yes<br>• Plan description: At the end of the trial, after the primary results have been published, the pseudo-anonymised Individual Patient Data and associated documentation (e.g. protocol, statistical analysis plan, annotated blank case report form) will be prepared to be shared with external researchers on reasonable request.
25. Protocol version and date	• Internal protocol: V4.0, 08/12/2021<br>• Manuscript for protocol publication: V1.13, 30/May/2022

For peer review only - http://bmjopen.bmj.com/site/about/guidelines.xhtml

Supplemental Table 2. Psychometrics and clinical relevance/minimal clinically important difference (CR/MCID) for CASTLE Sleep-E outcomes. Metrics refer to the single referenced publication. Further validation studies exist, but, due to differences in population, setting, and/or methods, results cannot be merged.

**Outcome**	**Description**	**Validity**	**Reliability**	**CR/MCID**
Children's Sleep Habits Questionnaire (CSHQ)[1]	Parent-reported, one-week retrospective sleep screening tool for children (4–10 years)<br><br>35 items (2 duplicated across subscales)<br>3-point Likert scales (rarely, sometimes, usually)<br>Total score (33 items): 33–99, lower is better<br>8 subscales:<br>• Bedtime Resistance (6 items)<br>• Sleep Onset Delay (1 item)<br>• Sleep Duration (3 items)<br>• Sleep Anxiety (4 items)<br>• Night Wakings (3 items)<br>• Parasomnias (7 items)<br>• Sleep-Disordered Breathing (3 items)<br>• Daytime Sleepiness (8 items)<br><br>Validation samples<br>Parents of 469 school children (community setting) and 154 children diagnosed with sleep disorder (hospital setting); English language; England, UK.<br>Test-retest: 60 parents from control sample	Classification accuracy<br>Sleep disorder (yes/no)<br>Receiver Operating Characteristic (ROC) analyses: See MCID<br><br>Construct validity<br>See MCID<br><br>Criterion validity<br>Not assessed	Test-retest<br>2-week delay<br>Pearson's *r:* 0.62–0.79<br><br>Internal consistency<br>Cronbach's α<br>Control sample: 0.68<br>Clinical sample: 0.78<br><br>Inter-rater reliability<br>Not assessed	Cut-off (total score): 41<br>• Sensitivity: 80 %<br>• Specificity: 72 %<br>• Accuracy: 80 %<br><br>MCID<br>Not assessed

For peer review only

For peer review only - http://bmjopen.bmj.com/site/about/guidelines.xhtml

Outcome	Description	Validity	Reliability	CR/MCID
SleepSuite[2] (iPad App): Bubble task<br><br>• Executive function (accuracy and response times [RT])	SleepSuite bubble tasks (iPad games) are adapted from a validated Balloon Task[3]: The goal is to burst upward drifting balloons with children's faces under multiple target conditions (e.g. happy faces only) and at increasing presentation conditions (speed, load: number of faces shown simultaneously).<br><br>Validation sample[3]<br>134 healthy children (7–12 years, 58 boys, 23 with clinical behavioural problems, 40% first-born) from middle- and upper-class families of which 25% included at least one parent who immigrated more than 10 years ago. Children lived with their parents in small households (on average 4.53 members). Parents were largely employed full-time (fathers: 90.71%, mothers: 49.31%) and well educated (on average for 16 years). Community setting (school, number unspecified); paid participation ($15 school supply voucher); language: Hebrew, Israel.	Classification accuracy<br>Not assessed<br><br>Construct validity<br>Not assessed<br><br>Criterion validity<br>Child Behavior Checklist (CBCL): total score, sub-scales (8), recode to externalising and internalising behaviours.<br><br>Pearson's *r* (age and sex partialled out), across conditions<br><br>**Completed levels/RT**<br>• Total score: -0.24/ns<br>• Delinquency: ns/0.18<br>• Aggression: -0.20/0.23<br>• Attention problems: -0.18/ns<br>• Social withdrawal: -0.24/ns<br>• Somatic complaints: ns/0.18<br>• Thought disorders: ns/ns<br>• Anxiety-Depression: -.28/ns<br>• Social problems: -0.20/ns<br>• Externalising behaviours: -0.18/0.23<br>• Internalising behaviours: -0.25/ns	Test-retest<br>Delay unspecified (likely none [immediate retest])<br><br>Pearson's *r*<br>• Hits: 0.60<br>• Misses: 0.37<br>• Completed levels: 0.39<br>• RT: 0.78<br><br>Internal consistency<br>Not assessed<br><br>Inter-rater reliability<br>Not assessed	Not assessed

For peer review only - http://bmjopen.bmj.com/site/about/guidelines.xhtml

Outcome	Description	Validity	Reliability	CR/MCID
World Health Organisation – Five Well-Being Index (WHO-5)[4]	Self-reported, two-week retrospective tool to assess subjective psychological well-being in people aged 9 years and older.<br><br>5 items<br>6-point Likert scales (0–5, at no time to all the time)<br>Raw score: 0–25<br>Total score multiplied by 4 to give final score: 0–100, higher is better<br><br>Validation samples<br>446 children analysed (9–12 years, 16 [3.6 %] with depressive disorder), 6 additional participants dropped due to incomplete data.<br>Hospital setting: 3 paediatric hospitals and 3 paediatric surgery hospitals (in- and out-patients for non-psychiatric reasons), Munich, Germany.<br>German language.	Classification accuracy<br>Depressive disorder (yes/no)<br>Receiver Operating Characteristic (ROC) analyses: See CR/MCID<br><br>Construct validity<br>See CR/MCID<br><br>Criterion validity<br>Diagnostic and Statistical Manual of Mental Disorders (DSM-IV) criteria for depressive disorder (major or minor depression only, dysthymia dropped due to mismatch in time-period of concept definitions), see CR/MCID.	Test-retest<br>Not assessed<br><br>Internal consistency<br>Not assessed<br><br>Inter-rater reliability<br>Cohen's κ = .90	Cut-off (total score): 10<br>• Sensitivity: 75 %<br>• Specificity: 92 %<br>• Accuracy: 88 %<br><br>MCID<br>Not assessed

For peer review only

For peer review only - http://bmjopen.bmj.com/site/about/guidelines.xhtml

Outcome	Description	Validity	Reliability	CR/MCID
Health-Related **Q**uality **O**f **L**ife Measure for **Ch**ildren with **E**pilepsy (CHEQOL)[5]	Quality of life assessment tool for children or parents with epilepsy (no specified time-period); child reported if ≥ 8 years, parent proxy-report if child 5 to <8 years<br>25 items<br>4-point Likert scales (0–4, opposites: true/sort of true)<br>Total score: 25–100, higher is better<br>5 subscales (5 items each):<br>• Interpersonal/social consequences<br>• Future worries<br>• Present worries<br>• Intrapersonal/emotional<br>• Epilepsy secrecy<br><br><u>Validation samples</u><br>381 children (6–15 years) with epilepsy and their parents (clinical setting); English language; Ontario, Canada. Test-retest: Additional 89, then 31 children; additional 48 parents.<br>Metrics refer to self-report for children 8–15 years and parent proxy report for children 5 to <8 years and were assessed for sub-scales, not total score.	<u>Classification accuracy</u><br>Not assessed<br><br><u>Construct validity (child)</u><br>Pearson's *r*<br>• Health care utilisation: 0.13–0.31<br>• Drug Adverse Events: 0.18–0.25<br>• Number of friends: 0.18<br>• Nº of extracurricular activities: 0.13<br>One-way ANOVA ($p \leq .05$)<br>• Seizure severity: All 5 subscales<br>• Anti-epileptic drug use: 4 subscales<br>*t–tests* ($p \leq .05$)<br>• Help at school: All 5 subscales<br>Results for parent-proxy similar<br><br><u>Criterion validity</u><br>Not assessed	<u>Test-retest</u><br>10– 14 days delay<br>Intraclass correlation coefficient<br>Child: 0.59–0.69<br>Parent: 0.60–0.81<br><br>*<u>Internal consistency</u>*<br>Cronbach's *α*, subscales<br>*Child: 0.63*–0.84<br>*Parent: 0.64*–0.86<br><br><u>Inter-rater reliability</u><br>Pearson's *r*<br>• Child/mother: 0.24–0.56<br>• Child/father: 0.18–0.54<br>• Mother/father: 0.40–0.71	Not assessed

For peer review only - http://bmjopen.bmj.com/site/about/guidelines.xhtml

Outcome	Description	Validity	Reliability	CR/MCID
Strengths and Difficulties Questionnaire (SDQ)[6]	Parent-, teacher-, or child-reported, retrospective screening tool of child psychopathology (2–18 years). Retrospective period: 6 months or current school year<br><br>25 items<br>3-point Likert scales (0–2, not/somewhat/certainly true)<br>Total score: 0–40, lower is better<br>5 subscales (5 items each):<br>• hyperactivity/inattention,<br>• emotional problems<br>• conduct problems<br>• peer problems<br>• prosocial behaviours (omitted from total score)<br><br><u>Validation samples</u><br>541 children (5–12 years) with and without psychiatric disorders (school setting); multiple languages; Italy, Germany, the Netherlands, Lithuania, Bulgaria, Romania, and Turkey. Metrics refer to parent-report, total score, and data aggregated across countries and psychiatric disorders.	<u>Classification accuracy</u><br>Psychiatric disorder (yes/no)<br>Receiver Operating Characteristic (ROC) analyses: See CR/MCID<br>Original total score cut-offs:<br>• Normal: 0–13<br>• Borderline: 14–16<br>• Abnormal: 17–40<br>transformed to binary:<br>• No: 0–16<br>• Yes: 17–40<br><br><u>Construct validity</u><br>See CR/MCID<br><br><u>Criterion validity</u><br>Diagnostic and Statistical Manual of Mental Disorders (DSM-IV), see CR/MCID.	<u>Test-retest</u><br>Not assessed<br><br><u>Internal consistency</u><br>Cronbach's α: 0.84<br><br><u>Inter-rater reliability</u><br>Not assessed.	Cut-off (total score): 17<br>• Sensitivity: 88 %<br>• Specificity: 59 %<br>• Accuracy: 74 %<br><br><u>MCID</u><br>Not assessed

For peer review only - http://bmjopen.bmj.com/site/about/guidelines.xhtml

Outcome	Description	Validity	Reliability	CR/MCID
Parenting Self Agency Measure (PSAM)[7]	Self-reported tool assessing overall confidence to successfully parent (including managing the child's behaviour and resolving problems with the child). The time-period for parental self-assessment is unspecified.<br><br>5 items<br>7-point Likert scales (1–7, rarely to always)<br>Total score: 5–35, higher is better<br><br>Validation sample<br>90 English-speaking mothers (all European-American, median age 36–40 years, median annual income >$40,000, median education bachelor's degree, 82% married or co-habiting) of 3–12-year-olds (community setting); 2 day-care centres and classes at a large university, 2 churches. English language, southwestern USA.	Classification accuracy<br>Not assessed<br><br>Construct validity<br>Convergent validity<br>Pearson's *r*<br>Active coping: 0.31<br>Parenting acceptance: 0.55<br>Positive re-interpretation: ns<br><br>Discriminant validity<br>Pearson's *r*<br>Inconsistent parental disciplining: -0.34<br>Acceptance coping: ns<br><br>Criterion validity<br>Not assessed	Test-retest<br>Not assessed<br><br>Internal consistency<br>Cronbach's α: 0.70<br>Comparative Fit Index: 0.94<br><br>Inter-rater reliability<br>Not assessed	Not assessed

For peer review only

For peer review only - http://bmjopen.bmj.com/site/about/guidelines.xhtml

Outcome	Description	Validity	Reliability	CR/MCID
Insomnia Severity Index (ISI)[8], patient version	Self-reported, one-month retrospective screening tool for insomnia in adults (≥ 18 years)<br>7 items<br>5-point Likert scales (0–4, no problem to severe problem)<br>Total score: 0–28, lower is better<br>• 0–7: Absence of insomnia<br>• 8–14: Subthreshold insomnia<br>• 15–21: Moderate insomnia<br>• 22–28: Severe insomnia<br>Dimensions:<br>• Severity of sleep onset<br>Sleep maintenance<br>• Early morning awakening problems<br>• Sleep dissatisfaction<br>• Interference of sleep difficulties with daytime functioning<br>• Noticeability of sleep problems by others<br>• Distress caused by the sleep difficulties<br><br>Validation samples<br>959 adults with and without insomnia (community setting), 183 adults with insomnia and 62 controls (clinical setting); English language; Québec, Canada.	Classification accuracy<br>Insomnia (yes/no)<br>ROC analyses, see MCID<br><br>Construct validity<br>See CR/MCID<br>Pearson's *r*<br>• Daily sleep diary: 0.54–0.59<br>• Activity level, Anxiety (state, trait), Depression, Fatigue (general, physical, mental), Motivation: 0.20–0.48<br><br>Criterion validity<br>Pearson's *r*<br>Polysomnography<br>• Sleep onset latency: ns<br>• Wake after sleep onset: ns<br>• Number of awakenings: ns<br>• Early morning awakening: ns<br>• Total wake time: ns<br>• Sleep efficiency: -0.16	Test-retest<br>Not assessed<br><br>Internal consistency<br>Cronbach's α, Control sample: 0.71<br>Clinical sample: 0.73<br><br>Inter-rater reliability<br>Not assessed	Control sample (self-diagnosis)<br>Cut-off (total score): 10<br>• Sensitivity: 86 %<br>• Specificity: 88 %<br>• Accuracy: 87 %<br><br>Clinical sample<br>Cut-off (total score): 11<br>• Sensitivity: 97 %<br>• Specificity: 100%<br>Accuracy: 98 %<br><br>MCID<br>Change required for improvement<br>Blinded assessor, *M*, [CI_{95}]:<br>• Slight: 4.65 [2.61–6.69]<br>• Moderate: 8.36 [7.20–9.53]<br>• Marked: 9.89 [8.74–11.04]<br>ROC analyses:<br>• Slight: not reported<br>• Moderate: ≥ 7<br>○ Sensitivity: 60 %<br>○ Specificity: 70 %<br>○ Accuracy: not reported<br>• Marked: ≥ 8<br>○ Sensitivity: 64 %<br>○ Specificity: 80 %<br>○ Accuracy: not reported

For peer review only

For peer review only - http://bmjopen.bmj.com/site/about/guidelines.xhtml

Outcome	Description	Validity	Reliability	CR/MCID
Hospital Anxiety and Depression Scale (HADS)[9]	Self-reported, one-week retrospective screening tool for anxiety and depression in people aged 16–65.<br><br>5-point Likert scales (0–3)<br>No total score<br>Subscale score: 0–21, lower is better<br>2 subscales (7 items each):<br>• Depression<br>• Anxiety<br><br>Validation samples<br>2 x 50 patients (16–65 years) with and without psychiatric disorders (hospital setting); English language; England, UK.	Classification accuracy<br>Psychiatric interview, see CR/MCID<br><br>Construct validity<br>See CR/MCID<br><br>Convergent validity<br>Spearman's ρ<br>Interview/self-rating<br>Depression/Depression: 0.79<br>Anxiety/Anxiety: 0.54<br><br>Discriminant validity<br>Spearman's ρ<br>Interview/self-rating<br>Depression/Anxiety: ns<br>Anxiety/Depression: ns<br><br>Criterion validity<br>See CR/MCID	Test-retest<br>Not assessed<br><br>Internal consistency<br>Spearman's ρ<br>Anxiety: 0.41–0.76<br>Depression: 0.30–0.60<br><br>Inter-rater reliability<br>Not assessed	Cut-offs (subscales)<br>Depression<br>Absent: $\leq$ 7<br>Borderline: 8–10<br>Definite: $\geq$ 11<br>• False positives: 1 %<br>• False negatives: 1 %<br>Borderline not counted as error<br><br>Anxiety<br>Absent: $\leq$ 7<br>Doubtful: 8–10<br>Definite: $\geq$ 11<br>• False positives: 5 %<br>• False negatives: 1 %<br>Borderline not counted as error<br><br>MCID<br>Not assessed
Knowledge About Sleep in Childhood (KASC)	Self-reported scale assessing parental efficacy in managing child sleep and knowledge about child sleep	Not evaluated (custom-scale devised for CASTLE Sleep-E)	Not evaluated (custom-scale devised for CASTLE Sleep-E)	Not evaluated (custom-scale devised for CASTLE Sleep-E)

For peer review only

For peer review only - http://bmjopen.bmj.com/site/about/guidelines.xhtml

Outcome	Description	Validity	Reliability	CR/MCID
Actigraphy: Micro Motionlogger® Watch, Watchware Software V 1.99.17.4, Action-W software, V 2.7.3285 (Ambulatory Monitoring, Inc., NY: USA) combined with sleep diaries (Child and Parent)<br><br>• Total sleep time (minutes)<br>• Sleep latency (minutes)<br>• Sleep efficiency (% asleep of sleep period)<br><br>All 2-week averages	The Micro-Motionlogger® Watch directly measures 3-D acceleration (in CASTLE Sleep-E and the referenced validation study of the non-dominant wrist). Raw data (zero-crossing mode) is initially recorded as periods of activity and inactivity (1 min epochs), and then recoded into periods of wakefulness and sleep using a combination of proprietary algorithms and manual processing (e.g. sleep periods are visually inspected and manually corrected with the aid of participant sleep diaries). Sleep- and wake parameters are then calculated automatically using validated public algorithms.<br><br><u>Validation sample[3]</u><br>27 children (3–17 years) with medically refractory epilepsy, of which 12 had parent-indicated sleep problems (44%). Hospital setting (in-patient epilepsy monitoring unit in tertiary paediatric hospital), English language, Toronto, Canada.	<u>Classification accuracy</u><br>Not assessed<br><br><u>Construct validity</u><br>Not assessed<br><br><u>Criterion validity</u><br>Agreement of actigraphy with continuous video-electroencephalography (24 hours), scored by neurologist and neurophysiologist.<br><br>Bland-Altman plots in combination with *t*-tests for significant bias:<br>• Total sleep time (minutes): Bias = 8.3 (SD = 31), n.s.<br>• Wake duration: Bias = -4.8 (SD = 31.1), n.s.<br><br>Pearson's *r:*<br>• Total sleep time (minutes): 0.96<br>• Wake duration: 0.93	<u>Test-retest</u><br>Not assessed<br><br><u>Internal consistency</u><br>Not assessed<br><br><u>Inter-rater reliability</u><br>Not assessed	Not assessed

For peer review only - http://bmjopen.bmj.com/site/about/guidelines.xhtml

Supplemental Table 3. Composition, roles and responsibilities of the Trial Management Group, Programme Steering Committee, and Independent Data and Safety Monitoring Committee for CASTLE Sleep-E.

Role	Name (Initials)	Affiliation
**Trial management Group (TMG)**<br>**Responsibilities**: Day-to-day running and management of the trial.<br>**Meeting frequency**: Bi-weekly to three-monthly, depending on trial stage.		
1. King's College Hospital Sponsor Representative	Rahman Ahmed	King's College Hospital NHS Foundation Trust
2. Chief Investigator	Deb K. Pal	King's College London, UK
3. Co-Chief Investigator	Paul Gringras	Evelina London Children's Hospital, UK
4. Co-Investigator<br>Public and Patient Involvement Lead	Lucy Bray	Edge Hill University, UK
5. Co-Investigator<br>Qualitative Research Lead<br>Public and Patient Involvement Co-Lead	Bernie Carter	Edge Hill University, UK
6. Co-Investigator<br>Health Economics Lead	Dyfrig Hughes	Bangor University, UK
7. Co-Investigator<br>Patient Reported Outcome Lead<br>Public and Patient Involvement Co-Lead	Christopher Morris	University of Exeter, UK
8. Co-Investigator<br>Lead Statistician	Catrin Tudur-Smith	University of Liverpool, UK
9. Co-Investigator<br>Intervention Development Lead	Luci Wiggs	Oxford Brookes University, UK
10. Supervising Trials Manager	Catherine Spowart	University of Liverpool, UK
11. Trial Manager	Lucy Stibbs-Eaton	University of Liverpool, UK
12. Trial Statistician	Victoria Watson	University of Liverpool, UK
13. CASTLE Programme Manager	Amber Collingwood	King's College London, UK
14. Researcher	Georgia Cook	Oxford Brookes University, UK
15. Researcher	Kristina C. Dietz	King's College London, UK
16. Health economist	Will A. S. Hardy	Bangor University, UK

For peer review only

Role	Name (Initials)	Affiliation
17. Researcher	Holly Saron	Edge Hill University, UK
**<u>Trial Steering Committee (TSC)</u>**<br>**Responsibilities**: Overall trial supervision and advice, ultimate decision for the continuation of the trial.<br>**Meeting frequency**: At least annually.		
1. Chair	Jeremy Parr	Newcastle University, UK
2. Medical statistician	Martyn Lewis	Keele University, UK
3. Paediatrician	Desaline Joseph	Evelina London Children's Hospital, UK

For peer review only

Role	Name (Initials)	Affiliation
4. Public and Patient Involvement Representative	Jo Conduit-Smith	CASTLE Advisory Panel
**<u>Independent Data and Safety Monitoring Committee (IDSMC)</u>**<br>**Responsibilities:** Interim monitoring of safety and effectiveness, trial conduct and external data. Recommendation to TSC about trial continuation.<br>**Meeting frequency:** At least annually		
1. Chair	Helen Cross	University College London, UK
2. Paediatrician	Alberto Verroti	University of L'aquila, Italy
3. Medical statistician	Anthony Johnson	University College London, UK

For peer review only

Supplemental Table 4. Categories used to define the causality and severity of Adverse Events in CASTLE Sleep-E

**Category**	**Definition**
Causality	
Almost Certainly	There is clear evidence to suggest a causal relationship, and other possible contributing factors can be ruled out.
Probably	There is evidence to suggest a causal relationship, and the influence of other factors is unlikely.
Possibly	There is some evidence to suggest a causal relationship (e.g. the event occurred within a reasonable time after administration of the study procedure). However, the influence of other factors may have contributed to the event (e.g. the participant's clinical condition, other concomitant events).
Unlikely	There is little evidence to suggest there is a causal relationship (e.g. the event did not occur within a reasonable time after administration of the study procedure). There is another reasonable explanation for the event (e.g. the participant's clinical condition).
Not related	There is no evidence of any causal relationship.
Severity	
Mild	The Adverse Event does not interfere with the participant's daily routine and does not require further procedure; it causes slight discomfort.
Moderate	The Adverse Event interferes with some aspects of the participant's routine, or requires further procedure, but is not damaging to health; it causes moderate discomfort.
Severe	The Adverse Event results in alteration, discomfort or disability which is clearly damaging to health.

For peer review only

References

1. Owens JA, Spirito A, McGuinn M. The Children's Sleep Habits Questionnaire (CSHQ): psychometric properties of a survey instrument for school-aged children. *Sleep* 2000;23(8):1043-51.
2. Colonna A, Smith AB, Smith S, et al. The Effects of Sleep on Emotional Target Detection Performance: A Novel iPad-Based Pediatric Game. *Frontiers in psychology* 2018;9:241-41. doi: 10.3389/fpsyg.2018.00241
3. Sadaka Y, Sadeh A, Bradbury L, et al. Validation of actigraphy with continuous video-electroencephalography in children with epilepsy. *Sleep Med* 2014;15(9):1075-81. doi: 10.1016/j.sleep.2014.04.021 [published Online First: 20140602]
4. Allgaier A-K, Pietsch K, Frühe B, et al. Depression in pediatric care: is the WHO-Five Well-Being Index a valid screening instrument for children and adolescents? *General Hospital Psychiatry* 2012;34(3):234-41. doi: https://doi.org/10.1016/j.genhosppsych.2012.01.007
5. Ronen GM, Streiner DL, Rosenbaum P, et al. Health-related quality of life in children with epilepsy: development and validation of self-report and parent proxy measures. *Epilepsia* 2003;44(4):598-612. doi: 10.1046/j.1528-1157.2003.46302.x
6. Goodman R. Psychometric Properties of the Strengths and Difficulties Questionnaire. *Journal of the American Academy of Child & Adolescent Psychiatry* 2001;40(11):1337-45. doi: 10.1097/00004583-200111000-00015
7. Dumka LE, Stoerzinger HD, Jackson KM, et al. Examination of the Cross-Cultural and Cross-Language Equivalence of the Parenting Self-Agency Measure. *Family Relations* 1996;45(2):216-22. doi: 10.2307/585293
8. Morin CM, Belleville G, Bélanger L, et al. The Insomnia Severity Index: psychometric indicators to detect insomnia cases and evaluate treatment response. *Sleep* 2011;34(5):601-08. doi: 10.1093/sleep/34.5.601
9. Zigmond AS, Snaith RP. The hospital anxiety and depression scale. *Acta Psychiatr Scand* 1983;67(6):361-70. doi: 10.1111/j.1600-0447.1983.tb09716.x

For peer review only

STANDARD PROTOCOL ITEMS: RECOMMENDATIONS FOR INTERVENTIONAL TRIALS

SPIRIT 2013 Checklist: Recommended items to address in a clinical trial protocol and related documents*

Section/item	Item No	Description	Addressed on page number
**Administrative information**			
Title	1	Descriptive title identifying the study design, population, interventions, and, if applicable, trial acronym	p1
Trial registration	2a	Trial identifier and registry name. If not yet registered, name of intended registry	p2, Suppl. Table 1 (item 1)
	2b	All items from the World Health Organization Trial Registration Data Set	Suppl. Table 1
Protocol version	3	Date and version identifier	Suppl. Table 1 (item 25)
Funding	4	Sources and types of financial, material, and other support	p22, Suppl. Table 1 (item 4)
Roles and responsibilities	5a	Names, affiliations, and roles of protocol contributors	p1-2, p22
	5b	Name and contact information for the trial sponsor	Suppl. Table 1 (items 5, 6)
	5c	Role of study sponsor and funders, if any, in study design; collection, management, analysis, and interpretation of data; writing of the report; and the decision to submit the report for publication, including whether they will have ultimate authority over any of these activities	p22

For peer review only

	5d	Composition, roles, and responsibilities of the coordinating centre, steering committee, endpoint adjudication committee, data management team, and other individuals or groups overseeing the trial, if applicable (see Item 21a for data monitoring committee)	Suppl. Table 3
**Introduction**			
Background and rationale	6a	Description of research question and justification for undertaking the trial, including summary of relevant studies (published and unpublished) examining benefits and harms for each intervention	p5
	6b	Explanation for choice of comparators	p5
Objectives	7	Specific objectives or hypotheses	Table 5, p18-19
Trial design	8	Description of trial design including type of trial (eg, parallel group, crossover, factorial, single group), allocation ratio, and framework (eg, superiority, equivalence, noninferiority, exploratory)	p8
**Methods: Participants, interventions, and outcomes**			
Study setting	9	Description of study settings (eg, community clinic, academic hospital) and list of countries where data will be collected. Reference to where list of study sites can be obtained	p8
Eligibility criteria	10	Inclusion and exclusion criteria for participants. If applicable, eligibility criteria for study centres and individuals who will perform the interventions (eg, surgeons, psychotherapists)	Suppl. Table 1 (item 14)
Interventions	11a	Interventions for each group with sufficient detail to allow replication, including how and when they will be administered	p8-9, Table 4
	11b	Criteria for discontinuing or modifying allocated interventions for a given trial participant (eg, drug dose change in response to harms, participant request, or improving/worsening disease)	p9
	11c	Strategies to improve adherence to intervention protocols, and any procedures for monitoring adherence (eg, drug tablet return, laboratory tests)	p9

For peer review only - http://bmjopen.bmj.com/site/about/guidelines.xhtml

	11d	Relevant concomitant care and interventions that are permitted or prohibited during the trial	p9
Outcomes	12	Primary, secondary, and other outcomes, including the specific measurement variable (eg, systolic blood pressure), analysis metric (eg, change from baseline, final value, time to event), method of aggregation (eg, median, proportion), and time point for each outcome. Explanation of the clinical relevance of chosen efficacy and harm outcomes is strongly recommended	Table 1, Suppl. Table 2
Participant timeline	13	Time schedule of enrolment, interventions (including any run-ins and washouts), assessments, and visits for participants. A schematic diagram is highly recommended (see Figure)	Table 4
Sample size	14	Estimated number of participants needed to achieve study objectives and how it was determined, including clinical and statistical assumptions supporting any sample size calculations	p13
Recruitment	15	Strategies for achieving adequate participant enrolment to reach target sample size	p13
**Methods: Assignment of interventions (for controlled trials)**			
Allocation:			
Sequence generation	16a	Method of generating the allocation sequence (eg, computer-generated random numbers), and list of any factors for stratification. To reduce predictability of a random sequence, details of any planned restriction (eg, blocking) should be provided in a separate document that is unavailable to those who enrol participants or assign interventions	p13
Allocation concealment mechanism	16b	Mechanism of implementing the allocation sequence (eg, central telephone; sequentially numbered, opaque, sealed envelopes), describing any steps to conceal the sequence until interventions are assigned	p13
Implementation	16c	Who will generate the allocation sequence, who will enrol participants, and who will assign participants to interventions	p13, p8
Blinding (masking)	17a	Who will be blinded after assignment to interventions (eg, trial participants, care providers, outcome assessors, data analysts), and how	p13
	17b	If blinded, circumstances under which unblinding is permissible, and procedure for revealing a participant's allocated intervention during the trial	p13

Methods: Data collection, management, and analysis

Data collection methods	18a	Plans for assessment and collection of outcome, baseline, and other trial data, including any related processes to promote data quality (eg, duplicate measurements, training of assessors) and a description of study instruments (eg, questionnaires, laboratory tests) along with their reliability and validity, if known. Reference to where data collection forms can be found, if not in the protocol	p15-17, Table 4, Suppl. Table 1 (item 8), Suppl. Table 2
	18b	Plans to promote participant retention and complete follow-up, including list of any outcome data to be collected for participants who discontinue or deviate from intervention protocols	p9
Data management	19	Plans for data entry, coding, security, and storage, including any related processes to promote data quality (eg, double data entry; range checks for data values). Reference to where details of data management procedures can be found, if not in the protocol	p15-17, Suppl. Table 1 (item 8)
Statistical methods	20a	Statistical methods for analysing primary and secondary outcomes. Reference to where other details of the statistical analysis plan can be found, if not in the protocol	p16-17, Table 5, Suppl. Table 1 (item 8)
	20b	Methods for any additional analyses (eg, subgroup and adjusted analyses)	p16-17, Table 5
	20c	Definition of analysis population relating to protocol non-adherence (eg, as randomised analysis), and any statistical methods to handle missing data (eg, multiple imputation)	p16-17

Methods: Monitoring

Data monitoring	21a	Composition of data monitoring committee (DMC); summary of its role and reporting structure; statement of whether it is independent from the sponsor and competing interests; and reference to where further details about its charter can be found, if not in the protocol. Alternatively, an explanation of why a DMC is not needed	p22, Suppl. Table 1 (item 8), Suppl . Table 3
	21b	Description of any interim analyses and stopping guidelines, including who will have access to these interim results and make the final decision to terminate the trial	p13, Suppl. Table 3
Harms	22	Plans for collecting, assessing, reporting, and managing solicited and spontaneously reported adverse events and other unintended effects of trial interventions or trial conduct	p20, Suppl. Table 4, Suppl. Figure 1

For peer review only - http://bmjopen.bmj.com/site/about/guidelines.xhtml

Auditing	23	Frequency and procedures for auditing trial conduct, if any, and whether the process will be independent from investigators and the sponsor	p20
**Ethics and dissemination**			
Research ethics approval	24	Plans for seeking research ethics committee/institutional review board (REC/IRB) approval	p3, p20, Suppl. Table 1 (item 21)
Protocol amendments	25	Plans for communicating important protocol modifications (eg, changes to eligibility criteria, outcomes, analyses) to relevant parties (eg, investigators, REC/IRBs, trial participants, trial registries, journals, regulators)	p20
Consent or assent	26a	Who will obtain informed consent or assent from potential trial participants or authorised surrogates, and how (see Item 32)	p15
	26b	Additional consent provisions for collection and use of participant data and biological specimens in ancillary studies, if applicable	N/A
Confidentiality	27	How personal information about potential and enrolled participants will be collected, shared, and maintained in order to protect confidentiality before, during, and after the trial	p15-p16
Declaration of interests	28	Financial and other competing interests for principal investigators for the overall trial and each study site	p22
Access to data	29	Statement of who will have access to the final trial dataset, and disclosure of contractual agreements that limit such access for investigators	p3, p16, Suppl. Table 1 (item 24)
Ancillary and post-trial care	30	Provisions, if any, for ancillary and post-trial care, and for compensation to those who suffer harm from trial participation	p20
Dissemination policy	31a	Plans for investigators and sponsor to communicate trial results to participants, healthcare professionals, the public, and other relevant groups (eg, via publication, reporting in results databases, or other data sharing arrangements), including any publication restrictions	p3, p20
	31b	Authorship eligibility guidelines and any intended use of professional writers	p22
	31c	Plans, if any, for granting public access to the full protocol, participant-level dataset, and statistical code	p3, p20, Suppl. Table 1 (item 24)

For peer review

Appendices

Informed consent materials	32	Model consent form and other related documentation given to participants and authorised surrogates	Suppl. Materials: Patient Information and Consent Sheet
Biological specimens	33	Plans for collection, laboratory evaluation, and storage of biological specimens for genetic or molecular analysis in the current trial and for future use in ancillary studies, if applicable	N/A

*It is strongly recommended that this checklist be read in conjunction with the SPIRIT 2013 Explanation & Elaboration for important clarification on the items. Amendments to the protocol should be tracked and dated. The SPIRIT checklist is copyrighted by the SPIRIT Group under the Creative Commons "Attribution-NonCommercial-NoDerivs 3.0 Unported" license.

For peer review only

The SPIRIT-PRO Protocol Guidance Checklist

Protocol Section	SPIRIT-PRO Item	Recommended Content	Page Addressed
**Administrative Information**			
Roles and responsibilities	SPIRIT-5a-PRO Elaboration	Specify the individual(s) responsible for the PRO content of the trial protocol.	p22
**Introduction**			
Background and rationale	SPIRIT-6a-PRO Extension	Describe the PRO-specific research question and rationale for PRO assessment and summarize PRO findings in relevant studies.	p5, p11
Objectives	SPIRIT-7-PRO Extension	State specific PRO objectives or hypotheses (including relevant PRO concepts/domains).	p5, p13, Table 5, Suppl. Table 2
**Methods: Participants, Interventions, and Outcomes**			
Eligibility criteria	SPIRIT-10-PRO Extension	Specify any PRO-specific eligibility criteria (eg, language/reading requirements or pre-randomization completion of PRO). If PROs will not be collected from the entire study sample, provide a rationale and describe the method for obtaining the PRO subsample.	Suppl. Table 1 (item 14)
Outcomes	SPIRIT-12-PRO Extension	Specify the PRO concepts/domains used to evaluate the intervention (eg, overall health-related quality of life, specific domain, specific symptom) and, for each one, the analysis metric (eg, change from baseline, final value, time to event) and the principal time point or period of interest.	Table 1, Table 4, Table 5
Participant timeline	SPIRIT-13-PRO Extension	Include a schedule of PRO assessments, providing a rationale for the time points, and justifying if the initial assessment is not pre-randomization. Specify time windows, whether PRO collection is prior to clinical assessments, and, if using multiple questionnaires, whether order of administration will be standardized.	Table 4
Sample size	SPIRIT-14-PRO Extension	When a PRO is the primary end point, state the required sample size (and how it was determined) and recruitment target (accounting for expected loss to follow-up). If sample size is not established based on the PRO end point, then discuss the power of the principal PRO analyses.	p13

Calvert M, Kyte D, Mercieca-Bebber R, et al. Guidelines for Inclusion of Patient-Reported Outcomes in Clinical Trial Protocols: The SPIRIT-PRO Extension. *JAMA*. 018;319(5):483-494. doi:10.1001/jama.2017.21903
Note: The SPIRIT-PRO Extension should be used with the SPIRIT 2013 Statement and any other relevant SPIRIT Extensions, found at spirit-statement.org

For peer review only - http://bmjopen.bmj.com/site/about/guidelines.xhtml

The SPIRIT-PRO Protocol Guidance Checklist

Protocol Section	SPIRIT-PRO Item	Recommended Content	Page Addressed
**Methods: Data Collection, Management, and Analysis**			
Data collection methods	SPIRIT-18a(i)-PRO Extension	Justify the PRO instrument to be used and describe domains, number of items, recall period, and instrument scaling and scoring (eg, range and direction of scores indicating a good or poor outcome). Evidence of PRO instrument measurement properties, interpretation guidelines, and patient acceptability and burden should be provided or cited if available, ideally in the population of interest. State whether the measure will be used in accordance with any user manual and specify and justify deviations if planned.	Suppl. Table 2
	SPIRIT-18a(ii)-PRO Extension	Include a data collection plan outlining permitted mode(s) of administration (eg, paper, telephone, electronic, other) and setting (eg, clinic, home, other).	p15-16
	SPIRIT-18a(iii)-PRO Extension	Specify whether more than 1 language version will be used and state whether translated versions have been developed using currently recommended methods.	Suppl. Table 1 (item 14)
	SPIRIT-18a(iv)-PRO Extension	When the trial context requires someone other than a trial participant to answer on his or her behalf (a proxy-reported outcome), state and justify the use of a proxy respondent. Provide or cite evidence of the validity of proxy assessment if available.	Table 1, Table 4
	SPIRIT-18b(i)-PRO Extension	Specify PRO data collection and management strategies for minimizing avoidable missing data.	p9, p13, p15-16, p20
	SPIRIT-18b(ii)-PRO Elaboration	Describe the process of PRO assessment for participants who discontinue or deviate from the assigned intervention protocol.	p9
Statistical methods	SPIRIT-20a-PRO Elaboration	State PRO analysis methods, including any plans for addressing multiplicity/ type I (α) error.	p16-17
	SPIRIT-20c-PRO Elaboration	State how missing data will be described and outline the methods for handling missing items or entire assessments (eg, approach to imputation and sensitivity analyses).	p16-17
**Methods: Monitoring**			
Harms	SPIRIT-22-PRO Extension	State whether or not PRO data will be monitored during the study to inform the clinical care of individual trial participants and, if so, how this will be managed in a standardized way. Describe how this process will be explained to participants; eg, in the participant information sheet and consent form.	p13, p16, p20, Suppl. Table 3, Suppl. Materials: Patient

Calvert M, Kyte D, Mercieca-Bebber R, et al. Guidelines for Inclusion of Patient-Reported Outcomes in Clinical Trial Protocols: The SPIRIT-PRO Extension. *JAMA*. 018;319(5):483-494. doi:10.1001/jama.2017.21903

Note: The SPIRIT-PRO Extension should be used with the SPIRIT 2013 Statement and any other relevant SPIRIT Extensions, found at spirit-statement.org

For peer review only - http://bmjopen.bmj.com/site/about/guidelines.xhtml

The SPIRIT-PRO Protocol Guidance Checklist

			Information and Consent Sheet

Calvert M, Kyte D, Mercieca-Bebber R, et al. Guidelines for Inclusion of Patient-Reported Outcomes in Clinical Trial Protocols: The SPIRIT-PRO Extension. *JAMA*. 018;319(5):483-494. doi:10.1001/jama.2017.21903

Note: The SPIRIT-PRO Extension should be used with the SPIRIT 2013 Statement and any other relevant SPIRIT Extensions, found at spirit-statement.org

For peer review only
For peer review only - http://bmjopen.bmj.com/site/about/guidelines.xhtml

Changing Agendas on Sleep, Treatment and Learning in Epilepsy (CASTLE) Sleep-E: A protocol for a randomised controlled trial comparing an online behavioural sleep intervention with standard care in children with Rolandic epilepsy

Journal:	*BMJ Open*
Manuscript ID	bmjopen-2022-065769.R1
Article Type:	Protocol
Date Submitted by the Author:	21-Nov-2022

Complete List of Authors:	Group, CASTLE; King's College London<br>Advisory Panel, CASTLE; Edge Hill University<br>Al-Najjar, Nadia; University of Liverpool<br>Bray, Lucy; Edge Hill University<br>Carter, Bernie; Edge Hill University<br>Collingwood, Amber; King's College London<br>Cook, Georgia; Oxford Brookes University<br>Crudgington, Holly; King's College London<br>Dietz, Kristina; King's College London<br>Gringras, Paul; Evelina London Children's Healthcare<br>Hardy, Will A. S.; Bangor University<br>Hiscock, Harriet; Murdoch Children's Research Institute<br>Hughes, Dyfrig; Bangor University<br>Morris, Christopher; University of Exeter<br>Pal, Deb; King's College London<br>Rouncefield-Swales, Alison; Edge Hill University<br>Saron, Holly; Edge Hill University<br>Spowart, Catherine; University of Liverpool<br>Stibbs-Eaton, Lucy; University of Liverpool<br>Tudur-Smith, Catrin; University of Liverpool<br>Watson, Victoria; University of Liverpool<br>Whittle, Liam; University of Liverpool<br>Wiggs, Luci; Oxford Brookes University<br>Williamson, Paula; University of Liverpool<br>Wood, Eifiona; Bangor University, CHEME

**Primary Subject Heading**:	Neurology
Secondary Subject Heading:	Paediatrics, Evidence based practice, Health economics, Qualitative research, Patient-centred medicine
Keywords:	Epilepsy < NEUROLOGY, Paediatric neurology < NEUROLOGY, SLEEP MEDICINE, Clinical trials < THERAPEUTICS, QUALITATIVE RESEARCH

TITLE

Changing Agendas on Sleep, Treatment and Learning in Epilepsy (CASTLE) Sleep-E: A protocol for a randomised controlled trial comparing an online behavioural sleep intervention with standard care in children with Rolandic epilepsy

Authorship including affiliations and ORCID IDs (alphabetic surname order)

1. CASTLE Group, King's College London, UK, castlesleepe@liverpool.ac.uk
2. CASTLE Advisory Panel (CAP), CASTLE Research Programme, UK, martinra@edgehill.ac.uk
3. Nadia Al-Najjar (NA-N), University of Liverpool, UK, Nadia.Al-Najjar@liverpool.ac.uk
4. Lucy Bray (LB), Edge Hill University, UK, brayl@edgehill.ac.uk http://orcid.org/0000-0001-8414-3233
5. Bernie Carter (BC), Edge Hill University, UK, bernie.carter@edgehill.ac.uk http://orcid.org/0000-0001-5226-9878
6. Amber Collingwood (AC), King's College London, UK, amber.collingwood@kcl.ac.uk
7. Georgia Cook (GC), Oxford Brookes University, UK, gcook@brookes.ac.uk http://orcid.org/0000-0002-1651-866X
8. Holly Crudgington (HC), King's College London, UK, holly.1.crudgington@kcl.ac.uk http://orcid.org/0000-0003-1048-4953
9. Kristina C. Dietz (KCD), King's College London, UK, kristina.dietz@kcl.ac.uk http://orcid.org/0000-0002-3074-6319
10. Paul Gringras (PG), Evelina London Children's Hospital, UK, paul.gringras@gstt.nhs.uk http://orcid.org/0000-0002-0495-3517
11. Will A. S. Hardy (WASH), Bangor University, UK, w.hardy@bangor.ac.uk http://orcid.org/0000-0001-5227-567X
12. Harriet Hiscock (HH), Murdoch Children's Research Institute, Australia, harriet.hiscock@mcri.edu.au http://orcid.org/0000-0003-3017-2770
13. Dyfrig Hughes (DH) Bangor University, UK, d.a.hughes@bangor.ac.uk http://orcid.org/0000-0001-8247-7459
14. Christopher Morris (CM), University of Exeter, UK, Christopher.Morris@exeter.ac.uk http://orcid.org/0000-0002-9916-507X
15. Deb K. Pal (DKP), King's College London, UK, deb.pal@kcl.ac.uk http://orcid.org/0000-0003-2655-0564
16. Alison Rouncefield-Swales (AR-S), University of Central Lancashire, UK, rouncefia@edgehill.ac.uk, https://orcid.org/0000-0001-9947-7375
17. Holly Saron (HS) Edge Hill University, UK, saronh@edgehill.ac.uk http://orcid.org/0000-0001-7563-3409
18. Catherine Spowart (CS), University of Liverpool, UK Catherine.Spowart@liverpool.ac.uk http://orcid.org/0000-0001-8641-2871
19. Lucy Stibbs-Eaton (LS-E), University of Liverpool, UK, l.stibbs-eaton@liverpool.ac.uk http://orcid.org/0000-0002-3672-4006
20. Catrin Tudur Smith (CTS), University of Liverpool, UK, Cat1@liverpool.ac.uk http://orcid.org/0000-0003-3051-1445
21. Victoria Watson (VW), University of Liverpool, UK, victoria.watson@liverpool.ac.uk
22. Liam Whittle (LWh), University of Liverpool, UK, Liam.Whittle@liverpool.ac.uk, https://orcid.org/0000-0001-8280-1984

For peer review only - http://bmjopen.bmj.com/site/about/guidelines.xhtml

23. Luci Wiggs (LW), Oxford Brookes University, UK, lwiggs@brookes.ac.uk
http://orcid.org/0000-0002-5697-6550
24. Paula R. Williamson (PRW), University of Liverpool, UK, P.R.Williamson@liverpool.ac.uk
25. Eifiona Wood (EW), Bangor University, UK, e.wood@bangor.ac.uk
https://orcid.org/0000-0002-2785-7325

Corresponding author: Dr Kristina C Dietz, Maurice Wohl Clinical Neuroscience Institute, King's College London, 5 Cutcombe Road, London, SE5 9RX, kristina.dietz@kcl.ac.uk, +44 (0) 207 848 025 9

Keywords (Medical Subject Headings 2022): Epilepsy, Rolandic; Sleep; Child; Randomized Controlled Trials as Topic; Evidence-Based Medicine

Word count: 4 000

For peer review only

ABSTRACT

Introduction Sleep and epilepsy have an established bi-directional relationship yet only one randomised controlled clinical trial has assessed the effectiveness of behavioural sleep interventions for children with epilepsy. The intervention was successful, but was delivered via face-to-face educational sessions with parents, which are costly and non-scalable to population level. The Changing Agendas on Sleep, Treatment and Learning in Epilepsy (CASTLE) Sleep-E trial addresses this problem by comparing clinical- and cost-effectiveness in children with Rolandic epilepsy between standard care and standard care augmented with a novel, tailored parent-led CASTLE Online Sleep Intervention (COSI) that incorporates evidence-based behavioural components.

Methods and analyses CASTLE Sleep-E is a UK-based, multi-centre, open label, active concurrent control, randomised, parallel-group, pragmatic superiority trial. A total of 110 children with Rolandic epilepsy will be recruited in out-patient clinics and allocated 1:1 to standard care (SC) or standard care augmented with COSI (SC + COSI). Primary clinical outcome is parent-reported sleep problem score (Children's Sleep Habits Questionnaire). Primary health economic outcome is the Incremental Cost Effectiveness Ratio (National Health Service and Personal Social Services perspective, Child Health Utility 9D instrument). Parents and children (≥ 7 years) can opt into qualitative interviews and activities to share their experiences and perceptions of trial participation and managing sleep with Rolandic epilepsy.

Ethics and dissemination The CASTLE Sleep-E protocol was approved by the Health Research Authority East Midlands (HRA) – Nottingham 1 Research Ethics Committee, reference: 21/EM/0205. Trial results will be disseminated to scientific audiences, families, professional groups, managers, commissioners, and policy makers. Pseudo-anonymised Individual Patient Data will be made available after dissemination on reasonable request.

Registration details ISRCTN registry (Trial ID: ISRCTN13202325, prospective registration 09/Sep/2021). See Supplemental Table 1 for the World Health Organisation Trial Registration Data Set (Version 1.3.1).

For peer review only

Strengths and limitations of this study

- First randomised controlled trial to evaluate the clinical- and cost-effectiveness of a novel, tailored, parent-led CASTLE Online Sleep Intervention (COSI) that incorporates evidence-based behavioural components for children with Rolandic epilepsy
- Extensive Patient and Public Involvement via dedicated CASTLE Advisory Panel
- Embedded health economic evaluation
- **Limitation**: Heavily reliant on parent and child self-report to assess intervention implementation, ameliorated by COSI e-analytics and actigraphy data

For peer review only

INTRODUCTION

Epilepsy is one of the most common long-term neurological conditions worldwide whose prevalence peaks during childhood (5–9 years) and again late in life (over 80 years).[1] Epilepsy in children (5 to <13 years) accounts for the annual loss of over 2.6 million disability-adjusted life years, equivalent to 1.8 % of the global burden of disease from all causes among children and adolescents.[2] Rolandic Epilepsy (RE) is the most common childhood epilepsy.[3]

In the UK, RE has a stable crude incidence rate of 5 in 100 000 children (<16 years) or 542 new cases annually.[4] Concurrent neuro-developmental disorders are very common (35 %).[5] Seizures are often triggered by sleep fragmentation.[6] Many parents co-sleep or monitor children with nocturnal seizures, and children themselves experience a fear of death during and after a seizure.[7] Problems related to sleep emerge as one of the top concerns for both children and parents,[8] but are often unaddressed.[9 10]

A recent systematic review and meta-analysis of clinical trials shows that parent-based behavioural sleep interventions are effective for typically-developing children and those affected by neurological and neuro-developmental disorders.[10] The review concluded that randomised controlled clinical trials assessing functional outcomes (e.g. cognition, emotion, behaviour) and targeting specific populations (e.g. epilepsy) are missing (but see two recent trials).[11 12] Harms capture for cognitive-behavioural and behavioural sleep interventions has been sparse (only 32.3 % of trials address Adverse Events) and predominantly inadequate (92.9 % of trials do not meet adequate reporting criteria).[13] Observed harms of behavioural sleep interventions in adults have been mild (e.g. transient fatigue/exhaustion from sleep restriction in insomnia in 25–33 % of participants).[14] The only published paediatric and adult epilepsy trials did not address harms.[11 12] Based on the existing evidence, the benefits of behavioural sleep interventions in children with epilepsy outweigh potential harms, especially because sleep problems not only affect seizure control, but overall child well-being, learning and memory, and parental quality of life.[9 10] There remains, however, uncertainty whether sleep interventions, which can be resource intensive, are cost-effective in public health systems.

This protocol describes the design for the Changing Agendas on Sleep, Treatment and Learning in Epilepsy (CASTLE) Sleep-E trial, which evaluates the clinical- and cost-effectiveness of a novel, tailored, parent-led CASTLE Online Sleep Intervention (COSI) that incorporates evidence-based behavioural components for children with epilepsy. COSI and CASTLE Sleep-E outcome-selection were co-produced by affected children, young people, and their parents, sleep- and epilepsy experts.[8 15-17] The CASTLE Sleep-E protocol follows Standard Protocol Items: Recommendations for Interventional Trials (SPIRIT),[18 19] its extension for Patient Reported Outcomes (SPIRIT-PRO),[20] and the Guidance for Reporting Involvement of Patients and the Public (GRIPP2).[21]

As CASTLE Sleep-E is a pragmatic superiority trial assessing whether UK standard care for children with RE should be augmented with an online behavioural sleep intervention, standard care is the appropriate comparator.[22-24] Current UK clinical guidelines[25-27] recommend that standard care for children with RE consist of a comprehensive care plan with the option of pharmacological treatment with anti-epileptic drugs.

The primary objective of CASTLE Sleep-E is to determine if standard care augmented with COSI is superior to standard care alone in reducing sleep problems in children with RE and cost-effective. Implementation details and secondary objectives are reported in Table 1.

For peer review only

Table 1. Outcomes for CASTLE Sleep-E (incl. participant level metrics, time-points, aggregation method). Child measures may be collected by parent proxy.

Outcome type	Specific measurement variable	Collected for	Participant-level analysis metric	Measurement time-point(s)
Primary				
1. Clinical	Children's Sleep Habits Questionnaire[28]	Child	Total score	Baseline, 3 months
2. Health economic	Cost utility of COSI[a]: National Health Service and Personal Social Services perspective, using outcomes 13–15	Child and Parent	• Time integral of utility<br>• Total costs	Baseline, 3 months, 6 months, (PLICS and HES at 6 months only)
Secondary				
1. Clinical	Children's Sleep Habits Questionnaire[28]	Child	Total score	Baseline, 6 months
2. Clinical	Seizure-free period	Child	Time to first seizure from randomisation (days)	Randomisation, 3 months, 6 months
3. Clinical	Seizure remission	Child	Time to 6-months seizure remission from randomisation (days)	
4. Clinical	Knowledge about Sleep in Childhood (unpublished custom-scale)	Parent	Total score	Baseline, 3 months
5. Clinical	Hospital Anxiety and Depression Scale[29]	Parent	Total score	Baseline, 3 months, 6 months
6. Clinical	Insomnia Severity Index[30]	Parent	Total score	
7. Clinical	SleepSuite[31] (iPad App)	Child	Reaction time (ms)<br>Executive function (accuracy)	Baseline, 3 months
8. Clinical	• Health-Related Quality Of Life Measure for Children with Epilepsy[32]<br>• World Health Organisation – Five Well-Being Index[33]	Child<br>Parent	• Total score<br>• Total score	Baseline. 6 months
9. Clinical	Strengths and Difficulties Questionnaire[34]	Child	Total score	Baseline, 3 months, 6 months
10. Clinical	Parenting Self Agency Measure[35]	Parent	Total score	
11. Clinical	Actigraphy[36]	Child and Parent	• Total sleep time (minutes)<br>• Sleep latency (minutes)<br>• Sleep efficiency (% asleep of sleep period)<br>All 2-week averages	Baseline, 3 months

[a] Reported as incremental cost per Quality-Adjusted Life Year (QALY) gained

For peer review only

Outcome type	Specific measurement variable	Collected for	Participant-level analysis metric	Measurement time-point(s)
12. Clinical	Sickness-related school absences	Child	Total number of days	Randomisation,3 months, 6 months
13. Health economic	Health-utilities derived from:<br>• EQ-5D-Y[37]<br>• Child Health Utility instrument[38]<br>• EQ-5D-5L[39]	Child and Parent<br>• Child<br>• Child<br>• Parent	Total score<br>• Utility score<br>• Utility score<br>• Utility score	Baseline, 3 months, 6 months
14. Health economic	Insomnia Severity Index mapped to EQ-5D health state utilities[40]	Parent	Total score<br>• Utility score	Baseline, 3 months, 6 months
15. Health economic	Direct costs: National Health Service and Personal Social Services perspective, measured using<br>• Resource Use Questionnaire<br>• Case Report Form data<br>• Patient Level Information and Costing System (PLICS) data<br>• Hospital Episode Statistics (HES) data<br>• Serious Adverse Events (assessed at 3 months, 6 months)	Child	Resource use and total cost	Baseline, 3 months, 6 months, (PLICS and HES at 6 months only)
16. Health economic	Indirect and direct non-medical costs, measured using:<br>• Resource Use Questionnaire<br>• Case Report Form data	Child and Parent	Resource use and total cost	Baseline, 3 months, 6 months
17. Health economic	Cost utility of COSI: Societal perspective, using Quality-Adjusted Life Years and Cost using outcomes 13, 14, and 16	Child and Parent	• Quality-adjusted life years from the time-integral of utility<br>• Mean of total costs	Baseline, 3 months, 6 months
Qualitative	Trial experience	Child and Parent	Qualitative interview transcript<br>Activity booklet transcript/photos	3 months + 3 weeks<br>6 months + 3 weeks

For peer review only

METHODS AND ANALYSES

Trial design

CASTLE Sleep-E is a UK-based, multi-centre, open-label, active concurrent control, randomised (1:1), parallel-group, pragmatic superiority trial (overall trial start date: 14/May/2018, first trial site opened: 12/May/2022, first recruitment: 30/August/2022, planned trial end date: 31/July/2023). Compared are clinical- and cost-effectiveness of standard care (SC) alone and SC augmented with a novel, tailored, parent-led CASTLE Online Sleep Intervention (SC + COSI) in reducing sleep problems in children (5 to <13 years) with RE at 3- and 6 months after randomisation. Parents and children (≥ 7 years) can opt into qualitative interviews and activities to share their experiences and perceptions within 3 weeks of completion of other data collection at 3- and 6 months after randomisation.

Patient and Public Involvement

The CASTLE programme (which subsumes CASTLE Sleep-E) recruited a dedicated Patient and Public Involvement (PPI) Advisory Panel (AP) through social media and epilepsy charities in 2017. The CASTLE Advisory Panel (CAP) consists of 17 adults with experience of childhood epilepsy and five children with epilepsy (aged 6–15 years). CAP has been involved in the research programme from the funding application onward (2 CAP members are co-applicants). Full PPI details are provided in GRIPP2 Short Form in Table 2.

Trial setting and eligibility criteria

Participants will be identified by staff in NHS out-patient general paediatric and paediatric epilepsy clinics in the UK (pre-dominantly urban setting). Eligibility criteria for participants are reported in Supplemental Table 1, as field 14 of the World Health Organisation Trial Registration Data Set (Version 1.3.1). Eligibility criteria for trial sites include a Capacity and Capability assessment as advised for NHS site set-up by the UK HRA. The expected number of trial sites is 40 (England: 34, Scotland: 4, Wales: 1, Northern Ireland: 1). A list of trial sites can be obtained from the Trial Manager (see Supplemental Table 1).

Intervention

Participants will be allocated to trial arms (SC or SC + COSI) using minimisation (1:1 ratio). On allocation to SC + COSI, participants will receive an email with access details to COSI. COSI consists of a self-paced, novel, tailored, e-learning package for parents of children with epilepsy that incorporates evidence-based behavioural components. Table 3 provides a brief overview; detailed reports on the development, content, and evaluation of COSI have been published.[15 16] COSI is divided into 13 modules (1 screening for child-specific sleep problems to allow tailoring, 10 content, 1 additional resources, 1 initially hidden evaluation), of which three are compulsory (1 screening, 2 content). The non-compulsory modules are recommended based on screening outcome, but all modules are accessible, repeatable, and printable. The advice in COSI supports parents to implement general prevention techniques (e.g. good sleep hygiene) and specific behavioural change techniques (e.g. bedtime fading) relevant to their child's sleep problems. Three months after first being given access to COSI, parents will be asked by email to complete a COSI evaluation module. At the end of a participant's trial timeline (6

For peer review only

months), access to COSI will be revoked. After the trial, all families (irrespective of trial allocation) have the option to receive the COSI content in electronic format via email.

Fidelity, adherence, retention, and acceptability

Fidelity (intervention delivery) will be monitored through e-analytics embedded in the COSI system (modules accessed, and time spent per module). Strategies to improve completion of COSI training in case of non-access include: (1) an automated text-reminder after two days; (2) an email reminder after four days; (3) a phone call from researchers who developed COSI (the Sleep Team) after six days. To improve adherence to the intervention, (1) all participants will receive a phone call from the Sleep Team six weeks after account creation; and (2) children will receive postcards with child-oriented activities (e.g. maze) at three time-points to welcome them to the trial (weeks 1–2), to stay in touch (weeks: 4–5), and to thank them for participating (weeks 4–8 post-trial). To encourage completion of the intervention evaluation, participants will receive: (1) an automated text-reminder after three days of non-completion, (2) and a phone call from the Sleep Team after eight days of non-completion. Fidelity (intervention implementation, acceptability, perceived helpfulness) will be captured jointly by the COSI evaluation module and the qualitative trial component.

Discontinuation, withdrawal, concomitant care, or interventions

Participants may discontinue the trial intervention or withdraw from the trial if (1) the parent/child withdraws consent/assent respectively; or (2) a change in the child's condition justifies discontinuation of treatment in their clinician's opinion. Trial site staff will record withdrawal with reason where provided in electronic Case Report Forms (eCRFs). Pseudo-anonymised data up to the time of consent withdrawal will be included in analyses in accordance with General Data Protection Regulation (GDPR)[41] under the UK Data Protection Act 2018[42] — the trial Data Controller relies on the legal bases of 'public interest' and 'research purposes'.

To avoid confounding and to minimise participant burden, co-enrolment into other clinical trials is discouraged. Where recruitment into another trial is considered appropriate, the trial coordinating centre will discuss enrolment with the Chief Investigator (CI). Participation in the Rolandic Epilepsy Genomewide Association International Study (REGAIN: https://childhoodepilepsy.org/research-studies/regain/) is complementary (same CI).

For peer review only

Table 2. GRIPP2- Short Form (SF)[21]: Guidance for Reporting Involvement of Patients and the Public in research

Section and topic	Item
**1: Aim**<br>Report the aim of PPI in the study	To contribute to and guide the CASTLE Sleep-E study:<br>• To ensure greater relevance and acceptability of the study and study procedures to children with epilepsy and their parents.<br>• To ensure the study is communicated to families and the public in an accessible way (e.g. recruitment, dissemination).
**2: Methods**<br>Provide a clear description of the methods used for PPI in the study	Two adults with experience of childhood epilepsy are co-applicants on the Changing Agendas on Sleep, Treatment and Learning in Childhood Epilepsy (CASTLE) Research Programme National Institute for Health and Care Research (NIHR) Award (https://tinyurl.com/ycyfkc63) and are an integral part of the CASTLE Advisory Panel (CAP). CAP is a dedicated Patient and Public Involvement (PPI) Advisory Panel that was recruited in 2017 through social media and epilepsy charities. CAP consists of 17 adults with experience of childhood epilepsy and five children with epilepsy (aged 6–15 years). CAP members are reimbursed for expenses and offered honorarium payments in acknowledgement of their contributions. Facilitated by a salaried Family Engagement Officer and the PPI lead (LB), CAP members have co-developed working practices (CAP Handbook: Adult version https://tinyurl.com/28u8jex4, child version: https://tinyurl.com/2p8d6bnx) and undertaken research training. CAP members communicate by video conference, telephone, email, social media, and face-to-face. CAP is represented in the Trial Steering Group (TSC, see Supplemental Table 4). CAP feedback and opinion is formally communicated to the CASTLE Sleep-E Trial Management Group (TMG, see Supplemental Table 4) via the CASTLE PPI lead (LB).
**3: Study results**<br>Outcomes—Report the results of PPI in the study, including both positive and negative outcomes	To date (at the recruitment stage of CASTLE Sleep-E), CAP has contributed to the following trial aspects:<br>**Initial funding application**<br>Two adults with experience of childhood epilepsy are co-applicants on the CASTLE Research Programme NIHR Award (https://tinyurl.com/ycyfkc63)<br>**Trial design**<br>• CAP strongly endorsed the investigation focus (sleep problems) and the focus on non-seizure related issues linked to epilepsy<br>• CAP tested and consulted on the trial intervention (CASTLE Online Sleep Intervention [COSI]) in respect to content, format, and acceptability (e.g. knowledge evaluation quiz was changed from compulsory to optional)<br>• CAP informed the selection of study questionnaires to ensure relevance to parents and children with epilepsy<br>• CAP guided trial design to ensure acceptability of processes (e.g. time, effort, schedule from a family perspective)<br>**Trial procedure**<br>• CAP led the development of a trial flowchart and clinician's guide (top tips for explaining the trial to families to aid recruitment)<br>• CAP guided data collection processes (assent/consent procedure, delivery of equipment, instructions, and packaging of Actigraphs and iPads)<br>• CAP guided the qualitative interview content and format (e.g. topics, question wording, length, delivery method and format)<br>**Trial materials**<br>• CAP informed the logo design (e.g. CASTLE website https://castlestudy.org.uk/) and name of the CASTLE Sleep-E trial

Section and topic	Item
	• CAP guided the development of all participant-facing trial materials including:<br>○ Information Sheets and Consent Forms<br>○ Child-friendly postcards to update and maintain interest in the trial<br>○ Wording of trial emails sent to participating families, strap lines for promotional materials (e.g. mugs and pens for trial sites)<br>**Dissemination**<br>• CAP informed liaison with stakeholders via social media and direct contact (charities, patient groups)<br>• CAP developed lay summaries for completed work as part of the CASTLE programme and helped ensure the CASTLE Sleep-E trial website (https://castlesleepetrial.org.uk/) is accessible to families<br>• CAP informed ongoing work to attract new CAP members
**4: Discussion and conclusions**<br>Outcomes—Comment on the extent to which PPI influenced the study overall. Describe positive and negative outcomes	• To date (recruitment stage of CASTLE Sleep-E), overall **positive outcomes** of CAP contributions to CASTLE Sleep-E have resulted in a trial design, procedure, materials, and dissemination that is likely to have greater appeal and relevance to parents of children affected by Rolandic epilepsy and to the children themselves. CAP has made the trial more family-focused, and enabled more direct public involvement (e.g. contact details of the Family Engagement Officer on the CASTLE Sleep-E webpage). This should increase the proportion of eligible patients to assent/consent to trial participation. Materials (including the trial intervention itself) and procedures should be more accessible and more feasible to complete for participants, which should positively affect adherence, compliance, and retention. Throughout their involvement, CAP contributions to the CASTLE programme have exceeded expectations, and taken on a greater, independent purpose (e.g. forming a support group via social media). The Coronavirus (COVID-19) pandemic meant that CAP's work had to move online, and whilst this has facilitated engagement between CAP members across the country, it made it more difficult for the children to join in some of the consultation exercises.
**5: Reflections/critical perspective**<br>Comment critically on the study, reflecting on the things that went well and those that did not, so others can learn from this experience	TBC (currently at recruitment stage of CASTLE Sleep-E)

For peer review only

Table 3. Content of the CASTLE Online Sleep Intervention (COSI)

Module	Module Name	Outline content	Compulsory or recommended
A	What is sleep and why is it important	Education about normal sleep physiology and processes	Compulsory
B	Sleep and seizures: a vicious cycle	Information about the relationship between sleep and seizures	Compulsory
C	Personalising this advice for your child	A sleep screening questionnaire to identify key areas of concern or problems around individual child sleep	Compulsory
D	Tips on sleep hygiene for everyone	General advice about key aspects of sleep hygiene	Recommended for all
E	Advanced sleep behaviour training	Introduction to principles of behavioural sleep interventions	Recommended for all
F	Learning difficulties, Attention Deficit Hyperactivity Disorder (ADHD), and Autism Spectrum Disorders	Advice for parents of children with other comorbid conditions	Recommended to parents who highlighted (in module C) their child may have comorbid conditions
G	Solving falling asleep problems	Sleep intervention options for typical falling asleep problems	Recommended to parents who highlighted (in module C) their child may have problems falling asleep
H	Solving difficult night wakings and early morning waking	Behavioural techniques to address typical night or early waking problems	Recommended to parents who highlight (in module C) their child may have problems with their sleep during night or early morning wakings
I	Solving night-time fears	Behavioural techniques to address typical night-time fears	Recommended to parents who highlight (in module C) their child may have problems with night-time fears
J	Sleep walking, sleep terrors, and nightmares	Information about different sleep behaviours, what causes them and how to identify and manage different conditions	Recommended to parents who highlight (in module C) their child may have problems with sleep walking, sleep terrors, and/or nightmares
K	Troubleshooting and maintaining good sleep	How to deal with common issues, such as the child being ill or parents disagreeing about how to manage sleep and advice about how to maintain any benefits	Recommended to all
L	Resources	Links to additional resources of support, information and advice relating to sleep	Recommended to all
M	Evaluation	Questionnaire in which parents are asked to report on their experiences of using COSI	Recommended to all

For peer review only

For peer review only

Outcomes and participant timeline

Outcomes are reported in Table 1 and were chosen collaboratively by children and young people with epilepsy and their parents, sleep- and epilepsy experts[8 17] in accordance with Core Outcome Measures in Effectiveness Trials (COMET) guidelines.[43] Psychometric properties and clinical relevance of outcomes are reported in Supplemental Table 2. Each participant will be followed up for 6 months. The participant timeline is shown in Table 4. The estimated time-requirement of CASTLE Sleep-E participation is shown in Supplemental Table 3.

Sample size

The target sample size (110 children with RE, 55 per trial arm) was calculated based on achieving 90 % power to detect the minimal clinically important difference (MCID) in the primary clinical outcome (CSHQ) at 3 months after randomisation, accounting for 10 % expected attrition (non-parametric test with two-sided 5% significance level). MCID was defined based on an individual-focused anchor-based method,[44] that is, 'the smallest difference in outcome that patients perceive as beneficial and which mandates a change in patient management'.[45] The MCID value was based on the estimated reduction in total CSHQ score required for children with epilepsy (M = 48.25, SD = 8.91)[7] to fall at or below the diagnostic cut-off score of 41 for sleep disorders in paediatric populations.[28]

Recruitment, stopping guidelines, interim analyses

An Independent Data and Safety Monitoring Committee (IDSMC) will monitor recruitment and make recommendations to the Trial Steering Committee (TSC) concerning trial continuation, adjustments of recruitment methods, and follow-up optimisation (see Supplemental Table 4). A traffic light approach will determine trial continuation: (1) Green: Continue if at least 30 trial sites have opened and 22 participants have been randomised by end of month 6; (2) Amber: Implement additional recruitment strategies if 15–21 participants have been randomised by end of month 6; (3) Red: If recruitment is <15 participants by end of month 6, then stopping the trial early will be discussed with the TSC. Formal interim analyses of the accumulating data will not be performed.

Treatment allocation

Participants will be allocated with a 1:1 ratio to either SC or SC + COSI based on a computer generated adaptive restricted randomisation procedure that minimises differences between trial arms in variables likely to affect outcomes. The allocation concealment mechanism is an online, central randomisation service implemented and maintained by the Liverpool Clinical Trial Centre (LCTC). The service will be accessed within four weeks of participant enrolment (once consent and eligibility confirmed, Participant ID issued, baseline dataset completed) by trained, authorised staff at trial sites. Randomisation will trigger allocation emails to the Trial Manager at LCTC and to the relevant trial site as well as enable COSI access for participants allocated to the intervention arm. Trial sites will notify the participant's General Practitioner (GP) of the treatment allocation by letter (electronic or hard copy, depending on preference).

For peer review only

Blinding

Only quantitative data analysts will be blinded (Participant IDs do not reveal treatment allocation). All other stakeholders (participants, parents, healthcare providers, data collectors, qualitative researchers) will be aware of the allocated intervention. Emergency unblinding procedures are therefore unnecessary.

Table 4. CASTLE SLEEP-E participant timeline and order of outcome completion.

	**T-4 weeks**[a] Consent and Baseline	**T0**[b] Randomisation	**T+3 months** Follow up visit	**T+6 months** Follow up visit
**Visit No**	**1**	**2**	**3**	**4**
Informed consent/assent	X			
Review of medical history and EEG[c] results	X			
Eligibility confirmation	X	X		
COVID-19 Screener	X		X	
Review of seizure occurrence		X	X	X
Hospital admissions		X	X	X
Demographics	X			
School absences		X	X	X
Check contact details for accuracy		X	X	X
Children's Sleep Habit Questionnaire[28]	X		X	X
SleepSuite[31] (iPad)	X		X	
WHO–Five Well-Being Index[33]	X			X
Health-Related Quality Of Life Measure for Children with Epilepsy[32]	X			X
Strengths and Difficulties Questionnaire[34]	X		X	X
CHU-9D[d]/CHU-9D proxy[38]	X		X	X
EQ-5D-Y/EQ-5D-Y proxy[37]	X		X	X
EQ-5D-5L[39]	X		X	X
Parenting Self Agency Measure[35]	X		X	X
Insomnia Severity Index[30]	X		X	X
Hospital Anxiety and Depression Scale[29]	X		X	X
Resource Use Questionnaire	X		X	X
Knowledge about Sleep in Childhood	X		X	
Randomisation<br>Standard Care (SC) or (SC + COSI[e])[16]		X		
Intervention arm only: COSI[16]		⟵	⟶	⟶
Actigraphy and sleep diary[36] (14 days)	X		X	

[a] Up to four weeks flexibility between consent and randomisation to allow delivery of actigraph and iPad.
[b] Randomisation may be performed once two weeks of actigraphy and the minimum dataset are complete.
[c] Electro-encephalogram (EEG)
[d] Child Health Utility Index 9D (CHU-9D)
[e] CASTLE Online Sleep Intervention (COSI)

For peer review only

	T-4 weeks[a] Consent and Baseline	T0[b] Randomisation	T+3 months Follow up visit	T+6 months Follow up visit
**Visit No**	**1**	**2**	**3**	**4**
Confirm continuing trial participation			X	X
Assessment of Serious Adverse Events			X	X
Completion of Follow-up Case Report Form			X	X
Review of concomitant medications		X	X	X
Qualitative Interview[f]			X	X

For peer review only

[f] Optional trial component: Consenting participants are interviewed within 3 weeks of follow up visits 3 and 4

Assent and consent

Potentially eligible children will be screened at trial centres by trained site staff. Screening outcome will be documented. Eligible children with interested parents will be invited to participate and provided with a Patient Information Sheet and Consent Form electronically and/or hard-copy (PISC, three versions: Parent, child [5–6 and 7–12 years]). Sufficient time will be allowed for discussion of the trial and the decision to assent/consent to trial entry and the optional qualitative component. Assent (children aged 7-12 years) and consent (parents) may be given face-to-face or remotely and will be electronically captured in a secure Consent Database managed by LCTC. Reasons for declining participation will be asked, but it will be made clear that children and parents do not have to provide a reason.

Data collection and management

Data collection will be carried out electronically except for Serious Adverse Events and Participant Transfer Forms (hard copy). At consent/assent, site staff will enter patient medical history (including electro-encephalogram), eligibility confirmation, COVID-19 screening, and demographics (see Table 4) into eCRFs stored in a secure Data Management System managed by LCTC. Trial participation will be added to the patient's medical records alongside their unique Participant ID.

Consent- and Contacts Databases are securely linked. The addition of a new participant will trigger email notifications to the parents containing access links to baseline assessments (see Table 4) and the Sleep Team who will access the Contacts Database to arrange the delivery of an iPad pre-configured by LCTC (optionally fitted with pre-paid SIMs), and two actigraphs with supporting documents. iPads (Generations 7–8, iOS 15.2 or 15.3) will be used to access the SleepSuite App, (V 1.4)[31], which assesses executive functions in child-friendly, interactive games (e.g. popping virtual bubbles with smiling children's faces). Access requires the Participant ID and is only possible at pre-specified trial time-points (see Table 4). Data is only stored on the iPad until the test-session completion, then automatically uploaded to a cloud-based server, and then securely downloaded for analyses by authorised LCTC staff. Families lacking other means of internet access can use iPads fitted with pre-paid SIMS to access other online trial materials (including email).

Actigraphs (Micro Motionlogger® Watch and Watchware Software V 1.99.17.4, Ambulatory Monitoring, Inc., NY: USA) will be used to collect 14 days of objective sleep data from child and parent. Concurrent sleep diaries (hard copies) will be completed by the parent with or without child input. At the end of the baseline period, actigraphs will be returned to the Sleep Team via pre-paid courier. The Sleep Team will download and securely store pseudo-anonymised (using Participant IDs) actigraphy data for pre-processing (manual selection of sleep periods cross-checked against sleep diaries) per night at participant-level. Summary variables (sleep latency, total sleep time and sleep efficiency) are then automatically calculated by actigraph software, manually collated, and securely transferred electronically to LCTC for trial-level analyses by the Trial Statistician.

Participants will be randomised to trial arms during a telephone/video call or clinic visit only *after* site staff have confirmed that baseline data (see Table 4) is complete, and eligibility, consent/assent, and contact details are still valid. Data collection will be repeated 3- and 6 months after randomisation, and iPads to LCTC via trial sites (see Table 4).

The Qualitative Research Team will access the Contacts Database to schedule audio-recorded interviews with children and parents who consented/assented to this optional trial

For peer review only

component. Interviews (audio- or audio-video) will take place remotely within 3 weeks of completion of other data collection at 3- and 6 months after randomisation. Parents and children will be interviewed together or separately as preferred. Parents and children will have the opportunity to think through their ideas prior to the interview (as proposed by parents and children from the CASTLE Advisory Panel). Children will be invited to complete activity booklets in advance of their interviews (the booklets will be mailed or emailed one week prior to their interview); the content they complete will support the interview. Parents will receive a list of proposed questions/topics. Children will be able to share the booklet with the Qualitative Team (e.g. screen or photograph sharing, verbal description).

The direct costs of health and personal social services, and indirect costs of productivity losses and school absenteeism will be collected using a Resource Use Questionnaire administered at baseline and during follow-up visits. Other data such as concomitant medications, study visits and Adverse Events will be collected using eCRFs. Trial participants' use of secondary care services will be collected from Patient-Level Information and Costing Systems (PLICS) data obtained from the finance departments of each recruiting hospital or from Hospital Episode Statistics (HES) data obtained from NHS Digital at the end of the trial. PLICS and HES data will be pseudo-anonymised and transferred securely to the trial health economists at Bangor University.

Data quality, security, and trial oversight

Reliability, validity, and clinical relevance of outcomes are reported in Supplemental Table 2. Processes to promote quality and security of collected data include general local training of site staff and research teams (Good Clinical Practice); and trial-specific training in the use of electronic forms and databases by LCTC. LCTC will request to see evidence of appropriate training and experience of all trial staff. Staff will be signed off as appropriately qualified by the CI. Electronic data capture provides several in-built validity and security checks (e.g. data type, range, and missingness checks in eCRFs, SleepSuite use/access restrictions). Some electronic and all hard-copy data will be repeat checked (e.g. eligibility, contact details). Data processing requiring more subjective judgement will be performed by minimum of two trained researchers on at least a subset of data (i.e. manually-assisted selection of actigraphy sleep period; thematic and content analysis of qualitative data).

Data will be processed and stored in accordance with GDPR under the UK Data Protection Act 2018. Central data monitoring will be performed by LCTC who will raise and resolve queries with site and research teams within the online system. The University of Liverpool is registered with the Information Commissioners Office. LCTC will receive trial participants' HES identifiers for secure transfer to the Health Economic team, who will access, securely store, and dispose of HES data in accordance with the Bangor University and NHS Digital Data Sharing Framework Contract.

Statistical methods

Statistical analyses of all but health economic and qualitative data will be performed by the Trial Statistician (LCTC) using SAS software, Version 9.4 or later. Intention-To-Treat (ITT) will be the main analysis strategy for primary and secondary outcomes (see Table 1 and Table 5). Statistical significance will be set at the conventional two-sided 5 % level; clinical relevance will be based on previous research (see Supplemental Table 2). Point estimates with 95 % two-sided confidence intervals will be reported adjusted and unadjusted for

For peer review only

covariates. No multiplicity adjustments will be made (only one primary clinical outcome, uncorrected secondary outcome analyses).

Sensitivity analyses will be carried out if the amount of missing data is greater than 10 %. Multiple imputation will be used to assess the robustness of the analysis to missing primary outcome data. The multiple imputation method will follow published guidelines.[46] PROC MI in SAS will be used to generate 50 complete data sets. The imputation model will include all variables included in the primary outcome analysis model. The overall summary adjusted mean difference will be presented with 95 % confidence intervals, to assess the sensitivity of the primary analysis to missing data. All analyses will be reported in accordance with the Consolidated Standards of Reporting Trials Checklist (CONSORT)[47] and regardless of statistical significance.

Health economic evaluation

The economic analysis will be performed in accordance with a Health Economics Analysis Plan, and by the trial health economists at Bangor University. The primary analysis will adopt an NHS and Personal Social Services perspective and, based on Quality-Adjusted Life Years (QALYs) as a measure of health outcome, estimate the incremental cost-effectiveness ratio from an incremental analysis of the mean costs and QALYs for the intervention and control trial arms.[48] Data assumed to be missing at random will be imputed using multiple imputation by chained equations.[49]

Sensitivity analyses will be conducted to test whether, and to what extent, the incremental cost effectiveness ratio is sensitive to key assumptions in the analysis (e.g. unit prices, different utility estimates from CHU-9D[38] vs. EQ-5D-Y[37]). The joint uncertainty in costs and QALYs will be addressed through application of bootstrapping and estimation of cost-effectiveness acceptability curves.[50] Alternative scenarios considering a broader cost perspective (including indirect costs, such as school absences and loss of productivity, valued by reference to published sources), and a range of outcomes (including parental QALYs, measured using the EQ-5D-5L[51] and ISI[30 40]) will be conducted. Inclusion of spillover disutility[52] (impact on parents' utility) will be based on the NICE reference case specification[53] that all QALYs are of equal weight and calculated assuming additive effects. Health-economic findings will be reported according to the Consolidated Health Economic Evaluation Reporting Standards (CHEERS).[54]

Qualitative component

Child and parent interviews will be analysed by the Qualitative Research Team using an interpretive, reflexive, and conceptual analytical approach. Audio-recordings of interviews will be transcribed and thematically analysed in discrete sets (e.g. intervention/control, child/parent, engagement/lack of engagement with intervention, types of decision-making, different responses/experiences). Parent and child transcripts will first be analysed separately, and then as dyads. All data will be used for synthesis. Thematic and content analyses will be used for child activity booklets (text and images). Qualitative and selected quantitative data (e.g. anxiety measures, actigraphy data) will be compared, as appropriate.

For peer review only

Table 5. Analysis plan for outcome variables in CASTLE Sleep-E. Further analyses details are reported in-text.

Outcome type	Specific measurement variable	Hypothesis	Method of analysis
Primary			
Clinical	Children's Sleep Habits Questionnaire[28]	Total score lower in intervention arm at 3 months	Linear mixed effect regression:<br>• Fixed effects: Intervention (binary)<br>• Random effects: Trial site (categorical)<br>• Co-variates:<br>○ Baseline score<br>○ Use of sleep medication (binary)
Health economic	Cost[a] per quality-adjusted life year gained	Not applicable (health economic evaluation)	Cost-effectiveness (utility) analysis
Secondary			
Clinical	Children's Sleep Habits Questionnaire[28]	Total score lower in intervention arm at 6 months	Linear mixed effect regression (as before)
Clinical	Seizure-free period	Time to first seizure (days) differs between trial arms at 3 and 6 months	Survival analyses<br>• Kaplan-Meier curves by trial arm<br>• Cox proportional hazards regression (if applicable)<br>○ Co-variates:<br>▪ Use of sleep medication (binary)<br>▪ Trial site (categorical)
Clinical	Time to 6-months seizure remission from randomisation (days)	Time to 6-months seizure remission (days) differs between trial arms at 6 months	Survival analyses (as before)
Clinical	• Knowledge about Sleep in Childhood<br>• Actigraphy[36] (2-week average):<br>○ Total sleep time<br>○ Sleep latency<br>○ Sleep efficiency	Total score differs between trial arms at 3 months	Linear mixed effect regression (as before)
Clinical	• Hospital Anxiety and Depression Scale[29]<br>• Insomnia severity index[30]	Total score lower in intervention arm at 3 and 6 months	Linear mixed effect regression (as before)
Clinical	• Sickness-related school absences	Total days differs between trial arms at 3 and 6 months	Poisson mixed-effects regression

[a] **Perspective**: NHS and PSS perspective; **Alternative perspective**: Societal (Indirect and direct non-medical costs)

For peer review only

Outcome type	Specific measurement variable	Hypothesis	Method of analysis
Clinical	• Health-Related Quality Of Life Measure for Children with Epilepsy[32]<br>• World Health Organisation – Five Well-Being Index[33]	Total score differs between trial arms at 6 months	Linear mixed effect regression (as before)
Clinical	• SleepSuite[31]: Animal task<br>• SleepSuite: Bubble task<br>○ Shape detection<br>○ Emotion detection<br>○ Gender detection<br>• SleepSuite: Maze task	Executive function, reaction time, and variability differ between trials arm at 3 months	• Poisson/zero-inflated negative binomial regression (depending on presence of overdispersion)<br>• 2 x 2 multi-variate repeated-measures Analysis of Variance (ANOVA)<br>Factors: Time (PM/AM) x Intervention (Pre/Post)<br>Fitted per detection task (Shape, Emotion, Gender)<br>• Linear mixed effect regression (as before)
Clinical	• Strengths and Difficulties[34] Questionnaire<br>• Parenting Self Agency Measure[35]	Total score differs between trial arms at 3 and 6 months	Linear mixed effect regression (as before)
Qualitative	Trial experience[b]	Not applicable (inductive)	Thematic analysis (interpretive, reflexive, and conceptual analytical approach)<br>• Discrete sets: Intervention/Control, Child/parent, Engagement with intervention/lack thereof, Decision making types, Responses/experiences<br>• Separately for child and parent, then jointly (dyad)<br>• Comparisons to selective objective data as emerging from analysis (e.g. Anxiety measures, Actigraphy)

[b] **Source data for trial experience**: Qualitative interviews (parents and children individually and as dyad), activity booklets (children only)

For peer review only - http://bmjopen.bmj.com/site/about/guidelines.xhtml

Harms

A flowchart of Adverse Event (AE) reporting requirements is shown in Supplemental Figure 1. Harms severity and causality will be graded by the investigator responsible for the care of the participant based on categories shown in Supplemental Table 5. If any doubt about causality exists, the local investigator should inform LCTC who will notify the CI. In case of discrepant views, the Research Ethics Committee (REC) will be informed of both views. Seriousness and expectedness of AEs will be defined based on International Council for Harmonisation of Technical Requirements for Pharmaceuticals for Human Use Definitions and Standards for Expedited Reporting (ICH E2A, ref: CPMP/ICH/377/95). Expectedness will be assessed by the CI. The only expected AEs in CASTLE Sleep-E are mild and transient worsening of sleep behaviours targeted by the trial intervention. Safety data will be quality-checked by a statistician not otherwise involved in the trial. Safety analysis will include all patients randomised and starting treatment and be presented descriptively split by treatment arm.

Auditing

The CI will ensure that the trial team conducts monitoring activities of sufficient quality and quantity (e.g. protocol adherence, consent/assent, data quality). The Sponsor will delegate monitoring duties and activities to LCTC. The CI and LCTC will inform the Sponsor of any concerns. Auditing does not meet the National Institute for Health and Care Research (NIHR) or SPIRIT Statement definitions of independence[19 55] as auditors (LCTC and CI) are part of the trial team.

Protocol amendments

Substantive protocol amendments will be notified to HRA via the UK's Integrated Research Application System (IRAS). Trial sites will receive an amendment pack of HRA- and REC-approved changes and unless an objection is received within 35 days, the trial will continue at site with a GO LIVE email.

Ancillary and post-trial care

King's College London (KCL) holds insurance against claims from participants for harm caused by their participation in this clinical study; compensation can be claimed in case of KCL negligence.

Ethics and dissemination

The CASTLE Sleep-E protocol was approved by the HRA East Midlands – Nottingham 1 REC, reference: 21/EM/0205. Trial results will be disseminated to scientific audiences in peer-reviewed publications and conferences, and — with the help of the CASTLE Advisory Panel (parent and child experts-by-experience), relevant charities (e.g. Epilepsy Action, Epilepsy Society and Cerebra) and professional groups (e.g. Royal College of Paediatrics and Child Health, Epilepsy Specialist Nurses Association) — as plain language summaries to families, other professional groups, managers, commissioners, and policy makers. Pseudo-anonymised Individual Patient Data and associated documentation (e.g. protocol, statistical analysis plan, annotated blank Case Report Form) will be made available after dissemination on reasonable request.

For peer review only - http://bmjopen.bmj.com/site/about/guidelines.xhtml

Registration details

ISRCTN registry (Trial ID: ISRCTN13202325, prospective registration 09/September/2021). The World Health Organisation Trial Registration Data Set (Version 1.3.1) for CASTLE Sleep-E is shown in Supplemental Table 1.

For peer review only

Author Statement

Contributorship (alphabetic surname order)
PG, DKP (Chief Investigators); CAP, CTS, HH, LW, BC, CM, DH, and LB (Co-Investigators) conceived the study and are award holders. Topic expertise for the core outcome set development was provided by CAP, LB, BC, AC, HC, PG, DH, CM, DKP, CTS, and PRW. Epilepsy expertise-by-experience is provided by CAP. Topic expertise for epilepsy is provided by DKP. Topic expertise for the health economic evaluation is provided by WASH, DH, and EW. Topic expertise for intervention development was provided by GC, PG, HH, DKP, and LW. Topic expertise for Patient and Public Involvement (Advisory Panel and Family Engagement) is provided by CAP, AR-S, LB, BC, and CM. Responsibility for the selection of Patient-Reported Outcomes lay with CM. Responsibility for Programme management lies with AC. Topic expertise for qualitative research components is provided by CAP, LB, BC, and HS. Topic expertise for sleep is provided by GC, PG, HH, and LW. Topic expertise for Statistical analyses is provided by CTS, VW, and LWh. Responsibility for trial management lies with NA-N, CS, and LS-E. All authors contributed to the design and refinement of the study protocol. The protocol manuscript was written by KCD (including supplemental materials but excluding Figure 1 and Patient Information Sheet and Consent Forms). Authors in the Trial Management Group (TMG) had the opportunity to provide feedback twice (initial and final draft); non-TMG authors had the opportunity to provide feedback once (final draft). Provided feedback was incorporated. The final manuscript was approved for publication by all authors. GRIPP2 content was checked for accuracy by LB. Sponsor name and contact information are provided in Supplemental Table 1. Details of trial committees and other groups and individuals overseeing the trial are listed in Supplemental Table 4. Trial site Principal Investigators will be listed alphabetically in resulting publications as members of the CASTLE Sleep-E Consortium in the Acknowledgements section. There has not been and will not be any use of hired writers.

Funding. This work is supported by the National Institute for Health and Care Research (NIHR), award number RP-PG-0615-20007. HH was supported by a National Health and Medical Research Council (NHMRC, Australia) Practitioner Fellowship (1136222). HH's institute — the Murdoch Children's Research Institute (MCRI, Australia) — is supported by the Victorian Government's Operational Infrastructure Support Program (no award/grant number).

Disclaimer. To avoid potential bias, neither the funder nor the sponsor of this trial has any role in or authority over the design, execution, analyses, interpretation of data, or result dissemination.

Competing interests. None declared.

Patient consent for publication. Not applicable.

Provenance and peer review. Not commissioned, externally peer reviewed.

Supplemental material. This content has been supplied by the author(s). It has not been vetted by BMJ Publishing Group Limited (BMJ) and may not have been peer-reviewed. Any opinions or recommendations discussed are solely those of the author(s) and are not endorsed by BMJ. BMJ disclaims all liability and responsibility arising from any reliance placed on the content. Where the content includes any translated material, BMJ does not warrant the accuracy and reliability

For peer review only - http://bmjopen.bmj.com/site/about/guidelines.xhtml

of the translations (including but not limited to local regulations, clinical guidelines, terminology, drug names and drug dosages), and is not responsible for any error and/or omissions arising from translation and adaptation or otherwise.

Licence statement. I, the Submitting Author has the right to grant and does grant on behalf of all authors of the Work (as defined in the below author licence), an exclusive licence and/or a non-exclusive licence for contributions from authors who are: i) UK Crown employees; ii) where BMJ has agreed a CC-BY licence shall apply, and/or iii) in accordance with the terms applicable for US Federal Government officers or employees acting as part of their official duties; on a worldwide, perpetual, irrevocable, royalty-free basis to BMJ Publishing Group Ltd ("BMJ") its licensees and where the relevant Journal is co-owned by BMJ to the co-owners of the Journal, to publish the Work in BMJ Open and any other BMJ products and to exploit all rights, as set out in our licence.

The Submitting Author accepts and understands that any supply made under these terms is made by BMJ to the Submitting Author unless you are acting as an employee on behalf of your employer or a postgraduate student of an affiliated institution which is paying any applicable article publishing charge ("APC") for Open Access articles. Where the Submitting Author wishes to make the Work available on an Open Access basis (and intends to pay the relevant APC), the terms of reuse of such Open Access shall be governed by a Creative Commons licence – details of these licences and which Creative Commons licence will apply to this Work are set out in our licence referred to above.

For peer review only

References

1. Collaborators GBDE. Global, regional, and national burden of epilepsy, 1990-2016: a systematic analysis for the Global Burden of Disease Study 2016. Lancet Neurol 2019;**18**(4):357-75 doi: 10.1016/S1474-4422(18)30454-X [published Online First: 20190214].
2. Olusanya BO, Wright SM, Nair MKC, et al. Global Burden of Childhood Epilepsy, Intellectual Disability, and Sensory Impairments. Pediatrics 2020;**146**(1) doi: 10.1542/peds.2019-2623.
3. Li Q, Westover MB, Zhang R, et al. Computational Evidence for a Competitive Thalamocortical Model of Spikes and Spindle Activity in Rolandic Epilepsy. Front Comput Neurosci 2021;**15**:680549 doi: 10.3389/fncom.2021.680549 [published Online First: 20210618].
4. Stephen J, Weir CJ, Chin RF. Temporal trends in incidence of Rolandic epilepsy, prevalence of comorbidities and prescribing trends: birth cohort study. Arch Dis Child 2020;**105**(6):569-74 doi: 10.1136/archdischild-2019-318212 [published Online First: 20200114].
5. Ross EE, Stoyell SM, Kramer MA, et al. The natural history of seizures and neuropsychiatric symptoms in childhood epilepsy with centrotemporal spikes (CECTS). Epilepsy Behav 2020;**103**(Pt A):106437 doi: 10.1016/j.yebeh.2019.07.038 [published Online First: 20191020].
6. Petropoulos M-C, Bonaiuto K, Currier J, et al. Practical aspects of childhood epilepsy. BMJ 2019;**367**:l6096 doi: 10.1136/bmj.l6096.
7. Larson AM, Ryther RC, Jennesson M, et al. Impact of pediatric epilepsy on sleep patterns and behaviors in children and parents. Epilepsia 2012;**53**(7):1162-9 doi: 10.1111/j.1528-1167.2012.03515.x [published Online First: 20120517].
8. Crudgington H, Rogers M, Bray L, et al. Core Health Outcomes in Childhood Epilepsy (CHOICE): Development of a core outcome set using systematic review methods and a Delphi survey consensus. Epilepsia 2019;**60**(5):857-71 doi: 10.1111/epi.14735 [published Online First: 20190425].
9. Gibbon FM, Maccormac E, Gringras P. Sleep and epilepsy: unfortunate bedfellows. Archives of Disease in Childhood 2019;**104**(2):189-92 doi: 10.1136/archdischild-2017-313421.
10. Phillips NL, Moore T, Teng A, et al. Behavioral interventions for sleep disturbances in children with neurological and neurodevelopmental disorders: a systematic review and meta-analysis of randomized controlled trials. Sleep 2020;**43**(9) doi: 10.1093/sleep/zsaa040.
11. Tsai S-Y, Lee W-T, Lee C-C, et al. Behavioral-educational sleep interventions for pediatric epilepsy: a randomized controlled trial. Sleep 2020;**43**(1):zsz211 doi: 10.1093/sleep/zsz211.
12. Ahorsu DK, Lin CY, Imani V, et al. Testing an app-based intervention to improve insomnia in patients with epilepsy: A randomized controlled trial. Epilepsy Behav 2020;**112**:107371 doi: 10.1016/j.yebeh.2020.107371 [published Online First: 20200827].
13. Condon HE, Maurer LF, Kyle SD. Reporting of adverse events in cognitive behavioural therapy for insomnia: A systematic examination of randomised controlled trials. Sleep Medicine Reviews 2021;**56**:101412 doi: https://doi.org/10.1016/j.smrv.2020.101412.
14. Sunnhed R, Hesser H, Andersson G, et al. Comparing internet-delivered cognitive therapy and behavior therapy with telephone support for insomnia disorder: a randomized controlled trial. Sleep 2019;**43**(2) doi: 10.1093/sleep/zsz245.
15. Wiggs L, Cook G, Hiscock H, et al. Development and Evaluation of the CASTLE Trial Online Sleep Intervention for Parents of Children with Epilepsy. Frontiers in Psychology 2021;**12** doi: 10.3389/fpsyg.2021.679804.
16. Cook G, Gringras P, Hiscock H, et al. A Qualitative Investigation Into What Parents Want From an Online Behavioural Sleep Intervention for Children With Epilepsy. Frontiers in Psychology 2021;**12** doi: 10.3389/fpsyg.2021.628605.
17. Morris C, Dunkley C, Gibbon FM, et al. Core Health Outcomes In Childhood Epilepsy (CHOICE): protocol for the selection of a core outcome set. Trials 2017;**18**(1):572 doi: 10.1186/s13063-017-2323-7.
18. Chan A-W, Tetzlaff JM, Altman DG, et al. SPIRIT 2013 Statement: Defining Standard Protocol Items for Clinical Trials. Annals of Internal Medicine 2013;**158**(3):200-07 doi: 10.7326/0003-4819-158-3-201302050-00583.
19. Chan A-W, Tetzlaff JM, Gøtzsche PC, et al. SPIRIT 2013 explanation and elaboration: guidance for protocols of clinical trials. BMJ : British Medical Journal 2013;**346**:e7586 doi: 10.1136/bmj.e7586.
20. Calvert M, King M, Mercieca-Bebber R, et al. SPIRIT-PRO Extension explanation and elaboration: guidelines for inclusion of patient-reported outcomes in protocols of clinical trials. BMJ Open 2021;**11**(6):e045105 doi: 10.1136/bmjopen-2020-045105.

For peer review only - http://bmjopen.bmj.com/site/about/guidelines.xhtml

21. Staniszewska S, Brett J, Simera I, et al. GRIPP2 reporting checklists: tools to improve reporting of patient and public involvement in research. BMJ 2017;**358**:j3453 doi: 10.1136/bmj.j3453.
22. Freedland KE, Mohr DC, Davidson KW, et al. Usual and unusual care: existing practice control groups in randomized controlled trials of behavioral interventions. Psychosom Med 2011;**73**(4):323-35 doi: 10.1097/PSY.0b013e318218e1fb [published Online First: 2011/05/02].
23. Thompson BT, Schoenfeld D. Usual care as the control group in clinical trials of nonpharmacologic interventions. Proc Am Thorac Soc 2007;**4**(7):577-82 doi: 10.1513/pats.200706-072JK.
24. Zuidgeest MGP, Welsing PMJ, van Thiel GJMW, et al. Series: Pragmatic trials and real world evidence: Paper 5. Usual care and real life comparators. Journal of Clinical Epidemiology 2017;**90**:92-98 doi: https://doi.org/10.1016/j.jclinepi.2017.07.001.
25. National Institute for Health and Care Excellence (NICE). Epilepsies: diagnosis and management (CG137). London: NICE, 2012.
26. Scottish Intercollegiate Guidelines Network (SIGN). Epilepsies in children and young people: investigative procedures and management (SIGN 159). Edinburgh: SIGN, 2020.
27. Health and Social Care Board (HSCB) Northern Ireland. Recommended Clinical Guidelines (CGs). Secondary Recommended Clinical Guidelines (CGs) 19/Jan/2022 2022. http://www.hscboard.hscni.net/nice/recommended-clinical-guidelines-cgs/.
28. Owens JA, Spirito A, McGuinn M. The Children's Sleep Habits Questionnaire (CSHQ): psychometric properties of a survey instrument for school-aged children. Sleep 2000;**23**(8):1043-51.
29. Zigmond AS, Snaith RP. The hospital anxiety and depression scale. Acta Psychiatr Scand 1983;**67**(6):361-70 doi: 10.1111/j.1600-0447.1983.tb09716.x.
30. Morin CM, Belleville G, Bélanger L, et al. The Insomnia Severity Index: psychometric indicators to detect insomnia cases and evaluate treatment response. Sleep 2011;**34**(5):601-08 doi: 10.1093/sleep/34.5.601.
31. Colonna A, Smith AB, Smith S, et al. The Effects of Sleep on Emotional Target Detection Performance: A Novel iPad-Based Pediatric Game. Frontiers in psychology 2018;**9**:241-41 doi: 10.3389/fpsyg.2018.00241.
32. Ronen GM, Streiner DL, Rosenbaum P, et al. Health-related quality of life in children with epilepsy: development and validation of self-report and parent proxy measures. Epilepsia 2003;**44**(4):598-612 doi: 10.1046/j.1528-1157.2003.46302.x.
33. Allgaier A-K, Pietsch K, Frühe B, et al. Depression in pediatric care: is the WHO-Five Well-Being Index a valid screening instrument for children and adolescents? General Hospital Psychiatry 2012;**34**(3):234-41 doi: https://doi.org/10.1016/j.genhosppsych.2012.01.007.
34. Goodman R. Psychometric Properties of the Strengths and Difficulties Questionnaire. Journal of the American Academy of Child & Adolescent Psychiatry 2001;**40**(11):1337-45 doi: 10.1097/00004583-200111000-00015.
35. Dumka LE, Stoerzinger HD, Jackson KM, et al. Examination of the Cross-Cultural and Cross-Language Equivalence of the Parenting Self-Agency Measure. Family Relations 1996;**45**(2):216-22 doi: 10.2307/585293.
36. Sadaka Y, Sadeh A, Bradbury L, et al. Validation of actigraphy with continuous video-electroencephalography in children with epilepsy. Sleep Med 2014;**15**(9):1075-81 doi: 10.1016/j.sleep.2014.04.021 [published Online First: 20140602].
37. Wille N, Badia X, Bonsel G, et al. Development of the EQ-5D-Y: a child-friendly version of the EQ-5D. Qual Life Res 2010;**19**(6):875-86 doi: 10.1007/s11136-010-9648-y [published Online First: 20100420].
38. Stevens K. Valuation of the Child Health Utility 9D Index. Pharmacoeconomics 2012;**30**(8):729-47 doi: 10.2165/11599120-000000000-00000.
39. Hernández-Alava M, Pudney S. Mapping between EQ-5D-3L and EQ-5D-5L: A survey experiment on the validity of multi-instrument data. Health Econ 2022;**31**(6):923-39 doi: 10.1002/hec.4487 [published Online First: 20220228].
40. Gu NY, Botteman MF, Ji X, et al. Mapping of the Insomnia Severity Index and other sleep measures to EuroQol EQ-5D health state utilities. Health Qual Life Outcomes 2011;**9**:119 doi: 10.1186/1477-7525-9-119 [published Online First: 20111230].
41. The European Parlament and the Council of the European Union. EU General Data Protection Regulation (GDPR). Secondary EU General Data Protection Regulation (GDPR) 2018. https://eur-lex.europa.eu/legal-content/EN/TXT/?uri=CELEX%3A02016R0679-20160504&qid=1532348683434.
42. The Government of the United Kingdom. UK Data Protection Act 2018. Secondary UK Data Protection Act 2018 2018. https://www.legislation.gov.uk/ukpga/2018/12/contents/enacted.

For peer review only - http://bmjopen.bmj.com/site/about/guidelines.xhtml

43. Williamson PR, Altman DG, Bagley H, et al. The COMET Handbook: version 1.0. Trials 2017;**18**(3):280 doi: 10.1186/s13063-017-1978-4.
44. Kallogjeri D, Spitznagel EL, Jr, Piccirillo JF. Importance of Defining and Interpreting a Clinically Meaningful Difference in Clinical Research. JAMA Otolaryngology–Head & Neck Surgery 2020;**146**(2):101-02 doi: 10.1001/jamaoto.2019.3744.
45. Jaeschke R, Singer J, Guyatt GH. Measurement of health status. Ascertaining the minimal clinically important difference. Control Clin Trials 1989;**10**(4):407-15 doi: 10.1016/0197-2456(89)90005-6.
46. Jakobsen JC, Gluud C, Wetterslev J, et al. When and how should multiple imputation be used for handling missing data in randomised clinical trials – a practical guide with flowcharts. BMC Medical Research Methodology 2017;**17**(1):162 doi: 10.1186/s12874-017-0442-1.
47. Schulz KF, Altman DG, Moher D. CONSORT 2010 statement: Updated guidelines for reporting parallel group randomised trials. J Pharmacol Pharmacother 2010;**1**(2):100-07 doi: 10.4103/0976-500X.72352.
48. Petrou S, Gray A. Economic evaluation alongside randomised controlled trials: design, conduct, analysis, and reporting. BMJ 2011;**342**:d1548 doi: 10.1136/bmj.d1548 [published Online First: 20110407].
49. Manca A, Palmer S. Handling missing data in patient-level cost-effectiveness analysis alongside randomised clinical trials. Applied Health Economics and Health Policy 2005;**4**(2):65-75 doi: 10.2165/00148365-200504020-00001.
50. Fenwick E, Claxton K, Sculpher M. Representing uncertainty: the role of cost-effectiveness acceptability curves. Health Econ 2001;**10**(8):779-87 doi: 10.1002/hec.635.
51. Bergmann M, Tschiderer L, Stefani A, et al. Sleep quality and daytime sleepiness in epilepsy: Systematic review and meta-analysis of 25 studies including 8,196 individuals. Sleep Medicine Reviews 2021;**57**:101466 doi: https://doi.org/10.1016/j.smrv.2021.101466.
52. Wittenberg E, James LP, Prosser LA. Spillover Effects on Caregivers' and Family Members' Utility: A Systematic Review of the Literature. PharmacoEconomics 2019;**37**(4):475-99 doi: 10.1007/s40273-019-00768-7.
53. National Institute for Health and Care Excellence (NICE). NICE health technology evaluations: the manual. Process and methods [PMG36]. London: NICE, 2022.
54. Husereau D, Drummond M, Petrou S, et al. Consolidated Health Economic Evaluation Reporting Standards (CHEERS) statement. BMJ : British Medical Journal 2013;**346**:f1049 doi: 10.1136/bmj.f1049.
55. National Institute for Health and Care Excellence (NICE). Clinical Trials Toolkit: Audit. Audit. London: NICE, 2022.

For peer review only

Supplemental Table 1. World Health Organization Trial Registration Data Set (Version 1.3.1) for CASTLE Sleep-E

Data category	Information
1. Primary registry and trial identifying number	ISRCTN: ISRCTN13202325
2. Date of registration in primary registry	09/September/2021
3. Secondary identifying numbers	CPMS 50413<br>RP-PG-0615-20007<br>IRAS 289580<br>21/EM/0205
4. Source(s) of monetary or material support	National Institute for Health and Care Research (NIHR)
5. Primary sponsor	Ms Jasmine Palmer<br>Research & Innovation Operational Manager<br>King's College Hospital NHS Foundation Trust<br>The Research & Innovation Office<br>First Floor, Coldharbour Works<br>245a Coldharbour Lane, Brixton<br>London SW9 8RR<br>jasmine.palmer1@nhs.net<br>+44 (0) 7790 950 219
6. Secondary sponsor(s)	Professor Reza Razavi<br>Director of Research Management &<br>Director of Administration (Health Schools)<br>Room 5.31<br>James Clerk Maxwell Building<br>57 Waterloo Road<br>London<br>SE1 8WA<br>reza.razavi@kcl.ac.uk<br>+44 (0)20 7848 3224
7. Contact for public queries	Trial Manager: Lucy Stibbs-Eaton<br>Liverpool Clinical Trials Centre<br>University of Liverpool<br>Liverpool<br>L69 3BX<br>LCTC@liverpool.ac.uk<br>+44 (0)151 795 8751
8. Contact for scientific queries	Professor Deb Pal<br>Professor of Paediatric Epilepsy<br>Maurice Wohl Clinical Neuroscience Institute<br>King's College London<br>5 Cutcombe Road<br>London<br>SE5 9RX<br>deb.pal@kcl.ac.uk<br>+44 (0) 207 848 5762
9. Public title	A trial comparing the effectiveness of an online sleep behavioural intervention versus standard care in children with rolandic epilepsy
10. Scientific title	Changing Agendas on Sleep, Treatment and Learning in Epilepsy (CASTLE) Sleep-E: A randomised controlled trial comparing an online behavioural sleep intervention with standard care in children with Rolandic epilepsy

For peer review only - http://bmjopen.bmj.com/site/about/guidelines.xhtml

Data category	Information
11. Countries of recruitment	England<br>Scotland<br>Wales<br>Northern Ireland
12. Health condition(s) or problem(s) studied	Sleep problems in Rolandic epilepsy also known as childhood epilepsy with centro-temporal spikes
13. Intervention(s)	**Intervention arm (SC + COSI)**: Novel, tailored, parent-led CASTLE Online Sleep Intervention (COSI) that incorporates evidence-based behavioural components. Delivered by parents to enrolled children with Rolandic epilepsy in their own homes after completion of self-paced online training. Standard care (SC) is augmented with the CASTLE Online Sleep Intervention (COSI).<br><br>**Active control arm (SC)**: UK National Health Service standard care (SC) for children with Rolandic epilepsy, which consists of a comprehensive care plan with the option of pharmacological treatment with anti-epileptic drugs (first-line mono-therapy with lamotrigine, levetiracetam, oxcarbazepine [girls and boys], carbamazepine or sodium valproate [both boys only]).
14. Key inclusion and exclusion criteria	Inclusion criteria<br>Main CASTLE Sleep-E study<br>1. Children diagnosed with RE/CECTS (see International League Against Epilepsy Diagnostic Manual at https://www.epilepsydiagnosis.org/syndrome/ects-overview.html)<br>2. EEG showing focal sharp waves with normal background (see International League Against Epilepsy Diagnostic Manual at https://www.epilepsydiagnosis.org/syndrome/ects-eeg.html)<br>3. Aged 5 to <13 years at the time of randomisation<br>4. Parent/Carer reported child sleep problem as defined by mild, moderate or severe score on Hiscock Australian global sleep question (Poor sleeper defined by caregiver responding 'Mild', 'Moderate' or 'Severe' to "Over the last 2 weeks, how much of a problem has your child's sleep been?")<br>5. Documented informed consent received from a person with parental responsibility<br>6. Family have an email address and mobile phone<br>7. Parent and child are to have a good enough understanding of the English language to read and answer study questionnaires<br>Qualitative component<br>1. Consent of care giver to participate and for their child to participate (optional item on main trial consent form)<br>2. Children need to be >=7 years of age<br><br>Exclusion criterion<br>1. Children with moderate/severe learning disability
15. Study type	Interventional<br>• Allocation: Minimisation using a bespoke LCTC system<br>Allocation concealment: Central web-interface<br>Sequence generation: Randomised, 1:1 ratio<br>Intervention model: Parallel assignment<br>• Blinding<br>Child, parent, healthcare providers, data collectors, qualitative researchers: None (open label)<br>Quantitative data analysts: Blinded<br>• Primary purpose: Clinical- and cost-effectiveness, process evaluation (qualitative trial component, COSI e-analytics and evaluation module)<br>• Phase: III (behavioural intervention)

For peer review only - http://bmjopen.bmj.com/site/about/guidelines.xhtml

Data category	Information
16. Date of first enrolment	24/June/2022
17. Target sample size	110 (55 children per arm)<br>Calculation based on:<br>• Achieving 90 % statistical power to detect Minimal Clinically Meaningful Difference in primary outcome<br>• 10 % expected attrition
18. Recruitment status	Recruiting<br>• First trial site opened: 12/May/2022<br>• First recruitment: 30/August/2022
19. Primary outcome(s)	• Clinical: Children's Sleep Habits Questionnaire at 3 months<br>• Health economic: Cost-effectiveness of the intervention over 6 months after randomisation, measured in terms of incremental cost per quality-adjusted life year gained (Child Health Utility instrument or EQ-5D-Y) from the perspective of the National Health Services and Personal Social Services in the UK.
20. Key secondary outcome(s)	• Clinical Outcome: Sleep problem reduction<br>Metric/method: Children's Sleep Habits Questionnaire<br>Timepoint: 6 months<br>• Clinical Outcome: Seizure frequency reduction<br>Metric/method: Time to first seizure (days)<br>Timepoint: 3 months, 6 months
21. Ethics Review	• Status: Approved<br>• Approval reference: 21/EM/0205<br>• Health Research Authority<br>East Midlands – Nottingham 1 Research Ethics Committee<br>Chair: Mr Paul Hamilton<br>+44 (0) 207 104 8115 or +44 (0) 207 104 8283<br>nottingham1.rec@hra.nhs.uk
22. Completion date	31/July/2023
23. Summary results	TBC
24. Individual patient data (IPD) sharing statement	• Plan to share IPD: Yes<br>• Plan description: At the end of the trial, after the primary results have been published, the pseudo-anonymised Individual Patient Data and associated documentation (e.g. protocol, statistical analysis plan, annotated blank case report form) will be prepared to be shared with external researchers on reasonable request.
25. Protocol version and date	• Internal protocol: V4.0, 08/December/2021<br>• Manuscript for protocol publication: V3.1, 07/November/2022

For peer review only

For peer review only - http://bmjopen.bmj.com/site/about/guidelines.xhtml

Supplemental Table 2. Psychometrics and clinical relevance/minimal clinically important difference (CR/MCID) for CASTLE Sleep-E outcomes (Table 1). Metrics refer to the single referenced publication. Further validation studies exist, but, due to differences in population, setting, and/or methods, results cannot be merged.

Outcome	Description	Validity	Reliability	CR/MCID
Children's Sleep Habits Questionnaire (CSHQ)[1]	Parent-reported, one-week retrospective sleep screening tool for children (4–10 years)<br><br>35 items (2 duplicated across subscales)<br>3-point Likert scales (rarely, sometimes, usually)<br>Total score (33 items): 33–99, lower is better<br>8 subscales:<br>• Bedtime Resistance (6 items)<br>• Sleep Onset Delay (1 item)<br>• Sleep Duration (3 items)<br>• Sleep Anxiety (4 items)<br>• Night Wakings (3 items)<br>• Parasomnias (7 items)<br>• Sleep-Disordered Breathing (3 items)<br>• Daytime Sleepiness (8 items)<br><br>Validation samples<br>Parents of 469 school children (community setting) and 154 children diagnosed with sleep disorder (hospital setting); English language; England, UK. Test-retest: 60 parents from control sample	Classification accuracy<br>Sleep disorder (yes/no)<br>Receiver Operating Characteristic (ROC) analyses: See MCID<br><br>Construct validity<br>See MCID<br><br>Criterion validity<br>Not assessed	Test-retest<br>2-week delay<br>Pearson's *r:* 0.62–0.79<br><br>Internal consistency<br>Cronbach's α<br>Control sample: 0.68<br>Clinical sample: 0.78<br><br>Inter-rater reliability<br>Not assessed	Cut-off (total score): 41<br>• Sensitivity: 80 %<br>• Specificity: 72 %<br>• Accuracy: 80 %<br><br>MCID<br>Not assessed
EQ-5D-Y[2 3]	Child- or adolescent reported (4–7 years: EQ-5D-Y proxy; 8–16 years: EQ-5D-Y, ≥16 years: EQ-5D-5L), standardised measure of current ('today')<br>• *health profile* across 5 dimensions,<br>• self-rated *health status*, and<br>• *EQ-5D-Y index value,* using a country-specific weighting	Not yet validated in UK (last updated 07/March/2022)	Not yet validated in UK (last updated 07/March/2022)	CR/MCID<br>Applicability to utility scores debated, suggested MCID: difference in index score between baseline health profile and single-level transitions in single domain (e.g. 33333 to 33332).

For peer review only - http://bmjopen.bmj.com/site/about/guidelines.xhtml

Outcome	Description	Validity	Reliability	CR/MCID
	(value set) of a given health profile.<br><br>Two components:<br>1. <u>Descriptive system</u><br>5 dimensions with 3 response severity options each (tick-box):<br>• Mobility<br>• Self-care<br>• Usual activities<br>• Pain/discomfort<br>• Anxiety/depression<br>2. <u>Visual Analogue Scale</u><br>Self-rated health on a vertical Visual Analogue Scale (VAS) that ranges from 'The best health you can imagine' (100) to 'The worst health you can imagine' (0).<br><br>Scoring:<br>• Descriptive system: 5-digit *health profile* (best health state: 11111, indicating no problem in each of the 5 dimensions; worst health state: 33333 indicating many problems in each of the 5 dimensions; 243 possible health states are coded)<br>• VAS: 0–100 subjective *health state* (worst to best)<br>• *EQ-5D-5L index value* Single summary number, calculated by subtracting country-specific weighing (value set) of an obtained health profile from 1, where 1 represents the best possible health profile of 11111.<br><br><u>Value set validation sample (UK)</u><br>Not yet validated in UK (last updated 07/March/2022)			

For peer review only

For peer review only - http://bmjopen.bmj.com/site/about/guidelines.xhtml

Outcome	Description	Validity	Reliability	CR/MCID
Child Health Utility instrument (CHU-9D)[4]	Child-reported (7–11 years) descriptive system for current ('today') generic health-related quality-of-life<br><br>9 dimensions with 5 response severity options each (circle):<br>• Worried<br>• Sad<br>• Pain<br>• Tired<br>• Annoyed<br>• School-/homework<br>• Sleep<br>• Daily routine<br>• Activities<br><br>Scoring:<br>• Descriptive system: 9-digit *health profile* (best health state: 111111111, indicating no problem in each of the 9 dimensions; worst health state: 555555555 indicating many problems in each of the 5 dimensions; 1953125 possible health states are coded)<br>• *CHU-9D index value* Single summary number indicating the utility value of a given health state, established using Standard Gamble (SG) tasks.<br><br><u>Value set validation sample (England)</u><br>1245 households were randomly sampled from a database of UK names and addresses in Sheffield and Huddersfield (England) were contacted by a research team of the Centre for Research and Evaluation (CRE) at Sheffield Hallam	<u>Predictive accuracy</u><br>Standard ordinary least squares (OLS) regression: 98.41 %<br>No systematic bias, no auto-correlated errors.<br><br><u>Construct validity</u><br>Not assessed<br><br><u>Criterion validity</u><br>Not assessed<br><br><u>Face-validity</u><br>Preference elicitation using Standard Gamble (SG) task, which give the choice of living in a specific health-state until death with certainty (Choice A), or taking a gamble (Choice B) that could result in living in perfect health for the rest of life with a probability *p*, or dying with a probability 1-*p*. The utility value of a given health-state is the point of indifference between options A and B.<br>Utility values are consistent with health profiles but required merging of response options.	<u>Test-retest</u><br>Not assessed<br><br><u>Internal consistency</u><br>Utility values are consistent with health profiles, but required merging of the initial 5 response-levels for all but one of the 9 dimensions as follows:<br>• Worried: 2<br>• Sad: 4<br>• Pain: 4<br>• Tired: 2<br>• Annoyed: 2<br>• School-/homework: 2<br>• Sleep: 4<br>• Daily routine: 5<br>• Activities: 3<br><br><u>Inter-rater reliability</u><br>Not assessed	<u>CR/MCID</u><br>Applicability to utility scores debated, suggested MCID: difference in index score between baseline health profile and single-level transitions in single domain (e.g. 555555555 to 555555554).

For peer review only

For peer review only - http://bmjopen.bmj.com/site/about/guidelines.xhtml

Outcome	Description	Validity	Reliability	CR/MCID
	University. 1195 households were approached at the door, of which 661 (55 %) were in, and 300 (25 %) agreed to take part. 282 respondents (all adults) were analysed (94 %). Compared to the general UK population, this adult sample was broadly representative, but more affluent and highly restricted geographically. Modelling did not include key demographic characteristics (e.g. age, gender, education, employment, religion and ethnicity). The sample consisted exclusively of adults but was used to derive a paediatric value set.			
EQ-5D-5L[5]	Adolescent or adult-reported (≥16 years), standardised measure of current ('today'):<br>• *health profile* across 5 dimensions,<br>• subjective *health status*, and<br>• *EQ-5D-5L index value,* using a country-specific weighting (value set) of an obtained health profile.<br><br>Two components:<br>1. Descriptive system<br>5 dimensions with 5 response severity options each (tick-box):<br>• Mobility<br>• Self-care<br>• Usual activities<br>• Pain/discomfort<br>• Anxiety/depression<br>2. Visual Analogue Scale<br>Self-rated health on a vertical Visual Analogue Scale (VAS) that ranges from 'The best health	Classification accuracy<br>Not assessed<br><br>Construct validity<br>Not assessed<br><br>Criterion validity<br>Not assessed<br><br>Face-validity<br>Preference elicitation using time trade-off (TTO) and discrete choice experiments (DCEs).<br>• TTOs: Confirmation of negative relationship between level sum score and average observed value.<br>• DCEs: Confirmation of assumption that health states with lower-level sum	Test-retest<br>Not assessed<br><br>Internal consistency<br>Not assessed<br><br>Inter-rater reliability<br>Not assessed	CR/MCID<br>Applicability to utility scores debated, suggested MCID: difference in index score between baseline health profile and single-level transitions in single domain (e.g. 55555 to 55554).

For peer review only - http://bmjopen.bmj.com/site/about/guidelines.xhtml

Outcome	Description	Validity	Reliability	CR/MCID
	you can imagine' (100) to 'The worst health you can imagine' (0).<br><br>Scoring:<br>• Descriptive system: 5-digit *health profile* (best health state: 11111, indicating no problem in each of the 5 dimensions; worst health state: 55555 indicating many problems in each of the 5 dimensions; 3125 possible health states are coded)<br>• VAS: 0–100 subjective *health state* (worst to best)<br>• *EQ-5D-5L index value* Single summary number, calculated by subtracting country-specific weighing (value set) of an obtained health profile from 1, where 1 represents the best possible health profile of 11111.<br><br><u>Value set validation sample (England)</u><br>2220 households from 66 post-code based primary sampling units in England were contacted by the market research company Ipsos MORI. 2088 participants were invited, of which 996 (47.7 %) completed the valuation questionnaire. Only complete responses were analysed (985 participants, 98.9 %). Compared to the general population of England, the sample included more people aged over 75 years, retired, and with health problems, but fewer younger	scores are more likely to be chosen.		

For peer review only

For peer review only - http://bmjopen.bmj.com/site/about/guidelines.xhtml

Outcome	Description	Validity	Reliability	CR/MCID
	participants, and fewer males.			
Knowledge About Sleep in Childhood (KASC, custom-scale devised for CASTLE Sleep-E)	13 items<br>Self-reported Likert-scales assessing parental efficacy in managing child sleep and knowledge about child sleep	Not evaluated	Not evaluated	Not evaluated
Hospital Anxiety and Depression Scale (HADS)[6]	Self-reported, one-week retrospective screening tool for anxiety and depression in people aged 16–65.<br><br>14 items<br>5-point Likert scales (0–3)<br>No total score<br>Subscale score: 0–21, lower is better<br>2 subscales (7 items each):<br>• Depression<br>• Anxiety<br><br>Validation samples<br>2 x 50 patients (16–65 years) with and without psychiatric disorders (hospital setting); English language; England, UK.	Classification accuracy<br>Psychiatric interview,<br>see CR/MCID<br><br>Construct validity<br>See CR/MCID<br><br>Convergent validity<br>Spearman's ρ<br>Interview/self-rating<br>Depression/Depression: 0.79<br>Anxiety/Anxiety: 0.54<br><br>Discriminant validity<br>Spearman's ρ<br>Interview/self-rating<br>Depression/Anxiety ns<br>Anxiety/Depression ns<br><br>Criterion validity<br>See CR/MCID	Test-retest<br>Not assessed<br><br>Internal consistency<br>Spearman's ρ<br>Anxiety: 0.41–0.76<br>Depression: 0.30–0.60<br><br>Inter-rater reliability<br>Not assessed	Cut-offs (subscales)<br>Depression<br>Absent:≤ 7<br>Borderline: 8–10<br>Definite: ≥ 11<br>• False positives: 1 %<br>• False negatives: 1 %<br>Borderline not counted as error<br><br>Anxiety<br>Absent:≤ 7<br>Doubtful: 8–10<br>Definite: ≥ 11<br>• False positives: 5 %<br>• False negatives: 1 %<br>Borderline not counted as error<br><br>MCID<br>Not assessed

For peer review only - http://bmjopen.bmj.com/site/about/guidelines.xhtml

Outcome	Description	Validity	Reliability	CR/MCID
Insomnia Severity Index (ISI)[7], patient version	Self-reported, one-month retrospective screening tool for insomnia in adults (≥18 years)<br>7 items<br>5-point Likert scales (0–4, no problem to severe problem)<br>Total score: 0–28, lower is better<br>• 0–7: Absence of insomnia<br>• 8–14: Subthreshold insomnia<br>• 15–21: Moderate insomnia<br>• 22–28: Severe insomnia<br>Dimensions:<br>• Severity of sleep onset<br>Sleep maintenance<br>• Early morning awakening problems<br>• Sleep dissatisfaction<br>• Interference of sleep difficulties with daytime functioning<br>• Noticeability of sleep problems by others<br>• Distress caused by the sleep difficulties<br><br>Validation samples<br>959 adults with and without insomnia (community setting), 183 adults with insomnia and 62 controls (clinical setting); English language; Québec, Canada.	Classification accuracy<br>Insomnia (yes/no)<br>ROC analyses, see MCID<br><br>Construct validity<br>See CR/MCID<br>Pearson's *r*<br>• Daily sleep diary: 0.54–0.59<br>• Activity level, Anxiety (state, trait), Depression, Fatigue (general, physical, mental), Motivation: 0.20–0.48<br><br>Criterion validity<br>Pearson's *r*<br>Polysomnography<br>• Sleep onset latency: ns<br>• Wake after sleep onset: ns<br>• Number of awakenings: ns<br>• Early morning awakening: ns<br>• Total wake time: ns<br>• Sleep efficiency: -0.16	Test-retest<br>Not assessed<br><br>Internal consistency<br>Cronbach's α, Control sample: 0.71<br>Clinical sample: 0.73<br><br>Inter-rater reliability<br>Not assessed	Control sample (self-diagnosis)<br>Cut-off (total score): 10<br>• Sensitivity: 86 %<br>• Specificity: 88 %<br>• Accuracy: 87 %<br><br>Clinical sample<br>Cut-off (total score): 11<br>• Sensitivity: 97 %<br>• Specificity: 100%<br>Accuracy: 98 %<br><br>MCID<br>Change required for improvement<br>Blinded assessor, *M*, [CI_{95}]:<br>• Slight: 4.65 [2.61–6.69]<br>• Moderate: 8.36 [7.20–9.53]<br>• Marked: 9.89 [8.74–11.04]<br>ROC analyses:<br>• Slight: not reported<br>• Moderate: ≥7<br>○ Sensitivity: 60 %<br>○ Specificity: 70 %<br>○ Accuracy: not reported<br>• Marked: ≥8<br>○ Sensitivity: 64 %<br>○ Specificity: 80 %<br>○ Accuracy: not reported

For peer review only

For peer review only - http://bmjopen.bmj.com/site/about/guidelines.xhtml

Outcome	Description	Validity	Reliability	CR/MCID
SleepSuite[8] (iPad App): Bubble task<br><br>• Executive function (accuracy and response times [RT])	SleepSuite bubble tasks (iPad games) are adapted from a validated Balloon Task[9]: The goal is to burst upward drifting balloons with children's faces under multiple target conditions (e.g. happy faces only) and at increasing presentation conditions (speed, load: number of faces shown simultaneously).<br><br>Validation sample[9]<br>134 healthy children (7–12 years, 58 boys, 23 with clinical behavioural problems, 40% first-born) from middle- and upper-class families of which 25% included at least one parent who immigrated more than 10 years ago. Children lived with their parents in small households (on average 4.53 members). Parents were largely employed full-time (fathers: 90.71%, mothers: 49.31%) and well educated (on average for 16 years). Community setting (school, number unspecified); paid participation ($15 school supply voucher); language: Hebrew, Israel.	Classification accuracy<br>Not assessed<br><br>Construct validity<br>Not assessed<br><br>Criterion validity<br>Child Behavior Checklist (CBCL): total score, sub-scales (8), recode to externalising and internalising behaviours.<br><br>Pearson's *r* (age and sex partialled out), across conditions<br><br>**Completed levels/RT**<br>• Total score: -0.24/ns<br>• Delinquency: ns/0.18<br>• Aggression: -0.20/0.23<br>• Attention problems: -0.18/ns<br>• Social withdrawal: -0.24/ns<br>• Somatic complaints: ns/0.18<br>• Thought disorders: ns/ns<br>• Anxiety-Depression: -.28/ns<br>• Social problems: -0.20/ns<br>• Externalising behaviours: -0.18/0.23<br>• Internalising behaviours: -0.25/ns	Test-retest<br>Delay unspecified (likely none [immediate retest])<br><br>Pearson's *r*<br>• Hits: 0.60<br>• Misses: 0.37<br>• Completed levels: 0.39<br>• RT: 0.78<br><br>Internal consistency<br>Not assessed<br><br>Inter-rater reliability<br>Not assessed	Not assessed

For peer review only - http://bmjopen.bmj.com/site/about/guidelines.xhtml

Outcome	Description	Validity	Reliability	CR/MCID
Health-Related **Q**uality **O**f **L**ife Measure for **Ch**ildren with **E**pilepsy (CHEQOL)[10]	Quality of life assessment tool for children or parents with epilepsy (no specified time-period); child reported if ≥8 years, parent proxy-report if child 5 to <8 years<br>25 items<br>4-point Likert scales (0–4, opposites: true/sort of true)<br>Total score: 25–100, higher is better<br>5 subscales (5 items each):<br>• Interpersonal/social consequences<br>• Future worries<br>• Present worries<br>• Intrapersonal/emotional<br>• Epilepsy secrecy<br><br><u>Validation samples</u><br>381 children (6–15 years) with epilepsy and their parents (clinical setting); English language; Ontario, Canada. Test-retest: Additional 89, then 31 children; additional 48 parents.<br>Metrics refer to self-report for children 8–15 years and parent proxy report for children 5 to <8 years and were assessed for sub-scales, not total score.	<u>Classification accuracy</u><br>Not assessed<br><br><u>Construct validity (child)</u><br>Pearson's *r*<br>• Health care utilisation: 0.13–0.31<br>• Drug Adverse Events: 0.18–0.25<br>• Number of friends: 0.18<br>• Nº of extracurricular activities: 0.13<br>One-way ANOVA ($p \leq .05$)<br>• Seizure severity: All 5 subscales<br>• Anti-epileptic drug use: 4 subscales<br>*t–tests* ($p \leq .05$)<br>• Help at school: All 5 subscales<br>Results for parent-proxy similar<br><br><u>Criterion validity</u><br>Not assessed	<u>Test-retest</u><br>10– 14 days delay<br>Intraclass correlation coefficient<br>Child: 0.59–0.69<br>Parent: 0.60–0.81<br><br>*<u>Internal consistency</u>*<br>Cronbach's *α,* subscales<br>*Child: 0.63–*0.84<br>*Parent: 0.64–*0.86<br><br><u>Inter-rater reliability</u><br>Pearson's *r*<br>• Child/mother: 0.24–0.56<br>• Child/father: 0.18–0.54<br>• Mother/father: 0.40–0.71	Not assessed

For peer review only

For peer review only - http://bmjopen.bmj.com/site/about/guidelines.xhtml

Outcome	Description	Validity	Reliability	CR/MCID
World Health Organisation – Five Well-Being Index (WHO-5)[11]	Self-reported, two-week retrospective tool to assess subjective psychological well-being in people aged 9 years and older.<br><br>5 items<br>6-point Likert scales (0–5, 'at no time' to 'all the time')<br>Raw score: 0–25<br>Total score multiplied by 4 to give final score: 0–100, higher is better<br><br>Validation samples<br>446 children analysed (9–12 years, 16 [3.6 %] with depressive disorder), 6 additional participants dropped due to incomplete data.<br>Hospital setting: 3 paediatric hospitals and 3 paediatric surgery hospitals (in- and out-patients for non-psychiatric reasons), Munich, Germany.<br>German language.	Classification accuracy<br>Depressive disorder (yes/no)<br>Receiver Operating Characteristic (ROC) analyses: See CR/MCID<br><br>Construct validity<br>See CR/MCID<br><br>Criterion validity<br>Diagnostic and Statistical Manual of Mental Disorders (DSM-IV) criteria for depressive disorder (major or minor depression only, dysthymia dropped due to mismatch in time-period of concept definitions), see CR/MCID.	Test-retest<br>Not assessed<br><br>Internal consistency<br>Not assessed<br><br>Inter-rater reliability<br>Cohen's k = .90	Cut-off (total score): 10<br>• Sensitivity: 75 %<br>• Specificity: 92 %<br>• Accuracy: 88 %<br><br>MCID<br>Not assessed

For peer review only

For peer review only - http://bmjopen.bmj.com/site/about/guidelines.xhtml

Outcome	Description	Validity	Reliability	CR/MCID
Strengths and Difficulties Questionnaire (SDQ)[12]	Parent-, teacher-, or child-reported, retrospective screening tool of child psychopathology (2–18 years). Retrospective period: 6 months or current school year<br><br>25 items<br>3-point Likert scales (0–2, not/somewhat/certainly true)<br>Total score: 0–40, lower is better<br>5 subscales (5 items each):<br>• hyperactivity/inattention,<br>• emotional problems<br>• conduct problems<br>• peer problems<br>• prosocial behaviours (omitted from total score)<br><br>Validation samples<br>541 children (5–12 years) with and without psychiatric disorders (school setting); multiple languages; Italy, Germany, the Netherlands, Lithuania, Bulgaria, Romania, and Turkey. Metrics refer to parent-report, total score, and data aggregated across countries and psychiatric disorders.	Classification accuracy<br>Psychiatric disorder (yes/no)<br>Receiver Operating Characteristic (ROC) analyses: See CR/MCID<br>Original total score cut-offs:<br>• Normal: 0–13<br>• Borderline: 14–16<br>• Abnormal: 17–40<br>transformed to binary:<br>• No: 0–16<br>• Yes: 17–40<br><br>Construct validity<br>See CR/MCID<br><br>Criterion validity<br>Diagnostic and Statistical Manual of Mental Disorders (DSM-IV), see CR/MCID.	Test-retest<br>Not assessed<br><br>Internal consistency<br>Cronbach's α: 0.84<br><br>Inter-rater reliability<br>Not assessed.	Cut-off (total score): 17<br>• Sensitivity: 88 %<br>• Specificity: 59 %<br>• Accuracy: 74 %<br><br>MCID<br>Not assessed

For peer review only - http://bmjopen.bmj.com/site/about/guidelines.xhtml

Outcome	Description	Validity	Reliability	CR/MCID
Parenting Self Agency Measure (PSAM)[13]	Self-reported tool assessing overall confidence to successfully parent (including managing the child's behaviour and resolving problems with the child). The time-period for parental self-assessment is unspecified.<br><br>5 items<br>7-point Likert scales (1–7, rarely to always)<br>Total score: 5–35, higher is better<br><br><u>Validation sample</u><br>90 English-speaking mothers (all European-American, median age 36–40 years, median annual income >$40,000, median education bachelor's degree, 82% married or co-habiting) of 3–12-year-olds (community setting); 2 day-care centres and classes at a large university, 2 churches. English language, southwestern USA.	<u>Classification accuracy</u><br>Not assessed<br><br><u>Construct validity</u><br>Convergent validity<br>Pearson's *r*<br>Active coping: 0.31<br>Parenting acceptance: 0.55<br>Positive re-interpretation: ns<br><br>Discriminant validity<br>Pearson's *r*<br>Inconsistent parental disciplining: -0.34<br>Acceptance coping: ns<br><br><u>Criterion validity</u><br>Not assessed	<u>Test-retest</u><br>Not assessed<br><br><u>Internal consistency</u><br>Cronbach's α: 0.70<br>Comparative Fit Index: 0.94<br><br><u>Inter-rater reliability</u><br>Not assessed	Not assessed

For peer review only

Outcome	Description	Validity	Reliability	CR/MCID
Actigraphy: Micro Motionlogger® Watch, Watchware Software V 1.99.17.4, Action-W software, V 2.7.3285 (Ambulatory Monitoring, Inc., NY: USA) combined with sleep diaries (Child and Parent)<br><br>• Total sleep time (minutes)<br>• Sleep latency (minutes)<br>• Sleep efficiency (% asleep of sleep period)<br><br>All 2-week averages	The Micro-Motionlogger® Watch directly measures 3-D acceleration (in CASTLE Sleep-E and the referenced validation study of the non-dominant wrist). Raw data (zero-crossing mode) is initially recorded as periods of activity and inactivity (1 min epochs), and then recoded into periods of wakefulness and sleep using a combination of proprietary algorithms and manual processing (e.g. sleep periods are visually inspected and manually corrected with the aid of participant sleep diaries). Sleep- and wake parameters are then calculated automatically using validated public algorithms.<br><br><u>Validation sample[9]</u><br>27 children (3–17 years) with medically refractory epilepsy, of which 12 had parent-indicated sleep problems (44%). Hospital setting (in-patient epilepsy monitoring unit in tertiary paediatric hospital), English language, Toronto, Canada.	<u>Classification accuracy</u><br>Not assessed<br><br><u>Construct validity</u><br>Not assessed<br><br><u>Criterion validity</u><br>Agreement of actigraphy with continuous video-electroencephalography (24 hours), scored by neurologist and neurophysiologist.<br><br>Bland-Altman plots in combination with *t*-tests for significant bias:<br>• Total sleep time (minutes): Bias = 8.3 (SD = 31), n.s.<br>• Wake duration: Bias = -4.8 (SD = 31.1), n.s.<br><br>Pearson's *r*:<br>• Total sleep time (minutes): 0.96<br>• Wake duration: 0.93	<u>Test-retest</u><br>Not assessed<br><br><u>Internal consistency</u><br>Not assessed<br><br><u>Inter-rater reliability</u><br>Not assessed	Not assessed

For peer review only

For peer review only - http://bmjopen.bmj.com/site/about/guidelines.xhtml

Table 3. Estimated overall time requirement for CASTLE Sleep-E (participant perspective). Time estimates for questionnaires/instruments are based on published estimates where available, and otherwise on an estimate (indicated by *) of 30 seconds per item derived from the Children's Sleep Habits Questionnaire (35 items, 10 minutes published completion time), plus an arbitrary estimate of 2 minutes to read instructions and consider responses. The total time requirement for participation in CASTLE Sleep-E varies from minimally 2 hours per month over a 6-month period in the Standard Care arm omitting optional qualitative interviews to maximally 3 hours per month over a 6-month period in the intervention arm including optional qualitative interviews.

Trial component	Time (mins)	Frequency	Overall time (mins)
Study visits (4)<br>Remote or in-person, combinable with standard care visits<br>• Consent and baseline data<br>• Randomisation<br>• Follow-up at 3 months<br>• Follow-up at 6 months	 <br>• 60 minutes<br>• 30 minutes<br>• 30 minutes<br>• 30 minutes	 <br>• 1<br>• 1<br>• 1<br>• 1	150 minutes
Questionnaires/instruments in order of the participant timeline shown in Table 4<br>• Children's Sleep Habits Questionnaire[1], 35 items<br>• World Health Organisation – Five Well-Being Index[11], 5 items<br>• Health-Related Quality Of Life Measure for Children with Epilepsy[10], 25 items<br>• Strengths and Difficulties Questionnaire[12], 25 items<br>• Child Health Utility Index 9D (CHU-9D)/CHU-9D proxy[4], 9 items<br>• EQ-5D-Y/EQ-5D-Y proxy[2], 15 items<br>• EQ-5D-5L[5], 25 items (note: Published time estimate same as for EQ-5D-Y [15 items])<br>• Parenting Self Agency Measure[13], 5 items<br>• Insomnia Severity Index[7], patient version, 7 items<br>• Hospital Anxiety and Depression Scale[6], 14 items<br>• Resource Use questionnaire (custom instrument), 11 items<br>• Knowledge About Sleep in Childhood (custom scale), 13 items	 • 10 minutes<br>• 5 minutes<br>• 12.5 + 2 minutes*<br>• 12.5 + 2 minutes*<br>• 4.5 + 2 minutes*<br>• 5 minutes<br>• 5 minutes<br>• 2.5 + 2 minutes*<br>• 3.5 + 2 minutes*<br>• 5 minutes<br>• 5.5 + 2 minutes*<br>• 6.5 + 2 minutes*	 • 3<br>• 2<br>• 2<br>• 3<br>• 3<br>• 3<br>• 3<br>• 3<br>• 3<br>• 3<br>• 3<br>• 2	246.5 minutes<br>• 30 minutes<br>• 10 minutes<br>• 29 minutes<br>• 43.5 minutes<br>• 19.5 minutes<br>• 15 minutes<br>• 15 minutes<br>• 13.5 minutes<br>• 16.5 minutes<br>• 15 minutes<br>• 22.5 minutes<br>• 21 minutes
SleepSuite[8] (iPad App)<br>• Morning of single day<br>• Evening of single day	40 minutes<br>• 20 minutes<br>• 20 minutes	2	80 minutes

For peer review only - http://bmjopen.bmj.com/site/about/guidelines.xhtml

Trial component	Time (mins)	Frequency	Overall time (mins)
Actigraphy<br>• Delivery arrangements to participants' home or collection point (incl. SleepSuite iPad)<br>○ Baseline<br>○ Follow-up at 3 months<br>• Return arrangements to participants' home or collection point (incl. SleepSuite iPad)<br>○ Baseline<br>○ Follow-up at 3 months<br>• Use: Removal and re-fitting of device once daily (2 x 0.25 minute) when showering, bathing, or swimming; otherwise, the device is worn like a wristwatch without requiring participant interventions.<br>○ Baseline: 14 days<br>○ Follow-up at 3 months: 14 days	• 15 minutes<br>• 15 minutes<br>• 15 minutes<br>• 15 minutes<br>• 7 minutes<br>• 7 minutes	• 1<br>• 1<br>• 1<br>• 1<br>• 1<br>• 1	74 minutes
Sleep diary<br>Once daily completion of parent- and child diary (2 x 2.5 minutes)<br>• Baseline: 14 days<br>• Follow-up at 3 months: 14 days	• 70 minutes<br>• 70 minutes	• 1<br>• 1	140 minutes
COSI (*intervention arm only*)<br>• 3 mandatory modules (core information about sleep relevant to all families)<br>• 3 recommended modules (e.g. sleep hygiene)<br>• 5 tailored modules (addressing specific sleep issues indicated by a given parent)<br>• List of additional resources, *optional*, 10 webpages, not included in time estimate<br>• Evaluation questionnaire, 3 sections, 47 items overall<br>A parent assigned to COSI (i.e. the intervention arm) would be expected to look at minimally 7 and maximally 11 modules. All modules are self-paced (i.e. do not have a fixed duration). To read and engage with a single module could take anywhere between 5–20 minutes depending on how quickly one reads, whether one watches the videos, does the quizzes, etc. Consequently, the estimated time requirement for initial material completion not including breaks or re-visits is 35–220 minutes for modules alone.<br>To be conservative, maximal estimates are used in calculations.	• 60 minutes<br>• 60 minutes<br>• 100 minutes<br>• 0 minutes<br>• 23.5 + 2 minutes*	• 1<br>• 1<br>• 1<br>• 1<br>• 1	245.5 minutes

For peer review only - http://bmjopen.bmj.com/site/about/guidelines.xhtml

Trial component	Time (mins)	Frequency	Overall time (mins)
Qualitative interviews (*optional*)<br>Two time-points (Follow-up at 3 months + 3 weeks, at 6 months + 3 weeks)<br>• Interview date and time arrangement<br>• Interview preparation using supplied interview guide<br>• Actual interview<br>• De-brief<br>For the qualitative interviews with parents, we typically expect that the total time burden for each of the two interviews would range from 30–70 minutes. However, we will tailor the core interview to fit with the time the parent has available, so some interviews may be a little longer or shorter.<br>To be conservative, maximal estimates are used in calculations.	• 10 minutes<br>• 10 minutes<br>• 40 minutes<br>• 10 minutes	• 2<br>• 2<br>• 2<br>• 2	140 minutes<br>• 20 minutes<br>• 20 minutes<br>• 80 minutes<br>• 20 minutes
**Total time for participation over a 6-months period**<br>• **Standard Care arm (SC), not participating in optional qualitative interviews**<br>• **Standard Care arm (SC), participating in optional qualitative interviews**<br>• **Intervention arm (SC + COSI), not participating in optional qualitative interviews**<br>• **Intervention arm (SC + COSI), participating in optional qualitative interviews**			• **690.5 minutes**<br>• **830.50 minutes**<br>• **936 minutes**<br>• **1076 minutes**

For peer review only

For peer review only - http://bmjopen.bmj.com/site/about/guidelines.xhtml

Supplemental Table 4. Composition, roles and responsibilities of the Trial Management Group, Programme Steering Committee, and Independent Data and Safety Monitoring Committee for CASTLE Sleep-E.

Role	Name (Initials)	Affiliation
**Trial management Group (TMG)**<br>**Responsibilities**: Day-to-day running and management of the trial.<br>**Meeting frequency**: Bi-weekly to three-monthly, depending on trial stage.		
1. King's College Hospital Sponsor Representative	Jasmine Palmer	King's College Hospital NHS Foundation Trust, UK
2. Chief Investigator	Deb K. Pal	King's College London, UK
3. Co-Chief Investigator	Paul Gringras	Evelina London Children's Hospital, UK
4. Co-Investigator<br>Public and Patient Involvement Lead	Lucy Bray	Edge Hill University, UK
5. Co-Investigator<br>Qualitative Research Lead<br>Public and Patient Involvement Co-Lead	Bernie Carter	Edge Hill University, UK
6. Co-Investigator<br>Health Economics Lead	Dyfrig Hughes	Bangor University, UK
7. Co-Investigator<br>Patient Reported Outcome Lead<br>Public and Patient Involvement Co-Lead	Christopher Morris	University of Exeter, UK
8. Co-Investigator<br>Lead Statistician	Catrin Tudur Smith	University of Liverpool, UK
9. Co-Investigator<br>Intervention Development Lead	Luci Wiggs	Oxford Brookes University, UK
10. Supervising Trials Manager	Catherine Spowart	University of Liverpool, UK
11. Trial Manager	Lucy Stibbs-Eaton	University of Liverpool, UK
12. Trial Statistician	Liam Whittle	University of Liverpool, UK
13. CASTLE Programme Manager	Amber Collingwood	King's College London, UK
14. Researcher	Georgia Cook	Oxford Brookes University, UK
15. Researcher	Kristina C. Dietz	King's College London, UK
16. Health economist	Will A. S. Hardy	Bangor University, UK
17. Researcher	Holly Saron	Edge Hill University, UK
**Trial Steering Committee (TSC)**<br>**Responsibilities**: Overall trial supervision and advice, ultimate decision for the continuation of the trial.<br>**Meeting frequency**: At least annually.		
1. Chair	Jeremy Parr	Newcastle University, UK
2. Medical statistician	Martyn Lewis	Keele University, UK
3. Paediatrician	Desaline Joseph	Evelina London Children's Hospital, UK
4. Public and Patient Involvement Representative	Jo Conduit-Smith	CASTLE Advisory Panel
5. Chief Investigator	Deb K. Pal	King's College London, UK
6. Co-Chief Investigator	Paul Gringras	Evelina London Children's Hospital, UK

For peer review only - http://bmjopen.bmj.com/site/about/guidelines.xhtml

Independent Data and Safety Monitoring Committee (IDSMC)<br>**Responsibilities:** Interim monitoring of safety and effectiveness, trial conduct and external data. Recommendation to TSC about trial continuation.<br>**Meeting frequency:** At least annually		
1. Chair	Helen Cross	University College London, UK
2. Paediatrician	Alberto Verroti	University of L'aquila, Italy
3. Medical statistician	• Anthony Johnson (to 31/August/2022)<br>• Appointment pending (07/November/2022)	University College London, UK

For peer review only

For peer review only - http://bmjopen.bmj.com/site/about/guidelines.xhtml

Supplemental Table 5. Categories used to define the causality and severity of Adverse Events in CASTLE Sleep-E

Category	Definition
Causality	
Almost Certainly	There is clear evidence to suggest a causal relationship, and other possible contributing factors can be ruled out.
Probably	There is evidence to suggest a causal relationship, and the influence of other factors is unlikely.
Possibly	There is some evidence to suggest a causal relationship (e.g. the event occurred within a reasonable time after administration of the study procedure). However, the influence of other factors may have contributed to the event (e.g. the participant's clinical condition, other concomitant events).
Unlikely	There is little evidence to suggest there is a causal relationship (e.g. the event did not occur within a reasonable time after administration of the study procedure). There is another reasonable explanation for the event (e.g. the participant's clinical condition).
Not related	There is no evidence of any causal relationship.
Severity	
Mild	The Adverse Event does not interfere with the participant's daily routine and does not require further procedure; it causes slight discomfort.
Moderate	The Adverse Event interferes with some aspects of the participant's routine, or requires further procedure, but is not damaging to health; it causes moderate discomfort.
Severe	The Adverse Event results in alteration, discomfort or disability which is clearly damaging to health.

For peer review only

For peer review only - http://bmjopen.bmj.com/site/about/guidelines.xhtml

References

1. Owens JA, Spirito A, McGuinn M. The Children's Sleep Habits Questionnaire (CSHQ): psychometric properties of a survey instrument for school-aged children. Sleep 2000;**23**(8):1043-51.
2. Wille N, Badia X, Bonsel G, et al. Development of the EQ-5D-Y: a child-friendly version of the EQ-5D. Qual Life Res 2010;**19**(6):875-86 doi: 10.1007/s11136-010-9648-y [published Online First: 20100420].
3. EuroQol Research Foundation. EQ-5D-Y User Guide, 2020.
4. Stevens K. Valuation of the Child Health Utility 9D Index. Pharmacoeconomics 2012;**30**(8):729-47 doi: 10.2165/11599120-000000000-00000.
5. Devlin NJ, Shah KK, Feng Y, et al. Valuing health-related quality of life: An EQ-5D-5L value set for England. Health Econ 2018;**27**(1):7-22 doi: 10.1002/hec.3564 [published Online First: 20170822].
6. Zigmond AS, Snaith RP. The hospital anxiety and depression scale. Acta Psychiatr Scand 1983;**67**(6):361-70 doi: 10.1111/j.1600-0447.1983.tb09716.x.
7. Morin CM, Belleville G, Bélanger L, et al. The Insomnia Severity Index: psychometric indicators to detect insomnia cases and evaluate treatment response. Sleep 2011;**34**(5):601-08 doi: 10.1093/sleep/34.5.601.
8. Colonna A, Smith AB, Smith S, et al. The Effects of Sleep on Emotional Target Detection Performance: A Novel iPad-Based Pediatric Game. Frontiers in psychology 2018;**9**:241-41 doi: 10.3389/fpsyg.2018.00241.
9. Sadaka Y, Sadeh A, Bradbury L, et al. Validation of actigraphy with continuous video-electroencephalography in children with epilepsy. Sleep Med 2014;**15**(9):1075-81 doi: 10.1016/j.sleep.2014.04.021 [published Online First: 20140602].
10. Ronen GM, Streiner DL, Rosenbaum P, et al. Health-related quality of life in children with epilepsy: development and validation of self-report and parent proxy measures. Epilepsia 2003;**44**(4):598-612 doi: 10.1046/j.1528-1157.2003.46302.x.
11. Allgaier A-K, Pietsch K, Frühe B, et al. Depression in pediatric care: is the WHO-Five Well-Being Index a valid screening instrument for children and adolescents? General Hospital Psychiatry 2012;**34**(3):234-41 doi: https://doi.org/10.1016/j.genhosppsych.2012.01.007.
12. Goodman R. Psychometric Properties of the Strengths and Difficulties Questionnaire. Journal of the American Academy of Child & Adolescent Psychiatry 2001;**40**(11):1337-45 doi: 10.1097/00004583-200111000-00015.
13. Dumka LE, Stoerzinger HD, Jackson KM, et al. Examination of the Cross-Cultural and Cross-Language Equivalence of the Parenting Self-Agency Measure. Family Relations 1996;**45**(2):216-22 doi: 10.2307/585293.

For peer review only - http://bmjopen.bmj.com/site/about/guidelines.xhtml

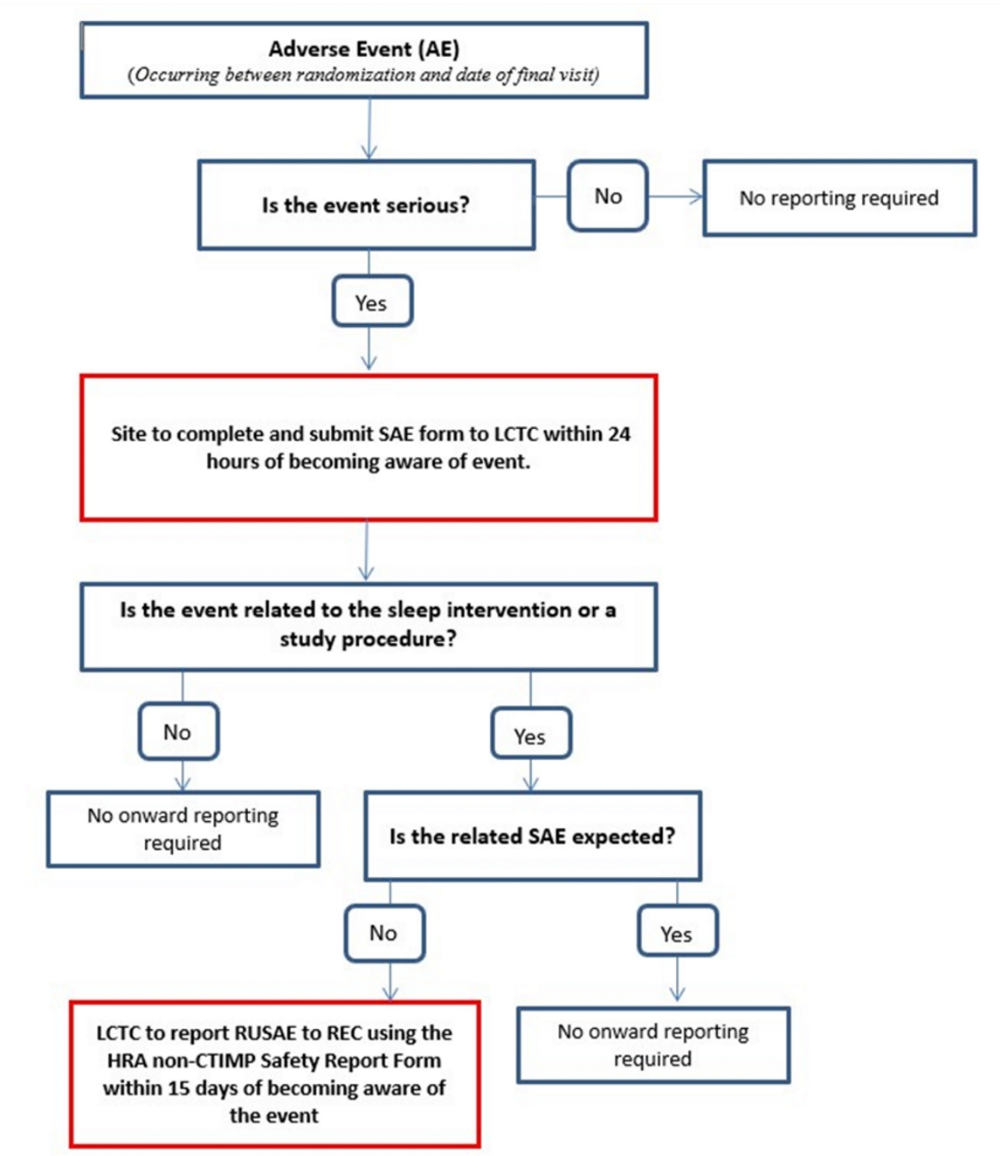

Supplemental Figure 1. Flowchart showing reporting requirements of Adverse Events for the CASTLE Sleep-E trial. Acronyms: Serious Adverse Event (SAE), Liverpool Clinical Trial Centre (LCTC), Related Unexpected Serious Adverse Event (RUSAE), Health Research Authority (HRA), non- Clinical Trial of Investigational Medicinal Products (non-CTIMP).

2184x2612mm (38 x 38 DPI)

STANDARD PROTOCOL ITEMS: RECOMMENDATIONS FOR INTERVENTIONAL TRIALS

SPIRIT 2013 Checklist: Recommended items to address in a clinical trial protocol and related documents*

Section/item	Item No	Description	Addressed on page number
**Administrative information**			
Title	1	Descriptive title identifying the study design, population, interventions, and, if applicable, trial acronym	p1
Trial registration	2a	Trial identifier and registry name. If not yet registered, name of intended registry	p2, Suppl. Table 1 (item 1)
	2b	All items from the World Health Organization Trial Registration Data Set	Suppl. Table 1
Protocol version	3	Date and version identifier	Suppl. Table 1 (item 25)
Funding	4	Sources and types of financial, material, and other support	p22, Suppl. Table 1 (item 4)
Roles and responsibilities	5a	Names, affiliations, and roles of protocol contributors	p1-2, p22
	5b	Name and contact information for the trial sponsor	Suppl. Table 1 (items 5, 6)
	5c	Role of study sponsor and funders, if any, in study design; collection, management, analysis, and interpretation of data; writing of the report; and the decision to submit the report for publication, including whether they will have ultimate authority over any of these activities	p22

For peer review only

	5d	Composition, roles, and responsibilities of the coordinating centre, steering committee, endpoint adjudication committee, data management team, and other individuals or groups overseeing the trial, if applicable (see Item 21a for data monitoring committee)	Suppl. Table 3
**Introduction**			
Background and rationale	6a	Description of research question and justification for undertaking the trial, including summary of relevant studies (published and unpublished) examining benefits and harms for each intervention	p5
	6b	Explanation for choice of comparators	p5
Objectives	7	Specific objectives or hypotheses	Table 5, p18-19
Trial design	8	Description of trial design including type of trial (eg, parallel group, crossover, factorial, single group), allocation ratio, and framework (eg, superiority, equivalence, noninferiority, exploratory)	p8
**Methods: Participants, interventions, and outcomes**			
Study setting	9	Description of study settings (eg, community clinic, academic hospital) and list of countries where data will be collected. Reference to where list of study sites can be obtained	p8
Eligibility criteria	10	Inclusion and exclusion criteria for participants. If applicable, eligibility criteria for study centres and individuals who will perform the interventions (eg, surgeons, psychotherapists)	Suppl. Table 1 (item 14)
Interventions	11a	Interventions for each group with sufficient detail to allow replication, including how and when they will be administered	p8-9, Table 4
	11b	Criteria for discontinuing or modifying allocated interventions for a given trial participant (eg, drug dose change in response to harms, participant request, or improving/worsening disease)	p9
	11c	Strategies to improve adherence to intervention protocols, and any procedures for monitoring adherence (eg, drug tablet return, laboratory tests)	p9

For peer review only - http://bmjopen.bmj.com/site/about/guidelines.xhtml

	11d	Relevant concomitant care and interventions that are permitted or prohibited during the trial	p9
Outcomes	12	Primary, secondary, and other outcomes, including the specific measurement variable (eg, systolic blood pressure), analysis metric (eg, change from baseline, final value, time to event), method of aggregation (eg, median, proportion), and time point for each outcome. Explanation of the clinical relevance of chosen efficacy and harm outcomes is strongly recommended	Table 1, Suppl. Table 2
Participant timeline	13	Time schedule of enrolment, interventions (including any run-ins and washouts), assessments, and visits for participants. A schematic diagram is highly recommended (see Figure)	Table 4
Sample size	14	Estimated number of participants needed to achieve study objectives and how it was determined, including clinical and statistical assumptions supporting any sample size calculations	p13
Recruitment	15	Strategies for achieving adequate participant enrolment to reach target sample size	p13
**Methods: Assignment of interventions (for controlled trials)**			
Allocation:			
Sequence generation	16a	Method of generating the allocation sequence (eg, computer-generated random numbers), and list of any factors for stratification. To reduce predictability of a random sequence, details of any planned restriction (eg, blocking) should be provided in a separate document that is unavailable to those who enrol participants or assign interventions	p13
Allocation concealment mechanism	16b	Mechanism of implementing the allocation sequence (eg, central telephone; sequentially numbered, opaque, sealed envelopes), describing any steps to conceal the sequence until interventions are assigned	p13
Implementation	16c	Who will generate the allocation sequence, who will enrol participants, and who will assign participants to interventions	p13, p8
Blinding (masking)	17a	Who will be blinded after assignment to interventions (eg, trial participants, care providers, outcome assessors, data analysts), and how	p13
	17b	If blinded, circumstances under which unblinding is permissible, and procedure for revealing a participant's allocated intervention during the trial	p13

Methods: Data collection, management, and analysis

Data collection methods	18a	Plans for assessment and collection of outcome, baseline, and other trial data, including any related processes to promote data quality (eg, duplicate measurements, training of assessors) and a description of study instruments (eg, questionnaires, laboratory tests) along with their reliability and validity, if known. Reference to where data collection forms can be found, if not in the protocol	p15-17, Table 4, Suppl. Table 1 (item 8), Suppl. Table 2
	18b	Plans to promote participant retention and complete follow-up, including list of any outcome data to be collected for participants who discontinue or deviate from intervention protocols	p9
Data management	19	Plans for data entry, coding, security, and storage, including any related processes to promote data quality (eg, double data entry; range checks for data values). Reference to where details of data management procedures can be found, if not in the protocol	p15-17, Suppl. Table 1 (item 8)
Statistical methods	20a	Statistical methods for analysing primary and secondary outcomes. Reference to where other details of the statistical analysis plan can be found, if not in the protocol	p16-17, Table 5, Suppl. Table 1 (item 8)
	20b	Methods for any additional analyses (eg, subgroup and adjusted analyses)	p16-17, Table 5
	20c	Definition of analysis population relating to protocol non-adherence (eg, as randomised analysis), and any statistical methods to handle missing data (eg, multiple imputation)	p16-17

Methods: Monitoring

Data monitoring	21a	Composition of data monitoring committee (DMC); summary of its role and reporting structure; statement of whether it is independent from the sponsor and competing interests; and reference to where further details about its charter can be found, if not in the protocol. Alternatively, an explanation of why a DMC is not needed	p22, Suppl. Table 1 (item 8), Suppl . Table 3
	21b	Description of any interim analyses and stopping guidelines, including who will have access to these interim results and make the final decision to terminate the trial	p13, Suppl. Table 3
Harms	22	Plans for collecting, assessing, reporting, and managing solicited and spontaneously reported adverse events and other unintended effects of trial interventions or trial conduct	p20, Suppl. Table 4, Suppl. Figure 1

For peer review only - http://bmjopen.bmj.com/site/about/guidelines.xhtml

Auditing	23	Frequency and procedures for auditing trial conduct, if any, and whether the process will be independent from investigators and the sponsor	p20
**Ethics and dissemination**			
Research ethics approval	24	Plans for seeking research ethics committee/institutional review board (REC/IRB) approval	p3, p20, Suppl. Table 1 (item 21)
Protocol amendments	25	Plans for communicating important protocol modifications (eg, changes to eligibility criteria, outcomes, analyses) to relevant parties (eg, investigators, REC/IRBs, trial participants, trial registries, journals, regulators)	p20
Consent or assent	26a	Who will obtain informed consent or assent from potential trial participants or authorised surrogates, and how (see Item 32)	p15
	26b	Additional consent provisions for collection and use of participant data and biological specimens in ancillary studies, if applicable	N/A
Confidentiality	27	How personal information about potential and enrolled participants will be collected, shared, and maintained in order to protect confidentiality before, during, and after the trial	p15-p16
Declaration of interests	28	Financial and other competing interests for principal investigators for the overall trial and each study site	p22
Access to data	29	Statement of who will have access to the final trial dataset, and disclosure of contractual agreements that limit such access for investigators	p3, p16, Suppl. Table 1 (item 24)
Ancillary and post-trial care	30	Provisions, if any, for ancillary and post-trial care, and for compensation to those who suffer harm from trial participation	p20
Dissemination policy	31a	Plans for investigators and sponsor to communicate trial results to participants, healthcare professionals, the public, and other relevant groups (eg, via publication, reporting in results databases, or other data sharing arrangements), including any publication restrictions	p3, p20
	31b	Authorship eligibility guidelines and any intended use of professional writers	p22
	31c	Plans, if any, for granting public access to the full protocol, participant-level dataset, and statistical code	p3, p20, Suppl. Table 1 (item 24)

For peer review only - http://bmjopen.bmj.com/site/about/guidelines.xhtml

Appendices

Informed consent materials	32	Model consent form and other related documentation given to participants and authorised surrogates	Suppl. Materials: Patient Information and Consent Sheet
Biological specimens	33	Plans for collection, laboratory evaluation, and storage of biological specimens for genetic or molecular analysis in the current trial and for future use in ancillary studies, if applicable	N/A

*It is strongly recommended that this checklist be read in conjunction with the SPIRIT 2013 Explanation & Elaboration for important clarification on the items. Amendments to the protocol should be tracked and dated. The SPIRIT checklist is copyrighted by the SPIRIT Group under the Creative Commons "Attribution-NonCommercial-NoDerivs 3.0 Unported" license.

For peer review only

The SPIRIT-PRO Protocol Guidance Checklist

Protocol Section	SPIRIT-PRO Item	Recommended Content	Page Addressed
**Administrative Information**			
Roles and responsibilities	SPIRIT-5a-PRO Elaboration	Specify the individual(s) responsible for the PRO content of the trial protocol.	p22
**Introduction**			
Background and rationale	SPIRIT-6a-PRO Extension	Describe the PRO-specific research question and rationale for PRO assessment and summarize PRO findings in relevant studies.	p5, p11
Objectives	SPIRIT-7-PRO Extension	State specific PRO objectives or hypotheses (including relevant PRO concepts/domains).	p5, p13, Table 5, Suppl. Table 2
**Methods: Participants, Interventions, and Outcomes**			
Eligibility criteria	SPIRIT-10-PRO Extension	Specify any PRO-specific eligibility criteria (eg, language/reading requirements or pre-randomization completion of PRO). If PROs will not be collected from the entire study sample, provide a rationale and describe the method for obtaining the PRO subsample.	Suppl. Table 1 (item 14)
Outcomes	SPIRIT-12-PRO Extension	Specify the PRO concepts/domains used to evaluate the intervention (eg, overall health-related quality of life, specific domain, specific symptom) and, for each one, the analysis metric (eg, change from baseline, final value, time to event) and the principal time point or period of interest.	Table 1, Table 4, Table 5
Participant timeline	SPIRIT-13-PRO Extension	Include a schedule of PRO assessments, providing a rationale for the time points, and justifying if the initial assessment is not pre-randomization. Specify time windows, whether PRO collection is prior to clinical assessments, and, if using multiple questionnaires, whether order of administration will be standardized.	Table 4
Sample size	SPIRIT-14-PRO Extension	When a PRO is the primary end point, state the required sample size (and how it was determined) and recruitment target (accounting for expected loss to follow-up). If sample size is not established based on the PRO end point, then discuss the power of the principal PRO analyses.	p13

Calvert M, Kyte D, Mercieca-Bebber R, et al. Guidelines for Inclusion of Patient-Reported Outcomes in Clinical Trial Protocols: The SPIRIT-PRO Extension. *JAMA*. 018;319(5):483-494. doi:10.1001/jama.2017.21903

<u>Note</u>: The SPIRIT-PRO Extension should be used with the SPIRIT 2013 Statement and any other relevant SPIRIT Extensions, found at spirit-statement.org

For peer review only - http://bmjopen.bmj.com/site/about/guidelines.xhtml

The SPIRIT-PRO Protocol Guidance Checklist

Protocol Section	SPIRIT-PRO Item	Recommended Content	Page Addressed
**Methods: Data Collection, Management, and Analysis**			
Data collection methods	SPIRIT-18a(i)-PRO Extension	Justify the PRO instrument to be used and describe domains, number of items, recall period, and instrument scaling and scoring (eg, range and direction of scores indicating a good or poor outcome). Evidence of PRO instrument measurement properties, interpretation guidelines, and patient acceptability and burden should be provided or cited if available, ideally in the population of interest. State whether the measure will be used in accordance with any user manual and specify and justify deviations if planned.	Suppl. Table 2
	SPIRIT-18a(ii)-PRO Extension	Include a data collection plan outlining permitted mode(s) of administration (eg, paper, telephone, electronic, other) and setting (eg, clinic, home, other).	p15-16
	SPIRIT-18a(iii)-PRO Extension	Specify whether more than 1 language version will be used and state whether translated versions have been developed using currently recommended methods.	Suppl. Table 1 (item 14)
	SPIRIT-18a(iv)-PRO Extension	When the trial context requires someone other than a trial participant to answer on his or her behalf (a proxy-reported outcome), state and justify the use of a proxy respondent. Provide or cite evidence of the validity of proxy assessment if available.	Table 1, Table 4
	SPIRIT-18b(i)-PRO Extension	Specify PRO data collection and management strategies for minimizing avoidable missing data.	p9, p13, p15-16, p20
	SPIRIT-18b(ii)-PRO Elaboration	Describe the process of PRO assessment for participants who discontinue or deviate from the assigned intervention protocol.	p9
Statistical methods	SPIRIT-20a-PRO Elaboration	State PRO analysis methods, including any plans for addressing multiplicity/ type I (α) error.	p16-17
	SPIRIT-20c-PRO Elaboration	State how missing data will be described and outline the methods for handling missing items or entire assessments (eg, approach to imputation and sensitivity analyses).	p16-17
**Methods: Monitoring**			
Harms	SPIRIT-22-PRO Extension	State whether or not PRO data will be monitored during the study to inform the clinical care of individual trial participants and, if so, how this will be managed in a standardized way. Describe how this process will be explained to participants; eg, in the participant information sheet and consent form.	p13, p16, p20, Suppl. Table 3, Suppl. Materials: Patient

Calvert M, Kyte D, Mercieca-Bebber R, et al. Guidelines for Inclusion of Patient-Reported Outcomes in Clinical Trial Protocols: The SPIRIT-PRO Extension. *JAMA*. 018;319(5):483-494. doi:10.1001/jama.2017.21903

Note: The SPIRIT-PRO Extension should be used with the SPIRIT 2013 Statement and any other relevant SPIRIT Extensions, found at spirit-statement.org

For peer review only

The SPIRIT-PRO Protocol Guidance Checklist

			Information and Consent Sheet

Calvert M, Kyte D, Mercieca-Bebber R, et al. Guidelines for Inclusion of Patient-Reported Outcomes in Clinical Trial Protocols: The SPIRIT-PRO Extension. *JAMA*. 018;319(5):483-494. doi:10.1001/jama.2017.21903

Note: The SPIRIT-PRO Extension should be used with the SPIRIT 2013 Statement and any other relevant SPIRIT Extensions, found at spirit-statement.org

For peer review only

BMJ Open

Changing Agendas on Sleep, Treatment and Learning in Epilepsy (CASTLE) Sleep-E: A protocol for a randomised controlled trial comparing an online behavioural sleep intervention with standard care in children with Rolandic epilepsy

Journal:	*BMJ Open*
Manuscript ID	bmjopen-2022-065769.R2
Article Type:	Protocol
Date Submitted by the Author:	20-Dec-2022

Complete List of Authors:	Group, CASTLE; King's College London<br>Advisory Panel, CASTLE; Edge Hill University<br>Al-Najjar, Nadia; University of Liverpool<br>Bray, Lucy; Edge Hill University<br>Carter, Bernie; Edge Hill University<br>Collingwood, Amber; King's College London<br>Cook, Georgia; Oxford Brookes University<br>Crudgington, Holly; King's College London<br>Dietz, Kristina; King's College London<br>Gringras, Paul; Evelina London Children's Healthcare<br>Hardy, Will A. S.; Bangor University<br>Hiscock, Harriet; Murdoch Children's Research Institute<br>Hughes, Dyfrig; Bangor University<br>Morris, Christopher; University of Exeter<br>Pal, Deb; King's College London<br>Rouncefield-Swales, Alison; Edge Hill University<br>Saron, Holly; Edge Hill University<br>Spowart, Catherine; University of Liverpool<br>Stibbs-Eaton, Lucy; University of Liverpool<br>Tudur-Smith, Catrin; University of Liverpool<br>Watson, Victoria; University of Liverpool<br>Whittle, Liam; University of Liverpool<br>Wiggs, Luci; Oxford Brookes University<br>Williamson, Paula; University of Liverpool<br>Wood, Eifiona; Bangor University, CHEME

**Primary Subject Heading**:	Neurology
Secondary Subject Heading:	Paediatrics, Evidence based practice, Health economics, Qualitative research, Patient-centred medicine
Keywords:	Epilepsy < NEUROLOGY, Paediatric neurology < NEUROLOGY, SLEEP MEDICINE, Clinical trials < THERAPEUTICS, QUALITATIVE RESEARCH

SCHOLARONE™
Manuscripts

TITLE

Changing Agendas on Sleep, Treatment and Learning in Epilepsy (CASTLE) Sleep-E: A protocol for a randomised controlled trial comparing an online behavioural sleep intervention with standard care in children with Rolandic epilepsy

Authorship including affiliations and ORCID IDs (alphabetic surname order)

1. CASTLE Group, King's College London, UK, castlesleepe@liverpool.ac.uk
2. CASTLE Advisory Panel (CAP), CASTLE Research Programme, UK, martinra@edgehill.ac.uk
3. Nadia Al-Najjar (NA-N), University of Liverpool, UK, Nadia.Al-Najjar@liverpool.ac.uk
4. Lucy Bray (LB), Edge Hill University, UK, brayl@edgehill.ac.uk http://orcid.org/0000-0001-8414-3233
5. Bernie Carter (BC), Edge Hill University, UK, bernie.carter@edgehill.ac.uk http://orcid.org/0000-0001-5226-9878
6. Amber Collingwood (AC), King's College London, UK, amber.collingwood@kcl.ac.uk
7. Georgia Cook (GC), Oxford Brookes University, UK, gcook@brookes.ac.uk http://orcid.org/0000-0002-1651-866X
8. Holly Crudgington (HC), King's College London, UK, holly.1.crudgington@kcl.ac.uk http://orcid.org/0000-0003-1048-4953
9. Kristina C. Dietz (KCD), King's College London, UK, kristina.dietz@kcl.ac.uk http://orcid.org/0000-0002-3074-6319
10. Paul Gringras (PG), Evelina London Children's Hospital, UK, paul.gringras@gstt.nhs.uk http://orcid.org/0000-0002-0495-3517
11. Will A. S. Hardy (WASH), Bangor University, UK, w.hardy@bangor.ac.uk http://orcid.org/0000-0001-5227-567X
12. Harriet Hiscock (HH), Murdoch Children's Research Institute, Australia, harriet.hiscock@mcri.edu.au http://orcid.org/0000-0003-3017-2770
13. Dyfrig Hughes (DH) Bangor University, UK, d.a.hughes@bangor.ac.uk http://orcid.org/0000-0001-8247-7459
14. Christopher Morris (CM), University of Exeter, UK, Christopher.Morris@exeter.ac.uk http://orcid.org/0000-0002-9916-507X
15. Deb K. Pal (DKP), King's College London, UK, deb.pal@kcl.ac.uk http://orcid.org/0000-0003-2655-0564
16. Alison Rouncefield-Swales (AR-S), University of Central Lancashire, UK, rouncefia@edgehill.ac.uk, https://orcid.org/0000-0001-9947-7375
17. Holly Saron (HS) Edge Hill University, UK, saronh@edgehill.ac.uk http://orcid.org/0000-0001-7563-3409
18. Catherine Spowart (CS), University of Liverpool, UK Catherine.Spowart@liverpool.ac.uk http://orcid.org/0000-0001-8641-2871
19. Lucy Stibbs-Eaton (LS-E), University of Liverpool, UK, l.stibbs-eaton@liverpool.ac.uk http://orcid.org/0000-0002-3672-4006
20. Catrin Tudur Smith (CTS), University of Liverpool, UK, Cat1@liverpool.ac.uk http://orcid.org/0000-0003-3051-1445
21. Victoria Watson (VW), University of Liverpool, UK, victoria.watson@liverpool.ac.uk
22. Liam Whittle (LWh), University of Liverpool, UK, Liam.Whittle@liverpool.ac.uk, https://orcid.org/0000-0001-8280-1984

For peer review only

23. Luci Wiggs (LW), Oxford Brookes University, UK, lwiggs@brookes.ac.uk
http://orcid.org/0000-0002-5697-6550
24. Paula R. Williamson (PRW), University of Liverpool, UK, P.R.Williamson@liverpool.ac.uk
25. Eifiona Wood (EW), Bangor University, UK, e.wood@bangor.ac.uk
https://orcid.org/0000-0002-2785-7325

Corresponding author: Dr Kristina C Dietz, Maurice Wohl Clinical Neuroscience Institute, King's College London, 5 Cutcombe Road, London, SE5 9RX, kristina.dietz@kcl.ac.uk, +44 (0) 207 848 025 9

Keywords (Medical Subject Headings 2022): Epilepsy, Rolandic; Sleep; Child; Randomized Controlled Trials as Topic; Evidence-Based Medicine

Word count: 3 998

For peer review only

ABSTRACT

Introduction Sleep and epilepsy have an established bi-directional relationship yet only one randomised controlled clinical trial has assessed the effectiveness of behavioural sleep interventions for children with epilepsy. The intervention was successful, but was delivered via face-to-face educational sessions with parents, which are costly and non-scalable to population level. The Changing Agendas on Sleep, Treatment and Learning in Epilepsy (CASTLE) Sleep-E trial addresses this problem by comparing clinical- and cost-effectiveness in children with Rolandic epilepsy between standard care and standard care augmented with a novel, tailored parent-led CASTLE Online Sleep Intervention (COSI) that incorporates evidence-based behavioural components.

Methods and analyses CASTLE Sleep-E is a UK-based, multi-centre, open label, active concurrent control, randomised, parallel-group, pragmatic superiority trial. A total of 110 children with Rolandic epilepsy will be recruited in out-patient clinics and allocated 1:1 to standard care (SC) or standard care augmented with COSI (SC + COSI). Primary clinical outcome is parent-reported sleep problem score (Children's Sleep Habits Questionnaire). Primary health economic outcome is the Incremental Cost Effectiveness Ratio (National Health Service and Personal Social Services perspective, Child Health Utility 9D instrument). Parents and children (≥ 7 years) can opt into qualitative interviews and activities to share their experiences and perceptions of trial participation and managing sleep with Rolandic epilepsy.

Ethics and dissemination The CASTLE Sleep-E protocol was approved by the Health Research Authority East Midlands (HRA) – Nottingham 1 Research Ethics Committee, reference: 21/EM/0205. Trial results will be disseminated to scientific audiences, families, professional groups, managers, commissioners, and policy makers. Pseudo-anonymised Individual Patient Data will be made available after dissemination on reasonable request.

Registration details ISRCTN registry (Trial ID: ISRCTN13202325, prospective registration 09/Sep/2021). See Supplemental Table 1 for the World Health Organisation Trial Registration Data Set (Version 1.3.1).

For peer review only

Strengths and limitations of this study

- First randomised controlled trial to evaluate the clinical- and cost-effectiveness of a novel, tailored, parent-led CASTLE Online Sleep Intervention (COSI) that incorporates evidence-based behavioural components for children with Rolandic epilepsy
- Extensive Patient and Public Involvement via dedicated CASTLE Advisory Panel
- Embedded health economic evaluation
- **Limitation**: Heavily reliant on parent and child self-report to assess intervention implementation, ameliorated by COSI e-analytics and actigraphy data

For peer review only

INTRODUCTION

Epilepsy is one of the most common long-term neurological conditions worldwide whose prevalence peaks during childhood (5–9 years) and late in life (over 80 years).[1] Epilepsy in children (5 to <13 years) accounts for the annual loss of 2.6 million disability-adjusted life years, equivalent to 1.8 % of the global burden of disease among children and adolescents.[2] Rolandic Epilepsy (RE) is the most common childhood epilepsy.[3]

In the UK, RE has a stable crude incidence rate of 5 in 100 000 children (<16 years) or 542 new cases annually.[4] Concurrent neuro-developmental disorders are very common (35 %).[5] Seizures are often triggered by sleep fragmentation.[6] Many parents co-sleep or monitor children with nocturnal seizures, and children experience a fear of death during and after a seizure.[7] Problems related to sleep emerge as a top concerns for both children and parents,[8] but are often unaddressed.[9 10]

A recent systematic review and meta-analysis of clinical trials shows that parent-based behavioural sleep interventions are effective for typically-developing children and those with neurological and neuro-developmental disorders.[10] The review concluded that randomised controlled clinical trials assessing functional outcomes (e.g. cognition, emotion, behaviour) and targeting specific populations (e.g. epilepsy) are missing (but see two recent trials).[11 12] Harms capture for cognitive-behavioural and behavioural sleep interventions has been sparse (only 32.3 % of trials address Adverse Events) and predominantly inadequate (92.9 % of trials do not meet adequate reporting criteria).[13] Observed harms of behavioural sleep interventions in adults have been mild (e.g. transient fatigue/exhaustion from sleep restriction in insomnia in 25–33 % of participants).[14] The only published paediatric and adult epilepsy trials did not address harms.[11 12] Based on the existing evidence, the benefits of behavioural sleep interventions in children with epilepsy outweigh potential harms, especially because sleep problems not only affect seizure control, but overall child well-being, learning and memory, and parental quality of life.[9 10] There remains, however, uncertainty whether sleep interventions, which can be resource intensive, are cost-effective in public health systems.

This protocol describes the design for the Changing Agendas on Sleep, Treatment and Learning in Epilepsy (CASTLE) Sleep-E trial, which evaluates the clinical- and cost-effectiveness of a novel, tailored, parent-led CASTLE Online Sleep Intervention (COSI) that incorporates evidence-based behavioural components for children with epilepsy. COSI and CASTLE Sleep-E outcome-selection were co-produced by affected children, young people, and their parents, sleep- and epilepsy experts.[8 15-17] The CASTLE Sleep-E protocol follows Standard Protocol Items: Recommendations for Interventional Trials (SPIRIT),[18 19] its extension for Patient Reported Outcomes (SPIRIT-PRO),[20] and the Guidance for Reporting Involvement of Patients and the Public (GRIPP2).[21]

As CASTLE Sleep-E is a pragmatic superiority trial assessing whether UK standard care for children with RE should be augmented with an online behavioural sleep intervention, standard care is the appropriate comparator.[22-24] Current UK clinical guidelines[25-27] recommend that standard care for children with RE consist of a comprehensive care plan with the option of pharmacological treatment with anti-epileptic drugs (AEDs).

The primary objective of CASTLE Sleep-E is to determine if standard care augmented with COSI is superior to standard care alone in reducing sleep problems in children with RE and cost-effective. Implementation details and secondary objectives are reported in Table 1.

Table 1. Outcomes for CASTLE Sleep-E (incl. participant level metrics, time-points, aggregation method). Child measures may be collected by parent proxy.

**Outcome type**	**Specific measurement variable**	**Collected for**	**Participant-level analysis metric**	**Measurement time-point(s)**
Primary				
1. Clinical	Children's Sleep Habits Questionnaire[28]	Child	Total score	Baseline, 3 months
2. Health economic	Cost utility of COSI[a]: National Health Service and Personal Social Services perspective, using outcomes 13–15	Child and Parent	• Time integral of utility<br>• Total costs	Baseline, 3 months, 6 months, (PLICS and HES at 6 months only)
Secondary				
1. Clinical	Children's Sleep Habits Questionnaire[28]	Child	Total score	Baseline, 6 months
2. Clinical	Seizure-free period	Child	Time to first seizure from randomisation (days)	Randomisation, 3 months, 6 months
3. Clinical	Seizure remission	Child	Time to 6-months seizure remission from randomisation (days)	
4. Clinical	Knowledge about Sleep in Childhood (unpublished custom-scale)	Parent	Total score	Baseline, 3 months
5. Clinical	Hospital Anxiety and Depression Scale[29]	Parent	Total score	Baseline, 3 months, 6 months
6. Clinical	Insomnia Severity Index[30]	Parent	Total score	
7. Clinical	SleepSuite[31] (iPad App)	Child	Reaction time (ms)<br>Executive function (accuracy)	Baseline, 3 months
8. Clinical	• Health-Related Quality Of Life Measure for Children with Epilepsy[32]<br>• World Health Organisation – Five Well-Being Index[33]	Child<br><br>Parent	• Total score<br><br>• Total score	Baseline. 6 months
9. Clinical	Strengths and Difficulties Questionnaire[34]	Child	Total score	Baseline, 3 months, 6 months
10. Clinical	Parenting Self Agency Measure[35]	Parent	Total score	
11. Clinical	Actigraphy[36]	Child and Parent	• Total sleep time (minutes)<br>• Sleep latency (minutes)<br>• Sleep efficiency (% asleep of sleep period)<br>All 2-week averages	Baseline, 3 months

[a] Reported as incremental cost per Quality-Adjusted Life Year (QALY) gained

For peer review only

Outcome type	Specific measurement variable	Collected for	Participant-level analysis metric	Measurement time-point(s)
12. Clinical	Sickness-related school absences	Child	Total number of days	Randomisation,3 months, 6 months
13. Health economic	Health-utilities derived from:<br>• EQ-5D-Y[37]<br>• Child Health Utility instrument[38]<br>• EQ-5D-5L[39]	Child and Parent<br>• Child<br>• Child<br>• Parent	Total score<br>• Utility score<br>• Utility score<br>• Utility score	Baseline, 3 months, 6 months
14. Health economic	Insomnia Severity Index mapped to EQ-5D health state utilities[40]	Parent	Total score<br>• Utility score	Baseline, 3 months, 6 months
15. Health economic	Direct costs: National Health Service and Personal Social Services perspective, measured using<br>• Resource Use Questionnaire<br>• Case Report Form data<br>• Patient Level Information and Costing System (PLICS) data<br>• Hospital Episode Statistics (HES) data<br>• Serious Adverse Events (assessed at 3 months, 6 months)	Child	Resource use and total cost	Baseline, 3 months, 6 months, (PLICS and HES at 6 months only)
16. Health economic	Indirect and direct non-medical costs, measured using:<br>• Resource Use Questionnaire<br>• Case Report Form data	Child and Parent	Resource use and total cost	Baseline, 3 months, 6 months
17. Health economic	Cost utility of COSI: Societal perspective, using Quality-Adjusted Life Years and Cost using outcomes 13, 14, and 16	Child and Parent	• Quality-adjusted life years from the time-integral of utility<br>• Mean of total costs	Baseline, 3 months, 6 months
Qualitative	Trial experience	Child and Parent	Qualitative interview transcript<br>Activity booklet transcript/photos	3 months + 3 weeks<br>6 months + 3 weeks

For peer review only

METHODS AND ANALYSES

Trial design

CASTLE Sleep-E is a UK-based, multi-centre, open-label, active concurrent control, randomised (1:1), parallel-group, pragmatic superiority trial (overall trial start date: 14/May/2018, first trial site opened: 12/May/2022, first recruitment: 30/August/2022, planned trial end date: 31/July/2023). Compared are clinical- and cost-effectiveness of standard care (SC) alone and SC augmented with a novel, tailored, parent-led CASTLE Online Sleep Intervention (SC + COSI) in reducing sleep problems in children (5 to <13 years) with RE at 3- and 6 months after randomisation. Parents and children (≥ 7 years) can opt into qualitative interviews and activities to share their experiences and perceptions within 3 weeks of completion of other data collection at 3- and 6 months after randomisation.

Patient and Public Involvement

The CASTLE programme (which subsumes CASTLE Sleep-E) recruited a dedicated Patient and Public Involvement (PPI) Advisory Panel (AP) through social media and epilepsy charities in 2017. The CASTLE Advisory Panel (CAP) consists of 17 adults with experience of childhood epilepsy and five children with epilepsy (aged 6–15 years). CAP has been involved in CASTLE from the funding application onward (2 CAP members are co-applicants). Full PPI details are provided in GRIPP2 Short Form in Table 2.

Trial setting and eligibility criteria

Participants will be identified by staff in NHS out-patient general paediatric and paediatric epilepsy clinics in the UK (pre-dominantly urban setting). Eligibility criteria for participants are reported in Supplemental Table 1, field 14 of the World Health Organisation Trial Registration Data Set (Version 1.3.1). In the UK, a clinical RE diagnosis is based on electroclinical criteria defined by the International League Against Epilepsy (https://www.ilae.org/). Semiology and EEG need to be judged as concordant by a consultant neurophysiologist. Neuroimaging does not form part of UK standard care for RE. Eligibility criteria for trial sites include a Capacity and Capability assessment as advised for NHS site set-up by the UK HRA. The expected number of trial sites is 40 (England: 34, Scotland: 4, Wales: 1, Northern Ireland: 1). A list of trial sites can be obtained from the Trial Manager (see Supplemental Table 1).

Intervention

Participants will be allocated to trial arms (SC or SC + COSI) using minimisation (1:1 ratio). On allocation to SC + COSI, participants will receive an email with access details to COSI. COSI consists of a self-paced, novel, tailored, e-learning package for parents of children with epilepsy that incorporates evidence-based behavioural components. Table 3 provides a brief overview; detailed reports on the development, content, and evaluation of COSI have been published.[15 16] COSI is divided into 13 modules (1 screening for child-specific sleep problems to allow tailoring, 10 content, 1 additional resources, 1 initially hidden evaluation), of which three are compulsory (1 screening, 2 content). The non-compulsory modules are recommended based on screening outcome, but all modules are accessible, repeatable, and printable. The advice in COSI supports parents to

implement general prevention techniques (e.g. good sleep hygiene) and specific behavioural change techniques (e.g. bedtime fading) relevant to their child's sleep problems. Three months after first being given access to COSI, parents will be asked by email to complete a COSI evaluation module. At the end of a participant's trial timeline (6 months), access to COSI will be revoked. After the trial, all families (irrespective of trial allocation) have the option to receive the COSI content in electronic format via email.

Fidelity, adherence, retention, and acceptability

Fidelity (intervention delivery) will be monitored through e-analytics embedded in the COSI system (modules accessed, and time spent per module). Strategies to improve completion of COSI training in case of non-access include: (1) an automated text-reminder after two days; (2) an email reminder after four days; (3) a phone call from researchers who developed COSI (the Sleep Team) after six days. To improve adherence to the intervention, (1) all participants will receive a phone call from the Sleep Team six weeks after account creation; and (2) children will receive postcards with child-oriented activities (e.g. maze) at three time-points to welcome them to the trial (weeks 1–2), to stay in touch (weeks: 4–5), and to thank them for participating (weeks 4–8 post-trial). To encourage completion of the intervention evaluation, participants will receive: (1) an automated text-reminder after three days of non-completion, (2) and a phone call from the Sleep Team after eight days of non-completion. Fidelity (intervention implementation, acceptability, perceived helpfulness) will be captured jointly by the COSI evaluation module and the qualitative trial component.

Discontinuation, withdrawal, concomitant care, or interventions

Participants may discontinue the trial intervention or withdraw from the trial if (1) the parent/child withdraws consent/assent respectively; or (2) a change in the child's condition justifies discontinuation of treatment in their clinician's opinion. Trial site staff will record withdrawal with reason where provided in electronic Case Report Forms (eCRFs). Pseudo-anonymised data up to the time of consent withdrawal will be included in analyses in accordance with General Data Protection Regulation (GDPR)[41] under the UK Data Protection Act 2018[42] — the trial Data Controller relies on the legal bases of 'public interest' and 'research purposes'.

To avoid confounding and to minimise participant burden, co-enrolment into other clinical trials is discouraged. Where recruitment into another trial is considered appropriate, the trial coordinating centre will discuss enrolment with the Chief Investigator (CI). Participation in the Rolandic Epilepsy Genomewide Association International Study (REGAIN: https://childhoodepilepsy.org/research-studies/regain/) is complementary (same CI).

Table 2. GRIPP2- Short Form (SF)[21]: Guidance for Reporting Involvement of Patients and the Public in research

Section and topic	Item
**1: Aim**<br>Report the aim of PPI in the study	To contribute to and guide the CASTLE Sleep-E study:<br>• To ensure greater relevance and acceptability of the study and study procedures to children with epilepsy and their parents.<br>• To ensure the study is communicated to families and the public in an accessible way (e.g. recruitment, dissemination).
**2: Methods**<br>Provide a clear description of the methods used for PPI in the study	Two adults with experience of childhood epilepsy are co-applicants on the Changing Agendas on Sleep, Treatment and Learning in Childhood Epilepsy (CASTLE) Research Programme National Institute for Health and Care Research (NIHR) Award (https://tinyurl.com/ycyfkc63) and are an integral part of the CASTLE Advisory Panel (CAP). CAP is a dedicated Patient and Public Involvement (PPI) Advisory Panel that was recruited in 2017 through social media and epilepsy charities. CAP consists of 17 adults with experience of childhood epilepsy and five children with epilepsy (aged 6–15 years). CAP members are reimbursed for expenses and offered honorarium payments in acknowledgement of their contributions. Facilitated by a salaried Family Engagement Officer and the PPI lead (LB), CAP members have co-developed working practices (CAP Handbook: Adult version https://tinyurl.com/28u8jex4, child version: https://tinyurl.com/2p8d6bnx) and undertaken research training. CAP members communicate by video conference, telephone, email, social media, and face-to-face. CAP is represented in the Trial Steering Group (TSC, see Supplemental Table 2). CAP feedback and opinion is formally communicated to the CASTLE Sleep-E Trial Management Group (TMG, see Supplemental Table 2) via the CASTLE PPI lead (LB).
**3: Study results**<br>Outcomes—Report the results of PPI in the study, including both positive and negative outcomes	To date (at the recruitment stage of CASTLE Sleep-E), CAP has contributed to the following trial aspects:<br>**Initial funding application**<br>Two adults with experience of childhood epilepsy are co-applicants on the CASTLE Research Programme NIHR Award (https://tinyurl.com/ycyfkc63)<br>**Trial design**<br>• CAP strongly endorsed the investigation focus (sleep problems) and the focus on non-seizure related issues linked to epilepsy<br>• CAP tested and consulted on the trial intervention (CASTLE Online Sleep Intervention [COSI]) in respect to content, format, and acceptability (e.g. knowledge evaluation quiz was changed from compulsory to optional)<br>• CAP informed the selection of study questionnaires to ensure relevance to parents and children with epilepsy<br>• CAP guided trial design to ensure acceptability of processes (e.g. time, effort, schedule from a family perspective)<br>**Trial procedure**<br>• CAP led the development of a trial flowchart and clinician's guide (top tips for explaining the trial to families to aid recruitment)<br>• CAP guided data collection processes (assent/consent procedure, delivery of equipment, instructions, and packaging of Actigraphs and iPads)<br>• CAP guided the qualitative interview content and format (e.g. topics, question wording, length, delivery method and format)<br>**Trial materials**<br>• CAP informed the logo design (e.g. CASTLE website https://castlestudy.org.uk/) and name of the CASTLE Sleep-E trial

Section and topic	Item
	• CAP guided the development of all participant-facing trial materials including:<br>○ Information Sheets and Consent Forms<br>○ Child-friendly postcards to update and maintain interest in the trial<br>○ Wording of trial emails sent to participating families, strap lines for promotional materials (e.g. mugs and pens for trial sites)<br>**Dissemination**<br>• CAP informed liaison with stakeholders via social media and direct contact (charities, patient groups)<br>• CAP developed lay summaries for completed work as part of the CASTLE programme and helped ensure the CASTLE Sleep-E trial website (https://castlesleepetrial.org.uk/) is accessible to families<br>• CAP informed ongoing work to attract new CAP members
**4: Discussion and conclusions**<br>Outcomes—Comment on the extent to which PPI influenced the study overall. Describe positive and negative outcomes	• To date (recruitment stage of CASTLE Sleep-E), overall **positive outcomes** of CAP contributions to CASTLE Sleep-E have resulted in a trial design, procedure, materials, and dissemination that is likely to have greater appeal and relevance to parents of children affected by Rolandic epilepsy and to the children themselves. CAP has made the trial more family-focused, and enabled more direct public involvement (e.g. contact details of the Family Engagement Officer on the CASTLE Sleep-E webpage). This should increase the proportion of eligible patients to assent/consent to trial participation. Materials (including the trial intervention itself) and procedures should be more accessible and more feasible to complete for participants, which should positively affect adherence, compliance, and retention. Throughout their involvement, CAP contributions to the CASTLE programme have exceeded expectations, and taken on a greater, independent purpose (e.g. forming a support group via social media). The Coronavirus (COVID-19) pandemic meant that CAP's work had to move online, and whilst this has facilitated engagement between CAP members across the country, it made it more difficult for the children to join in some of the consultation exercises.
**5: Reflections/critical perspective**<br>Comment critically on the study, reflecting on the things that went well and those that did not, so others can learn from this experience	TBC (currently at recruitment stage of CASTLE Sleep-E)

For peer review only

Table 3. Content of the CASTLE Online Sleep Intervention (COSI)

Module	Module Name	Outline content	Compulsory or recommended
A	What is sleep and why is it important	Education about normal sleep physiology and processes	Compulsory
B	Sleep and seizures: a vicious cycle	Information about the relationship between sleep and seizures	Compulsory
C	Personalising this advice for your child	A sleep screening questionnaire to identify key areas of concern or problems around individual child sleep	Compulsory
D	Tips on sleep hygiene for everyone	General advice about key aspects of sleep hygiene	Recommended for all
E	Advanced sleep behaviour training	Introduction to principles of behavioural sleep interventions	Recommended for all
F	Learning difficulties, Attention Deficit Hyperactivity Disorder (ADHD), and Autism Spectrum Disorders	Advice for parents of children with other comorbid conditions	Recommended to parents who highlighted (in module C) their child may have comorbid conditions
G	Solving falling asleep problems	Sleep intervention options for typical falling asleep problems	Recommended to parents who highlighted (in module C) their child may have problems falling asleep
H	Solving difficult night wakings and early morning waking	Behavioural techniques to address typical night or early waking problems	Recommended to parents who highlight (in module C) their child may have problems with their sleep during night or early morning wakings
I	Solving night-time fears	Behavioural techniques to address typical night-time fears	Recommended to parents who highlight (in module C) their child may have problems with night-time fears
J	Sleep walking, sleep terrors, and nightmares	Information about different sleep behaviours, what causes them and how to identify and manage different conditions	Recommended to parents who highlight (in module C) their child may have problems with sleep walking, sleep terrors, and/or nightmares
K	Troubleshooting and maintaining good sleep	How to deal with common issues, such as the child being ill or parents disagreeing about how to manage sleep and advice about how to maintain any benefits	Recommended to all
L	Resources	Links to additional resources of support, information and advice relating to sleep	Recommended to all
M	Evaluation	Questionnaire in which parents are asked to report on their experiences of using COSI	Recommended to all

For peer review only

For peer review only

Outcomes and participant timeline

Outcomes are reported in Table 1 and were chosen collaboratively by children and young people with epilepsy and their parents, sleep- and epilepsy experts[8 17] in accordance with Core Outcome Measures in Effectiveness Trials (COMET) guidelines.[43] Psychometric properties and clinical relevance of outcomes are reported in Supplemental Table 3. Each participant will be followed up for 6 months. The participant timeline and estimated time requirement are respectively shown in Table 4 and Supplemental Table 4.

Sample size

The target sample size (110 children with RE, 55 per trial arm) was calculated based on achieving 90 % power to detect the minimal clinically important difference (MCID) in the primary clinical outcome (CSHQ) at 3 months after randomisation, accounting for 10 % expected attrition (non-parametric test with two-sided 5% significance level). MCID was defined based on an individual-focused anchor-based method,[44] that is, 'the smallest difference in outcome that patients perceive as beneficial and which mandates a change in patient management'.[45] The MCID value was based on the estimated reduction in total CSHQ score required for children with epilepsy (*M* = 48.25, *SD* = 8.91)[7] to fall at or below the diagnostic cut-off score of 41 for sleep disorders in paediatric populations.[28]

Recruitment, stopping guidelines, interim analyses

An Independent Data and Safety Monitoring Committee (IDSMC) will monitor recruitment and make recommendations to the Trial Steering Committee (TSC) concerning trial continuation, adjustments of recruitment methods, and follow-up optimisation (see Supplemental Table 2). A traffic light approach will determine trial continuation: (1) Green: Continue if at least 30 trial sites have opened and 22 participants have been randomised by end of month 6; (2) Amber: Implement additional recruitment strategies if 15–21 participants have been randomised by end of month 6; (3) Red: If recruitment is <15 participants by end of month 6, then stopping the trial early will be discussed with the TSC. Formal interim analyses of the accumulating data will not be performed.

Treatment allocation

Participants will be allocated with a 1:1 ratio to either SC or SC + COSI based on a computer generated adaptive restricted randomisation procedure that minimises differences between trial arms in variables likely to affect outcomes. Minimisation algorithm details are not published to avoid subversion of allocation sequence concealment, but include seizure frequency, AED, and sleep medication details. The allocation concealment mechanism is an online, central randomisation service implemented and maintained by the Liverpool Clinical Trial Centre (LCTC). The service will be accessed within four weeks of participant enrolment (once consent and eligibility confirmed, Participant ID issued, baseline dataset completed) by trained, authorised staff at trial sites. Randomisation will trigger allocation emails to the Trial Manager at LCTC and to the relevant trial site as well as enable COSI access for participants allocated to the intervention arm. Trial sites will notify the participant's General Practitioner (GP) of the treatment allocation by letter (electronic or hard copy, depending on preference).

For peer review only

Blinding

Only quantitative data analysts will be blinded (Participant IDs do not reveal treatment allocation). All other stakeholders (participants, parents, healthcare providers, data collectors, qualitative researchers) will be aware of the allocated intervention. Emergency unblinding procedures are therefore unnecessary.

For peer review only

Table 4. CASTLE SLEEP-E participant timeline and order of outcome completion.

	**T-4 weeks**[a] Consent and Baseline	**T0**[b] Randomisation	**T+3 months** Follow up visit	**T+6 months** Follow up visit
**Visit No**	**1**	**2**	**3**	**4**
Informed consent/assent	X			
Review of medical history and EEG[c] results	X			
Eligibility confirmation	X	X		
COVID-19 Screener	X		X	
Review of seizure occurrence		X	X	X
Hospital admissions		X	X	X
Demographics	X			
School absences		X	X	X
Check contact details for accuracy		X	X	X
Children's Sleep Habit Questionnaire[28]	X		X	X
SleepSuite[31] (iPad)	X		X	
WHO–Five Well-Being Index[33]	X			X
Health-Related Quality Of Life Measure for Children with Epilepsy[32]	X			X
Strengths and Difficulties Questionnaire[34]	X		X	X
CHU-9D[d]/CHU-9D proxy[38]	X		X	X
EQ-5D-Y/EQ-5D-Y proxy[37]	X		X	X
EQ-5D-5L[39]	X		X	X
Parenting Self Agency Measure[35]	X		X	X
Insomnia Severity Index[30]	X		X	X
Hospital Anxiety and Depression Scale[29]	X		X	X
Resource Use Questionnaire	X		X	X
Knowledge about Sleep in Childhood	X		X	
Randomisation<br>Standard Care (SC) or (SC + COSI[e])[16]		X		
Intervention arm only: COSI[16]		←	———	→
Actigraphy and sleep diary[36] (14 days)	X		X	
Confirm continuing trial participation			X	X
Assessment of Serious Adverse Events			X	X
Completion of Follow-up Case Report Form			X	X
Review of concomitant medications		X	X	X

[a] Up to four weeks flexibility between consent and randomisation to allow delivery of actigraph and iPad.
[b] Randomisation may be performed once two weeks of actigraphy and the minimum dataset are complete.
[c] Electro-encephalogram (EEG)
[d] Child Health Utility Index 9D (CHU-9D)
[e] CASTLE Online Sleep Intervention (COSI)

For peer review only

	T-4 weeks[a] Consent and Baseline	T0[b] Randomisation	T+3 months Follow up visit	T+6 months Follow up visit
**Visit No**	**1**	**2**	**3**	**4**
Qualitative Interview[f]			X	X

For peer review only

[f] Optional trial component: Consenting participants are interviewed within 3 weeks of follow up visits 3 and 4

Assent and consent

Potentially eligible children will be screened at trial centres by trained site staff. Screening outcome will be documented. Eligible children with interested parents will be invited to participate and provided with a Patient Information Sheet and Consent Form electronically and/or hard-copy (PISC, three versions: Parent, child [5–6 and 7–12 years]). Sufficient time will be allowed for discussion of the trial and the decision to assent/consent to trial entry and the optional qualitative component. Assent (children aged 7-12 years) and consent (parents) may be given face-to-face or remotely and will be electronically captured in a secure Consent Database managed by LCTC. Reasons for declining participation will be asked, but it will be made clear that children and parents do not have to provide a reason.

Data collection and management

Data collection will be carried out electronically except for Serious Adverse Events and Participant Transfer Forms (hard copy). At consent/assent, site staff will enter patient medical history (including electro-encephalogram), eligibility confirmation, COVID-19 screening, and demographics (see Table 4) into eCRFs stored in a secure Data Management System managed by LCTC. Trial participation will be added to the patient's medical records alongside their unique Participant ID.

Consent- and Contacts Databases are securely linked. The addition of a new participant will trigger email notifications to the parents containing access links to baseline assessments (see Table 4) and the Sleep Team who will access the Contacts Database to arrange the delivery of an iPad pre-configured by LCTC (optionally fitted with pre-paid SIMs), and two actigraphs with supporting documents. iPads (Generations 7–8, iOS 15.2 or 15.3) will be used to access the SleepSuite App, (V 1.4)[31], which assesses executive functions in child-friendly, interactive games (e.g. popping virtual bubbles with smiling children's faces). Access requires the Participant ID and is only possible at pre-specified trial time-points (see Table 4). Data is only stored on the iPad until the test-session completion, then automatically uploaded to a cloud-based server, and then securely downloaded for analyses by authorised LCTC staff. Families lacking other means of internet access can use iPads fitted with pre-paid SIMS to access other online trial materials (including email).

Actigraphs (Micro Motionlogger® Watch and Watchware Software V 1.99.17.4, Ambulatory Monitoring, Inc., NY: USA) will be used to collect 14 days of objective sleep data from child and parent. Concurrent sleep diaries (hard copies) will be completed by the parent with or without child input. At the end of the baseline period, actigraphs will be returned to the Sleep Team via pre-paid courier. The Sleep Team will download and securely store pseudo-anonymised (using Participant IDs) actigraphy data for pre-processing (manual selection of sleep periods cross-checked against sleep diaries) per night at participant-level. Summary variables (sleep latency, total sleep time and sleep efficiency) are then automatically calculated by actigraph software, manually collated, and securely transferred electronically to LCTC for trial-level analyses by the Trial Statistician.

Participants will be randomised to trial arms during a telephone/video call or clinic visit only *after* site staff have confirmed that baseline data (see Table 4) is complete, and eligibility, consent/assent, and contact details are still valid. Data collection will be repeated 3- and 6 months after randomisation, and iPads to LCTC via trial sites (see Table 4).

The Qualitative Research Team will access the Contacts Database to schedule audio-recorded interviews with children and parents who consented/assented to this optional trial

For peer review only

component. Interviews (audio- or audio-video) will take place remotely within 3 weeks of completion of other data collection at 3- and 6 months after randomisation. Parents and children will be interviewed together or separately as preferred. Parents and children will have the opportunity to think through their ideas prior to the interview (as proposed by parents and children from the CASTLE Advisory Panel). Children will be invited to complete activity booklets in advance of their interviews (the booklets will be mailed or emailed one week prior to their interview); the content they complete will support the interview. Parents will receive a list of proposed questions/topics. Children will be able to share the booklet with the Qualitative Team (e.g. screen or photograph sharing, verbal description).

The direct costs of health and personal social services, and indirect costs of productivity losses and school absenteeism will be collected using a Resource Use Questionnaire administered at baseline and during follow-up visits. Other data such as concomitant medications, study visits and Adverse Events will be collected using eCRFs. Trial participants' use of secondary care services will be collected from Patient-Level Information and Costing Systems (PLICS) data obtained from the finance departments of each recruiting hospital or from Hospital Episode Statistics (HES) data obtained from NHS Digital at the end of the trial. PLICS and HES data will be pseudo-anonymised and transferred securely to the trial health economists at Bangor University.

Data quality, security, and trial oversight

Reliability, validity, and clinical relevance of outcomes are reported in Supplemental Table 3. Processes to promote quality and security of collected data include general local training of site staff and research teams (Good Clinical Practice); and trial-specific training in the use of electronic forms and databases by LCTC. LCTC will request to see evidence of appropriate training and experience of all trial staff. Staff will be signed off as appropriately qualified by the CI. Electronic data capture provides several in-built validity and security checks (e.g. data type, range, and missingness checks in eCRFs, SleepSuite use/access restrictions). Some electronic and all hard-copy data will be repeat checked (e.g. eligibility, contact details). Data processing requiring more subjective judgement will be performed by minimum of two trained researchers on at least a subset of data (i.e. manually-assisted selection of actigraphy sleep period; thematic and content analysis of qualitative data).

Data will be processed and stored in accordance with GDPR under the UK Data Protection Act 2018. Central data monitoring will be performed by LCTC who will raise and resolve queries with site and research teams within the online system. The University of Liverpool is registered with the Information Commissioners Office. LCTC will receive trial participants' HES identifiers for secure transfer to the Health Economic team, who will access, securely store, and dispose of HES data in accordance with the Bangor University and NHS Digital Data Sharing Framework Contract.

Statistical methods

Statistical analyses of all but health economic and qualitative data will be performed by the Trial Statistician (LCTC) using SAS software, Version 9.4 or later. Intention-To-Treat (ITT) will be the main analysis strategy for primary and secondary outcomes (see Table 1 and Table 5). Minimisation variables (including seizure frequency, AED, and sleep medication details) will be adjusted for at baseline. Statistical significance will be set at the conventional two-sided 5 % level; clinical relevance will be based on previous research (see Supplemental Table 3). Point estimates with 95 % two-sided confidence intervals will be reported adjusted

For peer review only - http://bmjopen.bmj.com/site/about/guidelines.xhtml

and unadjusted for covariates. No multiplicity adjustments will be made (only one primary clinical outcome, uncorrected secondary outcome analyses).

Sensitivity analyses will be carried out if the amount of missing data is greater than 10 %. Multiple imputation will be used to assess the robustness of the analysis to missing primary outcome data. The multiple imputation method will follow published guidelines.[46] PROC MI in SAS will be used to generate 50 complete data sets. The imputation model will include all variables included in the primary outcome analysis model. The overall summary adjusted mean difference will be presented with 95 % confidence intervals, to assess the sensitivity of the primary analysis to missing data. All analyses will be reported in accordance with the Consolidated Standards of Reporting Trials Checklist (CONSORT)[47] and regardless of statistical significance.

Health economic evaluation

The economic analysis will be performed in accordance with a Health Economics Analysis Plan, and by the trial health economists at Bangor University. The primary analysis will adopt an NHS and Personal Social Services perspective and, based on Quality-Adjusted Life Years (QALYs) as a measure of health outcome, estimate the incremental cost-effectiveness ratio from an incremental analysis of the mean costs and QALYs for the intervention and control trial arms.[48] Data assumed to be missing at random will be imputed using multiple imputation by chained equations.[49]

Sensitivity analyses will be conducted to test whether, and to what extent, the incremental cost effectiveness ratio is sensitive to key assumptions in the analysis (e.g. unit prices, different utility estimates from CHU-9D[38] vs. EQ-5D-Y[37]). The joint uncertainty in costs and QALYs will be addressed through application of bootstrapping and estimation of cost-effectiveness acceptability curves.[50] Alternative scenarios considering a broader cost perspective (including indirect costs, such as school absences and loss of productivity, valued by reference to published sources), and a range of outcomes (including parental QALYs, measured using the EQ-5D-5L[51] and ISI[30 40]) will be conducted. Inclusion of spillover disutility[52] (impact on parents' utility) will be based on the NICE reference case specification[53] that all QALYs are of equal weight and calculated assuming additive effects. Health-economic findings will be reported according to the Consolidated Health Economic Evaluation Reporting Standards (CHEERS).[54]

Qualitative component

Child and parent interviews will be analysed by the Qualitative Research Team using an interpretive, reflexive, and conceptual analytical approach. Audio-recordings of interviews will be transcribed and thematically analysed in discrete sets (e.g. intervention/control, child/parent, engagement/lack of engagement with intervention, types of decision-making, different responses/experiences). Parent and child transcripts will first be analysed separately, and then as dyads. All data will be used for synthesis. Thematic and content analyses will be used for child activity booklets (text and images). Qualitative and selected quantitative data (e.g. anxiety measures, actigraphy data) will be compared, as appropriate.

For peer review only

Table 5. Analysis plan for outcome variables in CASTLE Sleep-E. Further analyses details are reported in-text.

**Outcome type**	**Specific measurement variable**	**Hypothesis**	**Method of analysis**
Primary			
Clinical	Children's Sleep Habits Questionnaire[28]	Total score lower in intervention arm at 3 months	Linear mixed effect regression:<br>• Fixed effects: Intervention (binary)<br>• Random effects: Trial site (categorical)<br>• Co-variates:<br>○ Baseline score<br>○ Use of sleep medication (binary)
Health economic	Cost[a] per quality-adjusted life year gained	Not applicable (health economic evaluation)	Cost-effectiveness (utility) analysis
Secondary			
Clinical	Children's Sleep Habits Questionnaire[28]	Total score lower in intervention arm at 6 months	Linear mixed effect regression (as before)
Clinical	Seizure-free period	Time to first seizure (days) differs between trial arms at 3 and 6 months	Survival analyses<br>• Kaplan-Meier curves by trial arm<br>• Cox proportional hazards regression (if applicable)<br>○ Co-variates:<br>▪ Use of sleep medication (binary)<br>▪ Trial site (categorical)
Clinical	Time to 6-months seizure remission from randomisation (days)	Time to 6-months seizure remission (days) differs between trial arms at 6 months	Survival analyses (as before)
Clinical	• Knowledge about Sleep in Childhood<br>• Actigraphy[36] (2-week average):<br>○ Total sleep time<br>○ Sleep latency<br>○ Sleep efficiency	Total score differs between trial arms at 3 months	Linear mixed effect regression (as before)
Clinical	• Hospital Anxiety and Depression Scale[29]<br>• Insomnia severity index[30]	Total score lower in intervention arm at 3 and 6 months	Linear mixed effect regression (as before)
Clinical	• Sickness-related school absences	Total days differs between trial arms at 3 and 6 months	Poisson mixed-effects regression

[a] **Perspective**: NHS and PSS perspective; **Alternative perspective**: Societal (Indirect and direct non-medical costs)

For peer review only - http://bmjopen.bmj.com/site/about/guidelines.xhtml

Outcome type	Specific measurement variable	Hypothesis	Method of analysis
Clinical	• Health-Related Quality Of Life Measure for Children with Epilepsy[32]<br>• World Health Organisation – Five Well-Being Index[33]	Total score differs between trial arms at 6 months	Linear mixed effect regression (as before)
Clinical	• SleepSuite[31]: Animal task<br>• SleepSuite: Bubble task<br>○ Shape detection<br>○ Emotion detection<br>○ Gender detection<br>• SleepSuite: Maze task	Executive function, reaction time, and variability differ between trials arm at 3 months	• Poisson/zero-inflated negative binomial regression (depending on presence of overdispersion)<br>• 2 x 2 multi-variate repeated-measures Analysis of Variance (ANOVA)<br>Factors: Time (PM/AM) x Intervention (Pre/Post)<br>Fitted per detection task (Shape, Emotion, Gender)<br>• Linear mixed effect regression (as before)
Clinical	• Strengths and Difficulties[34] Questionnaire<br>• Parenting Self Agency Measure[35]	Total score differs between trial arms at 3 and 6 months	Linear mixed effect regression (as before)
Qualitative	Trial experience[b]	Not applicable (inductive)	Thematic analysis (interpretive, reflexive, and conceptual analytical approach)<br>• Discrete sets: Intervention/Control, Child/parent, Engagement with intervention/lack thereof, Decision making types, Responses/experiences<br>• Separately for child and parent, then jointly (dyad)<br>• Comparisons to selective objective data as emerging from analysis (e.g. Anxiety measures, Actigraphy)

[b] **Source data for trial experience**: Qualitative interviews (parents and children individually and as dyad), activity booklets (children only)

For peer review only - http://bmjopen.bmj.com/site/about/guidelines.xhtml

Harms

A flowchart of Adverse Event (AE) reporting requirements is shown in Supplemental Figure 1. Harms severity and causality will be graded by the investigator responsible for the care of the participant based on categories shown in Supplemental Table 5. If any doubt about causality exists, the local investigator should inform LCTC who will notify the CI. In case of discrepant views, the Research Ethics Committee (REC) will be informed of both views. Seriousness and expectedness of AEs will be defined based on International Council for Harmonisation of Technical Requirements for Pharmaceuticals for Human Use Definitions and Standards for Expedited Reporting (ICH E2A, ref: CPMP/ICH/377/95). Expectedness will be assessed by the CI. The only expected AEs in CASTLE Sleep-E are mild and transient worsening of sleep behaviours targeted by the trial intervention. Safety data will be quality-checked by a statistician not otherwise involved in the trial. Safety analysis will include all patients randomised and starting treatment and be presented descriptively split by treatment arm.

Auditing

The CI will ensure that the trial team conducts monitoring activities of sufficient quality and quantity (e.g. protocol adherence, consent/assent, data quality). The Sponsor will delegate monitoring duties and activities to LCTC. The CI and LCTC will inform the Sponsor of any concerns. Auditing does not meet the National Institute for Health and Care Research (NIHR) or SPIRIT Statement definitions of independence[19 55] as auditors (LCTC and CI) are part of the trial team.

Protocol amendments

Substantive protocol amendments will be notified to HRA via the UK's Integrated Research Application System (IRAS). Trial sites will receive an amendment pack of HRA- and REC-approved changes and unless an objection is received within 35 days, the trial will continue at site with a GO LIVE email.

Ancillary and post-trial care

King's College London (KCL) holds insurance against claims from participants for harm caused by their participation in this clinical study; compensation can be claimed in case of KCL negligence.

Ethics and dissemination

The CASTLE Sleep-E protocol was approved by the HRA East Midlands – Nottingham 1 REC, reference: 21/EM/0205. Trial results will be disseminated to scientific audiences in peer-reviewed publications and conferences, and — with the help of the CASTLE Advisory Panel (parent and child experts-by-experience), relevant charities (e.g. Epilepsy Action, Epilepsy Society and Cerebra) and professional groups (e.g. Royal College of Paediatrics and Child Health, Epilepsy Specialist Nurses Association) — as plain language summaries to families, other professional groups, managers, commissioners, and policy makers. Pseudo-anonymised Individual Patient Data and associated documentation (e.g. protocol, statistical analysis plan, annotated blank Case Report Form) will be made available after dissemination on reasonable request.

For peer review only - http://bmjopen.bmj.com/site/about/guidelines.xhtml

Registration details

ISRCTN registry (Trial ID: ISRCTN13202325, prospective registration 09/September/2021). The World Health Organisation Trial Registration Data Set (Version 1.3.1) for CASTLE Sleep-E is shown in Supplemental Table 1.

For peer review only

Author Statement

Contributorship (alphabetic surname order)
PG, DKP (Chief Investigators); CAP, CTS, HH, LW, BC, CM, DH, and LB (Co-Investigators) conceived the study and are award holders. Topic expertise for the core outcome set development was provided by CAP, LB, BC, AC, HC, PG, DH, CM, DKP, CTS, and PRW. Epilepsy expertise-by-experience is provided by CAP. Topic expertise for epilepsy is provided by DKP. Topic expertise for the health economic evaluation is provided by WASH, DH, and EW. Topic expertise for intervention development was provided by GC, PG, HH, DKP, and LW. Topic expertise for Patient and Public Involvement (Advisory Panel and Family Engagement) is provided by CAP, AR-S, LB, BC, and CM. Responsibility for the selection of Patient-Reported Outcomes lay with CM. Responsibility for Programme management lies with AC. Topic expertise for qualitative research components is provided by CAP, LB, BC, and HS. Topic expertise for sleep is provided by GC, PG, HH, and LW. Topic expertise for Statistical analyses is provided by CTS, VW, and LWh. Responsibility for trial management lies with NA-N, CS, and LS-E. All authors contributed to the design and refinement of the study protocol. The protocol manuscript was written by KCD (including supplemental materials but excluding Figure 1 and Patient Information Sheet and Consent Forms). Authors in the Trial Management Group (TMG) had the opportunity to provide feedback twice (initial and final draft); non-TMG authors had the opportunity to provide feedback once (final draft). Provided feedback was incorporated. The final manuscript was approved for publication by all authors. GRIPP2 content was checked for accuracy by LB. Sponsor name and contact information are provided in Supplemental Table 1. Details of trial committees and other groups and individuals overseeing the trial are listed in Supplemental Table 2. Trial site Principal Investigators will be listed alphabetically in resulting publications as members of the CASTLE Sleep-E Consortium in the Acknowledgements section. There has not been and will not be any use of hired writers.

Funding. This work is supported by the National Institute for Health and Care Research (NIHR), award number RP-PG-0615-20007. HH was supported by a National Health and Medical Research Council (NHMRC, Australia) Practitioner Fellowship (1136222). HH's institute — the Murdoch Children's Research Institute (MCRI, Australia) — is supported by the Victorian Government's Operational Infrastructure Support Program (no award/grant number).

Disclaimer. To avoid potential bias, neither the funder nor the sponsor of this trial has any role in or authority over the design, execution, analyses, interpretation of data, or result dissemination.

Competing interests. None declared.

Patient consent for publication. Not applicable.

Provenance and peer review. Not commissioned, externally peer reviewed.

Supplemental material. This content has been supplied by the author(s). It has not been vetted by BMJ Publishing Group Limited (BMJ) and may not have been peer-reviewed. Any opinions or recommendations discussed are solely those of the author(s) and are not endorsed by BMJ. BMJ disclaims all liability and responsibility arising from any reliance placed on the content. Where the content includes any translated material, BMJ does not warrant the accuracy and reliability

For peer review only - http://bmjopen.bmj.com/site/about/guidelines.xhtml

of the translations (including but not limited to local regulations, clinical guidelines, terminology, drug names and drug dosages), and is not responsible for any error and/or omissions arising from translation and adaptation or otherwise.

Licence statement. I, the Submitting Author has the right to grant and does grant on behalf of all authors of the Work (as defined in the below author licence), an exclusive licence and/or a non-exclusive licence for contributions from authors who are: i) UK Crown employees; ii) where BMJ has agreed a CC-BY licence shall apply, and/or iii) in accordance with the terms applicable for US Federal Government officers or employees acting as part of their official duties; on a worldwide, perpetual, irrevocable, royalty-free basis to BMJ Publishing Group Ltd ("BMJ") its licensees and where the relevant Journal is co-owned by BMJ to the co-owners of the Journal, to publish the Work in BMJ Open and any other BMJ products and to exploit all rights, as set out in our licence.

The Submitting Author accepts and understands that any supply made under these terms is made by BMJ to the Submitting Author unless you are acting as an employee on behalf of your employer or a postgraduate student of an affiliated institution which is paying any applicable article publishing charge ("APC") for Open Access articles. Where the Submitting Author wishes to make the Work available on an Open Access basis (and intends to pay the relevant APC), the terms of reuse of such Open Access shall be governed by a Creative Commons licence – details of these licences and which Creative Commons licence will apply to this Work are set out in our licence referred to above.

For peer review only

References

1. Collaborators GBDE. Global, regional, and national burden of epilepsy, 1990-2016: a systematic analysis for the Global Burden of Disease Study 2016. Lancet Neurol 2019;**18**(4):357-75 doi: 10.1016/S1474-4422(18)30454-X [published Online First: 20190214].
2. Olusanya BO, Wright SM, Nair MKC, et al. Global Burden of Childhood Epilepsy, Intellectual Disability, and Sensory Impairments. Pediatrics 2020;**146**(1) doi: 10.1542/peds.2019-2623.
3. Li Q, Westover MB, Zhang R, et al. Computational Evidence for a Competitive Thalamocortical Model of Spikes and Spindle Activity in Rolandic Epilepsy. Front Comput Neurosci 2021;**15**:680549 doi: 10.3389/fncom.2021.680549 [published Online First: 20210618].
4. Stephen J, Weir CJ, Chin RF. Temporal trends in incidence of Rolandic epilepsy, prevalence of comorbidities and prescribing trends: birth cohort study. Arch Dis Child 2020;**105**(6):569-74 doi: 10.1136/archdischild-2019-318212 [published Online First: 20200114].
5. Ross EE, Stoyell SM, Kramer MA, et al. The natural history of seizures and neuropsychiatric symptoms in childhood epilepsy with centrotemporal spikes (CECTS). Epilepsy Behav 2020;**103**(Pt A):106437 doi: 10.1016/j.yebeh.2019.07.038 [published Online First: 20191020].
6. Petropoulos M-C, Bonaiuto K, Currier J, et al. Practical aspects of childhood epilepsy. BMJ 2019;**367**:l6096 doi: 10.1136/bmj.l6096.
7. Larson AM, Ryther RC, Jennesson M, et al. Impact of pediatric epilepsy on sleep patterns and behaviors in children and parents. Epilepsia 2012;**53**(7):1162-9 doi: 10.1111/j.1528-1167.2012.03515.x [published Online First: 20120517].
8. Crudgington H, Rogers M, Bray L, et al. Core Health Outcomes in Childhood Epilepsy (CHOICE): Development of a core outcome set using systematic review methods and a Delphi survey consensus. Epilepsia 2019;**60**(5):857-71 doi: 10.1111/epi.14735 [published Online First: 20190425].
9. Gibbon FM, Maccormac E, Gringras P. Sleep and epilepsy: unfortunate bedfellows. Archives of Disease in Childhood 2019;**104**(2):189-92 doi: 10.1136/archdischild-2017-313421.
10. Phillips NL, Moore T, Teng A, et al. Behavioral interventions for sleep disturbances in children with neurological and neurodevelopmental disorders: a systematic review and meta-analysis of randomized controlled trials. Sleep 2020;**43**(9) doi: 10.1093/sleep/zsaa040.
11. Tsai S-Y, Lee W-T, Lee C-C, et al. Behavioral-educational sleep interventions for pediatric epilepsy: a randomized controlled trial. Sleep 2020;**43**(1):zsz211 doi: 10.1093/sleep/zsz211.
12. Ahorsu DK, Lin CY, Imani V, et al. Testing an app-based intervention to improve insomnia in patients with epilepsy: A randomized controlled trial. Epilepsy Behav 2020;**112**:107371 doi: 10.1016/j.yebeh.2020.107371 [published Online First: 20200827].
13. Condon HE, Maurer LF, Kyle SD. Reporting of adverse events in cognitive behavioural therapy for insomnia: A systematic examination of randomised controlled trials. Sleep Medicine Reviews 2021;**56**:101412 doi: https://doi.org/10.1016/j.smrv.2020.101412.
14. Sunnhed R, Hesser H, Andersson G, et al. Comparing internet-delivered cognitive therapy and behavior therapy with telephone support for insomnia disorder: a randomized controlled trial. Sleep 2019;**43**(2) doi: 10.1093/sleep/zsz245.
15. Wiggs L, Cook G, Hiscock H, et al. Development and Evaluation of the CASTLE Trial Online Sleep Intervention for Parents of Children with Epilepsy. Frontiers in Psychology 2021;**12** doi: 10.3389/fpsyg.2021.679804.
16. Cook G, Gringras P, Hiscock H, et al. A Qualitative Investigation Into What Parents Want From an Online Behavioural Sleep Intervention for Children With Epilepsy. Frontiers in Psychology 2021;**12** doi: 10.3389/fpsyg.2021.628605.
17. Morris C, Dunkley C, Gibbon FM, et al. Core Health Outcomes In Childhood Epilepsy (CHOICE): protocol for the selection of a core outcome set. Trials 2017;**18**(1):572 doi: 10.1186/s13063-017-2323-7.
18. Chan A-W, Tetzlaff JM, Altman DG, et al. SPIRIT 2013 Statement: Defining Standard Protocol Items for Clinical Trials. Annals of Internal Medicine 2013;**158**(3):200-07 doi: 10.7326/0003-4819-158-3-201302050-00583.
19. Chan A-W, Tetzlaff JM, Gøtzsche PC, et al. SPIRIT 2013 explanation and elaboration: guidance for protocols of clinical trials. BMJ : British Medical Journal 2013;**346**:e7586 doi: 10.1136/bmj.e7586.
20. Calvert M, King M, Mercieca-Bebber R, et al. SPIRIT-PRO Extension explanation and elaboration: guidelines for inclusion of patient-reported outcomes in protocols of clinical trials. BMJ Open 2021;**11**(6):e045105 doi: 10.1136/bmjopen-2020-045105.

For peer review only - http://bmjopen.bmj.com/site/about/guidelines.xhtml

21. Staniszewska S, Brett J, Simera I, et al. GRIPP2 reporting checklists: tools to improve reporting of patient and public involvement in research. BMJ 2017;**358**:j3453 doi: 10.1136/bmj.j3453.
22. Freedland KE, Mohr DC, Davidson KW, et al. Usual and unusual care: existing practice control groups in randomized controlled trials of behavioral interventions. Psychosom Med 2011;**73**(4):323-35 doi: 10.1097/PSY.0b013e318218e1fb [published Online First: 2011/05/02].
23. Thompson BT, Schoenfeld D. Usual care as the control group in clinical trials of nonpharmacologic interventions. Proc Am Thorac Soc 2007;**4**(7):577-82 doi: 10.1513/pats.200706-072JK.
24. Zuidgeest MGP, Welsing PMJ, van Thiel GJMW, et al. Series: Pragmatic trials and real world evidence: Paper 5. Usual care and real life comparators. Journal of Clinical Epidemiology 2017;**90**:92-98 doi: https://doi.org/10.1016/j.jclinepi.2017.07.001.
25. National Institute for Health and Care Excellence (NICE). Epilepsies: diagnosis and management (CG137). London: NICE, 2012.
26. Scottish Intercollegiate Guidelines Network (SIGN). Epilepsies in children and young people: investigative procedures and management (SIGN 159). Edinburgh: SIGN, 2020.
27. Health and Social Care Board (HSCB) Northern Ireland. Recommended Clinical Guidelines (CGs). Secondary Recommended Clinical Guidelines (CGs) 19/Jan/2022 2022. http://www.hscboard.hscni.net/nice/recommended-clinical-guidelines-cgs/.
28. Owens JA, Spirito A, McGuinn M. The Children's Sleep Habits Questionnaire (CSHQ): psychometric properties of a survey instrument for school-aged children. Sleep 2000;**23**(8):1043-51.
29. Zigmond AS, Snaith RP. The hospital anxiety and depression scale. Acta Psychiatr Scand 1983;**67**(6):361-70 doi: 10.1111/j.1600-0447.1983.tb09716.x.
30. Morin CM, Belleville G, Bélanger L, et al. The Insomnia Severity Index: psychometric indicators to detect insomnia cases and evaluate treatment response. Sleep 2011;**34**(5):601-08 doi: 10.1093/sleep/34.5.601.
31. Colonna A, Smith AB, Smith S, et al. The Effects of Sleep on Emotional Target Detection Performance: A Novel iPad-Based Pediatric Game. Frontiers in psychology 2018;**9**:241-41 doi: 10.3389/fpsyg.2018.00241.
32. Ronen GM, Streiner DL, Rosenbaum P, et al. Health-related quality of life in children with epilepsy: development and validation of self-report and parent proxy measures. Epilepsia 2003;**44**(4):598-612 doi: 10.1046/j.1528-1157.2003.46302.x.
33. Allgaier A-K, Pietsch K, Frühe B, et al. Depression in pediatric care: is the WHO-Five Well-Being Index a valid screening instrument for children and adolescents? General Hospital Psychiatry 2012;**34**(3):234-41 doi: https://doi.org/10.1016/j.genhosppsych.2012.01.007.
34. Goodman R. Psychometric Properties of the Strengths and Difficulties Questionnaire. Journal of the American Academy of Child & Adolescent Psychiatry 2001;**40**(11):1337-45 doi: 10.1097/00004583-200111000-00015.
35. Dumka LE, Stoerzinger HD, Jackson KM, et al. Examination of the Cross-Cultural and Cross-Language Equivalence of the Parenting Self-Agency Measure. Family Relations 1996;**45**(2):216-22 doi: 10.2307/585293.
36. Sadaka Y, Sadeh A, Bradbury L, et al. Validation of actigraphy with continuous video-electroencephalography in children with epilepsy. Sleep Med 2014;**15**(9):1075-81 doi: 10.1016/j.sleep.2014.04.021 [published Online First: 20140602].
37. Wille N, Badia X, Bonsel G, et al. Development of the EQ-5D-Y: a child-friendly version of the EQ-5D. Qual Life Res 2010;**19**(6):875-86 doi: 10.1007/s11136-010-9648-y [published Online First: 20100420].
38. Stevens K. Valuation of the Child Health Utility 9D Index. Pharmacoeconomics 2012;**30**(8):729-47 doi: 10.2165/11599120-000000000-00000.
39. Hernández-Alava M, Pudney S. Mapping between EQ-5D-3L and EQ-5D-5L: A survey experiment on the validity of multi-instrument data. Health Econ 2022;**31**(6):923-39 doi: 10.1002/hec.4487 [published Online First: 20220228].
40. Gu NY, Botteman MF, Ji X, et al. Mapping of the Insomnia Severity Index and other sleep measures to EuroQol EQ-5D health state utilities. Health Qual Life Outcomes 2011;**9**:119 doi: 10.1186/1477-7525-9-119 [published Online First: 20111230].
41. The European Parlament and the Council of the European Union. EU General Data Protection Regulation (GDPR). Secondary EU General Data Protection Regulation (GDPR) 2018. https://eur-lex.europa.eu/legal-content/EN/TXT/?uri=CELEX%3A02016R0679-20160504&qid=1532348683434.
42. The Government of the United Kingdom. UK Data Protection Act 2018. Secondary UK Data Protection Act 2018 2018. https://www.legislation.gov.uk/ukpga/2018/12/contents/enacted.

For peer review only - http://bmjopen.bmj.com/site/about/guidelines.xhtml

43. Williamson PR, Altman DG, Bagley H, et al. The COMET Handbook: version 1.0. Trials 2017;**18**(3):280 doi: 10.1186/s13063-017-1978-4.
44. Kallogjeri D, Spitznagel EL, Jr, Piccirillo JF. Importance of Defining and Interpreting a Clinically Meaningful Difference in Clinical Research. JAMA Otolaryngology–Head & Neck Surgery 2020;**146**(2):101-02 doi: 10.1001/jamaoto.2019.3744.
45. Jaeschke R, Singer J, Guyatt GH. Measurement of health status. Ascertaining the minimal clinically important difference. Control Clin Trials 1989;**10**(4):407-15 doi: 10.1016/0197-2456(89)90005-6.
46. Jakobsen JC, Gluud C, Wetterslev J, et al. When and how should multiple imputation be used for handling missing data in randomised clinical trials – a practical guide with flowcharts. BMC Medical Research Methodology 2017;**17**(1):162 doi: 10.1186/s12874-017-0442-1.
47. Schulz KF, Altman DG, Moher D. CONSORT 2010 statement: Updated guidelines for reporting parallel group randomised trials. J Pharmacol Pharmacother 2010;**1**(2):100-07 doi: 10.4103/0976-500X.72352.
48. Petrou S, Gray A. Economic evaluation alongside randomised controlled trials: design, conduct, analysis, and reporting. BMJ 2011;**342**:d1548 doi: 10.1136/bmj.d1548 [published Online First: 20110407].
49. Manca A, Palmer S. Handling missing data in patient-level cost-effectiveness analysis alongside randomised clinical trials. Applied Health Economics and Health Policy 2005;**4**(2):65-75 doi: 10.2165/00148365-200504020-00001.
50. Fenwick E, Claxton K, Sculpher M. Representing uncertainty: the role of cost-effectiveness acceptability curves. Health Econ 2001;**10**(8):779-87 doi: 10.1002/hec.635.
51. Bergmann M, Tschiderer L, Stefani A, et al. Sleep quality and daytime sleepiness in epilepsy: Systematic review and meta-analysis of 25 studies including 8,196 individuals. Sleep Medicine Reviews 2021;**57**:101466 doi: https://doi.org/10.1016/j.smrv.2021.101466.
52. Wittenberg E, James LP, Prosser LA. Spillover Effects on Caregivers' and Family Members' Utility: A Systematic Review of the Literature. PharmacoEconomics 2019;**37**(4):475-99 doi: 10.1007/s40273-019-00768-7.
53. National Institute for Health and Care Excellence (NICE). NICE health technology evaluations: the manual. Process and methods [PMG36]. London: NICE, 2022.
54. Husereau D, Drummond M, Petrou S, et al. Consolidated Health Economic Evaluation Reporting Standards (CHEERS) statement. BMJ : British Medical Journal 2013;**346**:f1049 doi: 10.1136/bmj.f1049.
55. National Institute for Health and Care Excellence (NICE). Clinical Trials Toolkit: Audit. Audit. London: NICE, 2022.

For peer review only - http://bmjopen.bmj.com/site/about/guidelines.xhtml

Supplemental Table 1. World Health Organization Trial Registration Data Set (Version 1.3.1) for CASTLE Sleep-E

Data category	Information
1. Primary registry and trial identifying number	ISRCTN: ISRCTN13202325
2. Date of registration in primary registry	09/September/2021
3. Secondary identifying numbers	CPMS 50413<br>RP-PG-0615-20007<br>IRAS 289580<br>21/EM/0205
4. Source(s) of monetary or material support	National Institute for Health and Care Research (NIHR)
5. Primary sponsor	Ms Jasmine Palmer<br>Research & Innovation Operational Manager<br>King's College Hospital NHS Foundation Trust<br>The Research & Innovation Office<br>First Floor, Coldharbour Works<br>245a Coldharbour Lane, Brixton<br>London SW9 8RR<br>jasmine.palmer1@nhs.net<br>+44 (0) 7790 950 219
6. Secondary sponsor(s)	Professor Reza Razavi<br>Director of Research Management &<br>Director of Administration (Health Schools)<br>Room 5.31<br>James Clerk Maxwell Building<br>57 Waterloo Road<br>London<br>SE1 8WA<br>reza.razavi@kcl.ac.uk<br>+44 (0)20 7848 3224
7. Contact for public queries	Trial Manager: Lucy Stibbs-Eaton<br>Liverpool Clinical Trials Centre<br>University of Liverpool<br>Liverpool<br>L69 3BX<br>LCTC@liverpool.ac.uk<br>+44 (0)151 795 8751
8. Contact for scientific queries	Professor Deb Pal<br>Professor of Paediatric Epilepsy<br>Maurice Wohl Clinical Neuroscience Institute<br>King's College London<br>5 Cutcombe Road<br>London<br>SE5 9RX<br>deb.pal@kcl.ac.uk<br>+44 (0) 207 848 5762
9. Public title	A trial comparing the effectiveness of an online sleep behavioural intervention versus standard care in children with rolandic epilepsy
10. Scientific title	Changing Agendas on Sleep, Treatment and Learning in Epilepsy (CASTLE) Sleep-E: A randomised controlled trial comparing an online behavioural sleep intervention with standard care in children with Rolandic epilepsy

For peer review only - http://bmjopen.bmj.com/site/about/guidelines.xhtml

Data category	Information
11. Countries of recruitment	England<br>Scotland<br>Wales<br>Northern Ireland
12. Health condition(s) or problem(s) studied	Sleep problems in Rolandic epilepsy also known as childhood epilepsy with centro-temporal spikes
13. Intervention(s)	**Intervention arm (SC + COSI)**: Novel, tailored, parent-led CASTLE Online Sleep Intervention (COSI) that incorporates evidence-based behavioural components. Delivered by parents to enrolled children with Rolandic epilepsy in their own homes after completion of self-paced online training. Standard care (SC) is augmented with the CASTLE Online Sleep Intervention (COSI).<br><br>**Active control arm (SC)**: UK National Health Service standard care (SC) for children with Rolandic epilepsy, which consists of a comprehensive care plan with the option of pharmacological treatment with anti-epileptic drugs (first-line mono-therapy with lamotrigine, levetiracetam, oxcarbazepine [girls and boys], carbamazepine or sodium valproate [both boys only]).
14. Key inclusion and exclusion criteria	Inclusion criteria<br>Main CASTLE Sleep-E study<br>1. Children diagnosed with RE/CECTS (see International League Against Epilepsy Diagnostic Manual at https://www.epilepsydiagnosis.org/syndrome/ects-overview.html)<br>2. EEG showing focal sharp waves with normal background (see International League Against Epilepsy Diagnostic Manual at https://www.epilepsydiagnosis.org/syndrome/ects-eeg.html)<br>3. Aged 5 to <13 years at the time of randomisation<br>4. Parent/Carer reported child sleep problem as defined by mild, moderate or severe score on Hiscock Australian global sleep question (Poor sleeper defined by caregiver responding 'Mild', 'Moderate' or 'Severe' to "Over the last 2 weeks, how much of a problem has your child's sleep been?")<br>5. Documented informed consent received from a person with parental responsibility<br>6. Family have an email address and mobile phone<br>7. Parent and child are to have a good enough understanding of the English language to read and answer study questionnaires<br>Qualitative component<br>1. Consent of care giver to participate and for their child to participate (optional item on main trial consent form)<br>2. Children need to be >=7 years of age<br><br>Exclusion criterion<br>1. Children with moderate/severe learning disability
15. Study type	Interventional<br>• Allocation: Minimisation using a bespoke LCTC system<br>Allocation concealment: Central web-interface<br>Sequence generation: Randomised, 1:1 ratio<br>Intervention model: Parallel assignment<br>• Blinding<br>Child, parent, healthcare providers, data collectors, qualitative researchers: None (open label)<br>Quantitative data analysts: Blinded<br>• Primary purpose: Clinical- and cost-effectiveness, process evaluation (qualitative trial component, COSI e-analytics and evaluation module)<br>• Phase: III (behavioural intervention)

For peer review only - http://bmjopen.bmj.com/site/about/guidelines.xhtml

Data category	Information
16. Date of first enrolment	24/June/2022
17. Target sample size	110 (55 children per arm)<br>Calculation based on:<br>• Achieving 90 % statistical power to detect Minimal Clinically Meaningful Difference in primary outcome<br>• 10 % expected attrition
18. Recruitment status	Recruiting<br>• First trial site opened: 12/May/2022<br>• First recruitment: 30/August/2022
19. Primary outcome(s)	• Clinical: Children's Sleep Habits Questionnaire at 3 months<br>• Health economic: Cost-effectiveness of the intervention over 6 months after randomisation, measured in terms of incremental cost per quality-adjusted life year gained (Child Health Utility instrument or EQ-5D-Y) from the perspective of the National Health Services and Personal Social Services in the UK.
20. Key secondary outcome(s)	• Clinical Outcome: Sleep problem reduction<br>Metric/method: Children's Sleep Habits Questionnaire<br>Timepoint: 6 months<br>• Clinical Outcome: Seizure frequency reduction<br>Metric/method: Time to first seizure (days)<br>Timepoint: 3 months, 6 months
21. Ethics Review	• Status: Approved<br>• Approval reference: 21/EM/0205<br>• Health Research Authority<br>East Midlands – Nottingham 1 Research Ethics Committee<br>Chair: Mr Paul Hamilton<br>+44 (0) 207 104 8115 or +44 (0) 207 104 8283<br>nottingham1.rec@hra.nhs.uk
22. Completion date	31/July/2023
23. Summary results	TBC
24. Individual patient data (IPD) sharing statement	• Plan to share IPD: Yes<br>• Plan description: At the end of the trial, after the primary results have been published, the pseudo-anonymised Individual Patient Data and associated documentation (e.g. protocol, statistical analysis plan, annotated blank case report form) will be prepared to be shared with external researchers on reasonable request.
25. Protocol version and date	• Internal protocol: V4.0, 08/December/2021<br>• Manuscript for protocol publication: V3.2, 20/December/2022

For peer review only - http://bmjopen.bmj.com/site/about/guidelines.xhtml

Supplemental Table 2. Composition, roles and responsibilities of the Trial Management Group, Programme Steering Committee, and Independent Data and Safety Monitoring Committee for CASTLE Sleep-E.

Role	Name (Initials)	Affiliation
**Trial management Group (TMG)**<br>**Responsibilities**: Day-to-day running and management of the trial.<br>**Meeting frequency**: Bi-weekly to three-monthly, depending on trial stage.		
1. King's College Hospital Sponsor Representative	Jasmine Palmer	King's College Hospital NHS Foundation Trust, UK
2. Chief Investigator	Deb K. Pal	King's College London, UK
3. Co-Chief Investigator	Paul Gringras	Evelina London Children's Hospital, UK
4. Co-Investigator<br>Public and Patient Involvement Lead	Lucy Bray	Edge Hill University, UK
5. Co-Investigator<br>Qualitative Research Lead<br>Public and Patient Involvement Co-Lead	Bernie Carter	Edge Hill University, UK
6. Co-Investigator<br>Health Economics Lead	Dyfrig Hughes	Bangor University, UK
7. Co-Investigator<br>Patient Reported Outcome Lead<br>Public and Patient Involvement Co-Lead	Christopher Morris	University of Exeter, UK
8. Co-Investigator<br>Lead Statistician	Catrin Tudur Smith	University of Liverpool, UK
9. Co-Investigator<br>Intervention Development Lead	Luci Wiggs	Oxford Brookes University, UK
10. Supervising Trials Manager	Catherine Spowart	University of Liverpool, UK
11. Trial Manager	Lucy Stibbs-Eaton	University of Liverpool, UK
12. Trial Statistician	Liam Whittle	University of Liverpool, UK
13. CASTLE Programme Manager	Amber Collingwood	King's College London, UK
14. Researcher	Georgia Cook	Oxford Brookes University, UK
15. Researcher	Kristina C. Dietz	King's College London, UK
16. Health economist	Will A. S. Hardy	Bangor University, UK
17. Researcher	Holly Saron	Edge Hill University, UK
**Trial Steering Committee (TSC)**<br>**Responsibilities**: Overall trial supervision and advice, ultimate decision for the continuation of the trial.<br>**Meeting frequency**: At least annually.		
1. Chair	Jeremy Parr	Newcastle University, UK
2. Medical statistician	Martyn Lewis	Keele University, UK
3. Paediatrician	Desaline Joseph	Evelina London Children's Hospital, UK
4. Public and Patient Involvement Representative	Jo Conduit-Smith	CASTLE Advisory Panel
5. Chief Investigator	Deb K. Pal	King's College London, UK
6. Co-Chief Investigator	Paul Gringras	Evelina London Children's Hospital, UK

For peer review only - http://bmjopen.bmj.com/site/about/guidelines.xhtml

Independent Data and Safety Monitoring Committee (IDSMC)<br>**Responsibilities:** Interim monitoring of safety and effectiveness, trial conduct and external data. Recommendation to TSC about trial continuation.<br>**Meeting frequency:** At least annually		
1. Chair	Helen Cross	University College London, UK
2. Paediatrician	Alberto Verroti	University of L'aquila, Italy
3. Medical statistician	• Anthony Johnson (to 31/August/2022)<br>• Appointment pending (20/December/2022)	University College London, UK

For peer review only

For peer review only - http://bmjopen.bmj.com/site/about/guidelines.xhtml

Supplemental Table 3. Psychometrics and clinical relevance/minimal clinically important difference (CR/MCID) for CASTLE Sleep-E outcomes (Table 1). Metrics refer to the single referenced publication. Further validation studies exist, but, due to differences in population, setting, and/or methods, results cannot be merged.

**Outcome**	**Description**	**Validity**	**Reliability**	**CR/MCID**
Children's Sleep Habits Questionnaire (CSHQ)[1]	Parent-reported, one-week retrospective sleep screening tool for children (4–10 years)<br><br>35 items (2 duplicated across subscales)<br>3-point Likert scales (rarely, sometimes, usually)<br>Total score (33 items): 33–99, lower is better<br>8 subscales:<br>• Bedtime Resistance (6 items)<br>• Sleep Onset Delay (1 item)<br>• Sleep Duration (3 items)<br>• Sleep Anxiety (4 items)<br>• Night Wakings (3 items)<br>• Parasomnias (7 items)<br>• Sleep-Disordered Breathing (3 items)<br>• Daytime Sleepiness (8 items)<br><br><u>Validation samples</u><br>Parents of 469 school children (community setting) and 154 children diagnosed with sleep disorder (hospital setting); English language; England, UK.<br>Test-retest: 60 parents from control sample	<u>Classification accuracy</u><br>Sleep disorder (yes/no)<br>Receiver Operating Characteristic (ROC) analyses: See MCID<br><br><u>Construct validity</u><br>See MCID<br><br><u>Criterion validity</u><br>Not assessed	<u>Test-retest</u><br>2-week delay<br>Pearson's *r:* 0.62–0.79<br><br><u>Internal consistency</u><br>Cronbach's α<br>Control sample: 0.68<br>Clinical sample: 0.78<br><br><u>Inter-rater reliability</u><br>Not assessed	Cut-off (total score): 41<br>• Sensitivity: 80 %<br>• Specificity: 72 %<br>• Accuracy: 80 %<br><br><u>MCID</u><br>Not assessed
EQ-5D-Y[2 3]	Child- or adolescent reported (4–7 years: EQ-5D-Y proxy; 8–16 years: EQ-5D-Y, ≥16 years: EQ-5D-5L), standardised measure of current ('today')<br>• *health profile* across 5 dimensions,<br>• self-rated *health status*, and<br>• *EQ-5D-Y index value,* using a country-specific weighting	Not yet validated in UK (last updated 07/March/2022)	Not yet validated in UK (last updated 07/March/2022)	<u>CR/MCID</u><br>Applicability to utility scores debated, suggested MCID: difference in index score between baseline health profile and single-level transitions in single domain (e.g. 33333 to 33332).

For peer review only

For peer review only - http://bmjopen.bmj.com/site/about/guidelines.xhtml

Outcome	Description	Validity	Reliability	CR/MCID
	(value set) of a given health profile.<br><br>Two components:<br>1. <u>Descriptive system</u><br>5 dimensions with 3 response severity options each (tick-box):<br>• Mobility<br>• Self-care<br>• Usual activities<br>• Pain/discomfort<br>• Anxiety/depression<br>2. <u>Visual Analogue Scale</u><br>Self-rated health on a vertical Visual Analogue Scale (VAS) that ranges from 'The best health you can imagine' (100) to 'The worst health you can imagine' (0).<br><br>Scoring:<br>• Descriptive system: 5-digit *health profile* (best health state: 11111, indicating no problem in each of the 5 dimensions; worst health state: 33333 indicating many problems in each of the 5 dimensions; 243 possible health states are coded)<br>• VAS: 0–100 subjective *health state* (worst to best)<br>• *EQ-5D-5L index value* Single summary number, calculated by subtracting country-specific weighing (value set) of an obtained health profile from 1, where 1 represents the best possible health profile of 11111.<br><br><u>Value set validation sample (UK)</u><br>Not yet validated in UK (last updated 07/March/2022)			

For peer review only - http://bmjopen.bmj.com/site/about/guidelines.xhtml

Outcome	Description	Validity	Reliability	CR/MCID
Child Health Utility instrument (CHU-9D)[4]	Child-reported (7–11 years) descriptive system for current ('today') generic health-related quality-of-life<br><br>9 dimensions with 5 response severity options each (circle):<br>• Worried<br>• Sad<br>• Pain<br>• Tired<br>• Annoyed<br>• School-/homework<br>• Sleep<br>• Daily routine<br>• Activities<br><br>Scoring:<br>• Descriptive system: 9-digit *health profile* (best health state: 111111111, indicating no problem in each of the 9 dimensions; worst health state: 555555555 indicating many problems in each of the 5 dimensions; 1953125 possible health states are coded)<br>• *CHU-9D index value* Single summary number indicating the utility value of a given health state, established using Standard Gamble (SG) tasks.<br><br><u>Value set validation sample (England)</u><br>1245 households were randomly sampled from a database of UK names and addresses in Sheffield and Huddersfield (England) were contacted by a research team of the Centre for Research and Evaluation (CRE) at Sheffield Hallam	<u>Predictive accuracy</u><br>Standard ordinary least squares (OLS) regression: 98.41 %<br>No systematic bias, no auto-correlated errors.<br><br><u>Construct validity</u><br>Not assessed<br><br><u>Criterion validity</u><br>Not assessed<br><br><u>Face-validity</u><br>Preference elicitation using Standard Gamble (SG) task, which give the choice of living in a specific health-state until death with certainty (Choice A), or taking a gamble (Choice B) that could result in living in perfect health for the rest of life with a probability *p*, or dying with a probability 1-*p*. The utility value of a given health-state is the point of indifference between options A and B.<br>Utility values are consistent with health profiles but required merging of response options.	<u>Test-retest</u><br>Not assessed<br><br><u>Internal consistency</u><br>Utility values are consistent with health profiles, but required merging of the initial 5 response-levels for all but one of the 9 dimensions as follows:<br>• Worried: 2<br>• Sad: 4<br>• Pain: 4<br>• Tired: 2<br>• Annoyed: 2<br>• School-/homework: 2<br>• Sleep: 4<br>• Daily routine: 5<br>• Activities: 3<br><br><u>Inter-rater reliability</u><br>Not assessed	<u>CR/MCID</u><br>Applicability to utility scores debated, suggested MCID: difference in index score between baseline health profile and single-level transitions in single domain (e.g. 555555555 to 555555554).

For peer review only - http://bmjopen.bmj.com/site/about/guidelines.xhtml

Outcome	Description	Validity	Reliability	CR/MCID
	University. 1195 households were approached at the door, of which 661 (55 %) were in, and 300 (25 %) agreed to take part. 282 respondents (all adults) were analysed (94 %). Compared to the general UK population, this adult sample was broadly representative, but more affluent and highly restricted geographically. Modelling did not include key demographic characteristics (e.g. age, gender, education, employment, religion and ethnicity). The sample consisted exclusively of adults but was used to derive a paediatric value set.			
EQ-5D-5L[5]	Adolescent or adult-reported (≥16 years), standardised measure of current ('today'):<br>• *health profile* across 5 dimensions,<br>• subjective *health status*, and<br>• *EQ-5D-5L index value,* using a country-specific weighting (value set) of an obtained health profile.<br><br>Two components:<br>1. Descriptive system<br>5 dimensions with 5 response severity options each (tick-box):<br>• Mobility<br>• Self-care<br>• Usual activities<br>• Pain/discomfort<br>• Anxiety/depression<br>2. Visual Analogue Scale<br>Self-rated health on a vertical Visual Analogue Scale (VAS) that ranges from 'The best health	Classification accuracy<br>Not assessed<br><br>Construct validity<br>Not assessed<br><br>Criterion validity<br>Not assessed<br><br>Face-validity<br>Preference elicitation using time trade-off (TTO) and discrete choice experiments (DCEs).<br>• TTOs: Confirmation of negative relationship between level sum score and average observed value.<br>• DCEs: Confirmation of assumption that health states with lower-level sum	Test-retest<br>Not assessed<br><br>Internal consistency<br>Not assessed<br><br>Inter-rater reliability<br>Not assessed	CR/MCID<br>Applicability to utility scores debated, suggested MCID: difference in index score between baseline health profile and single-level transitions in single domain (e.g. 55555 to 55554).

For peer review only - http://bmjopen.bmj.com/site/about/guidelines.xhtml

Outcome	Description	Validity	Reliability	CR/MCID
	you can imagine' (100) to 'The worst health you can imagine' (0).<br><br>Scoring:<br>• Descriptive system: 5-digit *health profile* (best health state: 11111, indicating no problem in each of the 5 dimensions; worst health state: 55555 indicating many problems in each of the 5 dimensions; 3125 possible health states are coded)<br>• VAS: 0–100 subjective *health state* (worst to best)<br>• *EQ-5D-5L index value* Single summary number, calculated by subtracting country-specific weighing (value set) of an obtained health profile from 1, where 1 represents the best possible health profile of 11111.<br><br><u>Value set validation sample (England)</u><br>2220 households from 66 post-code based primary sampling units in England were contacted by the market research company Ipsos MORI. 2088 participants were invited, of which 996 (47.7 %) completed the valuation questionnaire. Only complete responses were analysed (985 participants, 98.9 %). Compared to the general population of England, the sample included more people aged over 75 years, retired, and with health problems, but fewer younger	scores are more likely to be chosen.		

For peer review only

For peer review only - http://bmjopen.bmj.com/site/about/guidelines.xhtml

Outcome	Description	Validity	Reliability	CR/MCID
	participants, and fewer males.			
Knowledge About Sleep in Childhood (KASC, custom-scale devised for CASTLE Sleep-E)	13 items<br>Self-reported Likert-scales assessing parental efficacy in managing child sleep and knowledge about child sleep	Not evaluated	Not evaluated	Not evaluated
Hospital Anxiety and Depression Scale (HADS)[6]	Self-reported, one-week retrospective screening tool for anxiety and depression in people aged 16–65.<br><br>14 items<br>5-point Likert scales (0–3)<br>No total score<br>Subscale score: 0–21, lower is better<br>2 subscales (7 items each):<br>• Depression<br>• Anxiety<br><br>Validation samples<br>2 x 50 patients (16–65 years) with and without psychiatric disorders (hospital setting); English language; England, UK.	Classification accuracy<br>Psychiatric interview,<br>see CR/MCID<br><br>Construct validity<br>See CR/MCID<br><br>Convergent validity<br>Spearman's ρ<br>Interview/self-rating<br>Depression/Depression: 0.79<br>Anxiety/Anxiety: 0.54<br><br>Discriminant validity<br>Spearman's ρ<br>Interview/self-rating<br>Depression/Anxiety ns<br>Anxiety/Depression ns<br><br>Criterion validity<br>See CR/MCID	Test-retest<br>Not assessed<br><br>Internal consistency<br>Spearman's ρ<br>Anxiety: 0.41–0.76<br>Depression: 0.30–0.60<br><br>Inter-rater reliability<br>Not assessed	Cut-offs (subscales)<br>Depression<br>Absent: ≤ 7<br>Borderline: 8–10<br>Definite: ≥ 11<br>• False positives: 1 %<br>• False negatives: 1 %<br>Borderline not counted as error<br><br>Anxiety<br>Absent: ≤ 7<br>Doubtful: 8–10<br>Definite: ≥ 11<br>• False positives: 5 %<br>• False negatives: 1 %<br>Borderline not counted as error<br><br>MCID<br>Not assessed

For peer review only - http://bmjopen.bmj.com/site/about/guidelines.xhtml

Outcome	Description	Validity	Reliability	CR/MCID
Insomnia Severity Index (ISI)[7], patient version	Self-reported, one-month retrospective screening tool for insomnia in adults (≥18 years)<br>7 items<br>5-point Likert scales (0–4, no problem to severe problem)<br>Total score: 0–28, lower is better<br>• 0–7: Absence of insomnia<br>• 8–14: Subthreshold insomnia<br>• 15–21: Moderate insomnia<br>• 22–28: Severe insomnia<br>Dimensions:<br>• Severity of sleep onset<br>Sleep maintenance<br>• Early morning awakening problems<br>• Sleep dissatisfaction<br>• Interference of sleep difficulties with daytime functioning<br>• Noticeability of sleep problems by others<br>• Distress caused by the sleep difficulties<br><br>Validation samples<br>959 adults with and without insomnia (community setting), 183 adults with insomnia and 62 controls (clinical setting); English language; Québec, Canada.	Classification accuracy<br>Insomnia (yes/no)<br>ROC analyses, see MCID<br><br>Construct validity<br>See CR/MCID<br>Pearson's *r*<br>• Daily sleep diary: 0.54–0.59<br>• Activity level, Anxiety (state, trait), Depression, Fatigue (general, physical, mental), Motivation: 0.20–0.48<br><br>Criterion validity<br>Pearson's *r*<br>Polysomnography<br>• Sleep onset latency: ns<br>• Wake after sleep onset: ns<br>• Number of awakenings: ns<br>• Early morning awakening: ns<br>• Total wake time: ns<br>• Sleep efficiency: -0.16	Test-retest<br>Not assessed<br><br>Internal consistency<br>Cronbach's α, Control sample: 0.71<br>Clinical sample: 0.73<br><br>Inter-rater reliability<br>Not assessed	Control sample (self-diagnosis)<br>Cut-off (total score): 10<br>• Sensitivity: 86 %<br>• Specificity: 88 %<br>• Accuracy: 87 %<br><br>Clinical sample<br>Cut-off (total score): 11<br>• Sensitivity: 97 %<br>• Specificity: 100%<br>Accuracy: 98 %<br><br>MCID<br>Change required for improvement<br>Blinded assessor, *M*, [CI_{95}]:<br>• Slight: 4.65 [2.61–6.69]<br>• Moderate: 8.36 [7.20–9.53]<br>• Marked: 9.89 [8.74–11.04]<br>ROC analyses:<br>• Slight: not reported<br>• Moderate: ≥7<br>○ Sensitivity: 60 %<br>○ Specificity: 70 %<br>○ Accuracy: not reported<br>• Marked: ≥8<br>○ Sensitivity: 64 %<br>○ Specificity: 80 %<br>○ Accuracy: not reported

For peer review only - http://bmjopen.bmj.com/site/about/guidelines.xhtml

Outcome	Description	Validity	Reliability	CR/MCID
SleepSuite[8] (iPad App): Bubble task<br>• Executive function (accuracy and response times [RT])	SleepSuite bubble tasks (iPad games) are adapted from a validated Balloon Task[9]: The goal is to burst upward drifting balloons with children's faces under multiple target conditions (e.g. happy faces only) and at increasing presentation conditions (speed, load: number of faces shown simultaneously).<br><br>Validation sample[9]<br>134 healthy children (7–12 years, 58 boys, 23 with clinical behavioural problems, 40% first-born) from middle- and upper-class families of which 25% included at least one parent who immigrated more than 10 years ago. Children lived with their parents in small households (on average 4.53 members). Parents were largely employed full-time (fathers: 90.71%, mothers: 49.31%) and well educated (on average for 16 years). Community setting (school, number unspecified); paid participation ($15 school supply voucher); language: Hebrew, Israel.	Classification accuracy<br>Not assessed<br><br>Construct validity<br>Not assessed<br><br>Criterion validity<br>Child Behavior Checklist (CBCL): total score, sub-scales (8), recode to externalising and internalising behaviours.<br><br>Pearson's *r* (age and sex partialled out), across conditions<br><br>**Completed levels/RT**<br>• Total score: -0.24/ns<br>• Delinquency: ns/0.18<br>• Aggression: -0.20/0.23<br>• Attention problems: -0.18/ns<br>• Social withdrawal: -0.24/ns<br>• Somatic complaints: ns/0.18<br>• Thought disorders: ns/ns<br>• Anxiety-Depression: -.28/ns<br>• Social problems: -0.20/ns<br>• Externalising behaviours: -0.18/0.23<br>• Internalising behaviours: -0.25/ns	Test-retest<br>Delay unspecified (likely none [immediate retest])<br><br>Pearson's *r*<br>• Hits: 0.60<br>• Misses: 0.37<br>• Completed levels: 0.39<br>• RT: 0.78<br><br>Internal consistency<br>Not assessed<br><br>Inter-rater reliability<br>Not assessed	Not assessed

For peer review only - http://bmjopen.bmj.com/site/about/guidelines.xhtml

Outcome	Description	Validity	Reliability	CR/MCID
Health-Related **Q**uality **Of** **L**ife Measure for **Ch**ildren with **E**pilepsy (CHEQOL)[10]	Quality of life assessment tool for children or parents with epilepsy (no specified time-period); child reported if ≥8 years, parent proxy-report if child 5 to <8 years<br>25 items<br>4-point Likert scales (0–4, opposites: true/sort of true)<br>Total score: 25–100, higher is better<br>5 subscales (5 items each):<br>• Interpersonal/social consequences<br>• Future worries<br>• Present worries<br>• Intrapersonal/emotional<br>• Epilepsy secrecy<br><br><u>Validation samples</u><br>381 children (6–15 years) with epilepsy and their parents (clinical setting); English language; Ontario, Canada. Test-retest: Additional 89, then 31 children; additional 48 parents.<br>Metrics refer to self-report for children 8–15 years and parent proxy report for children 5 to <8 years and were assessed for sub-scales, not total score.	<u>Classification accuracy</u><br>Not assessed<br><br><u>Construct validity (child)</u><br>Pearson's *r*<br>• Health care utilisation: 0.13–0.31<br>• Drug Adverse Events: 0.18–0.25<br>• Number of friends: 0.18<br>• Nº of extracurricular activities: 0.13<br>One-way ANOVA ($p \leq .05$)<br>• Seizure severity: All 5 subscales<br>• Anti-epileptic drug use: 4 subscales<br>*t–tests* ($p \leq .05$)<br>• Help at school: All 5 subscales<br>Results for parent-proxy similar<br><br><u>Criterion validity</u><br>Not assessed	<u>Test-retest</u><br>10– 14 days delay<br>Intraclass correlation coefficient<br>Child: 0.59–0.69<br>Parent: 0.60–0.81<br><br>*<u>Internal consistency</u>*<br>Cronbach's *α*, subscales<br>*Child: 0.63–*0.84<br>*Parent: 0.64–*0.86<br><br><u>Inter-rater reliability</u><br>Pearson's *r*<br>• Child/mother: 0.24–0.56<br>• Child/father: 0.18–0.54<br>• Mother/father: 0.40–0.71	Not assessed

For peer review only

For peer review only - http://bmjopen.bmj.com/site/about/guidelines.xhtml

Outcome	Description	Validity	Reliability	CR/MCID
World Health Organisation – Five Well-Being Index (WHO-5)[11]	Self-reported, two-week retrospective tool to assess subjective psychological well-being in people aged 9 years and older.<br><br>5 items<br>6-point Likert scales (0–5, 'at no time' to 'all the time')<br>Raw score: 0–25<br>Total score multiplied by 4 to give final score: 0–100, higher is better<br><br>Validation samples<br>446 children analysed (9–12 years, 16 [3.6 %] with depressive disorder), 6 additional participants dropped due to incomplete data.<br>Hospital setting: 3 paediatric hospitals and 3 paediatric surgery hospitals (in- and out-patients for non-psychiatric reasons), Munich, Germany.<br>German language.	Classification accuracy<br>Depressive disorder (yes/no)<br>Receiver Operating Characteristic (ROC) analyses: See CR/MCID<br><br>Construct validity<br>See CR/MCID<br><br>Criterion validity<br>Diagnostic and Statistical Manual of Mental Disorders (DSM-IV) criteria for depressive disorder (major or minor depression only, dysthymia dropped due to mismatch in time-period of concept definitions), see CR/MCID.	Test-retest<br>Not assessed<br><br>Internal consistency<br>Not assessed<br><br>Inter-rater reliability<br>Cohen's k = .90	Cut-off (total score): 10<br>• Sensitivity: 75 %<br>• Specificity: 92 %<br>• Accuracy: 88 %<br><br>MCID<br>Not assessed

For peer review only - http://bmjopen.bmj.com/site/about/guidelines.xhtml

Outcome	Description	Validity	Reliability	CR/MCID
Strengths and Difficulties Questionnaire (SDQ)[12]	Parent-, teacher-, or child-reported, retrospective screening tool of child psychopathology (2–18 years). Retrospective period: 6 months or current school year<br><br>25 items<br>3-point Likert scales (0–2, not/somewhat/certainly true)<br>Total score: 0–40, lower is better<br>5 subscales (5 items each):<br>• hyperactivity/inattention,<br>• emotional problems<br>• conduct problems<br>• peer problems<br>• prosocial behaviours (omitted from total score)<br><br>Validation samples<br>541 children (5–12 years) with and without psychiatric disorders (school setting); multiple languages; Italy, Germany, the Netherlands, Lithuania, Bulgaria, Romania, and Turkey. Metrics refer to parent-report, total score, and data aggregated across countries and psychiatric disorders.	Classification accuracy<br>Psychiatric disorder (yes/no)<br>Receiver Operating Characteristic (ROC) analyses: See CR/MCID<br>Original total score cut-offs:<br>• Normal: 0–13<br>• Borderline: 14–16<br>• Abnormal: 17–40<br>transformed to binary:<br>• No: 0–16<br>• Yes: 17–40<br><br>Construct validity<br>See CR/MCID<br><br>Criterion validity<br>Diagnostic and Statistical Manual of Mental Disorders (DSM-IV), see CR/MCID.	Test-retest<br>Not assessed<br><br>Internal consistency<br>Cronbach's α: 0.84<br><br>Inter-rater reliability<br>Not assessed.	Cut-off (total score): 17<br>• Sensitivity: 88 %<br>• Specificity: 59 %<br>• Accuracy: 74 %<br><br>MCID<br>Not assessed

For peer review only

For peer review only - http://bmjopen.bmj.com/site/about/guidelines.xhtml

Outcome	Description	Validity	Reliability	CR/MCID
Parenting Self Agency Measure (PSAM)[13]	Self-reported tool assessing overall confidence to successfully parent (including managing the child's behaviour and resolving problems with the child). The time-period for parental self-assessment is unspecified.<br><br>5 items<br>7-point Likert scales (1–7, rarely to always)<br>Total score: 5–35, higher is better<br><br>Validation sample<br>90 English-speaking mothers (all European-American, median age 36–40 years, median annual income >$40,000, median education bachelor's degree, 82% married or co-habiting) of 3–12-year-olds (community setting); 2 day-care centres and classes at a large university, 2 churches. English language, southwestern USA.	Classification accuracy<br>Not assessed<br><br>Construct validity<br>Convergent validity<br>Pearson's *r*<br>Active coping: 0.31<br>Parenting acceptance: 0.55<br>Positive re-interpretation: ns<br><br>Discriminant validity<br>Pearson's *r*<br>Inconsistent parental disciplining: -0.34<br>Acceptance coping: ns<br><br>Criterion validity<br>Not assessed	Test-retest<br>Not assessed<br><br>Internal consistency<br>Cronbach's α: 0.70<br>Comparative Fit Index: 0.94<br><br>Inter-rater reliability<br>Not assessed	Not assessed

For peer review only - http://bmjopen.bmj.com/site/about/guidelines.xhtml

Outcome	Description	Validity	Reliability	CR/MCID
Actigraphy: Micro Motionlogger® Watch, Watchware Software V 1.99.17.4, Action-W software, V 2.7.3285 (Ambulatory Monitoring, Inc., NY: USA) combined with sleep diaries (Child and Parent)<br><br>• Total sleep time (minutes)<br>• Sleep latency (minutes)<br>• Sleep efficiency (% asleep of sleep period)<br><br>All 2-week averages	The Micro-Motionlogger® Watch directly measures 3-D acceleration (in CASTLE Sleep-E and the referenced validation study of the non-dominant wrist). Raw data (zero-crossing mode) is initially recorded as periods of activity and inactivity (1 min epochs), and then recoded into periods of wakefulness and sleep using a combination of proprietary algorithms and manual processing (e.g. sleep periods are visually inspected and manually corrected with the aid of participant sleep diaries). Sleep- and wake parameters are then calculated automatically using validated public algorithms.<br><br><u>Validation sample[9]</u><br>27 children (3–17 years) with medically refractory epilepsy, of which 12 had parent-indicated sleep problems (44%). Hospital setting (in-patient epilepsy monitoring unit in tertiary paediatric hospital), English language, Toronto, Canada.	<u>Classification accuracy</u><br>Not assessed<br><br><u>Construct validity</u><br>Not assessed<br><br><u>Criterion validity</u><br>Agreement of actigraphy with continuous video-electroencephalography (24 hours), scored by neurologist and neurophysiologist.<br><br>Bland-Altman plots in combination with *t*-tests for significant bias:<br>• Total sleep time (minutes): Bias = 8.3 (SD = 31), n.s.<br>• Wake duration: Bias = -4.8 (SD = 31.1), n.s.<br><br>Pearson's *r*:<br>• Total sleep time (minutes): 0.96<br>• Wake duration: 0.93	<u>Test-retest</u><br>Not assessed<br><br><u>Internal consistency</u><br>Not assessed<br><br><u>Inter-rater reliability</u><br>Not assessed	Not assessed

For peer review only - http://bmjopen.bmj.com/site/about/guidelines.xhtml

Table 4. Estimated overall time requirement for CASTLE Sleep-E (participant perspective). Time estimates for questionnaires/instruments are based on published estimates where available, and otherwise on an estimate (indicated by *) of 30 seconds per item derived from the Children's Sleep Habits Questionnaire (35 items, 10 minutes published completion time), plus an arbitrary estimate of 2 minutes to read instructions and consider responses. The total time requirement for participation in CASTLE Sleep-E varies from minimally 2 hours per month over a 6-month period in the Standard Care arm omitting optional qualitative interviews to maximally 3 hours per month over a 6-month period in the intervention arm including optional qualitative interviews.

**Trial component**	**Time (mins)**	**Frequency**	**Overall time (mins)**
Study visits (4)<br>Remote or in-person, combinable with standard care visits<br>• Consent and baseline data<br>• Randomisation<br>• Follow-up at 3 months<br>• Follow-up at 6 months	 <br>• 60 minutes<br>• 30 minutes<br>• 30 minutes<br>• 30 minutes	 <br>• 1<br>• 1<br>• 1<br>• 1	150 minutes
Questionnaires/instruments in order of the participant timeline shown in Table 4<br>• Children's Sleep Habits Questionnaire[1], 35 items<br>• World Health Organisation – Five Well-Being Index[11], 5 items<br>• Health-Related Quality Of Life Measure for Children with Epilepsy[10], 25 items<br>• Strengths and Difficulties Questionnaire[12], 25 items<br>• Child Health Utility Index 9D (CHU-9D)/CHU-9D proxy[4], 9 items<br>• EQ-5D-Y/EQ-5D-Y proxy[2], 15 items<br>• EQ-5D-5L[5], 25 items (note: Published time estimate same as for EQ-5D-Y [15 items])<br>• Parenting Self Agency Measure[13], 5 items<br>• Insomnia Severity Index[7], patient version, 7 items<br>• Hospital Anxiety and Depression Scale[6], 14 items<br>• Resource Use questionnaire (custom instrument), 11 items<br>• Knowledge About Sleep in Childhood (custom scale), 13 items	 • 10 minutes<br>• 5 minutes<br>• 12.5 + 2 minutes*<br>• 12.5 + 2 minutes*<br>• 4.5 + 2 minutes*<br>• 5 minutes<br>• 5 minutes<br>• 2.5 + 2 minutes*<br>• 3.5 + 2 minutes*<br>• 5 minutes<br>• 5.5 + 2 minutes*<br>• 6.5 + 2 minutes*	 • 3<br>• 2<br>• 2<br>• 3<br>• 3<br>• 3<br>• 3<br>• 3<br>• 3<br>• 3<br>• 3<br>• 2	246.5 minutes<br>• 30 minutes<br>• 10 minutes<br>• 29 minutes<br>• 43.5 minutes<br>• 19.5 minutes<br>• 15 minutes<br>• 15 minutes<br>• 13.5 minutes<br>• 16.5 minutes<br>• 15 minutes<br>• 22.5 minutes<br>• 21 minutes
SleepSuite[8] (iPad App)<br>• Morning of single day<br>• Evening of single day	40 minutes<br>• 20 minutes<br>• 20 minutes	2	80 minutes

For peer review only - http://bmjopen.bmj.com/site/about/guidelines.xhtml

Trial component	Time (mins)	Frequency	Overall time (mins)
Actigraphy<br>• Delivery arrangements to participants' home or collection point (incl. SleepSuite iPad)<br>○ Baseline<br>○ Follow-up at 3 months<br>• Return arrangements to participants' home or collection point (incl. SleepSuite iPad)<br>○ Baseline<br>○ Follow-up at 3 months<br>• Use: Removal and re-fitting of device once daily (2 x 0.25 minute) when showering, bathing, or swimming; otherwise, the device is worn like a wristwatch without requiring participant interventions.<br>○ Baseline: 14 days<br>○ Follow-up at 3 months: 14 days	• 15 minutes<br>• 15 minutes<br>• 15 minutes<br>• 15 minutes<br>• 7 minutes<br>• 7 minutes	• 1<br>• 1<br>• 1<br>• 1<br>• 1<br>• 1	74 minutes
Sleep diary<br>Once daily completion of parent- and child diary (2 x 2.5 minutes)<br>• Baseline: 14 days<br>• Follow-up at 3 months: 14 days	• 70 minutes<br>• 70 minutes	• 1<br>• 1	140 minutes
COSI (*intervention arm only*)<br>• 3 mandatory modules (core information about sleep relevant to all families)<br>• 3 recommended modules (e.g. sleep hygiene)<br>• 5 tailored modules (addressing specific sleep issues indicated by a given parent)<br>• List of additional resources, *optional*, 10 webpages, not included in time estimate<br>• Evaluation questionnaire, 3 sections, 47 items overall<br>A parent assigned to COSI (i.e. the intervention arm) would be expected to look at minimally 7 and maximally 11 modules. All modules are self-paced (i.e. do not have a fixed duration). To read and engage with a single module could take anywhere between 5–20 minutes depending on how quickly one reads, whether one watches the videos, does the quizzes, etc. Consequently, the estimated time requirement for initial material completion not including breaks or re-visits is 35–220 minutes for modules alone.<br>To be conservative, maximal estimates are used in calculations.	• 60 minutes<br>• 60 minutes<br>• 100 minutes<br>• 0 minutes<br>• 23.5 + 2 minutes*	• 1<br>• 1<br>• 1<br>• 1<br>• 1	245.5 minutes

For peer review only - http://bmjopen.bmj.com/site/about/guidelines.xhtml

Trial component	Time (mins)	Frequency	Overall time (mins)
Qualitative interviews (*optional*)<br>Two time-points (Follow-up at 3 months + 3 weeks, at 6 months + 3 weeks)<br>• Interview date and time arrangement<br>• Interview preparation using supplied interview guide<br>• Actual interview<br>• De-brief<br>For the qualitative interviews with parents, we typically expect that the total time burden for each of the two interviews would range from 30–70 minutes. However, we will tailor the core interview to fit with the time the parent has available, so some interviews may be a little longer or shorter.<br>To be conservative, maximal estimates are used in calculations.	 <br>• 10 minutes<br>• 10 minutes<br>• 40 minutes<br>• 10 minutes	 <br>• 2<br>• 2<br>• 2<br>• 2	140 minutes<br><br>• 20 minutes<br>• 20 minutes<br>• 80 minutes<br>• 20 minutes
**Total time for participation over a 6-months period**<br>• **Standard Care arm (SC), not participating in optional qualitative interviews**<br>• **Standard Care arm (SC), participating in optional qualitative interviews**<br>• **Intervention arm (SC + COSI), not participating in optional qualitative interviews**<br>• **Intervention arm (SC + COSI), participating in optional qualitative interviews**			 • **690.5 minutes**<br>• **830.50 minutes**<br>• **936 minutes**<br>• **1076 minutes**

For peer review only - http://bmjopen.bmj.com/site/about/guidelines.xhtml

Supplemental Table 5. Categories used to define the causality and severity of Adverse Events in CASTLE Sleep-E

Category	Definition
Causality	
Almost Certainly	There is clear evidence to suggest a causal relationship, and other possible contributing factors can be ruled out.
Probably	There is evidence to suggest a causal relationship, and the influence of other factors is unlikely.
Possibly	There is some evidence to suggest a causal relationship (e.g. the event occurred within a reasonable time after administration of the study procedure). However, the influence of other factors may have contributed to the event (e.g. the participant's clinical condition, other concomitant events).
Unlikely	There is little evidence to suggest there is a causal relationship (e.g. the event did not occur within a reasonable time after administration of the study procedure). There is another reasonable explanation for the event (e.g. the participant's clinical condition).
Not related	There is no evidence of any causal relationship.
Severity	
Mild	The Adverse Event does not interfere with the participant's daily routine and does not require further procedure; it causes slight discomfort.
Moderate	The Adverse Event interferes with some aspects of the participant's routine, or requires further procedure, but is not damaging to health; it causes moderate discomfort.
Severe	The Adverse Event results in alteration, discomfort or disability which is clearly damaging to health.

For peer review only - http://bmjopen.bmj.com/site/about/guidelines.xhtml

References

1. Owens JA, Spirito A, McGuinn M. The Children's Sleep Habits Questionnaire (CSHQ): psychometric properties of a survey instrument for school-aged children. Sleep 2000;**23**(8):1043-51.
2. Wille N, Badia X, Bonsel G, et al. Development of the EQ-5D-Y: a child-friendly version of the EQ-5D. Qual Life Res 2010;**19**(6):875-86 doi: 10.1007/s11136-010-9648-y [published Online First: 20100420].
3. EuroQol Research Foundation. EQ-5D-Y User Guide, 2020.
4. Stevens K. Valuation of the Child Health Utility 9D Index. Pharmacoeconomics 2012;**30**(8):729-47 doi: 10.2165/11599120-000000000-00000.
5. Devlin NJ, Shah KK, Feng Y, et al. Valuing health-related quality of life: An EQ-5D-5L value set for England. Health Econ 2018;**27**(1):7-22 doi: 10.1002/hec.3564 [published Online First: 20170822].
6. Zigmond AS, Snaith RP. The hospital anxiety and depression scale. Acta Psychiatr Scand 1983;**67**(6):361-70 doi: 10.1111/j.1600-0447.1983.tb09716.x.
7. Morin CM, Belleville G, Bélanger L, et al. The Insomnia Severity Index: psychometric indicators to detect insomnia cases and evaluate treatment response. Sleep 2011;**34**(5):601-08 doi: 10.1093/sleep/34.5.601.
8. Colonna A, Smith AB, Smith S, et al. The Effects of Sleep on Emotional Target Detection Performance: A Novel iPad-Based Pediatric Game. Frontiers in psychology 2018;**9**:241-41 doi: 10.3389/fpsyg.2018.00241.
9. Sadaka Y, Sadeh A, Bradbury L, et al. Validation of actigraphy with continuous video-electroencephalography in children with epilepsy. Sleep Med 2014;**15**(9):1075-81 doi: 10.1016/j.sleep.2014.04.021 [published Online First: 20140602].
10. Ronen GM, Streiner DL, Rosenbaum P, et al. Health-related quality of life in children with epilepsy: development and validation of self-report and parent proxy measures. Epilepsia 2003;**44**(4):598-612 doi: 10.1046/j.1528-1157.2003.46302.x.
11. Allgaier A-K, Pietsch K, Frühe B, et al. Depression in pediatric care: is the WHO-Five Well-Being Index a valid screening instrument for children and adolescents? General Hospital Psychiatry 2012;**34**(3):234-41 doi: https://doi.org/10.1016/j.genhosppsych.2012.01.007.
12. Goodman R. Psychometric Properties of the Strengths and Difficulties Questionnaire. Journal of the American Academy of Child & Adolescent Psychiatry 2001;**40**(11):1337-45 doi: 10.1097/00004583-200111000-00015.
13. Dumka LE, Stoerzinger HD, Jackson KM, et al. Examination of the Cross-Cultural and Cross-Language Equivalence of the Parenting Self-Agency Measure. Family Relations 1996;**45**(2):216-22 doi: 10.2307/585293.

For peer review only

For peer review only - http://bmjopen.bmj.com/site/about/guidelines.xhtml

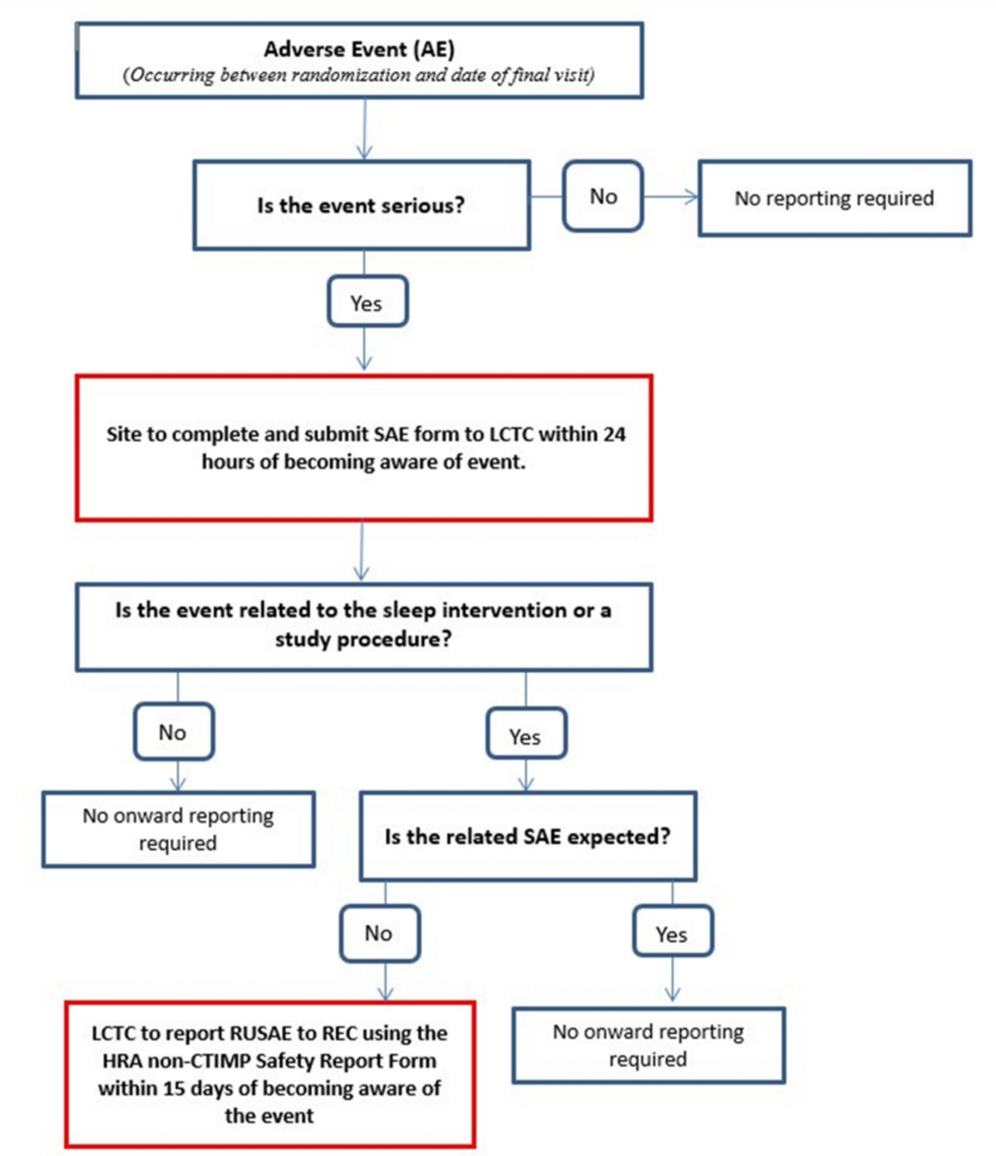

Supplemental Figure 1. Flowchart showing reporting requirements of Adverse Events for the CASTLE Sleep-E trial. Acronyms: Serious Adverse Event (SAE), Liverpool Clinical Trial Centre (LCTC), Related Unexpected Serious Adverse Event (RUSAE), Health Research Authority (HRA), non- Clinical Trial of Investigational Medicinal Products (non-CTIMP).

2184x2612mm (38 x 38 DPI)

SPIRIT
STANDARD PROTOCOL ITEMS: RECOMMENDATIONS FOR INTERVENTIONAL TRIALS

SPIRIT 2013 Checklist: Recommended items to address in a clinical trial protocol and related documents*

Section/item	Item No	Description	Addressed on page number
**Administrative information**			
Title	1	Descriptive title identifying the study design, population, interventions, and, if applicable, trial acronym	p1
Trial registration	2a	Trial identifier and registry name. If not yet registered, name of intended registry	p2, Suppl. Table 1 (item 1)
	2b	All items from the World Health Organization Trial Registration Data Set	Suppl. Table 1
Protocol version	3	Date and version identifier	Suppl. Table 1 (item 25)
Funding	4	Sources and types of financial, material, and other support	p22, Suppl. Table 1 (item 4)
Roles and responsibilities	5a	Names, affiliations, and roles of protocol contributors	p1-2, p22
	5b	Name and contact information for the trial sponsor	Suppl. Table 1 (items 5, 6)
	5c	Role of study sponsor and funders, if any, in study design; collection, management, analysis, and interpretation of data; writing of the report; and the decision to submit the report for publication, including whether they will have ultimate authority over any of these activities	p22

For peer review only

	5d	Composition, roles, and responsibilities of the coordinating centre, steering committee, endpoint adjudication committee, data management team, and other individuals or groups overseeing the trial, if applicable (see Item 21a for data monitoring committee)	Suppl. Table 3
**Introduction**			
Background and rationale	6a	Description of research question and justification for undertaking the trial, including summary of relevant studies (published and unpublished) examining benefits and harms for each intervention	p5
	6b	Explanation for choice of comparators	p5
Objectives	7	Specific objectives or hypotheses	Table 5, p18-19
Trial design	8	Description of trial design including type of trial (eg, parallel group, crossover, factorial, single group), allocation ratio, and framework (eg, superiority, equivalence, noninferiority, exploratory)	p8
**Methods: Participants, interventions, and outcomes**			
Study setting	9	Description of study settings (eg, community clinic, academic hospital) and list of countries where data will be collected. Reference to where list of study sites can be obtained	p8
Eligibility criteria	10	Inclusion and exclusion criteria for participants. If applicable, eligibility criteria for study centres and individuals who will perform the interventions (eg, surgeons, psychotherapists)	Suppl. Table 1 (item 14)
Interventions	11a	Interventions for each group with sufficient detail to allow replication, including how and when they will be administered	p8-9, Table 4
	11b	Criteria for discontinuing or modifying allocated interventions for a given trial participant (eg, drug dose change in response to harms, participant request, or improving/worsening disease)	p9
	11c	Strategies to improve adherence to intervention protocols, and any procedures for monitoring adherence (eg, drug tablet return, laboratory tests)	p9

For peer review only - http://bmjopen.bmj.com/site/about/guidelines.xhtml

	11d	Relevant concomitant care and interventions that are permitted or prohibited during the trial	p9
Outcomes	12	Primary, secondary, and other outcomes, including the specific measurement variable (eg, systolic blood pressure), analysis metric (eg, change from baseline, final value, time to event), method of aggregation (eg, median, proportion), and time point for each outcome. Explanation of the clinical relevance of chosen efficacy and harm outcomes is strongly recommended	Table 1, Suppl. Table 2
Participant timeline	13	Time schedule of enrolment, interventions (including any run-ins and washouts), assessments, and visits for participants. A schematic diagram is highly recommended (see Figure)	Table 4
Sample size	14	Estimated number of participants needed to achieve study objectives and how it was determined, including clinical and statistical assumptions supporting any sample size calculations	p13
Recruitment	15	Strategies for achieving adequate participant enrolment to reach target sample size	p13
**Methods: Assignment of interventions (for controlled trials)**			
Allocation:			
Sequence generation	16a	Method of generating the allocation sequence (eg, computer-generated random numbers), and list of any factors for stratification. To reduce predictability of a random sequence, details of any planned restriction (eg, blocking) should be provided in a separate document that is unavailable to those who enrol participants or assign interventions	p13
Allocation concealment mechanism	16b	Mechanism of implementing the allocation sequence (eg, central telephone; sequentially numbered, opaque, sealed envelopes), describing any steps to conceal the sequence until interventions are assigned	p13
Implementation	16c	Who will generate the allocation sequence, who will enrol participants, and who will assign participants to interventions	p13, p8
Blinding (masking)	17a	Who will be blinded after assignment to interventions (eg, trial participants, care providers, outcome assessors, data analysts), and how	p13
	17b	If blinded, circumstances under which unblinding is permissible, and procedure for revealing a participant's allocated intervention during the trial	p13

For peer review only - http://bmjopen.bmj.com/site/about/guidelines.xhtml

Methods: Data collection, management, and analysis

Data collection methods	18a	Plans for assessment and collection of outcome, baseline, and other trial data, including any related processes to promote data quality (eg, duplicate measurements, training of assessors) and a description of study instruments (eg, questionnaires, laboratory tests) along with their reliability and validity, if known. Reference to where data collection forms can be found, if not in the protocol	p15-17, Table 4, Suppl. Table 1 (item 8), Suppl. Table 2
	18b	Plans to promote participant retention and complete follow-up, including list of any outcome data to be collected for participants who discontinue or deviate from intervention protocols	p9
Data management	19	Plans for data entry, coding, security, and storage, including any related processes to promote data quality (eg, double data entry; range checks for data values). Reference to where details of data management procedures can be found, if not in the protocol	p15-17, Suppl. Table 1 (item 8)
Statistical methods	20a	Statistical methods for analysing primary and secondary outcomes. Reference to where other details of the statistical analysis plan can be found, if not in the protocol	p16-17, Table 5, Suppl. Table 1 (item 8)
	20b	Methods for any additional analyses (eg, subgroup and adjusted analyses)	p16-17, Table 5
	20c	Definition of analysis population relating to protocol non-adherence (eg, as randomised analysis), and any statistical methods to handle missing data (eg, multiple imputation)	p16-17

Methods: Monitoring

Data monitoring	21a	Composition of data monitoring committee (DMC); summary of its role and reporting structure; statement of whether it is independent from the sponsor and competing interests; and reference to where further details about its charter can be found, if not in the protocol. Alternatively, an explanation of why a DMC is not needed	p22, Suppl. Table 1 (item 8), Suppl . Table 3
	21b	Description of any interim analyses and stopping guidelines, including who will have access to these interim results and make the final decision to terminate the trial	p13, Suppl. Table 3
Harms	22	Plans for collecting, assessing, reporting, and managing solicited and spontaneously reported adverse events and other unintended effects of trial interventions or trial conduct	p20, Suppl. Table 4, Suppl. Figure 1

Auditing	23	Frequency and procedures for auditing trial conduct, if any, and whether the process will be independent from investigators and the sponsor	p20
**Ethics and dissemination**			
Research ethics approval	24	Plans for seeking research ethics committee/institutional review board (REC/IRB) approval	p3, p20, Suppl. Table 1 (item 21)
Protocol amendments	25	Plans for communicating important protocol modifications (eg, changes to eligibility criteria, outcomes, analyses) to relevant parties (eg, investigators, REC/IRBs, trial participants, trial registries, journals, regulators)	p20
Consent or assent	26a	Who will obtain informed consent or assent from potential trial participants or authorised surrogates, and how (see Item 32)	p15
	26b	Additional consent provisions for collection and use of participant data and biological specimens in ancillary studies, if applicable	N/A
Confidentiality	27	How personal information about potential and enrolled participants will be collected, shared, and maintained in order to protect confidentiality before, during, and after the trial	p15-p16
Declaration of interests	28	Financial and other competing interests for principal investigators for the overall trial and each study site	p22
Access to data	29	Statement of who will have access to the final trial dataset, and disclosure of contractual agreements that limit such access for investigators	p3, p16, Suppl. Table 1 (item 24)
Ancillary and post-trial care	30	Provisions, if any, for ancillary and post-trial care, and for compensation to those who suffer harm from trial participation	p20
Dissemination policy	31a	Plans for investigators and sponsor to communicate trial results to participants, healthcare professionals, the public, and other relevant groups (eg, via publication, reporting in results databases, or other data sharing arrangements), including any publication restrictions	p3, p20
	31b	Authorship eligibility guidelines and any intended use of professional writers	p22
	31c	Plans, if any, for granting public access to the full protocol, participant-level dataset, and statistical code	p3, p20, Suppl. Table 1 (item 24)

For peer review

Appendices

Informed consent materials	32	Model consent form and other related documentation given to participants and authorised surrogates	Suppl. Materials: Patient Information and Consent Sheet
Biological specimens	33	Plans for collection, laboratory evaluation, and storage of biological specimens for genetic or molecular analysis in the current trial and for future use in ancillary studies, if applicable	N/A

*It is strongly recommended that this checklist be read in conjunction with the SPIRIT 2013 Explanation & Elaboration for important clarification on the items. Amendments to the protocol should be tracked and dated. The SPIRIT checklist is copyrighted by the SPIRIT Group under the Creative Commons "Attribution-NonCommercial-NoDerivs 3.0 Unported" license.

For peer review only

The SPIRIT-PRO Protocol Guidance Checklist

Protocol Section	SPIRIT-PRO Item	Recommended Content	Page Addressed
**Administrative Information**			
Roles and responsibilities	SPIRIT-5a-PRO Elaboration	Specify the individual(s) responsible for the PRO content of the trial protocol.	p22
**Introduction**			
Background and rationale	SPIRIT-6a-PRO Extension	Describe the PRO-specific research question and rationale for PRO assessment and summarize PRO findings in relevant studies.	p5, p11
Objectives	SPIRIT-7-PRO Extension	State specific PRO objectives or hypotheses (including relevant PRO concepts/domains).	p5, p13, Table 5, Suppl. Table 2
**Methods: Participants, Interventions, and Outcomes**			
Eligibility criteria	SPIRIT-10-PRO Extension	Specify any PRO-specific eligibility criteria (eg, language/reading requirements or pre-randomization completion of PRO). If PROs will not be collected from the entire study sample, provide a rationale and describe the method for obtaining the PRO subsample.	Suppl. Table 1 (item 14)
Outcomes	SPIRIT-12-PRO Extension	Specify the PRO concepts/domains used to evaluate the intervention (eg, overall health-related quality of life, specific domain, specific symptom) and, for each one, the analysis metric (eg, change from baseline, final value, time to event) and the principal time point or period of interest.	Table 1, Table 4, Table 5
Participant timeline	SPIRIT-13-PRO Extension	Include a schedule of PRO assessments, providing a rationale for the time points, and justifying if the initial assessment is not pre-randomization. Specify time windows, whether PRO collection is prior to clinical assessments, and, if using multiple questionnaires, whether order of administration will be standardized.	Table 4
Sample size	SPIRIT-14-PRO Extension	When a PRO is the primary end point, state the required sample size (and how it was determined) and recruitment target (accounting for expected loss to follow-up). If sample size is not established based on the PRO end point, then discuss the power of the principal PRO analyses.	p13

Calvert M, Kyte D, Mercieca-Bebber R, et al. Guidelines for Inclusion of Patient-Reported Outcomes in Clinical Trial Protocols: The SPIRIT-PRO Extension. *JAMA*. 018;319(5):483-494. doi:10.1001/jama.2017.21903

Note: The SPIRIT-PRO Extension should be used with the SPIRIT 2013 Statement and any other relevant SPIRIT Extensions, found at spirit-statement.org

For peer review only

The SPIRIT-PRO Protocol Guidance Checklist

Protocol Section	SPIRIT-PRO Item	Recommended Content	Page Addressed
**Methods: Data Collection, Management, and Analysis**			
Data collection methods	SPIRIT-18a(i)-PRO Extension	Justify the PRO instrument to be used and describe domains, number of items, recall period, and instrument scaling and scoring (eg, range and direction of scores indicating a good or poor outcome). Evidence of PRO instrument measurement properties, interpretation guidelines, and patient acceptability and burden should be provided or cited if available, ideally in the population of interest. State whether the measure will be used in accordance with any user manual and specify and justify deviations if planned.	Suppl. Table 2
	SPIRIT-18a(ii)-PRO Extension	Include a data collection plan outlining permitted mode(s) of administration (eg, paper, telephone, electronic, other) and setting (eg, clinic, home, other).	p15-16
	SPIRIT-18a(iii)-PRO Extension	Specify whether more than 1 language version will be used and state whether translated versions have been developed using currently recommended methods.	Suppl. Table 1 (item 14)
	SPIRIT-18a(iv)-PRO Extension	When the trial context requires someone other than a trial participant to answer on his or her behalf (a proxy-reported outcome), state and justify the use of a proxy respondent. Provide or cite evidence of the validity of proxy assessment if available.	Table 1, Table 4
	SPIRIT-18b(i)-PRO Extension	Specify PRO data collection and management strategies for minimizing avoidable missing data.	p9, p13, p15-16, p20
	SPIRIT-18b(ii)-PRO Elaboration	Describe the process of PRO assessment for participants who discontinue or deviate from the assigned intervention protocol.	p9
Statistical methods	SPIRIT-20a-PRO Elaboration	State PRO analysis methods, including any plans for addressing multiplicity/ type I (α) error.	p16-17
	SPIRIT-20c-PRO Elaboration	State how missing data will be described and outline the methods for handling missing items or entire assessments (eg, approach to imputation and sensitivity analyses).	p16-17
**Methods: Monitoring**			
Harms	SPIRIT-22-PRO Extension	State whether or not PRO data will be monitored during the study to inform the clinical care of individual trial participants and, if so, how this will be managed in a standardized way. Describe how this process will be explained to participants; eg, in the participant information sheet and consent form.	p13, p16, p20, Suppl. Table 3, Suppl. Materials: Patient

Calvert M, Kyte D, Mercieca-Bebber R, et al. Guidelines for Inclusion of Patient-Reported Outcomes in Clinical Trial Protocols: The SPIRIT-PRO Extension. *JAMA*. 018;319(5):483-494. doi:10.1001/jama.2017.21903

Note: The SPIRIT-PRO Extension should be used with the SPIRIT 2013 Statement and any other relevant SPIRIT Extensions, found at spirit-statement.org

For peer review only - http://bmjopen.bmj.com/site/about/guidelines.xhtml

The SPIRIT-PRO Protocol Guidance Checklist

			Information and Consent Sheet

Calvert M, Kyte D, Mercieca-Bebber R, et al. Guidelines for Inclusion of Patient-Reported Outcomes in Clinical Trial Protocols: The SPIRIT-PRO Extension. *JAMA*. 018;319(5):483-494. doi:10.1001/jama.2017.21903

Note: The SPIRIT-PRO Extension should be used with the SPIRIT 2013 Statement and any other relevant SPIRIT Extensions, found at spirit-statement.org

For peer review only
